# Reduction in Inflammatory Gene Expression in Skeletal Muscle from Roux-en-Y Gastric Bypass Patients Randomized to Omentectomy

**DOI:** 10.1371/journal.pone.0028577

**Published:** 2011-12-16

**Authors:** Robyn A. Tamboli, Tahar Hajri, Aixiang Jiang, Pamela A. Marks-Shulman, D. Brandon Williams, Ronald H. Clements, Willie Melvin, Benjamin P. Bowen, Yu Shyr, Naji N. Abumrad, Charles Robb Flynn

**Affiliations:** 1 Department of Surgery, Vanderbilt University School of Medicine, Nashville, Tennessee, United States of America; 2 Department of Cancer Biostatistics, Vanderbilt University School of Medicine, Nashville, Tennessee, United States of America; 3 Department of GTL Bioenergy and Structural Biology, Life Sciences Division, Lawrence Berkeley National Laboratory, Berkeley, California, United States of America; Charité-Universitätsmedizin Berlin, Germany

## Abstract

**Objectives:**

To examine the effects of Roux-en-Y gastric bypass (RYGB) surgery with and without laparoscopic removal of omental fat (omentectomy) on the temporal gene expression profiles of skeletal muscle.

**Design:**

Previously reported were the whole-body metabolic effects of a randomized, single-blinded study in patients receiving RYGB surgery stratified to receive or not receive omentectomy. In this follow up study we report on changes in skeletal muscle gene expression in a subset of 21 patients, for whom biopsies were collected preoperatively and at either 6 months or 12 months postoperatively.

**Methodology/Principal Findings:**

RNA isolated from skeletal muscle biopsies of 21 subjects (8 without omentectomy and 13 with omentectomy) taken before RYGB or at 6 and 12 months postoperatively were subjected to gene expression profiling via Exon 1.0 S/T Array and Taqman Low Density Array. Robust Multichip Analysis and gene enrichment data analysis revealed 84 genes with at least a 4-fold expression difference after surgery. At 6 and 12 months the RYGB with omentectomy group displayed a greater reduction in the expression of genes associated with skeletal muscle inflammation (ANKRD1, CDR1, CH25H, CXCL2, CX3CR1, IL8, LBP, NFIL3, SELE, SOCS3, TNFAIP3, and ZFP36) relative to the RYGB non-omentectomy group. Expressions of IL6 and CCL2 were decreased at all postoperative time points. There was differential expression of genes driving protein turnover (IGFN1, FBXW10) in both groups over time and increased expression of PAAF1 in the non-omentectomy group at 12 months. Evidence for the activation of skeletal muscle satellite cells was inferred from the up-regulation of HOXC10. The elevated post-operative expression of 22 small nucleolar RNAs and the decreased expression of the transcription factors JUNB, FOS, FOSB, ATF3 MYC, EGR1 as well as the orphan nuclear receptors NR4A1, NR4A2, NR4A3 suggest dramatic reorganizations at both the cellular and genetic levels.

**Conclusions/Significance:**

These data indicate that RYGB reduces skeletal muscle inflammation, and removal of omental fat further amplifies this response.

**Trial Registration:**

ClinicalTrials.gov NCT00212160

## Introduction

Obesity is an important risk factor for prevalent chronic health complications, such as type 2 diabetes (T2D) and cardiovascular disease. At the nexus of obesity-related co-morbidities is insulin resistance, which is characterized by a reduced responsiveness of insulin-sensitive tissues such as skeletal muscle, adipose tissue and liver to insulin-mediated glucose and lipid metabolism. Insulin resistance in skeletal muscle is considered especially pathogenic, as this tissue accounts for the majority of insulin-stimulated glucose disposal [1]. Accumulation of intramuscular lipid and excess plasma free fatty acid levels are considered central to aberrant skeletal muscle insulin signaling [2]. The pro-inflammatory state associated with obesity [3] is also implicated as a factor in skeletal muscle insulin resistance [4], [5].

Expanded visceral fat, as opposed to subcutaneous fat, is more strongly associated with insulin resistance and various comorbidities, especially cardiovascular disease, hypertension and hyperlipidemia [6], [7], [8], [9], [10]; it is also considered an important source of systemic free fatty acid (FFA) overload and inflammation due to enhanced lipolysis, cytokine secretion [11], [12], and macrophage infiltration [13]. Based on the available literature, it is clear that visceral fat is associated with insulin resistance. Our recent findings show that removal of the omentum with RYGB does not impart any additional benefits on hepatic insulin sensitivity or on insulin induced peripheral (muscle) glucose utilization [14]. However, these observations do not rule out any beneficial effects of removal of visceral fat on other muscle-mediated variables that could influence muscle glucose utilization. Recently, Varma *et al.* provided evidence that pro-inflammatory macrophages infiltrate skeletal muscle of obese, insulin-resistant humans and are activated by fatty acids [15], suggesting that local inflammation might be causative of skeletal muscle insulin resistance. Elevated proinflammatory cytokines such as TNF-α as well as increased proinflammatory pathway activation such as NFκB signaling through IκB kinase (IKK) and JNK-mediated phosphorylation of IRS-1 are also observed in various models of obesity induced insulin resistance [16], [17], [18], [19]. These observations suggest local inflammation in muscle as a possible mechanism by which the effects of insulin may be governed.

RYGB surgery results in 40% weight loss and resolution of T2D in 80% of patients by one year after surgery [20]; additionally, skeletal muscle insulin sensitivity is improved ∼two-fold [14], [21]. Relatively few studies have examined the effect of RYGB on muscle lipid content [22], [23] and insulin signaling proteins [24], [25]. One study revealed a differential expression of genes involved in insulin signaling (growth factor receptor-bound protein 14; GRB14), triglyceride synthesis (glycerol-3-phosphate dehydrogenase 1; GPD1), and muscle mass (myostatin; GDF8) by performing microarray analysis on muscle biopsies from 3 subjects obtained before and 12 months after gastric bypass surgery [26]. In the current study, we report the effects of RYGB combined with omental fat removal on skeletal muscle glucose utilization and on gene expression profiles, especially those related to the inflammatory pathways. Serial skeletal muscle biopsies and hyperinsulinemic-euglycemic clamps were performed preoperatively and at 6 and/or 12 months post-RYGB in 21 obese subjects undergoing RYGB and randomized to omentectomy.

## Materials and Methods

### Ethics Statement

All subjects provided written, informed consent before participating in this study, which was approved by the Health Sciences Committee of the Vanderbilt University Institutional Review Board.

### Subjects

**Figure 1:**
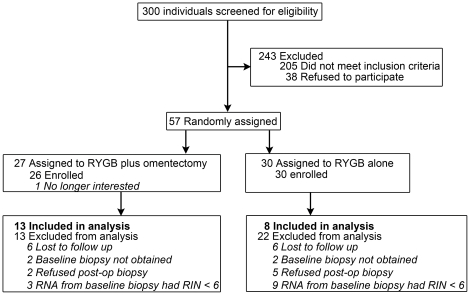
CONSORT Diagram.

Obese men and women between 18 and 60 years old, with and without T2D, and with physician's approval for RYGB, were recruited from the Center for Surgical Weight Loss at Vanderbilt University Medical Center (Nashville, TN). The cohorts consisted of 13 subjects receiving RYGB surgery plus omentectomy (11 women and 2 men, 44±8 years old, 7 with T2D) and 8 subjects receiving RYGB surgery alone (7 women and 1 man, 40±11 years old, 6 with T2D, see **Figure 1** and **Table 1**). Diabetic subjects using oral anti-diabetic agents were asked to discontinue all medications for 5 days prior to each study visit.

**Table 1:** Effect of Roux-en-Y Gastric Bypass surgery with or without omentectomy on body composition and metabolic variables.

Category	Before surgery		6 months post-op		12 months post-op		P value		
Omentectomy (n)	No (8)	Yes (13)	No (7)	Yes (12)	No (8)	Yes (8)	Group	G×T	Time
Gender (F/M)	7/1	11/2	6/1	10/2	7/1	6/2			
Ethnicity (Cau/AfAm)	6/2	9/4							
Diabetes (N/Y)	2/6	6/7	2/5	5/7	2/6	3/5			
Weight (kg)	129±22	137±17	97±16	98±13	87±16	89±8	0.311	0.191	<0.001
Body mass index (kg/m^2^)	46.3±6.2	49.6±8.6	34.3±4.8	34.8±5.7	31.1±4.5	31.8±4.7	0.291	0.174	<0.001
Fat mass (kg)	62±13	62±11	42±12	40±9	33±11	34±8	0.924	0.691	<0.001
Lean mass (kg)	64±12	67±11	53±14	54±9	50±11	52±8	0.538	0.353	<0.001
Body fat (%)	48±5	47±5	43±7	42±6	38±8	39±7	0.661	0.478	<0.001
Glucose (mg/dL)	141±52	133±44	93±15	96±10	92±13	107±50	0.601	0.455	<0.001
Insulin (mU/L)	29±14	24±12	8 ± 5	8±3	8±4	7±4	0.239	0.504	<0.001
Glycosylated hemoglobin (%)	6.3±0.7	6.6±0.8	5.7±0.8	5.8±0.5	5.7±0.5	6.0±1.4	0.706	0.843	<0.001
Free fatty acids (mmol/L)	0.64±0.12	0.70±0.23	0.65±0.23	0.60±0.15	0.53±0.08	0.58±0.16	0.731	0.905	0.054
Total triglyceride (mg/dL)	155±67	148±91	94±43	83±29	83±35	81±25	0.723	0.679	<0.001
Leptin (ng/mL)	36±13	35±10	20±12	16±5	17±12	13±9	0.701	0.507	<0.001
Total adiponectin (µg/mL)	7.5±5.0	8.1±6.6	9.8±3.7	11.0±4.3	18.6±9.7	13.4±8.3	0.508	0.096	<0.001
M (mg/kg·min)	4.4±1.8	4.8±1.3	6.9±2.0	8.6±1.5	8.9±1.9	9.5±2.2	0.287	0.917	<0.001
M/I (mg·mL/kg·min·mU)	16.0±9.3	21.1±13.9	32.8±13.5	35.3±13.3	36.8±14.5	37.2±10.4	0.367	0.595	<0.001

Values are means ± SD; P values for the main effects of group and time and their interaction (G×T) are reported. M, glucose disposal rate per kg of body weight; M/I, insulin sensitivity index (M-value per unit of plasma insulin).

### Study Protocol

We conducted a 12-month randomized, controlled trial wherein subjects underwent RYGB with or without omentectomy. Studies were conducted preoperatively and at 6 and 12 months post-RYGB at the Vanderbilt Clinical Research Center (CRC) between March 2005 and December 2008. The protocol for this trial and the CONSORT checklist are available as supporting information (**Protocol S1** and **Checklist S1**). Randomization was conducted using a computer generated code with a permuted block size of four. Separate randomization codes were used for Caucasian females, Caucasian males, African American females, and African American males to achieve equal gender and ethnic distributions in each group. The study coordinator generated the random allocation sequence and notified the surgeon at the beginning of the surgical procedure of the patient's randomization category. All recruited participants and researchers involved in the conduct of the study and sample analysis were blinded to treatment. The primary outcome measure in this study was insulin sensitivity as measured by the hyperinsulinemic euglycemic clamp [14], and a secondary outcome measure was gene expression in skeletal muscle biopsies. Enrollment was concluded when we obtained sufficient power for the primary outcome measure.

Muscle insulin sensitivity was estimated using a hyperinsulinemic euglycemic clamp as the rate of exogenous dextrose (20%) infusion to maintain euglycemia (plasma glucose of 90–100 mg/dl) during a high-dose insulin infusion (2–3 mU/kg.min). The clamp studies were performed in all subjects at three time points: (a) In the preoperative period, at least one week prior to the surgical procedure, and at (b) 6-months and (c) 12 months postoperatively. Muscle biopsies were acquired from the *vastus lateralis* muscle using a Bergstrom needle as previously described [14]. Basal (prior to RYGB) muscle biopsies were obtained from all subjects (n = 21) at the time of the surgical procedure, immediately after adequate induction of general anesthesia and prior to initiation of the RYGB procedure. The postoperative muscle biopsies were obtained on the CRC on the day of and prior to initiation of the insulin clamp, using 1% Lidocaine solution as local anesthetic. Fourteen subjects had muscle biopsies at the three specified time points (pre-RYGB and at 6 and 12 months post-RYGB); 5 subjects had muscle biopsies obtained during the basal period and at 6 months post-RYGB, and 2 subjects had biopsies performed during the basal period and at 12 months post-RYGB. Biopsy specimen were dissected free of fat and vasculature, blotted free of blood and immediately snap frozen in liquid nitrogen and stored at −80°C until analyzed. The surgical procedure consisted of a laparoscopic RYGB surgery, in which a small gastric pouch (∼25 ml) was created and a Roux-en-Y gastrojejunostomy with a 30- to 50-cm biliopancreatic limb and a 100- to 200-cm Roux limb was constructed. In patients randomized to receive an omentectomy, surgeons resected an average of 0.74 kg of greater omentum [14].

Blood was collected and assays were performed as previously described [14]. The inflammatory cytokines monocyte chemotactic protein-1 (MCP-1), interleukin (IL)-1β, IL-6, IL-8, IL-10, and tumor necrosis factor (TNF)-α were measured by multiplex BD™ Cytokine Bead Array combined with flow cytometry (BD Biosciences, San Diego, California). Fat and lean body mass were assessed with dual-energy x-ray absorptiometry (GE Lunar Prodigy, Madison, WI) using half-body scans[27].

### RNA isolation

Skeletal muscle biopsies were chopped into small pieces at −20°C and 1 ml Trizol (Invitrogen, Carlsbad, CA) was added per 100 mg of sample. RNA was extracted using Qiagen's RNase-free DNase Set (Valencia, CA) supplied protocol with the following modification. RNA was precipitated with 0.25 volumes isopropanol (Fisher Scientific, Fairlawn, NJ) and 0.25 volumes of RNA precipitation solution (7% NaCl/21% disodium citrate). DNase treatment was performed by adding 10 µl DNase and 70 µl Buffer RDD (Qiagen) per 100 µl sample. Trizol isolation was repeated once or twice to remove the DNase and any remaining fats or salts. RNA was re-suspended in RNase/DNase free water.RNA quality was assessed by the Vanderbilt Microarray Shared Resource on an Agilent 2100 Bioanalyzer utilizing RNA integrity numbers (RINs), 260/280 absorption and 28 s∶18 s ratios [28]. RNA samples included in this experiment exceeded the recommended RIN of 6, with an average RIN of 7.4.

### Generation of cDNA

To minimize costs, initial gene expression profiles of muscle RNA pools were determined using Affymetrix GeneChip Human Exon 1.0 ST arrays. Skeletal muscle RNA pools (pre-RYGB, 6 months post-op, and 12-months post-op) from RYGB subjects with and without omentectomy (6 pools total) were prepared from 42 different skeletal muscle RNA samples. RNA samples were prepared for microarray analysis using the standard Affymetrix protocol (Affymetrix Inc., Santa Clara, CA) and published techniques [29], [30]. Briefly, 200 ng pooled total RNA and poly-A spike in controls, were brought to 5 µl with nuclease free water. First Strand Synthesis Master mix was added to the sample mix and incubated at 42°C for 1 hr; enzymes were heat inactivated 10 minutes at 70°C, followed by a cooling to 4°C for 2 minutes. Subsequently, second strand synthesis master mix was added to the sample mix and incubated at 16°C for 2 hours. The reactions were heat-inactivated by 10 minute incubation at 75°C. The resulting cDNA was then processed in an IVT reaction to generate cRNA that was used in first strand synthesis reactions to generate targets of the correct sense for hybridization to the Affymetrix Exon arrays. The reactions were set up with 10 µg cRNA, random hexamer, and first strand synthesis reagents to make cDNA. The targets were then treated with RNAse to remove template RNA and cleaned up over kit supplied columns, or Agencourt SPRI beads, following manufacturer's protocol. A total of 5.5 µg of the clean cDNA target was then enzymatically fragmented and end-labeled using the Affymetrix kit reagents. The cRNA, cDNA, and fragmented and end-labeled targets were assessed by Agilent bioanalysis to ensure that the amplified targets met the recommended smear range, and that fragmentation and end-labeling were complete.

### Microarray Hybridization

The requisite amount of target was then added to hybridization cocktail to give a final target concentration of ∼25 ng/µl in the hybridization cocktail. The targets in hybridization cocktail were heat denatured at 99°C for 5 minutes, cooled to 45°C for 5 minutes, centrifuged and then loaded on the Affymetrix cartridge arrays for a hybridization period of 16 hours at 45°C, with rotation of 60RPM in the Affymetrix Model 645 Hybridization Oven. The cartridge arrays were washed, and stained per standard Affymetrix Protocols using Affymetrix Hybridization Wash and Stain kit reagents. After washing and staining, the arrays were scanned in an Affymetrix 7G plus scanner. The resulting data was acquired by GCOS software.

### Validation by Taqman Low Density Array

To validate the expression levels of probes from RNA analyses, the 42 individual RNA samples comprising the pool were analyzed using Taqman Low Density Array (TLDA) [31]. An additional 12 skeletal muscle RNA samples not previously analyzed by microarray (baseline and post-RYGB in seven additional subjects) also were included. Total RNA (500 ng) was reverse transcribed to ds cDNA using random primers, SuperScript II Reverse Transcriptase (Invitrogen, Carlsbad, CA) according to the supplied protocol. Following reverse transcription, each 20 µl reaction was diluted with 25 µl of DNase/RNase-free water. Quantitative PCR was then performed using 500 ng of cDNA per reaction well. Thirty-one experimental genes and one control gene were assayed by qPCR. Thirty-two gene specific assays were purchased from Applied Biosystems (Foster City, CA) as custom TLDA microfluidics cards (Format 32) with TLDA probes (**Table S3**) spotted in triplicate on 384 well plates. All reactions were carried out at the following thermocycling conditions: Step 1: 10 min at 95°C, Step 2: 40 cycles of 15 s at 95°C, and Step 3: 1 min at 60°C. An Applied Biosystems ABI 7900HT unit with automation attachment (Foster City, CA) was used for real-time PCR. This unit is capable of collecting spectral data at multiple points during a PCR run.

### Statistical Quality Control, Methods and Bioinformatics

The sample size for the current study was dictated by availability of a baseline and at least one postoperative muscle biopsy collected from the parent study [14]. The study power analysis was completed using the general linear model. A sample size of at least 7 patients per condition per time point, provided at least 90% power to detect an effect size of 4, i.e., 4-fold change, with two-sided false discovery rate (FDR) of 0.05.

**Figure 2:**
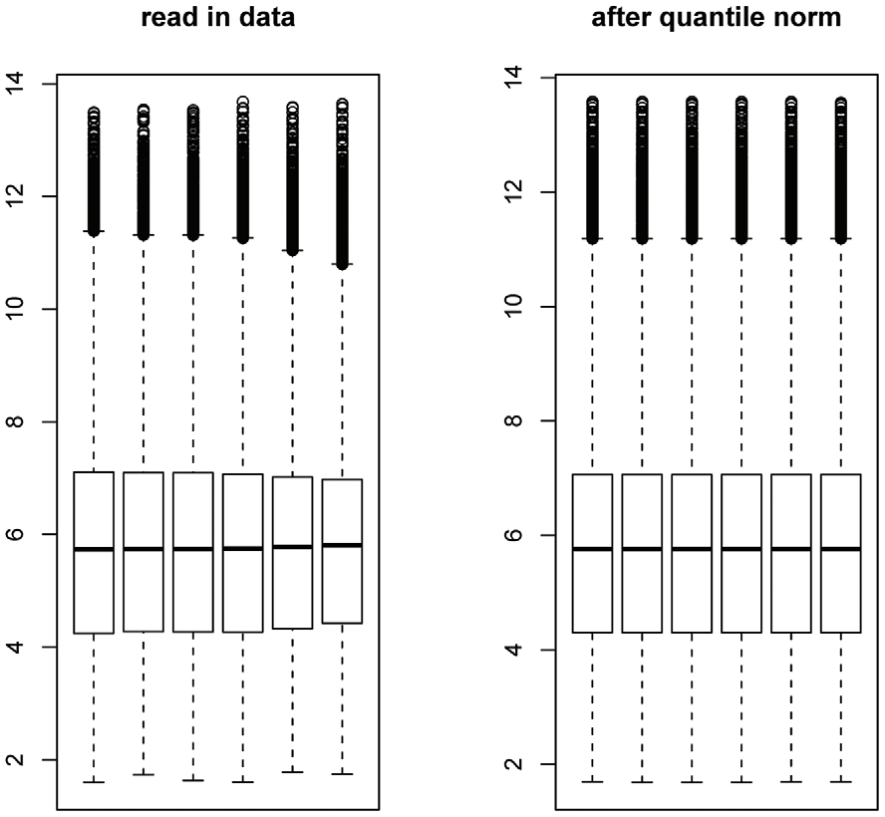
Box plot depicting the difference among microarray expression datasets (chips) before and after normalization by Robust Microarray Averaging (RMA). Boxes show the 25th and 75th percentiles in the distribution of log-transformed (log base 2) intensities. The median is the horizontal bar in the middle of the box. The whiskers (dotted lines extending from the boxes) illustrate the maximum value or 1.5 times the interquartile range of data (IQR), whichever is smaller. The circles display any points beyond these whiskers.

Anthropometric and metabolic parameters were analyzed for an effect of time and group (omentectomy) using linear mixed effects models in IBM SPSS version 18 (Chicago, IL). The overall intensities of all probes across the six microarrays were subjected to box plot analysis (**Figure 2**). Exon ST 1.0 array data pre-processing was done using GeneSpring v7.3 software (Agilent Technologies, Foster City, CA). Probe-level analysis was performed using the Robust Multichip Analysis (RMA) method [32], but the default quantile across-sample normalization method of RMA was not done in order to avoid over-correction for the small sample size. The expression values were in log_2_ format after RMA. The change between samples was calculated as the difference between the two expression values. Winner probes for each comparison were selected based on two stringency criteria: a cutoff of 2 fold (2,697 genes, used for global pathway analysis; **Table S1**) or 4-fold change (103 genes, subset selected for TLDA validation; **Table S2**). Relative quantitation and standard deviation calculations with TLDA data were performed by the comparative *C*_T_ method [33]. The 56 cDNA samples were assayed in triplicate for each gene of interest and the control. For each gene in each sample, the average *C*_T_ and the standard deviation were calculated, and the 18S control average *C*_T_ was subtracted from the gene average *C*_T_. The resulting value is the Δ*C*_T_. The standard deviation for the Δ*C*_T_ was calculated by taking the square root of the sum of the control and gene standard deviations. For comparisons to basal time points, the average Δ*C*_T_ for the basal period pools was subtracted from the respective average Δ*C*_T_ of the 6 and 12 months samples generating a ΔΔ*C*_T_. Fold changes between conditions are given by applying the expression 2^−ΔΔCT^, and ΔΔ*C*_T_ ± standard deviation applied to this expression gives the range. Correlation analysis was implemented using the “stat” package under R2.12.1 [34] without adjustment for multiple comparisons.

## Results

### Participant Flow, Recruitment, and Numbers Analyzed

Fifty-six of 300 screened subjects were enrolled in the trial (**Figure 1**). Twenty-one subjects completed all study components at all time points (pre-surgery, 6 and 12 month post-operative) and yielded a complement of muscle RNA samples of sufficient quality (average RIN of 7.4) to have expression analysis performed. The first participant was enrolled in March, 2005.

### Anthropometric and Metabolic Parameters

Both the omentectomy and non-omentectomy groups exhibited significant decreases in body weight, BMI, fat mass, and lean mass during the 12 months after RYGB; there was neither a group effect nor a group×time interaction (**Table 1**). Amount of initial weight lost was 28±3% in the first 6 months and 34±6% in 12 months following surgery across both groups. Similarly, fasting levels of glucose, insulin, triglycerides, leptin, and adiponectin were significantly decreased and free fatty acids were marginally decreased over time without a group or group×time effect. Insulin-stimulated glucose uptake in the muscle was significantly increased ∼2-fold by 12 months after RYGB in both groups, without significant differences between groups or group×time interactions.

### Circulating Inflammatory Markers

A main effect of time post-RYGB was detected for systemic concentrations of CRP and MCP-1 but not for IL-1β, IL-6, IL-8, IL-10, nor TNF-α. There was no effect of omentectomy or group×time interaction for any inflammatory cytokine (**Table 2**).

**Table 2:** Serological measures of inflammation determined before and after RYGB with and without omentectomy.

Category	Before surgery		6 months post-op		12 months post-op		P value		
Omentectomy	No	Yes	No	Yes	No	Yes	Group	G×T	Time
C-reactive protein (µg/mL)	11.0±9.1	9.3±11.0	2.4±1.4	3.2±3.6	2.8±3.8	1.0±0.5	0.716	0.983	<0.001
IL-1β (pg/ml)	2.9±4.6	18.6±38.5	2.2±1.2	6.2±10.5	1.9±1.4	16.2±34.5	0.194	0.119	0.06
IL-6 (pg/ml)	3.1±2.0	62.5±190.5	5.4±4.7	10.9±21.5	4.4±6.6	62.1±159.7	0.299	0.085	0.114
IL-8 (pg/ml)	3.7±2.1	18.6±45.4	3.3±1.4	8.5±9.0	3.8±1.7	19.6±38.8	0.265	0.246	0.263
IL-10 (pg/ml)	1.8±1.0	3.8±4.4	1.9±0.9	1.9±1.1	1.3±0.5	2.0±0.7	0.106	0.316	0.089
MCP-1 (pg/ml)	85.9±71.1	135.5±209.4	70.4±66.8	59.8±52.9	57.4±49.1	97.9±175.7	0.087	0.41	0.001
TNF-a (pg/ml)	1.8±1.7	12.1±23.3	1.8±1.0	4.7±7.4	1.2±0.6	9.4±15.7	0.123	0.18	0.119

### Overview of skeletal muscle gene expression patterns after RYGB with and without omentectomy

**Figure 3:**
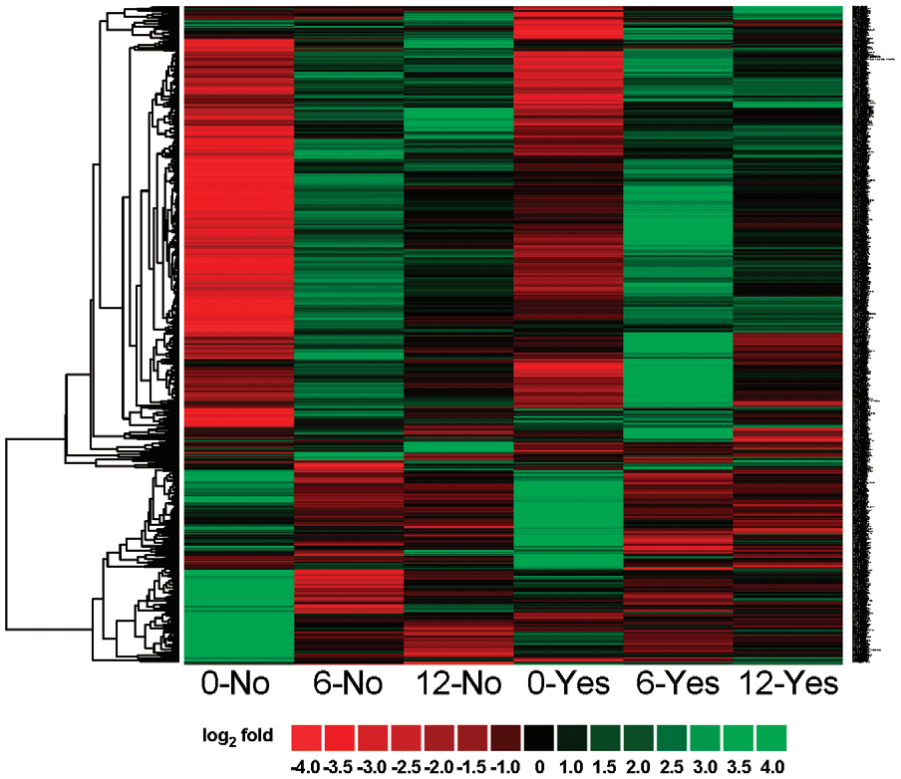
Heat map displaying hierarchical clustering results from microarray expression profile data derived from skeletal muscle RNA pools. Samples include 0, 6 and 12 months “No” omentectomy and 0, 6 and 12 months “Yes” omentectomy. The expression of 2,697 genes is represented as a dendrogram after applying filtering criteria for selecting genes with a log_2_ ratio intensity greater than 1 (ratio>2). Gene expression ratios are displayed by applying progressively brighter shades of red (down-regulated) or green (up-regulated) to log_2_ ratios that increasingly deviate from zero.

We compared skeletal muscle gene expression patterns between two surgical groups (RYGB surgery with no omentectomy vs. RYGB surgery with omentectomy) over time (6 vs. 0 months, and 12 vs. 0 months) in six skeletal muscle RNA sample pools derived by combining equal amounts of RNA from 7 subjects at each time point. For each time point, the observed gene expression value was compared to an overall average expression value derived from the union of all probe intensities (33,297) across all six Affymetrix Human Exon 1.0 ST Arrays. Probe intensities were then log_2_ transformed, and those genes expressed greater than 2 fold (log_1_ = 4 fold) within any time-point comparison were collated. In this manner, not all genes expressed were changed greater than 2-fold among all time-points. Expression levels of the 2,697 probes in each of the 6 microarrays changed greater than 2 fold after hierarchical clustering analysis is displayed graphically in **Figure 3** and individual expression values are listed in **Table S2**. While significance cannot be derived from these microarray hybridizations without experimental replication, general expression were noted. For example, groups without (No) and with (Yes) omentectomy displayed similar expression profiles at 0 months. Additionally, most genes downregulated at 0 month were upregulated at 6 months, and conversely most genes upregulated at 0 month were downregulated at 6 months. At 12 months in both the omentectomy and non-omentectomy groups, the overall abundance of genes changing more than 2-fold was diminished, with no apparent differences as a function of omentectomy.

### Skeletal muscle gene expression changes without omentectomy

To identify those genes with the greatest change in expression after RYGB we compared genes with greater than 2-fold (log_2_) changes in expression. **Table 3** lists those 25 genes that were differentially expressed comparing 6 vs. 0 months without omentectomy. The AP-1 transcription factors such as FOS, FOSB, JUNB as well as MYC and EGR1 were among those downregulated to the greatest degree at 6 months. These are immediate early genes acting on a specific biological response that directly induces or controls a variety of other genes [35]. HOXC10, a gene associated with muscle satellite cell activation, was the only transcription factor that was upregulated [36]. Genes regulating various cellular processes averaged a greater than 5-fold downregulation: *inflammation* (ANKRD1, CDR1, CCL2, IL6, MAOB, GADL1, ITLN1); *protein turnover* (IGFN1, FBXW10); and *extracellular matrix remodeling* (CYR61, THBS1, THBS4). The small nucleolar RNA C/D box genes SNORD115-44, SNORD25, and SNORD59B were among those genes which were upregulated at 6 months without omentectomy.

**Table 3:** Genes changed ≥4 fold (log 2 base ≥2) between 6 and 0 months no omentectomy.

Probe ID	Gene Symbol	Gene Description	Process	Cytoband	mRNA Accession	log2fold
7955858	HOXC10	homeobox C10	transcription factor	12q13.3	NM_017409	2.05587
8148317	MYC	v-myc myelocytomatosis viraloncogene homolog	transcription factor	8q24.21	NM_002467	−2.0047
8026047	JUNB	jun B proto-oncogene	transcription factor	19p13.2	NM_002229	−2.0214
8029693	FOSB	FBJ murine osteosarcoma viraloncogene homolog B	transcription factor	19q13.32	NM_006732	−2.17407
8108370	EGR1	early growth response 1	transcription factor	5q31.1	NM_001964	−2.5463
7975779	FOS	v-fos FBJ murine osteosarcoma viraloncogene homolog	transcription factor	14q24.3	NM_005252	−3.14739
7981990	PWCR1	Prader-Willi syndrome chromosome region 1	small nucleolar RNA expression	15q11.2	NR_003106	2.53618
7964246	SNORD59B	small nucleolar RNA, C/D box 59B	small nucleolar RNA expression	12q13.3	NR_003046	2.49785
7982094	SNORD115-44	small nucleolar RNA, C/D box 115-44	small nucleolar RNA expression	15q11.2	NR_003359	2.43735
7948910	SNORD25	small nucleolar RNA, C/D box 25	small nucleolar RNA expression	11q13	NR_002565	2.09656
8012949	FBXW10	F-box and WD repeat domaincontaining 10, mRNA	protein turnover	17p12	ENST00000308799	2.30938
7908650	IGFN1	eEF1A2 binding protein	protein turnover	1q32.1	NM_178275	−2.25232
8090872	KY	kyphoscoliosis peptidase	other	3q22.2	NM_178554	2.06643
8175311	CXorf48	chromosome X open reading frame48 transcript variant 2	other	Xq26.3	ENST00000344129	−2.09328
8156848	NR4A3	nuclear receptor subfamily 4, group A, member 3	orphan receptor	9q22	NM_173198	−2.75007
8085972	GADL1	glutamate decarboxylase-like 1	insulin signaling	3p24.1-p23	NM_207359	2.26062
7921690	ITLN1	intelectin 1 (galactofuranose binding) (omentin-1)	insulin signaling	1q22-q23.5	NM_017625	−2.40258
8172204	MAOB	monoamine oxidase B	inflammation	Xp11.23	NM_000898	2.08435
8131803	IL6	interleukin 6 (interferon, beta 2)	inflammation	7p21	NM_000600	−2.27324
8006433	CCL2	chemokine (C-C motif) ligand 2 (MCP-1)	inflammation	17q11.2-q12	NM_002982	−2.3367
8175531	CDR1	cerebellar degeneration-relatedprotein 1, 34kDa	inflammation	Xq27.1-q27.2	NM_004065	−2.51038
7934979	ANKRD1	ankyrin repeat domain 1 (cardiac muscle)	inflammation	10q23.31	NM_014391	−3.16087
7982597	THBS1	thrombospondin 1	extracellular matrix protein	15q15	NM_003246	−2.11267
8106573	THBS4	thrombospondin 4	extracellular matrix protein	5q13	NM_003248	−2.23557
7902687	CYR61	cysteine-rich, angiogenic inducer, 61	extracellular matrix protein	1p31-p22	NM_001554	−2.78682

**Table 4** lists the 36 genes that were most changed at 12 vs. 0 months in the RYGB without omentectomy cohort underscoring the durability of these responses. Several of the genes downregulated at 6 vs. 0 months without omentectomy also were observed at this time point, including the AP-1 transcription factors, markers of satellite cell activation, genes facilitating the inflammatory response, (CCL2, CDR1, and IL6), and the orphan receptor NR4A3, as well as genes regulating extracellular matrix formation and protein turnover. Additionally, over 20 genes regulating small nucleolar RNA (snoRNA) expression, mostly from the C/D box 115 group, were upregulated. SnoRNAs are not transcribed into protein products; they influence the structure of other mRNAs and their encoded proteins. Eighteen microarray probes mapping to fifteen SNORD 115 transcripts derived from cytoband 15q11.2 were upregulated, as were SNORD29, SNORD44, SNORD54 and SNORA73A (from the H/ACA group of snoRNAs).

**Table 4:** Genes changed ≥4 fold (log 2 base ≥2) between 12 and 0 months no omentectomy.

Probe ID	Gene Symbol	Gene Description	Process	Cytoband	mRNA Accession	log2fold
7908650	IGFN1	eEF1A2 binding protein	protein turnover	1q32.1	NM_178275	−2.55556
8012949	FBXW10	F-box and WD repeat domaincontaining 10	protein turnover	17p12	ENST00000308799	2.06496
7942476	PAAF1	proteasomal ATPase-associated factor 1	protein turnover	11q13.4	NM_025155	2.01504
7921690	ITLN1	intelectin 1 (galactofuranosebinding) (omentin -1)	insulin signaling	1q22-q23.5	NM_017625	−2.23682
8108370	EGR1	early growth response 1	transcription factor	5q31.1	NM_001964	−3.05413
7975779	FOS	v-fos FBJ murine osteosarcoma viral oncogene homolog	transcription factor	14q24.3	NM_005252	−4.09474
8029693	FOSB	FBJ murine osteosarcoma viraloncogene homolog B	transcription factor	19q13.32	NM_006732	−2.12644
8026047	JUNB	jun B proto-oncogene	transcription factor	19p13.2	NM_002229	−2.38627
8148317	MYC	v-myc myelocytomatosis viraloncogene homolog	transcription factor	8q24.21	NM_002467	−2.0096
7955858	HOXC10	homeobox C10	transcription factor	12q13.3	NM_017409	2.0778
8006433	CCL2	chemokine (C-C motif) ligand 2	inflammation	17q11.2-q12	NM_002982	−2.09334
8175531	CDR1	cerebellar degeneration-relatedprotein 1, 34kDa	inflammation	Xq27.1-q27.2	NM_004065	−3.36346
8086344	CX3CR1	chemokine (C-X3-C motif) receptor 1	inflammation	3p21|3p21.3	NM_001337	2.12768
8131803	IL6	interleukin 6 (interferon, beta 2)	inflammation	7p21	NM_000600	−2.57781
8156848	NR4A3	nuclear receptor subfamily 4,group A, member 3	orphan receptor	9q22	NM_173198	−2.72922
7899480	SNORA73A	small nucleolar RNA, H/ACA box 73A	small nucleolar RNA expression	1p36.1	NR_002907	2.48545
7982038	SNORD115-1	small nucleolar RNA, C/D box 115-1	small nucleolar RNA expression	15q11.2	NR_001291	2.68633
7982078	SNORD115-11	small nucleolar RNA, C/D box 115-11	small nucleolar RNA expression	15q11.2	NR_003303	2.45315
7982024	SNORD115-12	small nucleolar RNA, C/D box 115-12	small nucleolar RNA expression	15q11.2	NR_003304	2.54694
7982050	SNORD115-12	small nucleolar RNA, C/D box 115-12	small nucleolar RNA expression	15q11.2	NR_003304	2.53634
7982084	SNORD115-12	small nucleolar RNA, C/D box 115-12	small nucleolar RNA expression	15q11.2	NR_003304	2.03653
7982008	SNORD115-13	small nucleolar RNA, C/D box 115-13	small nucleolar RNA expression	15q11.2	NR_003305	2.68633
7982032	SNORD115-16	small nucleolar RNA, C/D box 115-16	small nucleolar RNA expression	15q11.2	NR_003308	2.68633
7982046	SNORD115-20	small nucleolar RNA, C/D box 115-20	small nucleolar RNA expression	15q11.2	NR_003312	2.56052
7982052	SNORD115-23	small nucleolar RNA, C/D box 115-23	small nucleolar RNA expression	15q11.2	NR_003315	3.03821
7982056	SNORD115-25	small nucleolar RNA, C/D box 115-25	small nucleolar RNA expression	15q11.2	NR_003342	2.77049
7982058	SNORD115-26	small nucleolar RNA, C/D box 115-26	small nucleolar RNA expression	15q11.2	NR_003343	2.46435
7982028	SNORD115-43	small nucleolar RNA, C/D box 115-43	small nucleolar RNA expression	15q11.2	NR_003358	2.45315
7982094	SNORD115-44	small nucleolar RNA, C/D box 115-44	small nucleolar RNA expression	15q11.2	NR_003359	2.75753
7982016	SNORD115-5	small nucleolar RNA, C/D box 115-5	small nucleolar RNA expression	15q11.2	NR_003297	2.54694
7982018	SNORD115-6	small nucleolar RNA, C/D box 115-6	small nucleolar RNA expression	15q11.2	NR_003298	2.30491
7982090	SNORD115-6	small nucleolar RNA, C/D box 115-6	small nucleolar RNA expression	15q11.2	NR_003298	2.27704
7982020	SNORD115-7	small nucleolar RNA, C/D box 115-7	small nucleolar RNA expression	15q11.2	NR_003299	3.00484
7982092	SNORD115-9	small nucleolar RNA, C/D box 115-9	small nucleolar RNA expression	15q11.2	NR_003301	2.45315
7948902	SNORD29	small nucleolar RNA, C/D box 29	small nucleolar RNA expression	11q13	NR_002559	2.34649
7922410	SNORD44	small nucleolar RNA, C/D box 44	small nucleolar RNA expression	1q25.1	NR_002750	2.10829
8150877	SNORD54	small nucleolar RNA, C/D box 54	small nucleolar RNA expression	8q12	NR_002437	2.06555
7902687	CYR61	cysteine-rich, angiogenic inducer, 61	extracellular matrix	1p31-p22	NM_001554	−2.6069
8106573	THBS4	thrombospondin 4	extracellular matrix	5q13	NM_003248	−2.07002
8042788	ACTG2	actin, gamma 2, smooth muscle, enteric	other	2p13.1	NM_001615	−2.1841
7931832	AKR1C2	aldo-keto reductase family 1, member C2	other	10p15-p14	NM_205845	2.20199
7924821	gm127	similar to RIKEN 2610020C11	other	1q42.13	AF387613	−2.183
8048014	RPE	ribulose-5-phosphate-3-epimerase	other	2q32-q33.3	NM_199229	2.17662

### Skeletal muscle gene expression changes with omentectomy

At 6 vs. 0 months in the RYGB with omentectomy group, the number of genes differentially expressed in muscle was nearly double that observed in the RYGB without omentectomy cohort at the same time point. **Table 5** lists the 46 genes that expressed greater than a 4-fold change. Genes known to be involved in facilitating the inflammatory response were particularly responsive. In addition to ANKRD1, CCL2, and IL6 observed as being greater than 4-fold downregulated in the RYGB without omentectomy group, the inflammatory genes CH25H, CXCL2, SOCS3, IL8, LBP, NFIL3, SELE and TNFAIP3 also were downregulated. All of the transcription factors differentially expressed at 6 months in the non-omentectomy group were also downregulated in the 6 months with omentectomy group, as was ATF3. Adiponectin (ADIPOQ) and the glucose transporter (SLC2A3) genes along with the extracellular matrix proteins ADAM metallopeptidase 1 and 4 (ADAMTS1 and ADAMTS4), CYR61, CD54, THBD and THBS1 were all downregulated; however a handful of genes with varied functions (ATRX, DLEU2, ZNF780B, YIPF7, RWDD3, ANGPT1) were upregulated. All 3 members of the nuclear orphan nuclear receptor family 4 (NRFA1-3) recently identified as controlling skeletal muscle metabolism [37], [38] were downregulated; in particular NR4A3 which was downregulated over 12 fold. Similar phenomena were observed in the 12 vs. 0 months with omentectomy group as were found at 6 vs. 0 months with omentectomy group. A subset of other proteins with varying functions complete the list of genes with differential expression values greater than 4 fold; in total 36 genes were observed (**Table 6** **)**.

**Table 5:** Genes changed ≥4 fold (log 2 base ≥2) between 6 and 0 months with omentectomy.

Probe ID	Gene Symbol	Gene Description	Process	Cytoband	mRNA Accession	log2fold
7908650	IGFN1	eEF1A2 binding protein	protein turnover	1q32.1	NM_178275	−3.10661
8108370	EGR1	early growth response 1	transcription factor	5q31.1	NM_001964	−4.47459
7975779	FOS	v-fos FBJ murine osteosarcoma viraloncogene homolog	transcription factor	14q24.3	NM_005252	−5.12292
8029693	FOSB	FBJ murine osteosarcoma viraloncogene homolog B	transcription factor	19q13.32	NM_006732	−2.78581
8026047	JUNB	jun B proto-oncogene	transcription factor	19p13.2	NM_002229	−3.56001
8148317	MYC	v-myc myelocytomatosis viraloncogene homolog (avian)	transcription factor	8q24.21	NM_002467	−3.08841
7909610	ATF3	activating transcription factor 3	transcription factor	1q32.3	NM_001040619	−2.23226
8084710	ADIPOQ	adiponectin, C1Q and collagen domain containing	insulin signaling	3q27	NM_004797	−2.25931
7960865	SLC2A3	solute carrier family 2 (facilitated glucose transporter), 3	insulin signaling	12p13.3	NM_006931	−2.60975
7934979	ANKRD1	ankyrin repeat domain 1 (cardiac muscle)	inflammation	10q23.31	NM_014391	−2.57698
8006433	CCL2	chemokine (C-C motif) ligand 2 (MCP-1)	inflammation	17q11.2-q12	NM_002982	−3.85999
7934916	CH25H	cholesterol 25-hydroxylase	inflammation	10q23	NM_003956	−2.56701
8100994	CXCL2	chemokine (C-X-C motif) ligand 2 (MIP-2a)	inflammation	4q21	NM_002089	−2.61796
8131803	IL6	interleukin 6 (interferon, beta 2)	inflammation	7p21	NM_000600	−5.01826
8018864	SOCS3	suppressor of cytokine signaling 3	inflammation	17q25.3	NM_003955	−3.40317
8095680	IL8	interleukin 8	inflammation	4q13-q21	NM_000584	−2.02015
8062461	LBP	lipopolysaccharide binding protein	inflammation	20q11.23-q12	NM_004139	−2.16848
8162276	NFIL3	nuclear factor, interleukin 3 regulated	inflammation	9q22	NM_005384	−2.63846
7922229	SELE	selectin E (endothelial adhesion molecule 1)	inflammation	1q22-q25	NM_000450	−2.46165
8122265	TNFAIP3	tumor necrosis factor, alpha-induced protein 3	inflammation	6q23	NM_006290	−2.0769
8028652	ZFP36	zinc finger protein 36, C3H type,homolog (mouse)	inflammation	19q13.1	NM_003407	−2.36152
7955589	NR4A1	nuclear receptor subfamily 4, group A,member 1	orphan receptor	12q13	NM_002135	−2.36834
8055952	NR4A2	nuclear receptor subfamily 4, group A,member 2	orphan receptor	2q22-q23	NM_006186	−2.11225
8156848	NR4A3	nuclear receptor subfamily 4, group A,member 3	orphan receptor	9q22	NM_173198	−3.65489
7929816	SCD	stearoyl-CoA desaturase (delta-9-desaturase)	fatty acid metabolism	10q23-q24	NM_005063	−2.69181
7920873	SNORA42	small nucleolar RNA, H/ACA box 42	small nucleolar RNA expression	1q22	NR_002974	−2.13869
8069676	ADAMTS1	ADAM metallopeptidasew/thrombospondin type 1 motif, 1	extracellular matrix protein	21q21.2	NM_006988	−2.77417
7921821	ADAMTS4	ADAM metallopeptidasew/thrombospondin type 1 motif, 4	extracellular matrix protein	1q21-q23	NM_005099	−2.82585
7902687	CYR61	cysteine-rich, angiogenic inducer, 61	extracellular matrix protein	1p31-p22	NM_001554	−2.94737
8025601	ICAM1	intercellular adhesion molecule 1 (CD54)	extracellular matrix protein	19p13.3-p13.2	NM_000201	−2.25818
8065353	THBD	thrombomodulin	extracellular matrix protein	20p11.2	NM_000361	−2.80578
7982597	THBS1	thrombospondin 1	extracellular matrix protein	15q15	NM_003246	−3.01553
8152297	ANGPT1	angiopoietin 1	other	8q22.3-q23	NM_001146	2.01173
8176276	ATRX	alpha thalassemia/mental retardationsyndrome X-linked	other	Xq13.1-q21.1	NM_138270	2.62294
8086330	AXUD1	AXIN1 up-regulated 1	other	3p22	NM_033027	−2.06593
8119088	CDKN1A	cyclin-dependent kinase inhibitor 1A(p21, Cip1)	other	6p21.2	NM_078467	−2.02657
7971653	DLEU2	deleted in lymphocytic leukemia, 2	other	13q14.3	NR_002612	2.22215
8024485	GADD45B	growth arrest and DNA-damage-inducible, beta	other	19p13.3	NM_015675	−2.811
7954090	EMP1	epithelial membrane protein 1	other	12p12.3	NM_001423	−2.17339
7995806	MT1A	metallothionein 1A	antioxidant	16q13	AY028617	−2.5602
7995787	MT1M	metallothionein 1M	antioxidant	16q13	NM_176870	−2.49124
7903171	RWDD3	RWD domain containing 3	other	1p21.3	NM_015485	2.03845
8135069	SERPINE1		other	7q21.3-q22	NM_000602	−2.67627
8070665	SNF1LK	SNF1-like kinase	other	21q22.3	NM_173354	−2.38371
8100076	YIPF7	Yip1 domain family, member 7	other	4p13	NM_182592	2.07083
8036813	ZNF780B	zinc finger protein 780B	other	19q13.2	NM_001005851	2.1765

**Table 6:** Genes changed ≥4 fold (log 2 base ≥2) between 12 and 0 months with omentectomy.

Probe ID	Gene Symbol	Gene Description	Process	Cytoband	mRNA Accession	log2fold
7908650	IGFN1	eEF1A2 binding protein	protein turnover	1q32.1	NM_178275	−2.33443
8108370	EGR1	early growth response 1	transcription factor	5q31.1	NM_001964	−4.20485
7975779	FOS	v-fos FBJ murine osteosarcoma viral oncogenehomolog	transcription factor	14q24.3	NM_005252	−5.064
8029693	FOSB	FBJ murine osteosarcoma viral oncogene homolog B	transcription factor	19q13.32	NM_006732	−2.66602
8026047	JUNB	jun B proto-oncogene	transcription factor	19p13.2	NM_002229	−3.42952
8148317	MYC	v-myc myelocytomatosis viral oncogenehomolog (avian)	transcription factor	8q24.21	NM_002467	−3.13093
7955858	HOXC10	homeobox C10	transcription factor	12q13.3	NM_017409	2.15884
7902687	CYR61	cysteine-rich, angiogenic inducer, 61	insulin signaling	1p31-p22	NM_001554	−2.51451
8163002	KLF4	Kruppel-like factor 4 (gut)	insulin signaling	9q31	NM_004235	−2.04104
7960865	SLC2A3	solute carrier family 2 (facilitated glucosetransporter) member3	insulin signaling	12p13.3	NM_006931	−2.51532
7934979	ANKRD1	ankyrin repeat domain 1 (cardiac muscle)	inflammation	10q23.31	NM_014391	−3.4932
8006433	CCL2	chemokine (C-C motif) ligand 2	inflammation	17q11.2-q12	NM_002982	−3.21861
7934916	CH25H	cholesterol 25-hydroxylase	inflammation	10q23	NM_003956	−2.42635
8100994	CXCL2	chemokine (C-X-C motif) ligand 2	inflammation	4q21	NM_002089	−2.2466
8162276	NFIL3	nuclear factor, interleukin 3 regulated	inflammation	9q22	NM_005384	−2.47028
7922229	SELE	selectin E (endothelial adhesion molecule 1)	inflammation	1q22-q25	NM_000450	−2.42547
8018864	SOCS3	suppressor of cytokine signaling 3	inflammation	17q25.3	NM_003955	−3.36272
8122265	TNFAIP3	tumor necrosis factor, alpha-induced protein 3	inflammation	6q23	NM_006290	−2.15921
8028652	ZFP36	zinc finger protein 36, C3H type, homolog(mouse)	inflammation	19q13.1	NM_003407	−2.21499
8131803	IL6	interleukin 6 (interferon, beta 2)	inflammation	7p21	NM_000600	−5.07812
8062461	LBP	lipopolysaccharide binding protein	inflammation	20q11.23-q12	NM_004139	−2.13523
8025828	LDLR	low density lipoprotein receptor (familial hypercholesterolemia)	receptors	19p13.3	NM_000527	−2.22451
7955589	NR4A1	nuclear receptor subfamily 4, group A, member 1	receptors	12q13	NM_002135	−2.04616
8156848	NR4A3	nuclear receptor subfamily 4, group A, member 3	receptors	9q22	NM_173198	−3.6856
8069676	ADAMTS1	ADAM metallopeptidase with thrombospondintype 1 motif, 1	extracellular matrix	21q21.2	NM_006988	−3.41834
7921821	ADAMTS4	ADAM metallopeptidase with thrombospondintype 1 motif, 4	extracellular matrix	1q21-q23	NM_005099	−2.4919
8065353	THBD	thrombomodulin	extracellular matrix	20p11.2	NM_000361	−2.61527
7982597	THBS1	thrombospondin 1	extracellular matrix	15q15	NM_003246	−3.12862
8086330	AXUD1	AXIN1 up-regulated 1	other	3p22	NM_033027	−2.08176
8084206	B3GNT5	UDP-GlcNAc:betaGal β-1,3-N-acetylglucosaminyltransferase 5	other	3q28	NM_032047	−2.25232
7954090	EMP1	epithelial membrane protein 1	other	12p12.3	NM_001423	−2.14602
8024485	GADD45B	growth arrest and DNA-damage-inducible, beta	other	19p13.3	NM_015675	−2.47919
8086538	LOC644714	hypothetical protein LOC644714	other	3p21.31	BC047037	−2.13574
7995806	MT1A	metallothionein 1A	other	16q13	AY028617	−2.47681
7995787	MT1M	metallothionein 1M	other	16q13	NM_176870	−2.24145
8135069	SERPINE1	serpin peptidase inhibitor, E1 (plasminogenactivator inhibitor type 1)	other	7q21.3-q22	NM_000602	−2.56719

### Effects of omentectomy evaluated by global pathway analysis

To identify pathways in skeletal muscle that were regulated by RYGB with and without omentectomy we performed two complimentary analyses. For these analyses those genes detected as being changed greater than 2-fold (2,697 genes; **Table S1**) were analyzed. First, the gene list was analyzed by the FuncAssociate Gene Ontology program (http://llama.mshri.on.ca/funcassociate/) to statistically identify overrepresented gene categories [39]. Cadmium ion binding (GO:0046870), positive regulation of leukocyte binding (GO:0002687) and cellular macromolecule catabolic processes (GO:0044265) were top ranked. Second, we annotated our expression data with the wiring diagrams of molecular interactions, reactions, and relations maintained at the Kyoto Encyclopedia of Genes and Genomes (KEGG) PATHWAY Database. As reported in **Table S4**, coverage of 201 pathways by our experimental datasets revealed that *metabolic pathways, cytokine-cytokine receptor interaction*, *MAPK signaling pathways, chemokine signaling pathways, and spliceosome* were top ranked.

### Validation of microarray expression profiles with TLDA

As the significance of gene expression changes determined with microarray could not be determined empirically due to the low number of array replicates, and because a single outlier sample within an RNA pool could in theory skew results, we elected to validate the expression changes of select genes in each of the individual RNA samples comprising the RNA pools using Taqman Low Density Arrays (TLDA). In addition to the RNA samples used to prepare the RNA pools, we also included RNA samples prepared from additional participants lacking a single study (either 6 or 12 months) time point RNA sample. Using a spot checking strategy, expression of 31 genes was used to validate differential expression data discovered during the microarray study (**Table S2**). This approach is admittedly restrictive; however, we focused on genes involved in pathways such as inflammation, transcriptional regulation, protein turnover and insulin signaling [40], [41]. Gene selection was guided based on literature reviews of the effects of weight loss on skeletal muscle, our recent report that omentectomy concurrent with RYGB did not improve muscle insulin sensitivity [14], and recent observations that altered intramuscular lipid metabolism, circulating cytokines and macrophage infiltration may act synergistically to coordinate insulin sensitivity [40], [41].

**Figure 4:**
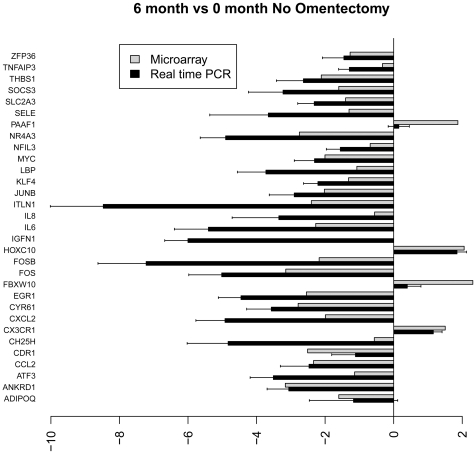
Bar graph illustrating the fold change of individual genes at 6 vs. 0 months without omentectomy as determined by microarray (grey) and TLDA profiling (black).

**Figures 4** **–** [Fig pone-0028577-g005] [Fig pone-0028577-g006] **7** show the relative average differential expression values determined by microarray (light grey) and TLDA (dark grey) for 31 genes, normalized to an 18S RNA control, when analyzed in triplicate at 0, 6 and 12 months. Of the 5,184 Taqman analyses performed to validate select microarray-derived expression values, only 3 reactions did not amplify. In general, there was good directional (increased or decreased) concordance between expression values derived from analysis of pooled RNA samples and the average of each RNA sample analyzed independently. In most instances, fold expression change values determined by exon array in pooled RNA samples were greater than the averages of individual expression values obtained by TLDA (e.g. FOSB, EGR and IL6 in **Figures 4** **–** [Fig pone-0028577-g005] [Fig pone-0028577-g006] **7**). We examined the relationship between gene expression values revealed by exon array and TLDA profiling. In all group comparisons, there were strong positive relationships: 1) 6 vs. 0 months without omentectomy rho = 0.559 (**Figure 8A** **)**; 12 vs. 0 months without omentectomy rho = 0.720 (**Figure 8B**); 6 vs. 0 months with omentectomy rho = 0.646 (**Figure 8C**); and 12 vs. 0 months with omentectomy rho = 0.640 (**Figure 8D**).

**Figure 5 pone-0028577-g005:**
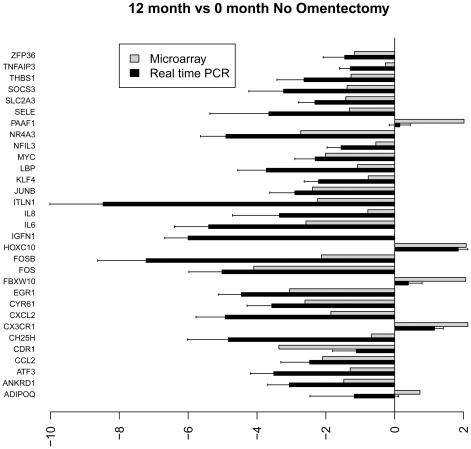
Bar graph illustrating the fold change of individual genes at 12 vs. 0 months without omentectomy as determined by microarray (grey) and TLDA profiling (black).

**Figure 6 pone-0028577-g006:**
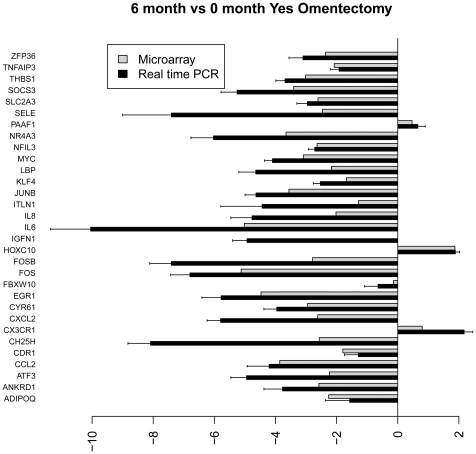
Bar graph illustrating the fold change of individual genes at 6 vs. 0 months with omentectomy as determined by microarray (grey) and TLDA profiling (black).

**Figure 7:**
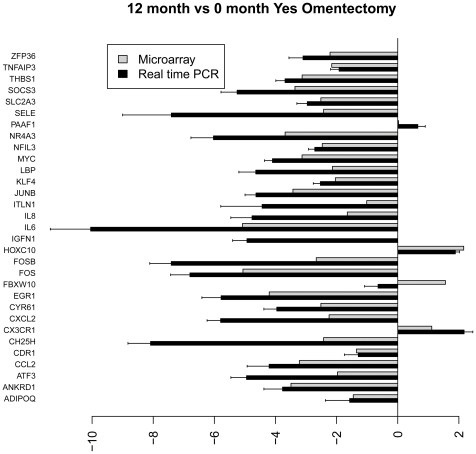
Bar graph illustrating the fold change of individual genes at 12 vs. 0 months with omentectomy as determined by microarray (grey) and TLDA profiling (black).

**Figure 8:**
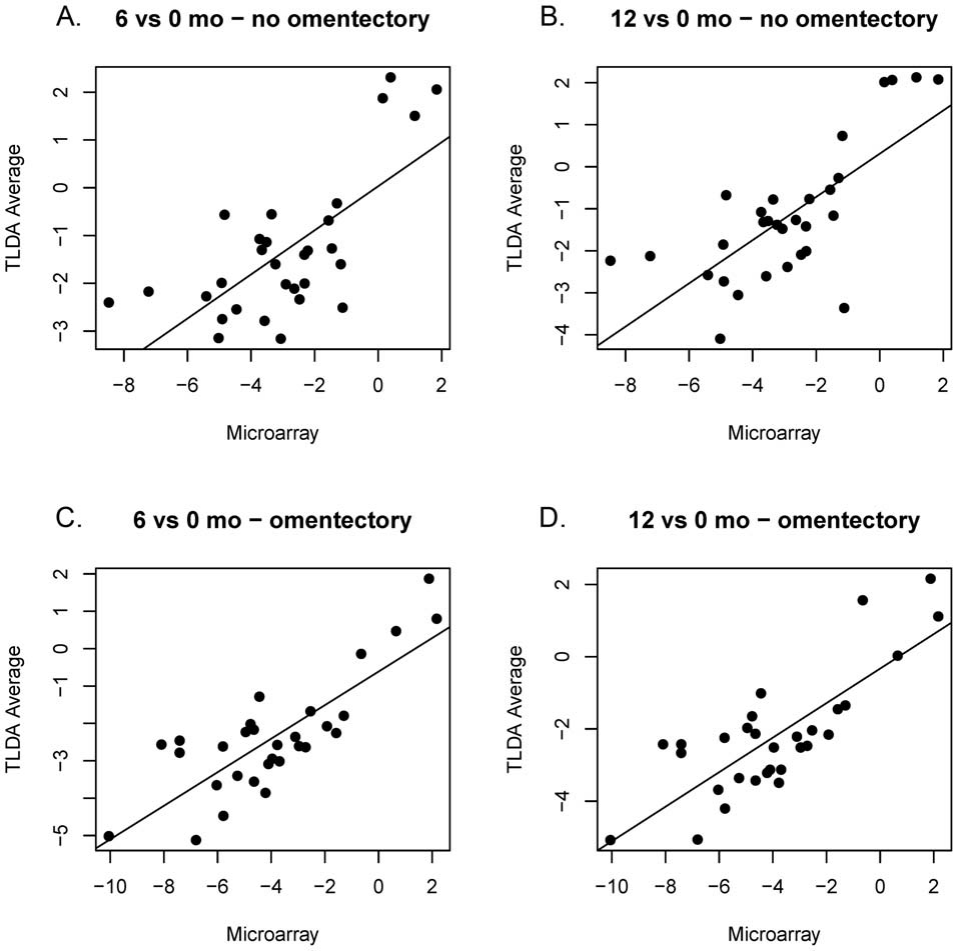
Correlation in fold change (log_2_) of gene expression as determined by Affymetrix Exon microarray and TLDA profiling. A) 6 vs. 0 months, no omentectomy, Pearson correlation coefficient rho = 0.559, *p*<0.0001; B) 12 vs. 0 months, no omentectomy, Pearson correlation coefficient rho = 0.646, *p* = 0.0002; C) 6 vs. 0 months, yes omentectomy, Pearson correlation coefficient rho = 0.649, *p* = 0.0002; D) 12 vs. 0 months, yes omentectomy, Pearson correlation coefficient rho = 0.640, *p* = 0.0002.

## Discussion

This is the first high-throughput study to explore the temporal changes in human skeletal muscle gene expression after weight loss through RYGB surgery with tandem laparoscopic omentectomy. In addition, randomization of subjects to removal of the omentum at the time of RYGB allowed us to explore the interplay between visceral adipose and skeletal muscle. While the effects of bariatric surgery on gene expression in muscle have been reported for a few targets [42], [43], [44], [45], the influence of the omentum (visceral adipose) on global skeletal muscle phenotypes (such as insulin resistance) has been understudied. Visceral adipose is the source of 14–20% of the total FFAs delivered to skeletal muscle in obese individuals [46], [47] and numerous studies have linked circulating FFA levels and muscle triglyceride accumulation with peripheral insulin sensitivity [48], [49], [50], [51]. Visceral adipose tissue is also the site of production of several cytokines thought to initiate and maintain pro-inflammatory states both locally and in other tissues [52]. Infiltration and local activation of macrophages in skeletal muscle also has been described [40], [41]. Our group recently reported, in the same subjects examined in the present study, that laparoscopic removal of the greater omentum at the time of RYGB did not significantly enhance weight loss-induced improvements in peripheral insulin sensitivity beyond those observed with RYGB alone [14]. These findings were unanticipated in light of the numerous studies associating increased visceral adiposity with insulin resistance and T2D [53], [54], [55]. Despite these findings, it remained possible that omentectomy produced local changes at the level of the muscle that may not be totally reflected with associated improvements in peripheral glucose utilization. The Affymetrix Exon arrays and TLDA approaches utilized in this study provided a robust and reproducible technique for quantifying gene expression in thousands of independent genes concurrently in RNA samples isolated from multiple tissue samples. This approach represents a significant advance in multivariate gene analysis that was less time- and labor-intensive than individually analyzing single genes by quantitative RT-PCR.

Our major finding was a broad-based downregulation of inflammatory-related pathways in skeletal muscle after RYGB surgery. This response was amplified in the cohort receiving omentectomy, as evidenced by a greater number of genes with at least a 4-fold decrease at 6 or 12 months (at least 4 genes in the RYGB non-omentectomy cohort compared to 11 in the RYGB with omentectomy cohort). Most of the observed inflammatory genes that were only decreased in the RYGB with omentectomy cohort are reportedly associated with macrophage activation (CH25H, CXCL2, SOCS3, LBP, NFIL3, ZFP36) [56], [57], [58], [59], [60], consistent with the systemic decrease in MCP-1, or associated with inflammatory events surrounding sarcopenia (ANKRD1, LBP) [61], [62]. Certain genes such as IL6 and CCL2 were decreased in both groups at both time points relative to pre-surgery levels, but the reductions were greater in the omentectomy group. Changes in IL6 expression levels at 6 and 12 months in the RYGB with omentectomy cohort were the greatest of any gene detected. IL6 is a pleiotropic cytokine described to impact insulin action [63], [64]. IL-6 infusion reportedly increased fatty acid oxidation in human skeletal muscle but not adipose tissue, suggesting a role for this cytokine in stimulating skeletal muscle lipolysis specifically [65]. In the current study, despite glucose utilization (M) being significantly improved at both time-points, omentectomy did not have a differential effect. Interestingly, systemic levels of IL-6 did not change after RYGB. In contrast, other cytokines such as CX3CR1 [66] were equally diminished at 6 and 12 months in both groups after RYGB. Circulating monocytes with high levels of CX3CR1 (frataxylin), are present in systemic disease, such as sepsis or HIV infection, while the macrophage subset that invades tissues during acute localized inflammation expresses lower levels of CX3CR1. These data provide evidence that RYGB reduces the local inflammatory response in muscle which is enhanced by omentectomy, and the physiological consequences warrant further study.

The expression of several transcription factors involved in proliferation control, (FOS, FOSB, JUNB, MYC and EGR1) was reduced at 6 and 12 months after RYGB in both RYGB cohorts with and without omentectomy. These five genes were among those that showed the greatest drop in expression post-RYGB, and their decrease was greater in the omentectomy cohort at both time-points. FOS, FOSB and JUNB are intermediate early genes comprising the AP-1 basic leucine zipper transcription factor family that act on specific biological responses to regulate gene expression. Each has been shown to be increased immediately after exercise [35], but this study is the first to associate them with changes in skeletal muscle after RYGB. Adipose-specific overexpression of ΔFOSB, a naturally occurring alternatively spliced variant of FosB that is antagonistic to normal FosB-mediated gene transcription, was recently shown to increase energy expenditure in muscle and increase insulin sensitivity [67]. Expression of EGR1, a GC box-binding transcription factor, has been shown to regulate TGF-β expression which, in turn increases myostatin expression [68]. The expression of EGR-1 was decreased over 4-fold in the omentectomy cohorts compared to the 2- and 3-fold reductions observed in the non-omentectomy cohort at 6 and 12 months, respectively. EGR-1 binding sites are prevalent in the promoter binding sites for genes such as peroxisomal gamma coactivator-1α (PGC-1α), that are important regulators of mitochondrial biogenesis [69]. The nuclear phosphoprotein MYC was decreased 3-fold at both 6 and 12 month time points in the RYGB with omentectomy cohort but only 2-fold at the same time points in the group without omentectomy. In contrast, HOXC10, a homeoprotein associated with muscle satellite cell activation was upregulated over 2-fold at 12 months in both cohorts, and at 6 months in the RYGB with omentectomy group. The protein product of this gene, together with Pax7, Asb5 and IgSF4, was shown to be markers for skeletal muscle satellite cells [70]. In the present study, increased HOXC10 expression at all time points in both cohorts implies that muscle satellite cell activation and deactivation of transcriptional responses are an important phenomenon in muscle remodeling, particularly after weight loss.

Genes facilitating extracellular matrix (ECM) remodeling such as CYR61, THBS1 and THBS4 were downregulated, consistent with an improvement in inflammation. High levels of muscle expression of ECM, inflammatory, and immediate early genes in obesity could indicate a continual, unsuccessful attempt to repair muscle damage caused by fat overload. A role for the ECM in insulin resistance has been described but is largely unappreciated. Infusion of FFA in subjects with normal glucose tolerance has been shown to increase the expression of ECM genes in muscle coincident with a decrease in insulin sensitivity [71]; furthermore, animal studies indicate that ECM expansion contributes to insulin resistance [72]. These studies in concert with our current results indicate that further studies connecting obesity, the ECM, and insulin resistance are warranted.

Three genes annotated as regulating protein turnover were significantly altered upon RYGB in both surgical groups. The gene encoding eEF1A2 binding protein (IGFN1 or Dkfzp434B1231) plays a key role in protein translation, carrying each aminoacyl-transfer RNA complex to the A site of the ribosome after codon-anticodon recognition [73]. In cultured myotubes, IGFN1 expression inhibited apoptosis and promoted cell proliferation in hyper-catabolic trauma patients [74] and was required for the muscle-specific expression of utrophin A, a sarcomellar protein causal in the fatal neuromuscular disease Duchenne Muscular Dystrophy [75]. In our studies IGNF1 expression was reduced greater than 4-fold, a pattern consistent with muscle catabolism known to occur in this population. Other genes involved in protein turnover such as FBXW10, a component of the E3 ubiquitin ligase system that determines substrate specificity during proteasomal degradation, demonstrated an increased expression pattern primarily at the 12-month time point. It seems likely that as a result of RYGB the regulation of muscle protein maintenance is upset. Future studies in this population examining muscle protein synthesis and muscle protein breakdown using state-of-the-art isotopic tracer methodologies may delineate mechanisms contributory to this observation.

At 6 and 12 months after RYGB in the non-omentectomy group, several small nucleolar RNAs from the C/D box group (SNORDs) were among the few genes that were upregulated in skeletal muscle. SNORDs are a class of small RNA non-protein coding micro RNAs that molecules that guide chemical modification, particularly 2′-O-methylation [76] and alternative splicing [77] of other RNAs. Interestingly, the absence of small nucleolar RNA C/D box 115 gene expression units have been implicated as causal in Prader-Willi Syndrome [78], the most common form of monogenetic obesity. Previous studies, however, indicate that expression of HBII-52 is restricted to the brain [78]; thus, the relevance of the observed expression of SNORD 115 variants in muscle and the increased expression after RYGB is highly unclear.

We have identified a set of genes, which were significantly and differentially altered after RYGB with and without omentectomy; however, there are some important limitations to this study. First, while the utmost effort was placed on ensuring the quality of the muscle biopsies used for RNA extraction both before and after surgery, it remains possible that some intercalated adipose, adventitia and/or microvasculature may have been present in some tissues. This phenomenon was more likely in biopsies obtained prior to RYGB surgery, compared to those at either time point after surgery when significant weight loss occurred, and may lead to significant gene expression changes that are not exclusive to skeletal muscle. For example, the observed changes in adiponectin (ADIPOQ) and omentin-1 (ITLN1) might be reflective of intercalated adipose tissue as this is their primary site of production, although muscle specific expression has been reported [79]. Thirdly, we elected to use Robust Multi-array Averaging, a common normalization method, but did not perform the default quantile across sample normalization method of RMA to avoid over-correction for the small sample size. The data were also analyzed without adjustments for multiplicity, which could alter differential expression estimates [80]. Nevertheless, our validation efforts focused primarily on those genes affected 4 fold or greater (log_2_ – 2 fold) and, as such, any misinterpretations based on this should be minimal. Finally, we are unable to account for the extrinsic factors, other than those reported in **Tables 1** and **2** that can affect end-organ metabolic response. As RYGB surgery is a procedure known for its systemic and interconnected effects on hormonal, neuronal, and biochemical impulses, the discrimination of a muscle-specific response due solely to the removal of omentum remains a challenge.

With regard to our overall study design, there are some limitations that should be mentioned. The population studied was reflective of Nashville, TN and the surrounding counties with an ethnic distribution (Caucasians to African Americans) of 4∶1. While both the omentectomy and non-omentectomy group contained participants with and without T2D, the proportion of subjects with T2D was unequal between groups. There were also no Hispanics in the studied cohorts, based on our geographic locale, and the subjects studied were also primarily female reflective of the patient population seeking this surgical approach; therefore, our findings may be gender and ethnically limited.

## Supporting Information

Checklist S1 **Consort 2010 checklist for randomized trial.**
(DOC)

Protocol S1 **Trial protocol.**
(DOC)

Table S1 **Expression values for genes changed greater than 2 fold between 6 vs. 0 months, no omentectomy; 12 vs. 0 months, no omentectomy; 6 vs. 0 months with omentectomy; and 12 vs. 0 months, with omentectomy.**
(PDF)

Table S2 **Expression values for genes changed greater than 4 fold between 6 vs. 0 months, no omentectomy; 12 vs. 0 months, no omentectomy; 6 vs. 0 months with omentectomy; and 12 vs. 0 months, with omentectomy.**
(PDF)

Table S3 **TLDA probes utilized in this study.**
(PDF)

Table S4 **Average gene expression values for those molecular interaction members within the indicated molecular pathway.** Pathways are ranked highest to lowest in terms of the number of genes detected per pathway.
(PDF)
