# Supplementary material for: Reduction in Inflammatory Gene Expression in Skeletal Muscle from Roux-en-Y Gastric Bypass Patients Randomized to Omentectomy
Source: PLoS One. 2011 Dec 16;6(12):e28577. doi: 10.1371/journal.pone.0028577 (PMC3241684; doi:10.1371/journal.pone.0028577)
Supplement: Protocol S1 — Trial protocol. (DOC) [file pone.0028577.s002.doc]

The Role of the Omentum in the Treatment of Morbid Obesity

| Naji Abumrad, MD | Principal Investigator: Chair, Department of Surgery. D-4313 MCN, Vanderbilt |
| --- | --- |
| **Co-Investigators** | |
| Alp Ikizler, MD | Division of Nephrology, Dept. of Medicine, Vanderbilt |
| Ronnie Clements, MD | Dept. of Surgery, Vanderbilt |
|  |  |
| Willie Melvin, MD | Dept. of Surgery, Vanderbilt |
| Brandon D. Williams, MD | Department of Surgery, Vanderbilt |
| Pam Marks, MS | Dept. of Surgery, Vanderbilt |
| Robyn Tamboli, PhD | Dept. of Surgery, Vanderbilt |
| Tahar Hajri, PhD | Dept. of Surgery, Vanderbilt |
| Marcy Buckley, RN | Dept. of Surgery, Vanderbilt |
|  |  |
| Igal Breitman, MD | Dept. of Surgery, Vanderbilt |
| Julia Dunn, MD | Dept. of Medicine, Vanderbilt |
| Robb Flynn, PhD | Dept. of Surgery, Vanderbilt |
| Phyllis Egbert, RN | Division of Nephrology, Dept. of Medicine, Vanderbilt |
| Yukiko Ueda, PhD | Dept. of Surgery, Vanderbilt. |
| Joan Kaiser, RN | Dept. of Surgery, Vanderbilt |
| Kareem Jabbour | Dept. of Surgery, Vanderbilt |

**Table of Contents:**

**Study Schema**

1. **Background**
2. **Rationale and Specific Aims**
3. **Animal Studies and Previous Human Studies**
4. **Inclusion/Exclusion Criteria**
5. **Enrollment/Randomization**
6. **Study Procedures**
7. **Risks of Investigational Agents/Devices (side effects)**
8. **Reporting of Adverse Events or Unanticipated Problems involving Risk to Participants or Others**
9. **Study Withdrawal/Discontinuation**
10. **Statistical Considerations**
11. **Privacy/Confidentiality Issues**
12. **Follow-up and Record Retention**

**Appendices**

**Appendix A Study Procedure Calendar**

1. **Background**

Obesity is defined as having a BMI greater than 30. Data from the 2001 BRFSS *(Mokdad 2003)*, a cross-sectional survey conducted by the CDC and state health departments, estimated incidence in obesity at 20.9% and that of diabetes at 7.9% among US adults. Data from NHANES III (Third National Health and Nutrition Examination Survey) *(Harris 1998)* conducted in two phases from 1988 to 1994, showed a graded increase in the obesity prevalence ratio with increasing severity of all associated pathologies especially T2DM *(Iscove 2002)*. Epidemiological data have established a positive correlation between increasing BMI and various metabolic and cardiovascular morbidities *(Kissebah 1994, Okossun 2000)*.

Incidence of obesity varies among different subpopulations. African American adults *(Cowries 1993, Burke 1996)* and “especially women” are at increased risk for the development of obesity *(Kumanyika 1987)*, T2DM *(Harris 1990)* or gestational diabetes *(Dooley 1991)*. Among men, Mexican-Americans have the highest prevalence of obesity (*Ogden 2005).* Increased risk for mortality from cardiovascular diseases *(Henderson 2000, Clark 2001)* has also been reported in some, but not all, studies.

Not all types of obesity are associated with increased risk of metabolic and cardiovascular complications. For example, individuals with increased peripheral fat distribution, such as in the lower abdomen and gluteofemoral regions are less prone to develop severe metabolic and cardiovascular complications *(Arner 1998)*. Conversely, the amount of visceral fat strongly correlates with insulin resistance and can account for most of the variability in insulin sensitivity in heterogeneous populations *(reviewed in Okosun 2000)*. Stolic and coworkers investigated basal and insulin stimulated 2-deoxyglucose (2-DG) uptake in omental and subcutaneous adipose tissue explants from obese patients. They found that insulin responsive 2-DG transport was significantly lower in omental fat tissue of subjects with central obesity as compared to that of subjects with peripheral obesity *(Stolic 2002)*. Another important reason behind the correlation between visceral fat and insulin resistance is that this tissue as compared to subcutaneous fat is a more important producer of several cytokines *(Fried 1998, Fruhbeck 2001)*. In addition, the omentum is a major down loader of FFA into the portal circulation leading to abnormally elevated hepatic glucose production and hyperinsulinemia *(Mittelman 2002)*.

***Role of adipose tissue-derived peptides:***

Some of the effects of visceral fat tissue on insulin resistance may be mediated by abnormal release of various peptides that function in regulating energy homeostasis *(Yudkin 1999, Berg 2001, Steppan 2001)*. These peptides include leptin, adiponectin (30-kDa adipocyte complement-related protein), resistin and PYY. Leptin is released by adipocytes in response to nutrient supply and acts in the brain to down regulate food intake and peripherally to increase energy consumption, especially by muscle tissue. Peripheral resistance to leptin associated with blunted relative diurnal excursion and dampened pulsatility have been reported in obese humans *(Saad 1998)* and may contribute to increasing fat storage in muscle and liver, conditions that positively correlate with insulin resistance *(Yudkin 1999)*.

Adiponectin is released by adipocytes, exerts effects similar to those of leptin as it increases fatty acid oxidation by muscle, and enhances hepatic insulin sensitivity. Obese JNK1 (Jun-N-terminal Kinase-1)-deficient mice exhibit higher levels of ACRP30/adiponectin concentrations *(Hirosumi 2002)* suggesting that adiponectin may link the JNK pathway to regulation of adiposity and insulin resistance. In humans, there is evidence to indicate that diurnal variations in circulating adiponectin levels are reduced in obese (both diabetic and non-diabetic) subjects and that sensitivity to the action of adiponectin may also be altered *(Mora 2002)*. More recently, single nucleotide polymorphisms (SNPs) in the proximal promoter region of the adiponectin gene were identified in Caucasians and proposed to contribute to the development of T2DMT2DM *(Gu 2004)*.

Resistin has also been implicated to be an important link between increased fat mass and insulin resistance. It is derived exclusively from adipocytes in rodents *(Steppan 2001)*, but produced mainly by macrophages in humans *(Savage 2001)*. In both species, however, it exerts hormonal actions at distant sites *(Steppan 2001)*. Neutralization of resistin activity by the injection of antibodies decreased blood glucose levels and improved insulin sensitivity in obese insulin-resistant mice. Also, transgenic mice with impaired resistin function exhibited improved insulin sensitivity *(Kim 2004)*. The role of resistin in humans remains to be determined. Although resistin plasma levels are elevated in type 2 diabetics *(Youn 2004)* only a weak relationship between resistin and insulin sensitivity could be demonstrated in non-obese subjects *(Heilbron 2004)*. Possibly exploring tissue-specific alterations of resistin levels, especially in visceral adipose tissue could prove more relevant to obesity-linked insulin resistance. For example, in visceral fat, obesity (ob/ob) increases and fasting decreases resistin expression, while in the pituitary both conditions decrease resistin mRNA *(Morash 2004)*.

Ghrelin is a recently discovered regulatory peptide that is released by the gut and by other tissues such as fat. Unlike leptin and adiponectin, ghrelin acts peripherally to decrease energy consumption and increase energy storage so its effects are opposite to those of leptin and adiponectin. Ghrelin levels peak before a meal and decrease following food ingestion *(Reviewed by Ukola 2002)*. Ghrelin levels are reduced with obesity and a recent study documented anti-inflammatory actions of ghrelin and suggested that low ghrelin levels could play a role in the susceptibility of obese individuals to atherosclerosis *(Li 2004)*.

Currently, there is little available data concerning the ultradian rhythm of pulsatility of adipokines and whether this influences insulin sensitivity. Weight loss restores abnormalities in leptin *(Saad 1998)* and insulin *(Gumbiner 1996)* pulsatility, but little is known about the pulsatility of adiponectin, resistin, and ghrelin. For example, levels of adiponectin increase following weight loss *(Calyani 2004)*, obtained by gastric partition *(Faraj 2003)* or RYGB *(Yang 2003)* in morbidly obese subjects, but there is no information regarding the effect on pulsatility. Maintenance of the proper discontinuous or pulsatile secretion may be very important, as it has often been linked to receptor sensitivity with continuous release producing desensitization. Changes in pulsatility may ultimately be more relevant to insulin responsiveness since they could maintain normal effectiveness of the various adipokines in interacting with their receptors to regulate energy consumption. These issues will be considered in our proposed studies during progressive periods of weight loss following RYGB. We hypothesize that the rate of restoration of pulsatility in these hormones are racially determined and the removal of the omentum in combination with RYGB would alter the clearance rates of several of these adipokines and influence their ultradian rhythms.

***Role of inflammatory mediators in the pathogenesis of insulin resistance****:*

There is increasing consensus that obesity is an inflammatory condition *(Das 2001)* and that chronic inflammation acts as a trigger of insulin resistance *(Grimble 2002)*. Many of the metabolic and hemodynamic abnormalities associated with obesity and T2DM may be related to altered production of pro-inflammatory factors and adipokines by adipose tissue and particularly in the intra-abdominal (visceral) location. Genes involved in inflammatory pathways are up-regulated in adipose tissue in obesity *(Soukas 2000)*. Tissue from obese humans and animals express increased amounts of TNF-α *(Hotamisligil 1993, 1995, 1998, Peraldi 1998)* and IL-6, leading to increased hepatic production of CRP. In turn this leads to increased production of adhesion molecules from endothelial cells, such as soluble ICAM-1 (intercellular adhesion molecule-1), VCAM-1 (vascular adhesion molecule-1) and monocyte chemotactic protein-1 (MCP-1) *(reviewed in Rajala 2003)*. A recently published study of a prospective, nested case-control study involving 32,826 women of the Nurses’ Health Study cohort strongly supports the role of inflammation in the pathogenesis of T2DM. Elevated levels of CRP were good predictors of T2DM and could mediate the association of TNF-αR2 (TNF-α receptor 2 as a measure of plasma TNF-α) and IL-6 with T2DM *(Hu 2004)*.

The inflammatory response associated with obesity appears to be “triggered and to reside predominantly” in adipose tissue and primarily in its visceral localization *(Hirosumi 2002)*. About one-third of circulating IL-6 may originate from adipose tissue *(Ali 1997)*, with omental fat producing three-fold more than subcutaneous fat *(Fried 1998)*. Similarly, stromal vascular cells present in adipose tissue also express receptors for several of the cytokines, such as TNF-α, IL-6 and IL-1β. Recent observations by Weisberg et al. *(2003)* and by Xu et al. *(2003)* indicate that a significant proportion of the visceral fat in obese humans and mice consists of macrophages with an estimated content equivalent to nearly 50% of the total omental mass in morbidly obese subjects. Interestingly, macrophages were found to account for expression of almost all of the adipose tissue TNF-α and of significant amounts of iNOS and IL-6 *(Weisberg 2003)*. It is perhaps revealing that up-regulation of the macrophage-specific inflammatory genes occurs progressively in diet-induced obesity and precedes the increase in plasma insulin levels *(Xu 2003)*. Furthermore, the adipocyte fatty acid binding protein, aP2, which is expressed by both macrophages and adipocytes, promotes insulin resistance in both genetic and diet-induced obesity *(Hotamisligil 1996)* and has been shown by our collaborator, Dr. MacRae Linton, to promote atherosclerosis, thus linking these features of the metabolic syndrome *(Makowski 2001)*. Interestingly, the expression of inflammatory cytokines such as TNF-α is dramatically reduced in adipocytes and macrophages null for aP2 expression *(Makowski 2001)*.

Cytokines can also contribute to insulin resistance by modulating adipocyte metabolism and enhancing adipose tissue lipolysis and particularly that of visceral fat. Intra-abdominal adipocytes are more lipolytically active than subcutaneous cells and this is attributed to increased complement of adrenergic receptors. Additionally excessive production of TNF-α *(Hotamisligil 1994, Zhang 2002)* and of IL-6 by visceral fat would confer resistance to the anti-lipolytic action of insulin *(Richelsin 1994, Yudkin 1999).*. The continuous omental delivery of FFA to the liver *(Bjorntorp 1990)* leads to hypertriglyceridemia by stimulating visceral fat hepatic triglyceride secretion *(Nonogaki 1995)* and decreases insulin sensitivity *(Li 2002)*. The ability of insulin to suppress hepatic glucose production is impaired, further contributing to the etiology of obesity-induced insulin resistance *(Boden 1997)*.

The mechanism by which FFAs induce insulin resistance has been under intense investigation. Elevated FFAs activate PKC-θ *(Griffin 1999)*, which in turn activates the serine kinase IKK that interferes with insulin signaling *(Hotamisligil 1993, Kim 2001, Rui 2001, Sun 2000, Schmitz Peiffer 2002)*. Both TNF-α and FFAs are potent activators of another serine kinase, JNK *(Kyriakis 1996, Iman 2000, Chang 2001)*, which would result in increased serine phosphorylation of insulin receptor substrate-1 (IRS-1), causing inhibition of insulin signaling *(Griffin 1999, Hotamisligil 1996)*. It has been suggested that a reasonable therapeutic strategy for human obesity, insulin resistance and T2DM could be based on selective interference with the serine kinases. In line with this, studies in rodents showed that very high doses of aspirin and/or sodium salicylate, known to be weak inhibitors of IKK, could alleviate the insulin resistance observed with obesity *(DeFelice 1968, Baron 1982, Yamauchi 2001)*. Similarly, treatment of type 2 diabetic subjects with high dose aspirin (~6.8 g/day) reduced fasting plasma glucose, cholesterol and triglycerides. Insulin clearance and basal hepatic glucose production were reduced and insulin-stimulated peripheral glucose uptake was improved *(Ripudaman 2002)*.

Other direct evidence for the association between human obesity and inflammatory responses was obtained by one of our collaborators, Dr. Jason Morrow, and his colleagues. In 1990 they discovered a group of molecules termed F_2_-isoprostanes (F_2_-IsoPs), which are derived from free radical-catalyzed peroxidation of arachidonic acid *(Daniel 1994)*. These investigators documented a very strong correlation between increased F2-isoprostanes and BMI (preliminary data) *(Block 2002)*, findings that were reproduced by Keany et al. *(2002)* in 2,800 subjects enrolled in the Framingham study. Additional indirect support is obtained from the very strong association of obesity with platelet-activation with more than a three-fold increase in the excretion of platelet-derived thromboxanes in the urine of obese women *(Davi 2002)*.

***Bariatric surgery, weight loss and insulin sensitivity:***

Among the various modalities used to treat obesity *(reviewed in Greenway 2002)*, RYGB represents the most effective for sustained weight loss *(NIH 1998).*  Long term follow up studies by Pories et al. showed that patients undergoing RYGB lost ~75% of the excess body weight within 24 months, with only about a 10% regain after 14 years *(Hickey 1998)*. The weight loss achieved with RYGB exceeds that with any other surgical approach, accounting for its higher use in the treatment of morbid obesity. The mechanism by which weight loss occurs is not completely clear, but could be attributed to caloric restriction in association with malabsorption.

Although research on vertical sleeve gastrectomy (VSG) is not as extensive as that for RYGB, it does appear to produce a similar weight loss. Rubin et al reported 53% excess weight loss at 1 year post-operatively, whereas Moon Han et al found an 83% excess weight loss in the same time span. The literature is also scant in measurements of early hormonal changes observed after VSG. This procedure does appear to approach the same degree of diabetes resolution as RYGB.

RYGB has been shown to correct some of the abnormalities associated with obesity including symptoms of T2DM *(Herbst 1984, Greenway 2002)*. Studies from the Pories laboratory showed that RYGB leads to sustained improvements of plasma glucose, insulin and glycosylated hemoglobin levels in about 80% of patients with diabetes and in about 90% of patients without diabetes who were noted to be glucose intolerant *(Houmard 2002)*. The mechanism and the timing behind these improvements remain unknown. Most of the work thus far attributes the improvements in glucose homeostasis to significant loss of body fat. However, there is recent evidence to argue against this being the only mechanism. Our unpublished observations in three obese humans indicate that surgical removal of substantial amounts of subcutaneous adipose tissue mass (removal of 11-18 lb by liposuction and accounting for 7-12% of total body weight) did not result in measurable improvements in insulin sensitivity in obese (BMI: 27-34) subjects. On the other hand, studies in obese adults have shown that a weight loss of 5-10% of total body weight, achieved by caloric restriction, is sufficient to cause improvements in plasma insulin and glucose and to reverse abnormal glucose tolerance. In addition, with bariatric surgery, the post-surgical reversal of glucose tolerance begins long before any significant weight loss has occurred. In general, the mechanisms underlying the metabolic improvements following bariatric surgery remain almost completely unexplored. We have preliminary data to show that the improvement in insulin sensitivity (n=45) occurss as early as 10 days postoperatively - when the loss in body weight is less than 2%.

Recent work by Cummings et al. *(2002)* showed that gastric bypass procedures result in marked reductions in plasma levels of the orexigenic hormone, ghrelin. However, these observations were made nearly two years following the bariatric procedures, and it is unclear whether they apply to the early metabolic changes observed in the immediate post-operative period, which will be one of the goals of this proposal. Laimer et al. *(2002)* showed marked decreases in CRP following gastric banding consistent with our hypothesis that the procedures are being associated with decreases in the inflammatory pathways. However, no changes were noted in TNF-α or IL-6.

Alternatively, some of the improvements following bariatric surgery could be related to a redistribution of energy stores with acute reductions in muscle triglyceride content (mTG). Significant correlation exists between insulin resistance of obesity and T2DM and mTG content *(Pan 1997, Manco 2000)*, and specifically with intramyocellular (inside fiber) lipid *(Andewald 2002, Greco 2002, Krssak 1999, Malenfant 2001, Sinha 2002, Virkamaki 2001)*. Increased mTG is associated with decreased insulin-stimulated glycogen synthase activity *(Phillips 1996)* and lower glycogen stores *(Levin 2001)*. High mTG probably reflects excess muscle fatty acid uptake over oxidative capacity, which is usually associated with increased intracellular levels of LCA-CoA *(Faergeman 1997)*. There is a negative correlation between human skeletal muscle LCA-CoA content and whole body insulin action *(Ellis 2000)*, most likely by reducing the activity of human skeletal muscle hexokinase *(Thompson 2000)*. Weight loss following bariatric surgery was associated with significant reductions in vastus lateralis LCA-CoA content in two groups, the first being six months following biliopancreatic surgery *(Greco 2002, Rasumussen 2002)* and the second at one year following the surgery *(Houmard 2002)*. These findings, which suggest that muscle lipid metabolism has improved, could contribute to the enhanced insulin action. There are no studies available examining these changes within the first four weeks after surgery, which is the time when the earliest improvements in glucose tolerance were shown to occur. This will be investigated in the studies proposed under **Specific Aim 1** to gain insight into whether changes in muscle LCA-CoA precede and are likely to play a role in the improved metabolic response or whether they represent one of its late manifestations.

***Omentectomy decreases insulin resistance:***

Epidemiologic *(Cefalu 1995, Shimokata 1989, Ferrannini 1997, Fujimoto 1999, Lamarche 1998, Carey 1996, O'Shaughnessy 1995)* studies (including our own) have correlated visceral fat with insulin insensitivity in both animals and humans. Several possible mechanisms have proposed this association. These include increased delivery of FFAs *(Bujalska 1997)* to the liver *(Bjorntorp 1990, Williamson 1996)* and/or abnormal expression of various cytokines (IL-6, TNF-α) and adipokines such as resistin, adiponectin, leptin and others *(Hotamisligil 1996, Steppan 2001).* Barzilai et al. *(1999)* demonstrated that surgical removal of visceral fat rapidly improved hepatic and peripheral insulin action in young rats and in rats predisposed to obesity and diabetes *(Gabriely 2002)*. Interestingly, removal of an equivalent amount of fat from the subcutaneous tissue in the same rats did not alter hepatic nor peripheral insulin sensitivities. Our recent studies in the conscious dog model (preliminary data) demonstrated that the removal of visceral fat resulted in improvement of insulin action in the absence of caloric restriction, challenging the notion that nutrient excess per se is the prominent cause of insulin resistance in obesity.

Thörne et al. *(2002)* randomized 50 non-diabetic morbidly obese subjects (BMI>35 kg/m^2^) to either adjustable gastric banding (AGB) alone or AGB plus partial removal of the greater omentum (omentectomy). The improvements in oral glucose tolerance, insulin sensitivity and fasting plasma glucose and insulin were 2-3 times greater in omentectomized subjects as compared to control subjects (P<0.05) and these were associated with a near 15% excess weight loss (p=0.07) over a two year period. There were no associated adverse effects related to omentectomy. It is important to note that the use of the adjustable gastric banding results in significantly less effective weight loss and less effective improvement in carbohydrate metabolism than RYGB and VSG. Ceelen and coworkers *(2003)* followed 625 subjects after laparoscopic AGB for three years and noted the median excess weight loss was limited to 47%. This is in comparison to a median excess weight loss up to 70% following RYGB.

1. **Rationale and Specific Aims**

**This is the “why” this study is important to conduct and what you plan to do.**

Obesity is often associated with insulin resistance. Large epidemiologic studies reveal that the risk for diabetes and insulin resistance increases from the very lean to the very obese. Studies carried out in genetically modified mice and in humans indicate that abnormal production of inflammatory cytokines contributes significantly to obesity-induced insulin resistance. Adipose tissue and especially visceral fat (and resident macrophages) release several cytokines *(Rajala 2003)* and play important roles in determining the inflammatory profile of the obese individual. A large visceral fat mass or central obesity (in contrast to gluteal or subcutaneous fat) represents a major risk for the development of type 2 diabetes mellitus (T2DM) and cardiovascular complications.

Bariatric surgery is the only modality that results in sustained weight loss and is usually associated with improvements in insulin resistance and other symptoms of T2DM. However, there is evidence to indicate that the weight loss after the surgery is not the sole mechanism behind the metabolic improvements. This is based on the findings that the improvements can often occur very early (within 10 days) after the surgery and that they precede any significant weight loss.

The degree of metabolic improvement following bariatric surgery varies greatly among individuals. The underlying mechanisms behind the improvements and the reasons for individual differences in their onset and magnitude are not well understood. Our preliminary data suggest that the improvements in weight loss and blood glucose after the operation are (a) related to distribution of body fat between subcutaneous and visceral fat, and (b) are racially biased, with African Americans and Hispanics showing blunted and more delayed responses than Caucasians. The omentum, the major deposit of visceral fat and macrophages in the abdomen, has been postulated to contribute to liver insulin resistance by increasing portal delivery of free fatty acids (FFAs), leading to inappropriately elevated hepatic glucose production, hyperinsulinemia, and eventually to pancreatic ß-cell failure. Another major reason behind this correlation is that visceral as compared to subcutaneous adipose tissue is a more important producer of several cytokines that are involved in the pathogenesis of insulin resistance. The ***central hypotheses*** of this application is that improvements in insulin sensitivity following bariatric surgery begin early in the postoperative period (10-30 days) and are mediated by changes in the secretion of energy-related peptides, while the long-term effects (greater than 1 month) are mediated by down-regulation of inflammatory factors. We speculate that these factors will exhibit racially biased trends and may underlie the different metabolic responses of African Americans and Hispanics versus Caucasians. .

Additionally, we hypothesize that the removal of the omentum in combination with bariatric surgery will speed up the reversal of insulin resistance state and will diminish the racial differences in response to bariatric surgery.

**Study Plan**

We propose a randomized study to evaluate changes in glucose, amino acid, and fatty acid metabolism at early and late stages after bariatric surgery. We will explore the mechanism(s) of changes in any of these substrates by examining alterations in the secretion and action of energy regulating peptides and of the inflammatory responses. Morbidly obese subjects will be divided into two groups with 150 participants in each arm; 75 Caucasian participants and 75 African American and Hispanics:

**Group I**: will undergo bariatric surgery (RYGB, VSG, AGB)

**Group II:** will undergo bariatric surgery (RYGB< VSG< AGB) combined with omentectomyThe study will include Caucasians, African Americans and Hispanics matched for BMI, gender and age. It will include short and long term aims with respect to the time after the surgical intervention in addition to the pre-operation basal period.

We will also obtain fat and muscle samples from normal weight individuals undergoing other abdominal surgery to serve as controls.

**Specific Aim 1: Determine the mechanism for the metabolic improvements after** bariatric surgery

Metabolic effects of the surgery will be examined at 4-weeks postoperatively, at a time when early improvements in insulin sensitivity are observed in the majority of Caucasian subjects and when changes in BMI or body composition are minimal. Subjects will also be studied at 6, 12 and 24 months when weight loss becomes significant. Subjects may also be asked to return at 3, 4, and 5 years post-operatively in order to continue to observe changes that occur in the long-term. We will evaluate which improvements following RYGB can be related to early alterations in specific energy-regulatory peptides and/or inflammatory factors and which simply follow the decrease in body weight.

**Specific Aim 2: Explore the mechanism for the blunted and delayed metabolic improvement after** bariatric surgery **in African Americans and Hispanics**

Comparison of the temporal changes in the metabolic profile will be made under matched experimental conditions for Caucasians, African Americans and Hispanics. Gene microarray analysis from two adipose tissue depots as well as skeletal muscle will be used to obtain an unbiased approach that will guide data interpretation.

**Specific Aim 3: Explore whether combining omentectomy with bariatric surgery helps accelerate and sustain post-surgical metabolic improvements especially in the more refractory African American and Hispanic populations**

Removal of the greater omentum will be combined with bariatric surgery randomly in a subgroup of subjects. We hypothesize that onset time and magnitude of the metabolic improvements (as in **Specific Aim 1**) in African Americans and Hispanics who undergo bariatric surgery combined with omentectomy will be comparable to Caucasians undergoing bariatric surgery alone (without omentectomy). As already indicated, the African American cohort of patients manifested significantly improved insulin sensitivity to glucose metabolism. We seek to test the mechanisms of improvement and to uphold the null hypothesis that the two said groups are comparable over time. In all three aims, the studies proposed include determination of the following parameters:

1. Regional fat stores and muscle distribution by body circumference measurements and DEXA scans
2. Adipocyte size, tissue macrophage content and macrophage gene expression
3. Diurnal and food-induced secretion of adipokines such as leptin, resistin and adiponectin
4. Diurnal and food-induced secretion of the energy regulating-peptides such as ghrelin and PYY
5. Kinetics of blood glucose, amino acids and fatty acids
6. Insulin responsiveness and secretion
7. Levels of inflammatory markers such as CRP, IL-6, TNF-α2R (measure of TNF-α in plasma)
8. **Animal Studies and Previous Human Studies**

**Describe the process and outcome of the animal and/or laboratory studies that have been done. Also, if any previous human studies have been done (e.g., Phase I, other phases maybe in a different population or for a different indication).**

**Preliminary studies:**

***Differences in metabolic response of African Americans and Caucasians to RYGB:* We recently** Caucasian and African American patients who underwent RYGB and analyzed body weight loss over a 24-month period. Data from 16 African American adults were compared to those for a similar group of gender and BMI-matched Caucasians. In preliminary studies**,** we found that the degree of body weight loss after bariatric surgery was significantly lower in African Americans as compared to Caucasians. African Americans lost 40±2.4 lb compared to 61±3.0 lb (p<0.0002) for Caucasian adults**.** Furthermore in another subgroup of patients (n=45) for whom blood samples were obtained immediately preoperatively (176±4 mg/dl, no difference between the two groups) and at day 10 post-operatively, we found that African American patients had significantly (p<0.005) higher fasting plasma glucose levels (135±3 mg/dl) than their white (101±2 mg/dl) counterparts matched for gender and BMI.

These findings demonstrate different metabolic responses of African Americans as compared to Caucasians to RYGB. The mechanisms underlying these differences are completely unknown and exploring them will constitute a main goal of this proposal..The work should contribute insight into some of the reasons behind differences in susceptibility to metabolic pathology. In addition, determining the earliest changes in metabolic regulators and in metabolic symptoms during the postoperative period will provide clues regarding the key determinants behind the metabolic improvements.  ***Body mass index correlates with IsoP levels in vivo:*** Our collaborator, Dr. Jason Morrow (Chief, Division of Clinical Pharmacology) and his group have carried out two separate studies showing that BMI was a major determinant of IsoP formation in humans. In the first, they determined factors associated with oxidant stress in a cohort of 298 healthy adults. Amongst a number of parameters the correlation was noted between IsoP was BMI and this association was independent of other risk factors for atherosclerosis. In a second, larger study by Dr. John Keaney *(2002)* involving over 2,800 persons enrolled in the Framingham study, a similar strong association between BMI and IsoP was found. These studies provide compelling evidence that the overweight/obese population has up-regulated inflammatory responses and is under increased oxidative stress. We propose that this is even more pronounced in African American subjects.

***Role of RYGB in reversing T2DM in morbidly obese subjects:*** Work performed by Dr. Torquati recently followed 50 patients with T2DM before and after laparoscopic gastric bypass surgery. The aim of our prospective study was to analyze the efficacy of the procedure in reversing T2DM over a 6-month period. Gastric bypass normalized HbA_1_C levels and resulted in decreased fasting plasma glucose (p<0.00, for both). Complete resolution of T2DM, defined by normal levels of fasting plasma glucose and HbA_1_C after discontinuing medical treatment, was achieved in 70% of patients with T2DM. The rest of the patients showed partial responses determined by significant decrements in daily medications.

Multivariate analysis was then used to identify preoperative variables unfavorable for complete resolution of T2DM. The predictive value of each variable in relation to response type (full or partial) was expressed in odds ratio (OR), p value and confidence interval (CI) of 95% of OR. Univariate analysis revealed that (a) treatment with insulin, (b) waist circumference > 50 inches, and (c) levels of HbA_1_C ≥ 8.0% were all associated (p<0.01) with partial responses. To eliminate the effect of confounding factors, we performed a multivariate analysis of the selected variables. The logistic regression model showed that the use of insulin and waist circumference greater than 50 inches remained statistically significant allowing the elaboration of a regression model to predict a full or partial response.

The analysis showed that patients with significant central obesity (waist circumference > 50 inches) when given insulin treatment are less likely to exhibit complete resolution of T2DM after gastric bypass surgery.

| **TABLE 1** | | |
| --- | --- | --- |
| **Variables** | **Baseline** | **6 Months** |
| **Fasting Plasma Glucose *(mg/dL)*** | 161±50 | 108±33 |
| **HbA_1_C *(%)*** | 7.5 ± 1.6 | 6.2 ± 1.2 |

| **TABLE 2** | | | | |
| --- | --- | --- | --- | --- |
| **Variables** | **χ^2^** | **P value** | **OR** | **95% CI** |
| **Age** | 2.9 | 0.085 | 0.930 | 0.85-1.01 |
| **Gender** | 0.07 | 0.81 | 0.83 | 0.2-3.9 |
| **Preop Insulin** | 9.4 | 0.002 | 9 | 2.2-36.5 |
| **Waist > 50 inches** | 11.2 | 0.001 | 40.4 | 4.6-352 |
| **BMI** | 3.2 | 0.074 | 1.08 | 0.99-1.2 |
| **Preop oral hypoglycemics** | 2.9 | 0.084 | 1.98 | 0.91-4.3 |
| **HbA_1c_ > 8.0%** | 5.9 | 0.01 | 5.1 | 1.4-18.5 |

***Role of the omentum in modulating insulin sensitivity:*** In an attempt to determine the mechanism by which omentectomy could influence insulin sensitivity *in vivo*, we resorted to the chronically catheterized conscious dog model that we have used extensively in our metabolic studies for the past 2½ decades. This model has the advantage of examining the relative contribution of the liver and peripheral tissue (mostly skeletal muscle) to glucose (and amino acid and FFA) mediated insulin sensitivity in a conscious un-sedated state *(Williams 1990)*. We have thus far finished in five dogs using sequential hyperinsulinemic euglycemic clamps conducted during pre-omentectomy versus post-omentectomy periods separated by a minimum of four weeks recovery. T**T**he amount of omental (+ visceral) fat removed averaged 550 grams.

**4.0 Inclusion/Exclusion Criteria**

**List the criteria:**

***Eligibility criteria for those having bariatric surgery*** include:

- Ages between 18-65
- Serum creatinine 1.5 mg/dL or lower
- Stable body weights for the past three months prior to their approval for the operation

***Exclusion criteria for those having bariatric surgery*** include:

- Significant hepatic enzyme elevations (more than 2 times the upper limits of normal)
- Serum creatinine >1.5 mg/dl and liver function tests greater than 2 times the upper limits of normal
- History of ketoacidosis or current metabolic acidosis
- Positive pregnancy test (β-human chorionic gonadotrophin) for females
- Intercurrent infections
- Taking drugs that are known to affect carbohydrate or lipid metabolism (e.g. steroids, high dose Niacin, β-adrenergic receptor agonists, but does not include antidiabetic drugs)
- Patients with previous bariatric surgical procedures

***Eligibility criteria for normal-weight subjects include:***

- Ages between 18-65 yrs
- BMI 22-27

***Exclusion criteria for normal-weight subjects include*:**

- Significant hepatic enzyme elevations (more than 2 times the upper limits of normal)
- Serum creatinine >1.5 mg/dl and liver function tests greater than 2 times the upper limits of normal
- History of ketoacidosis or current metabolic acidosis
- Positive urine pregnancy test for women in child bearing age.

**Enrollment/Randomization**

**List the process for randomization and/or registration; list the address, phone number or website where the registration will take place.**

***Enrollment for morbidly obese patients*:**

- Patients approved for RYGB, VSG, or AGB are reported to study coordinator
- Coordinator or study nurse screen subjects via Star Panel to determine eligibility

Subjects determined to be good candidates for the study are contacted and the study is described to them. If they show interest, detailed information, including the consent form, is sent to them to read over. The coordinator follows up to respond to questions or concerns.

***Normal Weight subjects who serve as controls:***

**Pre-study:**

- Surgery schedule is sent to study coordinator. Subjects are screened via Star Panel to determine potential candidates. Surgeon is contacted to confirm subjects are suitable.

**Day of Surgery:**

- Subject is met in pre-op area and told about study. Subject is asked if he/she would be willing to let surgeon take muscle and fat samples during the operation. If subject agrees, he/she is consented and surgeon is notified.

**Randomization:** The randomization schedule will be generated by statistical software. The randomization code will be generated using a permuted block size of four. No other restrictions will be used. As each subject is recruited, the randomization is assigned by the coordinator and the surgeon is notified. The PI, Dr. Naji Abumrad, will not be involved in the selection and allocation of patients. All recruited patients and researchers involved in the evaluation of the end-points will be blinded to the treatment assigned throughout the whole study period. No interim analyses will be carried out during the course of this study.

1. **Study Procedures**

**Provide a description of what the participants will experience. For example, a description of the instructions that will be given to the participants, activities in which they will engage, the length and timing of involvement, and the circumstances under which they will provide data (e.g., phone calls, spending time in an uncomfortable position, group assessments, one-on-one interview, videotaping, audiotaping, etc.)**

***Subjects who participate in metabolic chamber (the subjects will be assigned to the metabolic chamber in the preoperative period and will have chamber studies again at 6-, 12-, and 24-months post-operatively, as well as 3, 4, and/or 5 years post-operatively, unless otherwise noted):***

**Study Day 0:** Subject reports to the GCRC in the afternoon. The following are performed:

- Measurements of Height, Weight, and Body Circumferences.
- History and Physical Examination
- Performance of EKG (if over age 40)
- Performance of serum pregnancy test (if fertile female)
- Answer SF-36 Questionnaire
- Orders will be written for no food and caffeine after 20:00. They will be made completely NPO after 24:00

**Study Day 1: The following tests will be performed:**

- DEXA (except 1-month post op)
- IV placed and labs drawn (blood for serum potassium, and for complete blood count, hematocrit, liver function tests, HGB A1c, homocysteine)
- Subject will be escorted to metabolic chamber for 24 hour stay. Collection of 24-hour urine will also begin.
- Blood samples taken hourly from 0730 to 2030 to measure various plasma factors associated with obesity.

**Study Day 2:**

- Subject escorted from chamber to study room
- IVs placed
- Euglycemic-hyperinsulinemic clamp is begun
- Stable isotope tracer studies for amino acid and fat kinetics begun
- Muscle/fat biopsies are performed about 1 hour after study begins
- Prior to basal sampling period and prior to high-insulin sampling period, subject will be placed on metabolic cart for ½ hour of breathing testing
- Subjects will be given activity monitors to wear for the week after the study (one on wrist and one on waist) along with SASE to mail them back
- At end of study, subject is fed and monitored for at least an hour to assure blood glucose stability

**Subject will have operation, with or without omentectomy, then repeat the above procedures 1-, 6-, 12, and 24-months later. Subjects may also be asked to repeat these procedures at 3, 4, and 5 years post-operatively.**

***Subjects who DO NOT participate in the metabolic chamber (the following procedures will be performed in the preoperative period and at 1-, 6-, 12-, and 24-months post-operatively, as well as 3, 4, and/or 5 years post-operatively, unless otherwise noted):***

**Pre-study and Study Day 0 are the same, except subject is requested to collect 24 hour urine at home and bring the bottle(s) with them when they check into the GCRC.**

**Study Day 1 is like Day 2 (clamp study day) above, except:**

- DEXA is performed this day (prior to clamp study)
- Initial labs are drawn this day

**Subject will have operation, with or without omentectomy, then repeat the above procedures 1-, 6-, 12, and 24-months later. Subjects may also be asked to repeat these procedures at 3, 4, and 5 years post-operatively.**

1. **Risks**

**List all adverse effects observed in animal studies, previous human studies, or laboratory observations. List the frequency expected for the side effects and a statement that there may be unknown or unanticipated adverse effects.**

- If subject is uncomfortable in enclosed, small spaces, the metabolic chamber may cause anxiety
- IVs may cause slight discomfort, swelling, bleeding, bruising, clotting, or infection
- In the event of an arterial line being placed, insertion of catheter may cause bleeding, bruising, nerve damage, or infection
- Rarely, the insertion of a needle into an artery or vein may cause damage to the artery or vein and a clot may develop.
- There is a small risk of low blood sugar
- Lidocaine (used for biopsies) may cause burning and/or local rash or irritation. There is also a risk of heart rhythm problems. Although rare, it is possible that lidocaine could cause a tingling feeling around the mouth, dizziness, blurred vision, drowsiness, confusion, and/or seizures.
- There is exposure to radiation (from DEXA and tritiated glucose) equivalent to 3 months of radiation from natural surroundings
- The muscle/fat biopsies may cause soreness, bruising, infection, nerve damage or a hematoma.
- Removal of the omentum may increase chances of bleeding and will increase time under anesthesia
- As with any medical treatment, there may be unknown or unforeseeable risks associated with taking part in this research study.

1. **Reporting of Adverse Events or Unanticipated Problems involving Risk to Participants or Others**

**Adverse event grading and attribution scale:**

A serious adverse event (AE) is defined as either fatal or life-threatening, requires inpatient hospitalization or prolongation of an existing hospitalization, results in persistent or significant disability/incapacity, is medically significant or requires intervention to prevent one or other of the outcomes listed above.

Grading scale for adverse event intensity:

- ***Mild:*** Discomfort noticed but no disruption of normal daily activity.
- ***Moderate:*** Discomfort sufficient to reduce or affect normal daily activity.
- ***Severe:*** Incapacitating with inability to work or perform normal daily activity.

Attribution scale for AE reporting:

- ***Probable:*** Adverse event is related to the procedures (insulin clamp, muscle biopsy, omentectomy), including pain, bleeding, perforation of contiguous organs, infection, and death, if death resulted from one of the aforementioned complications.
- ***Possible:*** Adverse event follows the procedures within a reasonable period (within 7 days), but may have been produced by the subject's clinical state or other factors.
- ***Remote:*** Adverse event does not follow the procedures within a reasonable period (more than 7 days) and could readily have been produced by the subject's clinical state or other factors.
- ***Unrelated:*** Adverse event is judged to be clearly due to extraneous causes and does not meet the above criteria.

**AE reporting:**

- All study-related mild and moderate adverse events will be evaluated by the Principal Investigator and/or the Safety Officer or sub-PI within 72 hours; serious AEs will be evaluated within 24 hours, and promptly as needed.
- Serious adverse events will be reported to Human Subjects/IRB no later than 10 days after becoming aware of the event.
- Annual reporting of adverse events will be conducted with the Human Subjects annual review/renewal according to their protocol.

**Safety Reports:**

- The study coordinator will produce quarterly administrative reports that describe study progress including accrual, demographics, and subjects’ status. Reports will also describe adherence to inclusion/exclusion criteria and study protocol, and any adverse events.
- Study-related adverse events will be monitored by the study coordinator, sub-investigators, or Principal Investigator, until resolved.
- The Principal Investigator and study coordinator will each be responsible for ensuring that adverse events are reported to the IRB and Safety Officer in compliance with their requirements.
- The Safety Officer as well as study physicians will be alerted if any unexpected serious (e.g., injury which requires medical treatment) adverse event occurs, to determine whether changes in the study protocol (e.g., additional safety measures, change in exclusion criteria) are needed.
- The Principal Investigator will notify the IRB for any changes needed to the protocol. In addition,
- The Principal Investigator will review all data collection forms on an annual basis for completeness and accuracy of the data, as well as protocol compliance, and a statement reflecting the results of the review will be sent to the NIDDK in the annual report and to the IRB at the time of Continuing Review.

1. **Study Withdrawal/Discontinuation**

**List the process for withdrawing from the study. List the process for being withdrawn from the study and the indications for withdrawal.**

**At any time, subjects may withdraw from the study (i.e., withdraw consent to participate) at their own request. A subject’s participation in the study may be discontinued at any time at the discretion of the Investigator and in accordance with his/her clinical judgment. No disadvantage will arise for any subject who withdraws consent for participation at any time or who is withdrawn from the study by the Investigator.**

Reasons for discontinuation of study treatment will be recorded on the appropriate page of the CRF in any case and may include the following:

- Subject's request for withdrawal
- Investigator's decision that discontinuation is in the best interest of the subject
- Non-compliance with the regimen and timing that might result in dropping out from the study
- Development of an intolerable adverse event due to study participation as determined by the Investigator, subject, or both.
- Development of an intercurrent illness, condition, or procedural complication, which would interfere with the subject's continued participation.
- Subject is lost to follow-up

**If a subject fails to return for a scheduled visit, a documented effort must be made to determine the reason. This information should be recorded in the study records.**

Based on IRB recommendations, any adverse event that is identified and has not been addressed in the consent form must be added. The Principal Investigator will discontinue implementation of a protocol for the following reasons:

- There is clear evidence of harm or harmful side effects of the treatment or procedure;
- In the event that a serious adverse event occurs that increases the risk to participants, the study will be stopped and an investigation will be conducted and a findings report generated to the investigators, IRB, and the NIDDK;
- There is overwhelming evidence of the benefit of the treatment versus the control arm.

1. **Statistical Considerations**

**Describe the statistical power of the study, the confidence intervals and the method for analysis. Describe any possible deviations and their statistical impact.**

***Statistical Analysis:*** This protocol will provide information regarding basal carbohydrate, lipid and amino acid metabolism. Insulin sensitivity of hepatic glucose production will be determined using the 0.6 mU/kg.min insulin infusion rate. Peripheral glucose utilization will be determined using the 2.0 mU/kg.min insulin infusion rate *(Bonadonna 1990)*. The study will also provide information regarding insulin sensitivity of fatty acid metabolism (lipolysis and re-esterification). Studies in obese subjects and type 2 diabetic subjects demonstrated a right shift in the rates of insulin-stimulated suppression of lipolysis, hepatic glucose production and stimulation of peripheral glucose uptake. The insulin levels required were most sensitive for suppression of lipolysis followed by suppression in glucose production and then by insulin of stimulated glucose uptake *(Matthaei 2000)*.

***Statistical Methods, Sample Size, and Power Estimates:*** We based sample size estimates on historical case volumes (>600 procedures per year), patient demographic distributions (70% Caucasian, 25% African American, 5% Hispanic, 80% female 20% male). Persons who consent to participate will be enrolled in both the short and long term phases of the study. We assumed attrition for the short-term phase, which entails data points through postoperative week four, to be negligible, and for the long-term study, which entails a two-year postoperative follow-up period, to be ~ 25% by postoperative month 24. We based this rate on reported attrition following bariatric surgery over a comparable time, and the rate is assumed to be comparable between experimental groups. Anticipated effect sizes were based on previous research or standard conventions for large, medium, and small effects *(Cohen 1988)*. Unless noted, power estimates were determined using standard methods and software (Power and Precision™ v. 2.0, nQuery Advisor™ v. 3.0) for non-directional tests at the 0.05 alpha level.

Demographic information such as age, race, and gender will be tabulated. Descriptive statistics, including means, standard deviations, and ranges for continuous measures, as well as percentages and frequencies for categorical measures will be presented. Statistical analyses will be performed using standard packages (SPSS or SAS).

**Specific Aim 1:** The short-term phase of the investigation will include a group of 66 Caucasians and group of 66 African Americans and Hispanics (each of the two groups randomized to 33 bariatric surgery and 33 bariatric surgery + omentectomy). The long-term phase of the investigation will include at least 100 patients (25 in each of the four racial and procedure groups).

Initial analyses will examine the short-term effects of race and surgical procedure (bariatric surgery + omentectomy), and the interaction effect of race by surgical procedure on change in measures of body composition, inflammatory factors, hormones, energy-related peptides, and health-related quality of life following bariatric surgery. The general case statistical method is mixed-model repeated measures analysis of variance with tests of: (a) the within-subjects effect of time; (b) the between-subjects main effects race and surgical procedure; and (c) race by procedure by time interaction effects. Simple contrasts will be used to test effects at postoperative week four in relation to preoperative measure. Statistical tests of main and interaction effects of race and omentectomy on any contrast are analogous to interaction of these effects with the within-subject change scores (simple contrasts). Assuming a within-subject correlation between longitudinal data points that is as low as 0.35 in the combined sample (n=132), power is adequate (85%) to detect a small effect (d=0.3) of time for any contrast. Power to detect, for any contrast, medium (f=0.30) overall main effects of race and omentectomy and the race by omentectomy interaction effect is 92%. This statistical design will also be applied within each racial group (n=66) to more explicitly examine the effect of omentectomy, and within each surgical group (n=66) to more explicitly examine the effect of race. For these analyses, the power to detect medium to large (d=0.70) effects for any contrast is 80%.

The long-term data, which entails five data points (preoperative, 1 month, 6 months, 12 months, and 24 months). Mixed effects modeling will be employed to determine whether patterns of individual change in outcome measures vary due to race, surgical procedure (bariatric surgery + omentectomy), and clinical covariates. Advantages of this method are that: (a) data from individuals with missing data may be included providing that the point(s) can be considered ‘missing at random’; (b) between-subject variation in the timing of assessment points can be accommodated (i.e. time is treated as a continuous variable); and (c) continuous or discrete time-sensitive covariates (e.g. weight loss, syndrome X symptom expression) can be incorporated. Follow-up analyses will be conducted on the sample having complete data through month 24 (expected n=100, 25 in each of four unique race by surgical procedure conditions) using methods described above for the short-term phase of the investigation. Simple and repeated contrasts will be employed to test within-subjects effects of time in relation to the preoperative measurement and the preceding time point, respectively. Assuming a within-subject correlation as low as 0.45, power is adequate (83%) to detect a small effect (d=0.3) of time on any contrast. Power to detect, for any contrast, medium (f=0.30) overall main effects of race and omentectomy and the race by omentectomy interaction effect is 84%. The power to detect large (d=0.81) effects of surgical procedure within each racial group (and of race within each surgical procedure group) for any contrast is 80%.

**Specific Aim 3:** Analyses addressing **Specific Aim 3** will be performed using the statistical methods described above for both the short and long term phases of the investigation (mixed-model repeated measures analysis of variance and mixed effects modeling). Two subject groups will be included in these analyses: (a) African American and Hispanic patients receiving bariatric surgery plus omentectomy, and (b) Caucasian patients receiving bariatric surgery without omentectomy. Our hypothesized mechanism for the effects of omentectomy and race implies that these two groups will experience comparable outcomes over time. Given our previous data, which demonstrated a large effect of race (d > 1.5) on weight reduction following bariatric surgery, and our hypothesis that omentectomy will blunt the disparity in African Americans and Hispanics, effect sizes were set at > 0.81. The power to detect an effect of group (d=0.81) on a within-subject contrast (change over time) in the short-term phase of the study (33 patients per group) is 90%. Power is 80% to detect a comparable effect (d=0.81) on any contrast in the long-term study (minimum group size = 25). In order to analyze the effect of omentectomy independent of race, a sample size of 11 participants per group would allow us to detect a 25% difference in insulin sensitivity between groups, with an *alpha* value of 0.05 and power of 80% (*beta* = 0.2). This is based on data on glucose kinetics obtained previously in obese participants (Klein 2004). A difference of this magnitude is clinically meaningful because it represents the lower end of the observed effect of current treatment strategies for insulin resistance, such as moderate weight loss (Kelley, 1993; Kirk 2009; Tiikkainen 2004; Ross 2000) and thiazolidinedione therapy (Schrauwen-Hinderling 2008; Li 2007; Coletta 2009) which increase hepatic and skeletal muscle insulin sensitivity by 25-50%.

1. **Privacy/Confidentiality Issues**

**Discuss the methods for ensuring participant privacy, and the methods for protecting privacy.**

Only research associates in our laboratories or those individuals directly involved with the study will have access to data. Information is for research purposes only and will be used for publication purposes. All participants will have their names concealed.

Access to identified patient information will be limited to the investigators listed within this IRB application. De-identified information with HIPAA identifiers removed will be available to other investigators following IRB approval. Confidentiality and security will be maintained for the database. The database is stored behind a firewall (in addition to the institutional firewall) with the highest level of protection, i.e. the same level of protection as the on-line hospital information system at Vanderbilt. This means that users must logon to a web server that sits between the institutional firewall and the firewall to the database, and only this application server is allowed to query the database. Only users approved through our institutional review board will be allowed access to patient identifiers. Other levels of authorization may exist for future approved users following IRB approval, e.g. access to de-identified data.

1. **Follow-up and Record Retention**

List the duration of the study. List the duration of record retention and the method for destruction or the possibility of indefinite archival of information.

The study is scheduled to last for 5 years. The samples and records will be stored for investigation for at least six years and may be indefinitely.

**Table of Events**

|  | **Before Surgery**  **(GCRC)** | **Surgery** | **4 wks**  **post-op** | **6 mon**  **post-op** | **12 mon**  **post-op** | **24 mon**  **post-op** | **3, 4, 5 yrs post op** |
| --- | --- | --- | --- | --- | --- | --- | --- |
| **History and Physical/Labs** | ✓ |  | ✓ | ✓ | ✓ | ✓ | ✓ |
| **DXA** | ✓ |  | ✓ | ✓ | ✓ | ✓ | ✓ |
| **Metabolic Chamber^1^** | ✓ |  |  | ✓ | ✓ | ✓ | ✓ |
| **Chamber Blood Samples^2^** | ✓ |  |  | ✓ | ✓ | ✓ | ✓ |
| **24 hour Urine** | ✓ |  | ✓ | ✓ | ✓ | ✓ | ✓ |
| **Metabolic Studies^3^** | ✓ |  | ✓ | ✓ | ✓ | ✓ | ✓ |
| **Omentum Biopsy^4^** |  | ✓ |  | ✓ | ✓ | ✓ | ✓ |
| **Muscle Biopsy** | ✓ | ✓ | ✓ | ✓ | ✓ | ✓ | ✓ |
| **Subcutaneous Fat Biopsy** | ✓ | ✓ | ✓ | ✓ | ✓ | ✓ | ✓ |
| **SF 36 Questionnaire** | ✓ |  | ✓ | ✓ | ✓ | ✓ | ✓ |

^1^ Only subjects enrolled to be in the metabolic chamber

^2^ Only subjects enrolled to be in the metabolic chamber

^3^ May include insulin clamp (using tritiated glucose and/or deuterated glucose), amino

acid infusion for protein kinetics, and glycerol/palmitate infusion for lipolysis and FFA

kinetics

^4^ Only if other abdominal operations are needed
