# Supplementary material for: Reduction in Inflammatory Gene Expression in Skeletal Muscle from Roux-en-Y Gastric Bypass Patients Randomized to Omentectomy
Source: PLoS One. 2011 Dec 16;6(12):e28577. doi: 10.1371/journal.pone.0028577 (PMC3241684; doi:10.1371/journal.pone.0028577)
Supplement: Table S2 — Expression values for genes changed greater than 4 fold between 6 vs. 0 months, no omentectomy; 12 vs. 0 months, no omentectomy; 6 vs. 0 months with omentectomy; and 12 vs. 0 months, with omentectomy. (PDF) [file pone.0028577.s004.pdf]

Supplemental Table 2. Gene expression values changed greater than 4 fold.							
**Affymetrix ST 1.0 Array ID**	**0-No**	**6-No**	**12-No**	**0-Yes**	**6-Yes**	**12-Yes**	**Selected for TLDA validation**
8177277_RBMY2EP	4.80685	3.122865	3.440672	2.462251	3.371255	3.441513	
7933192_HNRPA3P1	8.510757	7.32415	7.104011	6.302529	6.594611	7.076342	
7924821_gm127	4.064403	2.170289	1.881405	2.047589	2.066068	2.186831	
8078918_SNORA62	7.51971	7.281384	8.218526	6.808687	6.836918	5.998769	
7962827_SNORA2A	6.199946	5.857545	7.095224	5.029156	5.425472	5.045056	
8086330_AXUD1	7.539693	6.40331	6.276914	8.580238	6.514306	6.498477	
7960865_SLC2A3	8.558565	7.155959	7.138912	9.925293	7.315548	7.409976	*
8062461_LBP	6.286387	5.212593	5.207602	7.377002	5.208519	5.241776	*
8131803_IL6	6.572118	4.298875	3.994312	9.20948	4.191224	4.131359	*
8065353_THBD	8.380108	7.252122	7.300261	10.15004	7.344264	7.534771	
8018864_SOCS3	7.390345	5.788718	6.00757	9.394246	5.991076	6.031529	*
8028652_ZFP36	8.770538	7.499611	7.605748	9.742706	7.381183	7.527718	*
7922229_SELE	5.4181	4.115772	4.101108	6.422738	3.961088	3.99727	*
8148317_MYC	8.694508	6.689812	6.684913	9.890925	6.802518	6.759993	*
7909610_ATF3	6.150059	5.011442	4.857962	7.030092	4.797831	5.057142	*
8006433_CCL2	8.090573	5.753876	5.997232	9.892403	6.032413	6.673797	*
8055952_NR4A2	6.371752	5.120424	5.204181	7.01383	4.901578	5.416981	
8024485_GADD45B	8.762878	7.112932	7.4543	9.710638	6.899641	7.231446	
8025828_LDLR	7.551499	6.668352	6.744151	8.694988	6.803265	6.47048	
8069676_ADAMTS1	7.485305	6.225791	6.256622	9.018669	6.244499	5.600326	
7954090_EMP1	8.039775	6.908692	7.437132	9.176338	7.00295	7.030322	
7982597_THBS1	7.204099	5.091434	5.93718	8.742728	5.727201	5.614112	*
7995806_MT1A	8.134527	7.166582	7.689427	9.871501	7.3113	7.394695	
7995787_MT1M	5.978323	4.848859	5.551747	7.50469	5.013453	5.263241	
8119088_CDKN1A	7.242183	6.294436	6.662832	8.00008	5.973511	6.232096	
8135069_SERPINE1	6.657064	5.812365	5.85717	8.362505	5.686234	5.795318	
7934916_CH25H	5.362375	4.79632	4.684476	6.996476	4.429471	4.570127	*
8162276_NFIL3	6.329495	5.64381	5.782287	7.884641	5.246184	5.414359	*
8095680_IL8	5.897976	5.341805	5.117891	7.110512	5.090364	5.459977	*
8122265_TNFAIP3	5.849085	5.521491	5.580415	7.643896	5.566999	5.484686	*
7921821_ADAMTS4	6.092053	5.468916	5.466109	8.154138	5.328287	5.662237	
8025601_ICAM1	6.756937	6.393631	6.274061	8.733776	6.475596	6.775353	
8156848_NR4A3	8.099947	5.349873	5.370732	9.016953	5.362062	5.331358	*
8029693_FOSB	8.151516	5.977442	6.025079	8.817471	6.031658	6.151453	*
8100994_CXCL2	6.671572	4.678912	4.818462	7.235061	4.617105	4.988464	*
7955589_NR4A1	9.449126	7.551409	7.632787	9.822363	7.454026	7.776206	
7902687_CYR61	8.880627	6.09381	6.273723	8.932739	5.985374	6.418232	*
8070665_SNF1LK	8.290503	6.896962	6.344887	8.853451	6.469741	6.887445	
7908650_DKFZp434B1231	7.949509	5.697185	5.393947	8.434153	5.327543	6.099721	* (IGFN1)
8026047_JUNB	9.293178	7.271778	6.906909	10.38707	6.827059	6.95755	*
7975779_FOS	10.57765	7.430259	6.48291	11.66186	6.538942	6.597856	*
8108370_EGR1	10.80861	8.262308	7.754484	11.49982	7.02523	7.29497	*
8163002_KLF4	7.328327	6.011024	6.559502	8.030248	6.353899	5.98921	*
7934979_ANKRD1	10.04154	6.880675	8.563455	11.09228	8.515297	7.59908	*
8084206_B3GNT5	5.200742	5.2751	4.990438	7.003935	5.581347	4.751616	

Supplemental Table 2. Gene expression values changed greater than 4 fold.

Affymetrix ST 1.0 Array ID	0-No	6-No	12-No	0-Yes	6-Yes	12-Yes	Selected for TLDA validation
8042788_ACTG2	6.832311	5.304186	4.648211	5.931125	4.828603	5.087222	
7921690_ITLN1	5.735618	3.333037	3.498801	4.553938	3.268131	3.538781	*
8175531_CDR1	9.056747	6.546371	5.693292	8.43884	6.643586	7.088392	*
8106573_THBS4	10.34515	8.109584	8.275132	10.1714	8.606795	8.910921	
8175311_CXorf48	5.806885	3.713603	4.654748	5.100195	4.146898	4.521109	
8086538_LOC644714	5.599468	4.351937	4.099707	5.02061	3.552668	2.884873	
7929816_SCD	6.044382	5.579355	6.807779	7.409997	4.718183	5.818217	
7920873_SNORA42	9.00288	9.065637	9.69422	9.829914	7.691227	8.2898	
8084710_ADIPOQ	6.652029	5.050607	7.386399	7.43758	5.178266	5.982008	*
8172204_MAOB	7.000094	9.08445	8.611274	8.012955	8.795534	8.489779	
8085972_GADL1	4.765821	7.026444	6.326088	5.806799	6.522945	6.299081	
7981990_PWCR1	7.560956	10.09714	9.457023	8.297421	10.0244	10.00622	
7964246_SNORD59B	4.904507	7.402366	6.834342	5.578261	7.184622	7.083579	
8048014_RPE	4.137071	6.044708	6.313691	4.883709	6.615355	5.941701	
8100076_YIPF7	4.604855	6.148519	6.079689	4.579904	6.650743	6.063931	
8152297_ANGPT1	4.704666	6.313934	5.670928	5.109509	7.121245	5.92354	
8176276_ATRX	4.807721	5.916878	5.88197	3.963777	6.58672	5.746941	
7903171_RWDD3	5.353331	5.977451	6.043171	4.516362	6.554813	5.697886	
7971653_DLEU2	5.351854	6.803867	6.876899	4.535005	6.757162	6.079217	
8036813_ZNF780B	6.145348	6.995065	6.385077	5.224241	7.400749	6.154045	
7982056_SNORD115-25	5.203169	6.588611	7.973662	6.177649	6.490047	6.190371	
7899480_SNORA73A	7.67704	9.235293	10.16249	8.76265	8.890179	9.008179	
7922410_SNORD44	3.65333	4.832745	5.761621	4.458853	5.326138	4.546572	
7942476_PAAF1	4.523174	6.396067	6.538217	5.659863	6.12923	5.687025	*
7964927_TSPAN8	5.448067	7.252806	7.658467	5.376956	7.15762	7.176741	
7955858_HOXC10	6.880888	8.936766	8.958694	6.700663	8.572292	8.859509	*
8090872_KY	6.278491	8.344924	8.159504	6.40702	7.698461	8.085257	
8086344_CX3CR1	4.645247	6.1487	6.772928	5.155847	5.955734	6.268933	*
8150877_SNORD54	6.451601	8.223743	8.517153	6.360693	7.253968	7.656013	
7982094_SNORD115-44	4.851773	7.28913	7.609309	4.9387	6.004126	6.570806	
7982090_SNORD115-6	6.987512	8.683914	9.264561	7.298702	8.348167	8.238748	
7982018_SNORD115-6	6.842948	8.608117	9.14786	7.255433	8.288251	8.112522	
7948902_SNORD29	5.424897	7.207188	7.771396	5.733396	6.846253	6.581488	
7982084_SNORD115-12	8.160503	9.866423	10.19704	8.370279	9.339622	9.109344	
7982064_SNORD115-43	6.873795	8.621606	9.326948	7.069919	8.375408	7.939776	
7982028_SNORD115-43	6.873795	8.621606	9.326948	7.069919	8.375408	7.939776	
7982078_SNORD115-11	6.873795	8.621606	9.326948	7.069919	8.375408	7.939776	
7982092_SNORD115-9	6.873795	8.621606	9.326948	7.069919	8.375408	7.939776	
7982058_SNORD115-26	6.885887	8.678682	9.350237	7.080369	8.397387	7.980684	
7982050_SNORD115-12	6.590799	8.211433	9.127142	6.533457	7.990268	7.631471	
7982024_SNORD115-12	6.737932	8.331493	9.284879	7.020904	8.361834	7.845249	
7982016_SNORD115-5	6.737932	8.331493	9.284879	7.020904	8.361834	7.845249	
7982030_SNORD115-5	6.737932	8.331493	9.284879	7.020904	8.361834	7.845249	
7982052_SNORD115-23	4.80685	6.55149	7.845061	4.94208	6.645257	5.704524	
7982020_SNORD115-7	3.424789	5.185725	6.429635	3.542834	5.350282	4.105939	

Supplemental Table 2. Gene expression values changed greater than 4 fold.							
**Affymetrix ST 1.0 Array ID**	**0-No**	**6-No**	**12-No**	**0-Yes**	**6-Yes**	**12-Yes**	**Selected for TLDA validation**
7982032_SNORD115-16	6.227508	8.221359	8.913842	6.973832	7.520998	7.457349	
7982008_SNORD115-13	6.227508	8.221359	8.913842	6.973832	7.520998	7.457349	
7982038_SNORD115-1	6.227508	8.221359	8.913842	6.973832	7.520998	7.457349	
7945801_SNORA54	6.836258	7.388804	8.671158	6.417747	7.055333	6.548425	
7982046_SNORD115-20	4.859962	6.130544	7.420487	4.39804	5.780389	5.337734	
7982070_SNORD115-32	5.876432	5.709281	7.798697	6.026777	6.501827	6.058663	
7931832_AKR1C2	7.142639	7.82648	9.344638	8.681243	8.485197	8.169376	
8012949_FBXW10	3.211884	5.521269	5.27685	4.130258	3.989397	5.691766	*
7948910_SNORD25	2.377039	4.473607	2.849568	3.189246	3.781446	4.432712	
7925525_CEP170	5.10986	6.63482	4.537164	5.67205	6.527928	5.029363	
7957023_LYZ	6.47368	5.155236	6.849636	6.121959	7.168526	7.163974	
7920244_S100A8	7.059299	5.333441	6.849293	6.809156	7.459221	7.209466	
8048227_IL8RB	4.734203	3.785798	5.326231	4.017436	5.988665	5.119635	
