# Supplementary material for: Reduction in Inflammatory Gene Expression in Skeletal Muscle from Roux-en-Y Gastric Bypass Patients Randomized to Omentectomy
Source: PLoS One. 2011 Dec 16;6(12):e28577. doi: 10.1371/journal.pone.0028577 (PMC3241684; doi:10.1371/journal.pone.0028577)
Supplement: Table S3 — TLDA probes utilized in this study. (PDF) [file pone.0028577.s005.pdf]

Supplemental Table 3. TLDA probes utilized in this study.

Gene Symbol	Assay ID	RefSeq ID	Amplicon length
18S	Hs99999901_s1	X03205.1	187
FOS	Hs99999140_m1	NM_005252.3	77
EGR1	Hs00152928_m1	NM_001964.2	72
JUNB	Hs00357891_s1	NM_002229.2	89
MYC	Hs99999003_m1	NM_002467.4	65
ITLN1	Hs00914745_m1	NM_017625.2	71
HOXC10	Hs00213579_m1	NM_017409.3	60
ANKRD1	Hs00173317_m1	NM_014391.2	63
FOSB	Hs00171851_m1	NM_001114171.1	106
CDR1	Hs00601346_s1	NM_004065.2	86
CCL2	Hs00234140_m1	NM_002982.3	101
IL6	Hs00985641_m1	NM_000600.3	89
NR4A3	Hs00175072_m1	D78579.1	60
CYR61	Hs00155479_m1	NM_001554.4	88
ADIPOQ	Hs00605917_m1	NM_004797.2	71
THBS1	Hs00962908_m1	NM_003246.2	59
SLC2A3	Hs00359840_m1	NM_006931.2	112
CH25H	Hs02379634_s1	NM_003956.3	155
CXCL2	Hs00236966_m1	NM_002089.3	68
SOCS3	Hs02330328_s1	NM_003955.3	89
IL8	Hs99999034_m1	NM_000584.2	81
LBP	Hs00188074_m1	NM_004139.2	113
SELE	Hs00174057_m1	NM_000450.2	83
NFIL3	Hs00356605_g1	NM_005384.2	80
TNFAIP3	Hs00234713_m1	NM_006290.2	63
ATF3	Hs00910173_m1	NM_001030287.2	78
ZFP36	Hs00185658_m1	NM_003407.2	105
KLF4	Hs01034973_g1	NM_004235.4	74
IGFN1	Hs00297580_m1	AL137493.1	81
PAAF1	Hs00228523_m1	NM_025155.1	80
CX3CR1	Hs00365842_m1	NM_001337.3	84
