# Supplementary material for: Reduction in Inflammatory Gene Expression in Skeletal Muscle from Roux-en-Y Gastric Bypass Patients Randomized to Omentectomy
Source: PLoS One. 2011 Dec 16;6(12):e28577. doi: 10.1371/journal.pone.0028577 (PMC3241684; doi:10.1371/journal.pone.0028577)
Supplement: Table S1 — Expression values for genes changed greater than 2 fold between 6 vs. 0 months, no omentectomy; 12 vs. 0 months, no omentectomy; 6 vs. 0 months with omentectomy; and 12 vs. 0 months, with omentectomy. (PDF) [file pone.0028577.s003.pdf]

Supplemental Table 1. Gene expression values changed greater than 2 fold.

Affymetrix ST 1.0 Array ID	0-No	6-No	12-No	0-Yes	6-Yes	12-Yes
7897378_PER3	7.712401	9.442497	9.192862	7.966899	9.374891	9.148988
7897441_H6PD	6.972074	8.455997	7.942984	7.587202	8.361097	8.536241
7898184_ELA2B	6.531908	5.506136	5.87053	6.257228	5.605608	5.765348
7898211_DDI2	7.841185	9.083575	8.830819	8.050409	8.992342	8.87251
7898249_SLC25A34	7.022054	8.038309	7.832349	7.740161	7.724892	8.029376
7898750_ZBTB40	5.651703	6.761204	6.388281	6.040879	6.349188	6.426052
7898809_EPHB2	6.675691	5.339785	5.820138	6.360767	5.418223	5.682487
7899018_TMEM57	7.091779	8.161407	7.740479	7.546247	8.029388	8.009798
7899134_CCDC21	8.12105	9.232584	8.764424	8.368528	9.21834	9.10297
7899480_SNORA73A	7.67704	9.235293	10.16249	8.76265	8.890179	9.008179
7899499_RAB42	5.083649	4.024024	3.724668	4.407245	3.418161	4.097682
7899829_S100PBP	5.532532	6.594707	6.571042	5.463645	6.532047	6.550624
7899943_ZMYM1	4.489073	5.729646	5.568569	5.172237	5.852386	5.123255
7900639_WDR65	4.963748	3.924896	4.00947	4.444339	3.987656	4.067546
7900888_IPO13	8.478042	9.606329	9.229533	9.051744	9.526773	9.569528
7900979_C1orf164	6.577703	7.701122	7.282383	7.253912	7.451965	7.408103
7901050_SNORD38A	5.536177	6.960075	6.1626	5.377732	5.720782	5.592023
7901052_SNORD38B	3.52077	4.89827	4.540081	4.106845	4.776683	4.127563
7901447_PRPF38A	6.304611	7.54671	7.441687	7.052757	7.890058	7.507276
7901479_ZYG11B	9.416599	10.44664	9.876553	9.605691	10.40965	10.12685
7901513_SCP2	7.585578	8.609533	8.540633	7.96253	8.580132	8.239998
7901549_CPT2	5.941399	7.094528	6.816711	6.524556	6.894964	7.251828
7901720_PRKAA2	9.224003	10.80478	10.27502	9.684426	10.92737	10.34443
7901748_FLJ10986	7.088806	8.240517	8.074528	7.29676	8.18076	8.216621
7901804_INADL	7.43561	8.488841	8.047464	7.1206	8.438684	7.920391
7902308_FPGT	5.430799	6.434458	5.933953	5.547908	6.908981	5.78975
7902398_SNORD45A	7.609259	9.201031	9.058615	7.074402	8.308164	8.510227
7902660_WDR63	4.871388	3.799009	3.569521	4.181886	3.912667	4.06029
7902687_CYR61	8.880627	6.09381	6.273723	8.932739	5.985374	6.418232
7902992_RPAP2	5.446306	6.672682	6.328497	5.742944	6.532559	6.160312
7903022_SNORD21	6.661381	8.011631	8.559915	6.917399	7.225257	7.031159
7903032_MTF2	5.843695	7.52298	6.866727	6.416734	7.559393	7.025429
7903092_FNBP1L	5.388411	6.940162	6.447353	5.969518	6.893336	6.075122
7903119_ABCD3	7.038216	8.203807	8.014683	7.447354	8.388824	7.77713
7903239_AGL	9.758735	10.90979	10.31507	10.17174	10.96077	10.37436
7903308_CCDC76	4.961726	6.084066	5.902998	5.536613	6.671669	5.938532
7903414_AMY2A	6.983387	8.220758	7.957669	6.833988	8.57758	8.178908
7903719_AMPD2	6.724887	5.348964	5.928594	6.01209	6.10405	6.029334
7903908_CEPT1	6.278512	7.351043	7.445953	6.292813	7.275163	7.063796
7903972_ATP5F1	6.782824	7.961823	7.622641	6.999607	7.746897	7.80989
7904086_LRIG2	5.656481	6.741579	6.570188	5.942444	7.102684	6.621468
7904965_PDE4DIP	8.791035	10.19039	9.773458	8.851847	10.6144	9.268391
7905099_VPS45	6.543622	7.936934	7.548269	6.829583	7.877841	7.417967
7905131_CA14	5.098902	7.055441	6.50269	4.597713	6.040648	5.962478
7905299_PRUNE	6.725837	7.874307	7.524009	7.388271	7.731702	7.736188

Supplemental Table 1. Gene expression values changed greater than 2 fold.

Affymetrix ST 1.0 Array ID	0-No	6-No	12-No	0-Yes	6-Yes	12-Yes
7905365_PIP5K1A	6.720689	7.873975	7.612956	7.560603	7.953259	7.493714
7905544_SPRR1A	6.43085	5.3757	5.595567	5.994649	5.833218	5.870211
7905571_S100A9	7.131792	5.839152	7.163359	6.485156	7.294042	7.537636
7905918_EFNA3	5.657512	4.493148	4.932879	5.621579	4.905891	4.659453
7906501_ATP1A2	9.823015	11.01262	10.43841	10.57769	11.01533	10.94016
7906757_FCGR2A	4.766393	3.657707	4.490148	4.205696	4.059308	4.659383
7906852_UHMK1	7.049228	8.151859	8.085075	7.697061	8.368994	8.225247
7907790_CEP350	7.361232	8.443571	7.720811	7.566477	8.579355	7.98707
7908312_PRG4	6.324037	5.066641	5.806762	6.692111	5.183486	5.699412
7908409_RGS2	6.815633	5.578104	6.402673	7.519507	6.286294	6.385618
7908437_CDC73	7.074789	8.106672	7.562782	7.119611	8.138166	7.68771
7908543_NEK7	7.663748	8.920084	8.545651	8.37961	9.054414	8.624672
7908650_DKFZp434B1231	7.949509	5.697185	5.393947	8.434153	5.327543	6.099721
7909285_PFKFB2	5.966134	7.022254	6.855516	6.804313	7.581002	7.459081
7909610_ATF3	6.150059	5.011442	4.857962	7.030092	4.797831	5.057142
7909708_CENPF	4.689474	3.643044	4.050648	4.722671	4.438543	4.684241
7909730_KCNK2	5.794893	4.626433	4.294761	5.180014	4.557068	4.328588
7909992_FBXO28	7.269589	8.42024	8.25089	7.979071	8.541053	8.153375
7910099_SRP9	7.270954	8.29775	8.700847	7.910778	8.483679	8.179251
7910134_MIXL1	7.08057	5.951983	6.155089	6.406289	5.900795	6.381441
7910446_COG2	5.489211	6.578127	6.073348	5.899915	6.275343	6.615429
7910494_ARV1	6.176527	7.187618	7.357352	6.720867	7.418234	7.049861
7910520_GNPAT	8.216242	9.237536	9.128108	8.728464	9.286971	9.33746
7910550_TSNAX	6.62306	7.750168	7.446056	7.278303	8.05946	7.548641
7911271_OR5BF1	6.412159	5.126331	5.481972	5.289832	5.053002	6.138761
7911854_KIAA0495	5.749821	7.170243	6.483469	6.548923	6.584503	6.838635
7911989_RPL22	4.513953	5.745264	5.979668	5.396596	5.513153	6.038169
7912852_EIF1AP1	8.535515	9.668947	9.251089	9.106701	9.996798	9.489832
7912937_PADI2	9.091736	10.10327	9.724176	9.440122	10.0233	10.17273
7913001_UBR4	8.396511	9.427826	8.974443	8.880209	9.266331	9.206156
7913216_PLA2G2A	6.554422	5.288746	5.733143	6.724938	5.690238	6.107346
7913824_C1orf63	5.699743	6.718598	6.391284	6.138628	6.831132	6.450955
7913907_TRIM63	9.444783	8.143925	8.022605	10.17051	8.424899	8.587295
7914194_DNAJC8	5.446329	6.458485	7.394448	6.296825	7.260778	5.721976
7914270_LAPTM5	7.995487	6.838611	8.12381	7.483727	8.066666	8.326672
7914334_WDR57	5.599468	6.726997	6.327133	5.927063	6.383535	6.404986
7915902_CYP4A11	5.327782	4.240215	5.125796	4.519845	4.543338	4.989584
7915995_FAF1	7.774125	8.863217	8.629351	7.961153	8.69525	8.640055
7916185_ZCCHC11	6.815774	7.966434	7.702673	7.053366	8.149158	7.405843
7916316_TMEM48	4.555729	5.87002	5.737312	5.515882	6.066275	5.612991
7916443_USP24	7.218563	8.726732	8.112701	7.925046	8.713532	8.097191
7916592_MYSM1	6.407435	7.652003	6.860335	6.153525	7.762661	6.99821
7916808_SLC35D1	5.78775	7.360125	6.927846	6.02558	7.304158	6.959639
7916910_LRRC40	5.193475	6.21772	6.172382	5.849927	6.50427	5.26552
7916969_ZRANB2	7.028177	8.051065	7.667218	7.256671	8.55508	7.289648

Supplemental Table 1. Gene expression values changed greater than 2 fold.

Affymetrix ST 1.0 Array ID	0-No	6-No	12-No	0-Yes	6-Yes	12-Yes
7917052_SLC44A5	4.994397	6.705709	5.929971	4.568189	6.174399	5.559584
7917103_ZZZ3	6.478559	7.486691	7.251248	6.978029	7.996298	7.116692
7917156_FUBP1	7.453942	8.457616	8.236328	7.669734	8.656336	8.189022
7917182_ELTD1	5.95022	7.285161	6.860318	6.732375	7.737977	6.446063
7917338_BCL10	6.353327	5.308936	5.958723	6.055409	6.061459	5.815225
7917604_ZNF644	5.797105	6.8062	5.803767	5.951231	7.089797	6.258408
7917634_HFM1	3.05177	4.72797	4.144707	3.354531	4.599661	4.420312
7917674_LOC646817	5.640166	7.364303	6.659543	7.29194	6.214132	6.871243
7917875_F3	6.728136	5.60519	6.164267	6.494208	5.956309	6.194727
7917885_CNN3	7.837473	6.636129	7.072771	7.673286	6.905659	7.10385
7918275_WDR47	5.66009	6.714017	6.150603	6.273272	6.776109	6.415729
7918681_RSBN1	5.8333	7.036482	6.86195	6.623199	6.862065	6.703194
7918768_DENND2C	7.343499	9.272776	8.810558	7.574734	9.428287	8.299162
7918847_SIKE	5.960392	6.961527	6.687902	6.125453	7.270464	6.715409
7919028_TBX15	7.810836	8.845461	8.357852	8.08619	8.544907	8.440896
7919193_NUDT4P1	9.043447	10.22394	9.974525	9.789412	10.20014	9.998625
7919598_LOC729135	7.977771	6.757434	7.614509	8.166626	6.840531	6.745648
7919780_GOLPH3L	5.998682	7.289384	7.523585	6.569331	7.67235	7.505148
7920217_SPRR2G	7.428424	6.314856	6.559028	6.85321	6.092404	6.451922
7920244_S100A8	7.059299	5.333441	6.849293	6.809156	7.459221	7.209466
7920271_S100A4	8.226213	7.029446	7.430868	7.748932	6.854006	7.518386
7920401_SLC39A1	6.326639	7.337937	6.946799	7.017888	7.174952	7.38083
7921344_ELL2	6.495969	5.486327	5.899697	7.131314	6.374963	6.102988
7921690_ITLN1	5.735618	3.333037	3.498801	4.553938	3.268131	3.538781
7922130_DPT	9.408064	8.090887	8.403824	8.978993	8.325325	7.91557
7922174_F5	4.976734	3.941191	4.339502	4.86931	4.710709	4.472007
7922219_SELL	6.066992	4.382864	6.104496	5.75419	6.33848	6.3734
7922229_SELE	5.4181	4.115772	4.101108	6.422738	3.961088	3.99727
7922301_MYOC	6.598734	5.572201	6.108788	6.616009	5.710069	6.111521
7922337_TNFSF18	3.862098	2.633387	3.11287	2.947266	2.893591	3.172516
7922382_ANKRD45	4.206589	3.053114	3.413944	3.197436	3.755053	3.57338
7922410_SNORD44	3.65333	4.832745	5.761621	4.458853	5.326138	4.546572
7922646_IFRG15	6.239637	7.293316	7.262277	7.279453	7.74089	7.472694
7922717_RGS16	7.16904	6.10574	6.246448	7.887644	6.074288	6.147824
7922793_ARPC5	8.492075	7.269164	7.066993	6.917423	7.037793	7.341008
7922823_EDEM3	6.168793	7.246652	6.77143	6.943861	7.573845	6.873375
7922912_TPR	5.845604	7.089738	6.573099	6.437571	7.470452	6.046757
7923131_DENND1B	5.127088	6.217109	5.26655	5.825559	6.407216	5.612083
7923662_PIK3C2B	7.352428	8.417028	7.930951	7.270733	8.06023	8.163708
7923824_SLC41A1	7.581366	8.872017	8.481997	8.653718	8.488787	8.701275
7924107_LPGAT1	4.888825	6.467775	5.631765	6.010705	6.457621	5.733325
7924190_ANGEL2	5.491495	6.69608	6.36926	5.854252	6.670052	6.075377
7924351_EPRS	7.306175	8.445157	7.853142	7.631472	8.716325	8.03392
7924582_WDR26	7.011246	8.474017	7.858272	7.46068	8.484728	8.155376
7924733_PARP1	6.762709	8.027592	7.824077	7.227588	7.962647	8.065903

Supplemental Table 1. Gene expression values changed greater than 2 fold.

Affymetrix ST 1.0 Array ID	0-No	6-No	12-No	0-Yes	6-Yes	12-Yes
7924821_gm127	4.064403	2.170289	1.881405	2.047589	2.066068	2.186831
7924923_NUP133	5.462933	6.491109	6.343398	6.019335	6.673923	6.233939
7924977_PGBD5	5.298303	4.262201	4.48838	4.721096	4.238822	4.709409
7925130_TARBP1	6.295164	7.659285	7.179032	6.476052	7.480801	7.168402
7925318_RNU5D	5.543795	4.018827	5.430852	4.837314	4.170686	4.866488
7925413_MT1P2	6.897096	5.43096	6.384799	6.917777	6.153512	6.030943
7925525_CEP170	5.10986	6.63482	4.537164	5.67205	6.527928	5.029363
7925622_AHCTF1	6.097492	7.104242	6.585877	6.359031	7.322691	6.454631
7926889_LYZL1	3.600091	2.515484	2.821513	2.991102	2.607226	3.42289
7927082_HSD17B7P2	4.316813	5.561161	4.890635	5.763695	5.415923	4.838937
7927108_BMS1	5.778536	7.049506	6.984607	6.581257	7.164033	6.88644
7927389_MAPK8	6.138204	7.37911	7.086916	6.908863	7.590135	7.403549
7927536_NCOA4	8.410883	9.477771	9.372342	9.052563	9.631329	9.618219
7927606_PRKG1	6.395963	7.926332	6.940856	6.373055	7.602846	6.391475
7928855_BMPR1A	7.329635	8.41813	8.215093	8.129118	8.808744	8.387865
7929168_TNKS2	8.450926	9.470849	9.302419	8.975413	9.721732	9.26227
7929201_BTAF1	6.303833	7.379881	6.958168	6.703206	7.723103	7.302223
7929288_EXOC6	6.829117	8.243822	7.889975	7.403015	8.451591	7.299603
7929858_C10orf6	5.447515	6.772028	6.368397	6.227252	6.7841	6.444219
7929958_BTRC	5.58582	6.906124	6.949566	6.160963	7.051244	6.845613
7930008_NOLC1	6.309098	7.322058	7.296401	7.139826	7.421044	7.348027
7930614_NHLRC2	6.982253	7.994248	7.7381	7.499	8.326989	7.661799
7930682_KIAA1600	7.131596	8.265631	7.980057	7.542573	8.117787	7.720143
7930996_BRWD2	5.545506	6.648837	6.474128	6.179858	6.662948	6.591754
7931168_ACADSB	5.8293	7.478564	6.974944	6.330594	7.589787	7.312929
7931187_BUB3	6.248101	7.470512	7.314632	6.619675	7.509692	7.343683
7931199_GPR26	5.469147	4.463443	4.611827	5.362543	4.779906	4.564666
7931216_KIAA0157	6.464684	7.591636	7.126347	6.800746	7.557269	7.328141
7931239_C10orf137	6.025633	7.075771	6.396795	5.895802	6.701425	6.243168
7931348_FOXI2	7.933256	6.913684	7.222964	7.475429	6.932417	7.357245
7931683_DIP2C	7.09917	8.490732	8.23436	7.476255	8.057785	8.259709
7931930_PRKCQ	8.451411	10.06261	9.691088	8.921316	9.990833	9.765594
7932000_KIN	5.528401	6.838516	6.616577	5.712165	7.069311	6.383595
7932014_FLJ45983	6.32116	5.107527	5.71888	5.565569	5.516863	5.878724
7932082_CCDC3	6.245486	5.190481	5.431258	6.339293	5.349018	5.582045
7932160_FAM107B	6.968598	5.849704	6.008671	6.852857	5.953978	6.185438
7932584_PRTFDC1	4.914431	3.914063	4.149348	4.202885	4.290505	4.105749
7932678_YME1L1	8.121176	9.511111	9.238324	8.852858	9.73529	9.201176
7932703_ACBD5	5.735771	6.816326	6.297496	6.469386	6.727925	6.260243
7932765_MPP7	6.418778	7.736593	7.313473	6.717615	7.777471	7.369256
7933084_PBEF1	7.628618	8.807714	8.234005	9.283002	9.088581	8.370642
7933105_ZNF25	6.347587	7.495165	7.211531	6.737309	7.704841	7.111027
7933192_HNRPA3P1	8.510757	7.32415	7.104011	6.302529	6.594611	7.076342
7933821_RHOBTB1	8.906891	10.23095	10.05363	9.213324	10.23231	9.769841
7934278_P4HA1	7.426291	8.683338	8.475496	8.101501	9.065012	8.644314

Supplemental Table 1. Gene expression values changed greater than 2 fold.

Affymetrix ST 1.0 Array ID	0-No	6-No	12-No	0-Yes	6-Yes	12-Yes
7934477_CAMK2G	8.089342	9.30025	8.981012	8.298325	9.087111	8.993837
7934513_AP3M1	6.259734	7.529691	7.399075	6.807798	7.875098	7.317777
7934653_POLR3A	6.074419	7.151503	7.063205	6.283716	7.093118	7.245053
7934812_WAPAL	6.340271	7.591949	7.276625	7.365654	7.957161	7.347258
7934906_ACTA2	10.05999	8.688472	8.38601	10.04505	8.901258	9.210277
7934970_HTR7	6.02369	4.701099	5.0865	5.34652	4.84235	5.243355
7934979_ANKRD1	10.04154	6.880675	8.563455	11.09228	8.515297	7.59908
7935270_BLNK	5.999241	4.741586	5.012475	5.095496	4.849327	4.987073
7935462_EXOSC1	6.319663	7.393083	7.222641	6.85513	7.178488	7.269885
7935627_GOT1	9.391324	10.51618	10.35929	9.5512	10.09475	10.356
7935660_DNMBP	6.363698	7.428152	7.180047	6.812031	7.437364	7.369585
7935910_MGEA5	6.631908	7.832595	7.661877	7.237651	8.112237	7.643407
7935930_KCNIP2	6.660831	5.595162	6.574207	6.447344	5.895319	6.043195
7936346_ZDHHC6	5.684397	6.70372	6.706237	5.93799	6.85034	6.47022
7936419_C10orf118	5.50346	6.529963	5.505962	5.462848	6.539266	5.344844
7936835_CPXM2	6.295775	5.117462	5.305709	6.32412	4.953495	5.796651
7937287_PSMD13	7.116261	8.116365	8.057549	7.494477	7.930982	7.993281
7937882_ART1	6.598363	7.641568	7.794053	7.268846	7.777237	7.915422
7937915_RRM1	6.611575	7.83392	7.635237	6.768371	7.846894	7.817499
7938082_CNGA4	5.575114	4.554374	4.587892	5.549037	4.887454	4.920905
7938128_CCDC35	4.437448	5.864219	5.652805	5.131901	5.775476	5.057366
7938231_PPFIBP2	6.554075	7.569458	7.20445	6.663146	7.399459	7.183368
7938422_CTR9	6.839156	7.91909	7.80158	7.99272	8.189868	7.503828
7938448_USP47	6.561507	7.604328	7.026395	7.071151	7.998784	7.072193
7938646_CALCB	6.32653	5.061045	5.496904	6.103659	5.213251	5.589829
7938687_NUCB2	5.824955	6.940904	6.521542	6.573863	7.15299	6.089358
7938890_PRMT3	4.465033	5.882029	5.442248	4.691463	5.147241	5.25071
7938951_TMEM16E	7.432667	8.819654	8.457426	8.136708	8.962114	8.350226
7939341_CD44	8.734995	7.734219	8.284981	8.990583	8.244493	8.462143
7939368_TRIM44	6.196914	7.258756	7.168052	6.922303	7.18548	6.767885
7939424_API5	5.946821	7.255363	7.008331	6.601284	7.859115	7.134348
7939595_CRY2	8.024079	9.096125	8.659237	8.311354	8.816998	9.049553
7939676_KIAA0652	6.339572	7.457302	7.285256	6.744117	7.359168	7.573608
7940135_GLYATL1	5.636578	4.570923	4.543472	5.319007	4.356184	4.603097
7941478_SART1	5.804991	7.077762	6.82069	6.634764	6.906568	7.30293
7941702_RBM14	5.706565	6.831916	6.844974	5.43913	6.965766	6.579839
7941707_TMEM137	5.509568	6.76488	6.694849	5.300335	7.024874	6.525361
7942255_KRTAP5-7	9.384828	8.007571	8.707135	8.610138	8.357739	8.59015
7942284_DEFB108B	5.177466	3.841081	3.939958	3.8974	4.016368	3.934324
7942476_PAAF1	4.523174	6.396067	6.538217	5.659863	6.12923	5.687025
7942914_EED	5.965971	7.043611	7.043188	6.249831	7.058435	6.766214
7943580_CUL5	6.429287	8.011423	7.016457	7.024047	8.429155	7.199132
7943690_DDX10	4.07438	5.458169	5.658148	5.223794	5.997416	5.848737
7943867_BCDO2	4.562122	5.936123	5.63127	4.940023	5.96311	5.602447
7943984_ZBTB16	8.883875	9.902096	9.328176	9.76802	10.04768	9.994171

Supplemental Table 1. Gene expression values changed greater than 2 fold.

Affymetrix ST 1.0 Array ID	0-No	6-No	12-No	0-Yes	6-Yes	12-Yes
7944006_RBM7	4.470741	5.684896	5.676188	5.049571	5.682742	5.355659
7944082_TAGLN	8.867623	7.343748	6.944646	8.431709	7.213915	7.799849
7944152_IL10RA	6.496221	5.458008	6.337466	6.011842	5.589516	5.786373
7945283_ACAD8	6.404446	7.484582	7.331846	6.632231	7.556128	7.558772
7946015_OR51A4	5.261591	4.164721	4.425677	4.575928	4.312773	4.341267
7946680_BTBD10	5.984497	7.036389	6.989353	6.854509	6.930604	7.369242
7946703_COPB1	6.402876	7.625017	7.387445	7.359604	8.091261	7.343762
7946757_SOX6	6.615962	7.86642	7.491667	6.953649	8.222624	7.566281
7946957_SAAL1	4.75157	5.838504	5.516755	4.925906	5.68035	5.124696
7947199_LGR4	5.866853	7.037198	6.451834	6.534831	7.058878	7.033472
7947274_MPPED2	5.622757	6.922632	6.537446	6.379322	6.969501	6.811179
7947540_TRAF6	6.775719	7.802624	7.429347	7.030092	7.807103	7.57304
7948900_SNORD30	5.250289	6.280297	6.313286	5.576506	6.437998	5.956008
7948902_SNORD29	5.424897	7.207188	7.771396	5.733396	6.846253	6.581488
7948904_SNORD28	8.993412	10.43047	10.47924	8.669391	9.941541	9.987778
7948906_SNORD27	3.115833	4.399708	3.984803	3.477305	3.622722	4.508466
7948910_SNORD25	2.377039	4.473607	2.849568	3.189246	3.781446	4.432712
7948937_SLC22A8	5.919475	4.781873	5.164365	5.334146	5.344793	5.234889
7949754_CLCF1	7.809997	6.592528	7.241943	7.363725	6.928617	7.018634
7950036_FGF3	6.784634	5.658772	6.213163	6.808062	6.319212	6.361411
7950447_XRRA1	5.817733	6.911978	6.587605	5.919652	6.770237	6.836244
7950578_PAK1	8.294887	9.577038	9.390524	8.36146	9.326305	9.429348
7951036_SNORD5	5.256818	6.875929	6.559634	6.258263	7.409386	6.333004
7951038_SNORA40	3.83975	5.046115	4.725283	4.291674	4.746634	4.696733
7951554_RDX	7.029065	8.277302	7.876453	8.109705	8.689704	7.754053
7952382_OR6T1	5.493036	4.381796	4.241134	4.490776	4.304293	4.357386
7952673_FLJ45950	4.099259	3.019678	3.503995	3.621249	3.334599	3.576492
7952785_OPCML	4.996345	3.918137	4.321175	4.369952	4.049076	4.256368
7953094_CACNA1C	5.496572	4.187675	4.421006	4.871652	4.805077	4.653995
7954055_APOLD1	7.725192	6.120894	6.260097	7.977384	6.266138	6.575664
7954090_EMP1	8.039775	6.908692	7.437132	9.176338	7.00295	7.030322
7954196_MGST1	6.097776	4.434164	5.720728	5.734787	4.520525	5.059388
7954344_LST-3TM12	3.378104	2.322484	3.052196	3.040582	2.663649	2.748761
7954419_ETNK1	5.291352	6.396302	6.174642	5.674673	6.614998	6.036669
7954481_SSPN	8.024391	9.341201	9.015967	8.243484	9.266547	8.749661
7955195_TROAP	6.668996	5.623292	6.73528	6.595302	6.240203	6.299743
7955589_NR4A1	9.449126	7.551409	7.632787	9.822363	7.454026	7.776206
7955694_IGFBP6	8.916887	7.864858	8.048764	8.47302	8.08292	8.333384
7955858_HOXC10	6.880888	8.936766	8.958694	6.700663	8.572292	8.859509
7955991_OR6C3	4.363152	3.285076	3.410406	3.548612	3.438285	3.331712
7956242_COQ10A	8.810081	9.89503	9.992277	8.737965	9.884452	10.0484
7956648_XRCC6BP1	5.359934	6.479819	6.565708	5.642349	5.996802	5.967306
7956670_USP15	6.681127	7.851945	7.789001	7.386151	8.388375	7.364673
7957023_LYZ	6.47368	5.155236	6.849636	6.121959	7.168526	7.163974
7957140_LGR5	8.281805	9.36385	9.217535	8.566196	9.405725	9.400226

Supplemental Table 1. Gene expression values changed greater than 2 fold.

Affymetrix ST 1.0 Array ID	0-No	6-No	12-No	0-Yes	6-Yes	12-Yes
7957167_TMEM19	6.655021	7.769046	7.665725	7.143153	8.067914	7.624547
7957514_FLJ46688	3.963487	2.940927	2.88624	3.053044	2.935263	3.35998
7957540_MRPL42	5.553662	6.917414	6.463756	6.000208	7.304592	6.628301
7958147_TDG	6.054002	4.939977	5.025285	4.932997	5.19488	5.041258
7958216_KIAA1033	5.698091	6.968565	6.791705	6.276196	7.450041	6.373036
7959623_GTF2H3	5.636232	6.64364	6.451686	5.60692	6.402944	6.128086
7960221_JARID1A	6.660345	7.66463	7.095278	6.636802	7.705543	7.045237
7960730_MBOAT5	7.767424	9.33556	8.669312	8.570092	9.107569	9.120054
7960865_SLC2A3	8.558565	7.155959	7.138912	9.925293	7.315548	7.409976
7960933_M6PR	7.468973	8.552448	8.62111	7.810685	8.688821	8.649019
7961208_FLJ10292	4.78331	6.498605	6.109534	5.653383	5.99406	5.812165
7961243_TAS2R8	3.685275	2.597612	2.425645	2.417956	2.473766	2.564529
7961275_TAS2R13	3.358253	4.790191	3.790367	3.618433	4.185231	3.480561
7961291_TAS2R44	4.547587	5.599552	5.298092	5.092071	5.801285	5.304895
7961339_LRP6	6.940216	8.072444	7.737458	7.75313	8.228834	7.876827
7961514_MGP	8.421636	6.785046	6.981288	8.3666	7.054726	6.916861
7961710_ABCC9	7.834933	9.091743	8.858164	8.334843	9.345599	8.890047
7961865_KRAS	5.339935	6.355408	5.972382	6.23329	6.785291	6.388741
7962058_TMTC1	6.831606	8.273609	7.432775	7.464421	8.147808	7.989196
7962085_IPO8	5.410478	6.860222	6.640103	6.254288	7.285598	6.506641
7962139_OVOS2	5.382689	4.217415	4.487341	4.715242	5.274678	3.80547
7962151_MGC24039	5.736344	7.048231	6.993609	6.425771	6.774439	6.405624
7962427_PUS7L	4.477508	5.500532	4.834427	4.806531	5.668226	4.636638
7962783_ASB8	9.105448	10.34517	10.14871	9.308296	10.39942	10.3319
7962884_RND1	6.119822	5.036358	5.082441	6.637861	5.078963	5.152263
7963235_FAM130A1	5.863656	7.017344	7.11234	6.862297	7.097569	6.857189
7963459_KRT72	6.263217	5.2428	5.840202	5.957596	5.608885	5.637856
7963646_AAAS	7.225586	8.267645	8.211182	7.629678	8.133509	8.211498
7963750_CBX5	6.400119	7.477901	7.376051	6.949025	7.83438	7.40912
7963774_ZNF385	8.136925	7.081254	7.198051	8.142369	6.823036	7.507892
7964246_SNORD59B	4.904507	7.402366	6.834342	5.578261	7.184622	7.083579
7964248_SNORD59A	4.142777	5.636344	5.272453	3.884358	5.408653	5.227264
7964555_AVIL	6.417888	7.483203	7.245014	5.881768	6.968646	6.626981
7964852_BEST3	5.65123	6.706385	6.002404	6.191661	6.793487	6.372588
7964927_TSPAN8	5.448067	7.252806	7.658467	5.376956	7.15762	7.176741
7965022_KRR1	5.934102	7.225543	6.842498	6.445162	7.759602	6.094734
7965036_LOC390345	3.828344	5.398029	4.340328	5.541739	3.970392	4.259015
7965040_PHLDA1	7.055039	5.613521	5.801248	7.598489	5.94644	6.227485
7965343_WDR51B	5.08628	6.121791	6.346327	5.641911	6.392546	6.069661
7965873_IGF1	4.987069	6.173654	6.51981	5.745476	6.128358	5.829129
7965956_NFYB	5.172542	6.281389	5.980572	5.538249	6.501982	6.007563
7965964_SLC41A2	5.009968	3.929342	4.608198	4.82773	4.61002	4.732024
7966098_SART3	6.081388	7.319409	7.080039	6.558871	7.077433	7.217814
7966345_PPTC7	8.725797	9.770384	9.364488	9.271078	9.731506	9.517562
7966462_C12orf30	6.519786	7.633011	7.590121	6.808769	8.053102	7.216999

Supplemental Table 1. Gene expression values changed greater than 2 fold.

Affymetrix ST 1.0 Array ID	0-No	6-No	12-No	0-Yes	6-Yes	12-Yes
7966779_NOS1	6.597936	7.732068	7.061758	7.078367	7.061055	7.775905
7966938_GCN1L1	7.299224	8.381123	8.117994	7.925283	8.266176	8.438551
7967034_PLA2G1B	5.675995	4.665778	4.903146	5.033041	4.750628	4.794576
7967210_LOC338799	5.723403	6.746174	6.343219	5.965175	6.355631	6.880146
7967420_SBNO1	5.794516	7.184895	6.546443	6.443549	7.421419	6.55203
7968029_PCOTH	4.896974	3.839198	4.002916	4.520694	3.894672	4.056328
7968265_PDX1	6.739718	5.405687	5.918863	6.138239	5.621932	6.036872
7968274_PAN3	6.21549	7.235641	7.120636	6.862308	7.560554	7.197128
7968333_USPL1	5.570078	6.992312	5.887183	6.230898	6.92394	6.075557
7968417_FRY	6.735267	7.99498	7.510176	7.311023	8.065331	7.805065
7968577_NBEA	4.729606	6.325797	5.713347	5.200394	6.320286	5.864945
7968658_EXOSC8	5.059279	6.597675	6.315135	5.549018	6.820366	6.344152
7968678_FREM2	5.771898	7.119233	6.42892	5.876694	7.056285	6.527089
7968711_COG6	6.062191	7.401433	7.043769	6.674949	7.652014	6.999867
7968761_NARG1L	5.69183	6.880849	6.318027	5.431666	6.923592	6.22429
7969228_ALG11	5.588706	6.848796	6.319231	6.073044	6.977848	6.107809
7969438_LMO7	6.701544	7.789031	7.378103	6.849098	7.989608	7.071064
7969640_CLDN10	4.382684	3.19299	3.548167	3.747845	3.812832	4.171161
7970084_FLJ10769	6.131978	7.206497	6.885475	6.668318	7.220768	7.245433
7970262_CUL4A	7.547009	8.690262	8.564706	8.146703	8.81376	8.720233
7970390_LOC646442	4.747457	3.509342	3.617012	3.681694	2.999748	3.184413
7970413_PSPC1	6.419052	7.481643	7.384428	6.398399	7.724641	7.372821
7970473_XPO4	8.26455	9.496952	9.172211	9.263167	9.914754	8.997782
7970716_LNX2	6.569933	7.58488	7.367975	7.081789	7.78382	7.599452
7970949_MAB21L1	5.489309	7.011252	6.025339	5.318387	6.254269	6.393887
7971015_SMAD9	7.313359	6.264742	6.273356	7.075328	6.216509	6.048706
7971039_FAM48A	6.508678	7.576325	7.185455	6.49483	7.567503	7.282061
7971077_POSTN	5.498773	3.520882	3.556915	4.551127	3.302715	3.677628
7971218_KBTBD7	5.144623	6.497226	6.351595	5.755753	6.382465	6.163141
7971246_RP11-125A7.3	7.954955	9.222939	8.774076	8.374631	9.240779	8.711739
7971388_SLC25A30	7.013086	8.24252	8.088476	7.915873	8.437658	7.991664
7971541_SUCLA2	8.502356	9.50612	9.502485	9.08336	9.795846	9.229658
7971653_DLEU2	5.351854	6.803867	6.876899	4.535005	6.757162	6.079217
7971967_DIS3	5.117945	6.359574	5.784288	5.648583	6.36887	5.69662
7971998_LOC387934	8.892447	7.846696	7.731297	8.510266	7.918783	8.299938
7972003_KLF12	6.562792	7.590814	7.048069	6.990554	7.381346	7.059621
7972190_RBM26	6.461434	7.536587	7.295637	6.8379	8.055744	7.248146
7972867_PCID2	5.527327	6.60195	7.003847	6.06971	6.794125	6.798627
7972888_PCID2	5.422059	6.426019	6.516535	5.836734	6.437489	6.395649
7973018_OR4K15	4.462031	3.176115	3.419706	3.69408	2.702365	3.554748
7973098_FAM12B	4.039186	3.027809	3.076738	3.292309	3.169424	3.116779
7973101_RNASE6	5.765183	4.593422	5.414215	5.061409	4.661626	5.263477
7973214_TOX4	7.430901	8.500625	8.264494	7.853893	8.403193	8.323716
7973580_LOC161247	9.515796	10.57342	10.62114	9.817879	10.69159	10.72274
7973826_NUBPL	4.916039	6.574765	6.163661	5.427159	6.552551	6.43069

Supplemental Table 1. Gene expression values changed greater than 2 fold.

Affymetrix ST 1.0 Array ID	0-No	6-No	12-No	0-Yes	6-Yes	12-Yes
7973850_AKAP6	7.913624	9.59721	8.711749	8.453056	9.55558	8.548788
7974090_CTAGE5	6.658442	7.784111	7.12683	6.839192	7.81641	6.956395
7974207_MGAT2	5.555884	6.601359	5.890294	5.825888	5.8468	6.187972
7974214_KLHDC1	5.735391	6.807591	6.621864	5.938799	6.870379	6.346104
7974387_STYX	6.931818	8.05792	7.889472	7.610474	8.352086	7.852168
7974473_FBXO34	7.743048	8.919518	8.582281	8.374956	9.010432	8.741149
7974725_C14orf100	6.558983	7.597815	7.559527	7.2332	7.946392	7.575287
7974771_C14orf135	6.022868	7.173074	6.44969	6.230333	7.401852	6.62685
7975045_MTHFD1	7.360154	8.441478	8.145391	7.831725	8.189781	8.233896
7975068_ZBTB1	5.465344	7.016655	6.543499	6.002326	7.19306	6.03197
7975167_GPHN	6.607666	7.662087	7.194535	6.516463	7.430566	7.12606
7975203_MPP5	5.257129	6.620358	6.16209	6.039177	6.865361	6.04274
7975224_EIF2S1	6.928309	7.964279	7.513978	7.59016	8.47273	7.893757
7975344_SLC39A9	5.586933	6.901406	6.478811	6.289346	6.856567	6.845432
7975416_PCNX	7.285675	8.464334	8.130766	7.889189	8.598889	8.152982
7975521_RBM25	6.622028	7.69325	7.476995	7.082442	8.170958	7.13226
7975632_ZADH1	6.193093	7.402138	7.014849	6.591737	7.389489	6.726654
7975779_FOS	10.57765	7.430259	6.48291	11.66186	6.538942	6.597856
7975863_C14orf118	6.376912	7.987737	7.619264	6.895096	8.16627	7.808733
7975926_KIAA1737	8.443223	9.678195	9.352191	8.510129	9.689687	9.348637
7976000_ADCK1	5.342738	6.380428	5.986982	6.293692	6.349683	5.96852
7976598_PAPOLA	6.950075	8.259896	7.881936	7.513622	8.492677	7.860492
7976783_DLK1	8.695677	7.34766	7.517384	8.720717	7.747356	7.572537
7976816_SNORD114-3	5.313947	4.222312	4.460029	4.434792	3.88451	4.096412
7977058_EIF5	7.808366	8.93576	8.519386	8.280414	9.095202	8.537617
7977674_SUPT16H	6.670742	7.71645	7.197276	7.23898	7.943741	7.522952
7977732_SNORD8	6.701683	7.903078	8.098154	6.820168	6.692726	6.410833
7978449_HECTD1	8.069723	9.285997	8.757417	8.699004	9.40532	8.855536
7978718_SEC23A	7.258608	8.435619	8.343794	8.052366	8.566441	8.259127
7978760_KLHL28	6.691598	7.782438	7.829757	7.767167	8.317565	7.574347
7978866_SDCCAG1	5.931714	7.237086	6.600245	6.431616	7.685472	6.482382
7978932_SOS2	7.809685	9.125398	8.829565	8.226318	9.442496	8.603673
7979367_EXOC5	5.261713	7.016685	6.12567	6.51944	7.27528	6.496926
7979510_SIX4	7.724225	8.781199	7.977987	7.795402	8.614059	8.522747
7979615_SPTB	7.419456	8.49659	8.075581	8.026445	8.620378	8.466902
7979698_ATP6V1D	5.926424	6.929158	6.865646	6.195696	6.915819	6.870818
7979757_ZFYVE26	6.163427	7.300344	6.849067	6.574878	7.088573	7.203506
7979916_SYNJ2BP	7.048641	8.193443	7.788196	7.543999	8.092673	7.392218
7980547_SEL1L	6.821537	7.822017	7.250279	7.438023	7.944654	7.611652
7980891_TC2N	5.703985	6.761152	6.697585	6.613022	7.092431	6.394365
7980923_TRIP11	5.621151	6.920033	5.929098	6.031148	7.531641	6.109618
7981111_DICER1	7.081975	8.560495	8.184135	7.70401	8.764952	7.965411
7981192_ATG2B	6.309099	7.675885	7.369429	6.672482	7.78665	7.15515
7981947_SNRPN // SNORD109B	4.51234	5.676344	6.015477	4.457691	5.846297	5.140306
7981958_SNORD116-7	7.324945	8.451006	8.960497	7.308559	8.208894	8.027693

Supplemental Table 1. Gene expression values changed greater than 2 fold.

Affymetrix ST 1.0 Array ID	0-No	6-No	12-No	0-Yes	6-Yes	12-Yes
7981962_SNORD116-5	7.324945	8.451006	8.960497	7.308559	8.208894	8.027693
7981972_SNORD116-12	3.175331	4.947911	4.003371	3.777763	4.502797	4.290521
7981990_PWCR1	7.560956	10.09714	9.457023	8.297421	10.0244	10.00622
7981994_SNORD116-23	4.419128	5.865212	5.857364	4.662179	5.300843	5.385629
7981998_SNORD116-25	4.616308	5.631879	5.507012	4.339075	5.524244	5.039993
7982008_SNORD115-13	6.227508	8.221359	8.913842	6.973832	7.520998	7.457349
7982016_SNORD115-5	6.737932	8.331493	9.284879	7.020904	8.361834	7.845249
7982018_SNORD115-6	6.842948	8.608117	9.14786	7.255433	8.288251	8.112522
7982020_SNORD115-7	3.424789	5.185725	6.429635	3.542834	5.350282	4.105939
7982024_SNORD115-12	6.737932	8.331493	9.284879	7.020904	8.361834	7.845249
7982028_SNORD115-43	6.873795	8.621606	9.326948	7.069919	8.375408	7.939776
7982030_SNORD115-5	6.737932	8.331493	9.284879	7.020904	8.361834	7.845249
7982032_SNORD115-16	6.227508	8.221359	8.913842	6.973832	7.520998	7.457349
7982036_SNORD115-15	3.641683	2.336758	2.416313	3.198722	2.596413	3.105982
7982038_SNORD115-1	6.227508	8.221359	8.913842	6.973832	7.520998	7.457349
7982046_SNORD115-20	4.859962	6.130544	7.420487	4.39804	5.780389	5.337734
7982050_SNORD115-12	6.590799	8.211433	9.127142	6.533457	7.990268	7.631471
7982052_SNORD115-23	4.80685	6.55149	7.845061	4.94208	6.645257	5.704524
7982056_SNORD115-25	5.203169	6.588611	7.973662	6.177649	6.490047	6.190371
7982058_SNORD115-26	6.885887	8.678682	9.350237	7.080369	8.397387	7.980684
7982064_SNORD115-43	6.873795	8.621606	9.326948	7.069919	8.375408	7.939776
7982066_SNORD115-6	3.221999	4.284264	4.478868	3.524369	3.874911	4.79637
7982072_SNORD115-33	3.461617	4.812021	4.785821	3.307405	4.001802	3.987327
7982078_SNORD115-11	6.873795	8.621606	9.326948	7.069919	8.375408	7.939776
7982084_SNORD115-12	8.160503	9.866423	10.19704	8.370279	9.339622	9.109344
7982090_SNORD115-6	6.987512	8.683914	9.264561	7.298702	8.348167	8.238748
7982092_SNORD115-9	6.873795	8.621606	9.326948	7.069919	8.375408	7.939776
7982094_SNORD115-44	4.851773	7.28913	7.609309	4.9387	6.004126	6.570806
7982098_SNRPN // SNORD109A	4.51234	5.676344	6.015477	4.457691	5.846297	5.140306
7982154_LOC283755	7.711431	8.868116	8.653095	8.219402	9.088242	8.754229
7982392_RYR3	6.849535	7.956578	7.648678	7.544686	7.749746	7.538987
7982597_THBS1	7.204099	5.091434	5.93718	8.742728	5.727201	5.614112
7982868_CHAC1	7.084189	5.849288	6.046004	6.777822	5.528991	6.052958
7983335_CTDSPL2	6.387259	7.482919	7.039899	6.811018	7.800317	6.950962
7983527_SEMA6D	6.259961	8.013513	7.46137	6.788976	7.954804	7.305271
7983650_SLC27A2	4.755197	3.732539	3.687527	4.580329	3.826108	4.022776
7983663_USP8	4.91449	6.179205	5.655586	5.292537	6.110963	5.110401
7983763_MAPK6	5.55599	6.606092	5.821989	5.717339	6.786687	5.861782
7983779_UNC13C	5.180216	6.42917	4.960587	5.733309	5.774908	5.074489
7983910_AQP9	5.756472	4.387712	5.765105	5.955864	6.024629	6.29668
7984257_RNU5C	5.598293	6.908949	6.345459	5.611399	6.107598	5.588367
7984704_NEO1	7.078072	8.37323	8.253353	7.803445	8.605838	8.22995
7985032_LOC391532	4.152253	2.521715	3.596854	3.755812	3.392321	3.184141
7985119_HMG20A	6.600867	7.96478	7.594084	7.111344	7.941904	7.778893
7985134_IDH3A	7.883264	9.14045	9.080046	8.629501	9.192154	8.830404

Supplemental Table 1. Gene expression values changed greater than 2 fold.

Affymetrix ST 1.0 Array ID	0-No	6-No	12-No	0-Yes	6-Yes	12-Yes
7985166_IREB2	6.240996	7.604453	6.978313	7.010103	8.083308	7.261808
7985493_TM6SF1	6.985487	8.241129	8.231585	7.519082	8.301531	8.10331
7986385_DMN	9.097154	10.16109	9.566978	9.811449	10.06132	9.882142
7986569_LOC283755	8.067093	9.238362	8.976819	8.568641	9.402379	9.134899
7986701_HERC2P2	7.917196	9.122541	8.807771	8.470856	9.276455	9.045446
7986755_MAGEL2	6.493975	4.893207	5.304336	5.353848	4.858669	4.912381
7987192_SLC12A6	6.293642	7.389579	7.012082	6.818395	7.329552	7.198072
7987325_AQR	5.723678	6.950915	6.62739	6.385786	7.358883	6.642734
7987385_MEIS2	5.928487	4.772517	5.351493	6.207692	5.064224	5.447621
7987840_VPS39	7.03584	8.158874	7.71753	7.562329	8.097175	8.146554
7988605_COPS2	7.301822	8.507669	8.047618	8.336338	9.178962	7.69763
7989013_C15orf15	6.402182	7.41525	7.257342	7.049652	7.574589	7.172071
7989069_PYGO1	6.310332	7.885092	7.517199	6.845091	8.133836	7.711082
7989224_ADAM10	6.675467	7.74777	7.360277	7.483615	7.892399	7.502199
7989253_SLTM	6.520682	7.583914	7.19216	6.756005	7.943995	7.018928
7989387_VPS13C	6.371605	8.038184	7.289301	6.936008	8.188631	7.084823
7989516_HERC1	7.765437	8.804976	8.5203	8.145855	8.968182	8.482639
7989806_DPP8	6.293427	7.808478	7.48872	7.334926	8.17049	7.805589
7989937_LCTL	5.067819	3.722734	4.263781	4.422825	4.375585	4.628237
7990054_UACA	7.803162	9.086407	8.384631	8.103522	9.351433	8.120877
7990879_EFTUD1	3.667874	4.722384	4.890718	4.399141	4.638193	4.742443
7991357_AP3S2	7.218989	8.310281	8.255864	7.52896	8.526538	8.454184
7991598_SNRPA1	4.493773	5.550381	5.599261	4.697084	5.448271	5.133947
7991640_TARSL2	6.639365	7.65738	7.702647	7.309888	7.60197	7.695898
7992973_DNAJA3	6.968384	8.010855	7.826274	7.596728	7.970135	8.050112
7993185_NUBP1	7.179554	8.256622	8.325664	7.689623	8.400172	8.511273
7993274_RUNDC2A	5.259144	6.603547	5.786715	6.201289	6.388264	6.114323
7993298_ERCC4	4.454451	5.965493	6.047838	5.299079	6.229619	5.681031
7993807_TMEM159	8.078325	9.247772	8.978682	8.309193	8.991655	9.173955
7993999_LOC595101	8.796907	9.947578	9.683175	9.457724	9.991631	9.64847
7994102_DCTN5	6.415819	7.538289	7.505569	6.927855	7.573433	7.444868
7994572_RUNDC2C	5.607218	4.475885	5.600057	5.082418	5.550523	5.553273
7995040_BCKDK	7.291671	8.306354	8.21248	7.5962	7.98733	8.304163
7995362_GPT2	7.812478	9.109612	8.82351	8.213055	8.908861	9.32863
7995382_PHKB	8.11807	9.124011	8.832225	8.290735	9.253351	8.678106
7995783_MT2A	11.14036	9.925705	10.31396	11.70416	10.17318	10.1877
7995787_MT1M	5.978323	4.848859	5.551747	7.50469	5.013453	5.263241
7995797_MT1E	8.354473	7.176481	7.866343	8.529554	7.028539	7.735909
7995803_MT1JP	6.423655	5.387477	5.639471	6.873962	5.53973	6.084567
7995820_MT1B	7.53267	6.37919	6.58363	6.921707	6.611999	6.840165
7996891_CIRH1A	7.288742	8.315379	8.161737	7.993927	8.16612	8.517843
7997025_PDPR	6.672795	8.651706	8.287353	7.550328	8.404697	8.345158
7997192_HPR	5.134195	3.986496	4.517295	4.033701	3.710308	3.695733
7997504_CDH13	8.199394	9.213752	8.708538	8.538912	8.816585	8.944429
7998722_SNORD60	6.690416	8.271506	8.415817	6.661349	7.555954	7.441556

Supplemental Table 1. Gene expression values changed greater than 2 fold.

Affymetrix ST 1.0 Array ID	0-No	6-No	12-No	0-Yes	6-Yes	12-Yes
7999093_SRL	8.659077	10.12178	9.590651	9.38308	9.841619	9.902458
7999423_SOCS1	7.365277	6.365047	6.897037	7.396024	6.560286	7.002117
7999841_SMG1	7.377321	9.043295	8.541747	8.149724	9.303326	8.489798
7999909_GPRC5B	9.392569	10.3966	10.02644	10.12237	10.33312	10.35433
8000167_SLC7A5P1	9.257591	10.27834	9.999461	9.694777	10.36836	10.03387
8000192_LOC730092	6.910297	8.111471	7.763426	7.328786	8.177502	7.809082
8000222_DKFZp547E087	8.796907	9.947578	9.683175	9.457724	9.991631	9.64847
8000687_LOC440354	8.419394	10.1454	9.959718	9.792502	10.20316	9.78537
8001149_VPS35	6.499281	7.536713	7.317121	7.344039	7.500046	7.125318
8001658_C16orf80	5.754499	6.95351	7.27175	6.286336	6.750374	6.693974
8001748_SNORA50	6.700175	7.849204	8.039983	6.700806	7.615144	7.299942
8001818_TK2	6.338817	7.470169	7.065893	6.936267	7.242442	7.276068
8003635_TUSC5	6.374988	5.34666	6.420922	6.699403	5.985199	5.967027
8003814_ASPA	4.423386	6.053864	5.710095	4.618715	5.720396	5.65793
8004086_USP6	5.515454	6.523645	6.071888	5.146119	6.71472	5.910402
8004510_CD68	8.35681	7.084236	7.593764	8.233806	7.507165	7.666256
8004905_USP43	5.999183	4.972907	5.146793	5.748484	5.085036	5.43952
8005225_LOC162632	5.720523	7.046516	5.854419	5.512035	6.881706	5.993348
8005603_SLC47A1	6.688583	8.073594	7.646477	6.867723	7.738812	7.752247
8005689_LOC220594	4.887909	6.071057	5.876252	5.472519	6.376763	5.838735
8005951_SNORD42B	3.565483	4.726322	4.943255	4.58505	4.538857	4.776594
8006170_LRRC37B2	5.744456	6.94156	6.57516	5.786478	7.026635	6.384038
8006345_RHOT1	7.19576	8.198502	8.185233	7.630177	8.57045	8.017447
8006433_CCL2	8.090573	5.753876	5.997232	9.892403	6.032413	6.673797
8006509_UNC45B	7.653521	8.776016	8.576044	7.771036	8.431292	8.984682
8006594_CCL18	6.055904	5.042464	4.813446	5.629448	4.823668	5.012527
8007302_TUBG1	7.55556	8.729509	8.669348	8.057404	8.595145	8.713403
8007992_KPNB1	7.549962	8.681219	8.604633	8.25679	8.638978	8.221544
8008113_CALCOCO2	7.932325	9.067933	8.669006	8.442888	8.998064	8.81111
8008263_PDK2	9.702329	10.78612	10.37655	9.72969	10.41352	10.57854
8008493_CROP	7.127501	8.175114	7.651681	7.168355	8.501	7.542933
8008540_FLJ42842	5.772655	4.485407	4.453759	4.815627	4.946381	4.775295
8008664_AKAP1	7.981293	9.214104	8.984337	8.550756	8.876668	9.136745
8008795_C17orf71	4.828935	5.899799	5.221764	4.839854	5.461836	5.484149
8009301_PRKCA	5.264237	6.478047	6.173228	5.565277	6.307843	6.12415
8009343_CACNG1	7.556518	8.652667	8.577797	7.296059	8.601245	8.708272
8009476_MAP2K6	6.977916	8.196355	7.748158	7.642143	8.191634	8.099048
8009502_KCNJ2	7.523865	8.687671	8.440494	7.820426	8.839031	8.419513
8009685_SLC9A3R1	8.26914	7.116876	7.949925	8.242977	7.627664	7.717839
8009951_ITGB4	6.685945	5.50676	6.03368	6.070224	5.827281	6.103102
8010184_9-Sep	6.615193	5.215899	4.879224	6.627812	5.529774	5.002107
8010614_FSCN2	6.973382	5.644114	6.156911	6.534693	6.039447	6.585034
8011245_TSR1	6.314096	7.431059	6.964174	6.797646	7.046362	7.093005
8011415_P2RX5	5.98095	7.017712	6.649821	6.642416	6.728076	6.555532
8011823_ZNF594	4.635202	5.744463	4.926837	3.900357	5.455372	5.284664

Supplemental Table 1. Gene expression values changed greater than 2 fold.

Affymetrix ST 1.0 Array ID	0-No	6-No	12-No	0-Yes	6-Yes	12-Yes
8012466_KRBA2	5.163751	6.330189	5.862883	5.694758	5.702297	5.663682
8012949_FBXW10	3.211884	5.521269	5.27685	4.130258	3.989397	5.691766
8013159_TOM1L2	8.154251	9.209115	8.803733	8.551154	9.143461	9.244507
8013179_ATPAF2	6.294757	7.374553	7.074933	6.663646	6.997	7.142905
8013272_CCDC144C	5.280826	6.335307	5.769302	5.171264	6.61957	5.173728
8013431_AKAP10	5.642874	6.769859	5.99282	5.656436	6.76721	6.325866
8013567_LOC201229	3.603835	4.773942	5.168099	4.230052	4.282166	5.391495
8014100_C17orf75	5.774077	6.913505	5.977755	5.933717	6.977577	6.920405
8014574_DDX52	5.130112	6.426229	6.253617	5.420112	6.761448	6.179259
8016285_ARL17	6.403239	7.730775	7.393723	6.869364	7.678817	7.415296
8016300_ARL17P1	6.102335	7.392986	7.069839	6.65417	7.364916	7.130486
8016476_HOXB9	6.542073	5.375922	6.421338	6.389879	6.11764	6.229599
8016562_SPOP	7.457162	8.479856	8.046143	7.748907	8.680914	8.463014
8016646_COL1A1	9.659952	7.83478	8.017358	9.249361	8.093105	8.837736
8016806_LOC388401	5.303204	4.011613	4.82434	4.36771	5.00739	4.833532
8016858_COIL	6.960186	8.024923	7.70947	7.432282	7.858983	7.888649
8017133_FAM33A	4.811843	5.849753	6.069588	5.219915	6.216478	5.54773
8017521_SCN4A	8.374663	10.00743	9.06273	8.80832	9.561268	9.470993
8017776_HELZ	7.205755	8.297948	7.807843	7.593405	8.392673	7.846717
8017927_ABCA9	6.323241	7.341015	6.235921	6.578165	6.920684	6.441907
8017964_ABCA6	6.003899	7.139393	6.884711	6.360341	7.488166	6.692997
8018038_ABCA5	7.437246	8.867956	8.558906	7.785386	9.299903	8.264122
8018652_RNF157	8.231567	9.252164	9.295876	8.295543	8.968086	9.218641
8018694_PRPSAP1	5.760538	6.856174	6.636733	5.96904	6.559267	6.723954
8018774_ST6GALNAC1	6.344422	5.279576	5.710016	5.92775	5.766925	5.994818
8018864_SOCS3	7.390345	5.788718	6.00757	9.394246	5.991076	6.031529
8019751_FLJ44838	4.68816	3.3121	3.519145	3.03574	2.704187	3.52436
8020068_ANKRD12	6.06398	7.13848	6.439643	6.439692	7.422382	6.392536
8020495_CABLES1	6.555467	5.539186	5.929005	6.181655	5.839379	5.876185
8020717_C18orf16	4.730324	3.390762	3.717109	3.932611	3.654122	3.847904
8020847_DTNA	7.223598	8.756746	8.446871	7.741168	8.677773	8.444525
8021113_C18orf25	6.386891	7.45376	7.067573	6.912151	7.806269	7.382002
8021653_SERPINB8	5.83204	3.908387	4.448271	5.478779	4.663085	5.071868
8021707_SOCS6	6.506655	7.68851	7.347572	7.303381	7.747348	7.281161
8022251_PPP4R1	5.952492	7.279	6.831706	7.011807	7.395327	7.264595
8022655_AQP4	7.6215	9.51124	8.924267	8.076221	9.625311	9.055943
8022767_KIAA1012	5.861487	6.982376	6.498351	6.213699	7.0164	6.655301
8022882_ZNF24	6.04055	7.603309	7.168509	6.546805	7.753398	6.846593
8022996_KIAA1632	6.476169	7.532572	7.417461	6.760932	7.548553	7.283916
8023165_HDHD2	6.710511	7.772547	7.534159	6.680486	7.907969	7.412574
8023191_SMAD2	5.318632	6.629969	6.368358	5.403514	6.742487	5.598137
8023605_PIGN	5.207051	6.223186	6.229778	5.492325	6.408622	6.142612
8023672_VPS4B	5.527772	6.649926	6.025972	6.425143	6.740703	6.496567
8023766_RTTN	5.534518	6.721046	6.22328	5.657667	6.819018	6.36318
8023984_HCN2	8.005863	6.998672	7.633749	7.968236	6.947374	7.351749

Supplemental Table 1. Gene expression values changed greater than 2 fold.

Affymetrix ST 1.0 Array ID	0-No	6-No	12-No	0-Yes	6-Yes	12-Yes
8024485_GADD45B	8.762878	7.112932	7.4543	9.710638	6.899641	7.231446
8024728_ITGB1BP3	8.639132	9.917049	9.551899	8.279519	7.992431	8.822286
8024792_EBI3	6.407486	5.189504	6.234445	6.021938	5.873393	6.084909
8025918_CNN1	7.071182	5.604464	5.650414	6.259866	5.423306	5.988407
8025998_ZNF136	4.991434	6.12733	5.634088	5.116683	6.139085	5.042331
8026047_JUNB	9.293178	7.271778	6.906909	10.38707	6.827059	6.95755
8026405_OR1I1	6.103382	5.063945	5.618841	5.824687	5.21541	5.140668
8026564_KLF2	9.868684	8.785648	8.951928	9.576199	8.657049	9.096869
8027564_FLJ12355	5.966549	4.905815	5.670523	5.389422	5.117832	5.027607
8028186_ZNF146	4.991184	6.392044	6.409647	5.873875	6.848838	5.465622
8028227_ZNF383	5.799856	6.966209	6.597018	5.962866	6.816703	6.411579
8028241_ZNF527	4.637551	6.058791	5.759415	5.545917	5.828475	5.653366
8028407_RYR1	9.824238	11.00854	10.55682	10.36052	10.85885	10.95736
8028546_LGALS7	8.100057	7.09552	7.235346	7.770919	6.969345	7.259626
8028652_ZFP36	8.770538	7.499611	7.605748	9.742706	7.381183	7.527718
8029065_BCKDHA	8.147715	9.175028	8.907167	8.733942	9.078901	9.207125
8029188_ZNF526	6.25941	7.265206	6.580303	6.646384	6.828934	6.564731
8029377_ZNF224	3.471896	5.293269	5.134138	4.83599	5.664361	5.048587
8029693_FOSB	8.151516	5.977442	6.025079	8.817471	6.031658	6.151453
8029710_VASP	8.174299	6.992518	7.408635	7.998082	7.354133	7.333655
8030366_SNORD35A	7.505475	8.694273	9.461499	7.514063	8.424659	8.258094
8030437_PRMT1	8.04505	9.068617	8.846718	8.361515	8.567266	9.037039
8030908_ZNF480	4.928524	6.280619	5.740882	5.225259	6.33567	5.447728
8031047_MYADM	9.089186	7.831856	8.601224	8.868054	8.088936	8.440325
8031057_PRKCG	5.476134	4.429543	4.820923	5.320653	4.951384	5.24624
8031573_LOC729974	4.390224	3.258468	2.759319	2.947773	3.377353	3.045353
8031714_ZNF460	7.713623	8.998984	8.662478	8.487189	8.826349	8.588904
8031726_ZNF304	4.265149	6.153916	5.510536	5.177927	5.490474	5.219018
8031744_ZNF17	4.872152	5.877206	6.065254	4.954644	6.270109	5.668549
8031799_ZSCAN4	4.150594	2.805118	3.201998	3.250821	3.345097	3.254894
8031807_ZNF551	4.841312	5.895798	5.763266	5.679883	5.707386	5.757251
8031815_ZNF776	5.618985	6.854077	6.652856	5.577194	7.048048	6.707155
8031837_ZNF587	5.175187	6.202733	6.173305	5.777835	5.451464	6.201465
8032365_KLF16	7.565694	6.361664	6.951841	7.206972	6.728872	6.978408
8032829_KIAA1881	7.921265	8.984493	8.318874	8.182335	8.73407	8.562179
8033809_LOC162993	2.960886	4.499238	3.067014	2.98553	3.508916	2.934981
8034021_CDC37	8.144083	9.210224	9.014595	8.691789	9.102393	9.208267
8034055_KRI1	5.446702	6.522729	6.260612	6.392742	6.457096	6.628263
8034454_DHPS	7.277514	8.358682	8.404413	7.547175	8.255015	8.53166
8035095_CYP4F11	5.823956	4.508492	4.671153	4.553885	4.501223	4.497857
8035345_INSL3	6.751441	5.604595	6.638082	6.399405	5.473648	6.341092
8035842_ZNF91	7.41842	8.657055	7.732789	7.293056	9.060096	7.394605
8036318_ZNF566	4.770161	6.079914	5.36564	4.63763	6.032548	5.702591
8036333_ZNF529	4.968304	6.084525	5.695606	5.560193	6.271187	6.022077
8036584_LGALS7	8.027025	6.979503	7.053347	7.520329	6.88337	7.168386

Supplemental Table 1. Gene expression values changed greater than 2 fold.

Affymetrix ST 1.0 Array ID	0-No	6-No	12-No	0-Yes	6-Yes	12-Yes
8037231_PSG3	4.583789	3.051515	3.499433	2.940063	3.532195	3.175056
8037236_PSG8	3.965631	2.854579	2.811694	2.606483	3.015734	2.977043
8037240_PSG1	4.559294	3.548354	4.217962	3.666389	3.881647	3.765754
8037970_PLA2G4C	6.095061	7.306896	7.146917	6.341307	7.150084	6.981429
8038109_FAM83E	7.737388	6.73482	7.050775	7.542614	6.562777	6.898401
8038213_HSD17B14	5.85631	4.646996	5.157691	5.721134	5.141565	5.489499
8038556_NAPSA	6.267007	5.264774	5.377411	5.795012	5.365956	5.478025
8038885_SIGLEC14	6.238766	5.174525	5.034288	4.953688	4.861942	5.665214
8038899_FPR1	6.248845	5.028698	6.33204	5.870804	6.529543	6.358839
8039144_TMC4	6.844451	5.674363	6.436612	6.141277	6.1882	6.100252
8039574_hCG_2042202	4.599171	3.547654	3.823635	4.224359	4.132582	3.45016
8039642_VN1R1	4.373217	5.889231	5.181575	4.986858	6.202434	4.955666
8039674_ZNF154	5.696438	6.712754	6.553059	5.598901	6.282957	6.276246
8039892_KIR2DS5	4.667335	3.656862	4.180315	4.280828	3.985627	4.367939
8040103_ID2	6.132775	5.01087	4.961761	6.266914	5.132195	5.041309
8040386_DDX1	8.225559	9.573435	9.122071	8.992666	9.693298	9.206366
8040889_DNAJC5G	5.036654	3.762761	3.725726	4.276723	3.442362	4.124261
8040985_ZNF512	5.597447	7.071549	6.55233	6.679574	7.084233	6.577807
8041179_CLIP4	7.580062	8.834744	8.647177	8.250185	8.815485	8.677566
8041283_BIRC6	6.845835	8.186935	7.94211	7.470812	8.392957	7.809272
8041422_RASGRP3	6.834305	8.269086	7.744912	7.060179	8.354463	7.77389
8041508_QPCT	5.197602	4.148986	4.2465	4.488847	4.325042	4.602765
8042161_PEX13	6.381371	7.549708	7.460664	6.822592	7.876948	7.357813
8042601_ZNF638	6.486288	7.656687	7.172963	7.114093	7.932032	7.042516
8042788_ACTG2	6.832311	5.304186	4.648211	5.931125	4.828603	5.087222
8042896_TLX2	8.069262	6.876853	7.507648	7.359385	7.019188	7.284833
8043251_PTCD3	8.311978	9.374013	9.231271	8.517942	9.519922	9.105098
8043278_MRPL35	5.512281	6.838221	6.908037	6.082808	7.493215	6.443816
8043310_RMND5A	8.056419	9.066272	8.931254	8.250842	9.204844	9.023059
8043697_KIAA1641	6.311493	7.390662	7.091472	6.449117	7.712357	6.794632
8043791_INPP4A	6.593919	7.636213	7.096076	6.799582	7.541551	7.735806
8044111_MRPS9	5.72906	6.843647	6.543657	6.234484	6.855472	6.86809
8044154_CD8B	6.662758	5.624859	6.048988	6.037159	5.762564	6.340213
8044263_RANBP2	6.569986	7.903286	6.651652	7.125744	8.199804	6.944616
8044450_ZC3H6	6.801493	8.234943	7.910236	7.338302	8.454557	7.465095
8045398_RAB3GAP1	6.52243	7.934356	7.588729	7.182148	8.079316	7.596789
8045455_UBXD2	7.349028	8.576765	8.110289	7.974014	8.979795	8.054813
8045688_TNFAIP6	5.088363	3.923174	4.210936	5.705601	3.945829	4.350649
8045697_RIF1	6.444125	7.891795	7.35905	6.911079	8.129822	7.247359
8045919_7-Mar	7.488984	8.893446	8.431789	8.420074	9.20686	8.470615
8046213_ZNF650	7.980405	9.246323	8.820024	8.636374	9.459205	8.731318
8046340_DYNC1I2	4.614342	5.818214	5.264596	5.238194	5.858253	5.308355
8046461_ZAK	7.530085	8.814208	8.20063	8.250947	8.976681	8.333865
8046502_SCRN3	4.80685	6.267849	5.459005	5.242998	5.940299	5.331595
8046604_AGPS	5.831501	7.055394	6.903346	6.731065	7.372208	6.934729

Supplemental Table 1. Gene expression values changed greater than 2 fold.

Affymetrix ST 1.0 Array ID	0-No	6-No	12-No	0-Yes	6-Yes	12-Yes
8046922_COL3A1	8.632978	7.392135	7.804177	8.600721	7.977146	8.247339
8047161_OBFC2A	6.039226	4.902498	5.579001	5.963352	5.275014	4.847351
8047228_MOBKL3	5.445461	7.136976	6.599523	6.151581	7.148664	6.571331
8047487_FZD7	7.229817	8.232013	8.077064	7.607108	8.241328	7.953997
8047518_NOP5/NOP58	6.179907	7.729946	7.258759	7.407353	8.178865	6.962898
8047784_ZDBF2	5.952007	7.469983	6.783531	6.444276	7.69734	6.521552
8047865_PIP5K3	6.244543	7.28382	6.768931	6.159254	7.293223	6.949435
8048014_RPE	4.137071	6.044708	6.313691	4.883709	6.615355	5.941701
8048370_BCS1L	5.94193	6.977545	6.622271	6.227985	6.459239	6.518669
8048411_TTLL4	5.394901	6.531255	6.087612	5.899049	6.123264	6.675405
8049097_DIS3L2	6.189643	7.237823	6.988499	6.663884	7.146883	6.66963
8049123_ALPP	6.551351	5.260187	5.512899	6.440424	5.434453	5.758164
8049187_EFHD1	7.858713	6.833043	7.175713	7.224105	7.472353	7.204
8049827_2-Sep	7.171836	8.29972	8.05811	7.750743	8.629712	8.438231
8050089_TMSL2	9.000086	7.547786	8.061917	8.254589	7.767682	7.756679
8050128_KIDINS220	7.30099	8.468151	8.037884	7.74211	8.468601	7.872897
8050278_PDIA6	8.250886	9.377458	8.993569	9.220822	9.517711	9.355036
8050302_ROCK2	7.120729	8.525672	7.598351	7.804646	8.822482	7.620656
8050367_NAG	6.214716	7.573433	7.306918	6.700234	7.592027	7.032726
8050443_SMC6	5.27668	6.370641	5.518306	5.437283	6.802105	5.310426
8050497_OSR1	6.68541	5.592231	5.898867	6.502452	5.766436	5.949377
8050565_PUM2	7.400354	8.628882	8.238811	7.644619	8.849766	8.277607
8051187_FNDC4	6.790419	5.782953	5.924991	6.476347	5.554602	5.807517
8051204_SUPT7L	6.083217	7.614254	7.347925	6.52326	7.502958	7.353221
8051464_HEATR5B	5.939003	7.029675	7.005178	6.067208	7.074584	6.537836
8051605_HNRPLL	5.587458	6.77112	6.47763	5.534837	6.859943	6.249777
8051622_SFRS7	6.353623	7.691301	7.609867	6.94929	7.489477	7.293655
8051746_THUMPD2	4.692863	6.177022	5.797101	5.449101	6.369881	5.472073
8051820_THADA	5.600204	6.767699	6.760129	6.398377	7.086381	6.956575
8051882_LRPPRC	8.022514	9.29416	8.807138	8.669077	9.412244	8.759909
8051963_SRBD1	5.381197	6.545294	6.241297	5.250446	5.790504	5.487973
8051998_MCFD2	6.223897	7.583145	7.401635	6.840006	7.584584	7.237233
8052125_ASB3	5.751332	6.849546	6.35129	6.253231	6.957207	6.405028
8052149_PSME4	7.169946	8.21274	7.859864	8.182014	8.545101	8.061656
8052250_MTIF2	5.078366	6.593877	6.150297	5.83564	6.808629	5.967824
8052331_PNPT1	6.191531	7.517735	7.229072	6.803967	7.799739	7.236409
8052443_USP34	7.0859	8.141494	7.664607	7.319059	8.370236	7.504652
8052526_XPO1	6.89491	8.253815	7.864547	7.58782	8.478696	7.868464
8052733_WDR92	3.177299	2.132719	2.077127	2.162792	2.191856	2.085004
8052956_EXOC6B	5.765168	7.344803	7.084164	6.371142	7.223253	7.000013
8053288_C2orf3	4.981508	6.093198	5.532322	5.131231	6.428814	6.220582
8053801_LOC400986	6.542854	7.818015	7.586531	6.799006	8.143288	7.366791
8053834_ANKRD36	6.422538	8.223663	7.695903	6.879513	7.980084	7.625381
8053909_ASCC3L1	8.259038	9.275983	8.820666	8.538218	9.107604	9.299545
8054004_SEMA4C	6.834575	5.782662	6.141354	6.612709	6.411488	6.363964

Supplemental Table 1. Gene expression values changed greater than 2 fold.

Affymetrix ST 1.0 Array ID	0-No	6-No	12-No	0-Yes	6-Yes	12-Yes
8054054_KIAA1641	6.634901	7.771272	7.555642	6.486748	7.776549	6.65754
8054092_TMEM131	7.563684	8.802523	8.430947	7.77535	8.708357	8.283321
8054217_TXNDC9	3.569757	4.589591	4.644726	4.450646	5.179024	4.754526
8054227_REV1	6.245727	7.28048	7.053826	6.503706	6.992728	7.079955
8054614_ANAPC1	6.711529	7.882618	7.786877	7.209598	8.11938	7.802591
8054804_CCDC93	5.836243	7.283175	6.768058	6.429832	7.142121	6.723699
8054888_CLASP1	8.375849	9.430321	9.147204	8.657588	9.506293	9.177184
8054978_ERCC3	6.663968	7.67887	7.415561	6.779675	7.622743	7.422354
8055060_WDR33	6.574886	7.652749	7.062961	6.95796	7.813223	7.365849
8055645_ORC4L	5.799585	7.096401	6.444378	6.793717	7.404749	6.752995
8055952_NR4A2	6.371752	5.120424	5.204181	7.01383	4.901578	5.416981
8056060_BAZ2B	6.259481	7.443087	6.881425	6.438134	7.629235	6.728921
8056184_ITGB6	8.187037	9.46306	8.930511	8.174288	9.73649	8.933064
8056220_AHCTF1	5.723403	7.489018	6.945007	6.662965	7.650494	6.844319
8056766_SLC25A12	8.825564	10.1359	9.720874	9.006014	9.985952	9.683853
8056943_KIAA1715	7.931066	9.007393	8.843438	8.083227	9.171571	8.669041
8056959_EVX2	7.494597	6.244774	6.657982	6.66888	6.276026	6.211392
8057713_MSTN	3.660225	5.300138	5.062201	4.905748	5.929182	4.350984
8057887_STK17B	7.529081	6.36418	6.756229	7.457719	6.734733	6.687523
8057933_GTF3C3	5.668039	7.192365	6.949188	6.567526	7.401725	7.136538
8058373_WDR12	5.511561	6.571756	6.970222	6.222913	7.017307	6.683805
8058415_FLJ20309	7.003546	8.141356	7.793265	7.359811	8.07551	7.79087
8058428_NDUFS1	8.759866	9.864206	9.642447	9.142391	9.887994	9.585054
8058591_ACADL	4.691312	6.051514	5.364834	5.099936	5.825628	5.074595
8058670_IKZF2	5.279056	6.37287	5.901131	5.488519	6.436861	5.833286
8058695_BARD1	6.175232	7.18737	6.929228	6.478902	7.601887	7.219054
8059319_FARSB	6.974694	7.978939	7.926203	7.180621	8.153651	7.816172
8059538_SLC19A3	5.052458	3.975205	4.99995	5.138447	4.179455	4.724062
8059596_TRIP12	7.899312	9.115261	8.736516	8.856354	9.387424	8.889963
8059965_C2orf19	5.370965	4.178483	4.635401	4.86318	4.480506	4.023216
8060447_TGM6	6.294	5.165223	4.975148	5.624228	4.95809	5.264364
8060627_ATRN	6.298578	7.465286	7.222036	6.826812	7.228728	7.17651
8060850_BMP2	6.891404	5.886711	6.537416	7.0521	6.237359	6.333465
8060854_PLCB1	5.667174	7.321629	6.746586	5.992881	7.249249	6.416526
8061227_SLC24A3	6.422753	7.940776	7.952073	6.12347	7.688782	7.905032
8061497_C20orf80	5.268326	6.605289	6.118631	6.708419	6.50378	6.181037
8062119_MT1P3	7.180611	6.002219	6.477207	7.548656	6.256279	6.472919
8062461_LBP	6.286387	5.212593	5.207602	7.377002	5.208519	5.241776
8062490_SNORA60	7.588645	6.473553	8.203283	7.096704	6.196376	6.46226
8062539_SLC32A1	6.059535	5.001223	5.339808	5.514997	5.440259	5.624235
8062695_SFRS6	7.455022	8.456853	8.293722	7.874181	8.822213	8.435187
8063129_SLC12A5	5.139679	3.900044	4.162793	4.737593	4.117938	4.32607
8063242_ARFGEF2	6.363956	7.52328	7.046239	7.010069	7.45298	7.14084
8063283_CSE1L	6.872151	8.107263	7.940551	7.110138	8.415841	7.703716
8063458_DOK5	5.770695	7.466708	7.391335	5.9925	7.372475	7.683246

Supplemental Table 1. Gene expression values changed greater than 2 fold.

Affymetrix ST 1.0 Array ID	0-No	6-No	12-No	0-Yes	6-Yes	12-Yes
8063942_NTSR1	5.903194	4.767906	5.162936	5.697961	4.944909	5.156658
8064302_PCMTD2	7.531715	8.741731	8.569899	7.814797	8.806947	8.626911
8064522_IDH3B	7.281675	8.491836	8.538614	7.854524	8.737604	8.5681
8064859_PROKR2	5.210982	4.196023	4.85405	4.640471	4.487923	4.947051
8065032_ESF1	4.516368	6.158349	5.128051	5.30434	6.27484	4.997325
8065280_C20orf74	7.854138	8.959286	8.516755	8.138299	9.036542	8.656762
8065353_THBD	8.380108	7.252122	7.300261	10.15004	7.344264	7.534771
8065795_HMG4L	5.275943	4.266675	4.055606	4.624536	4.841227	4.770298
8065832_TRPC4AP	7.358172	8.672301	8.54744	8.396754	8.426548	8.504089
8066136_RBL1	6.091452	7.228913	6.925132	6.480974	7.608819	7.144086
8066262_SNORA71D	8.432831	7.222007	8.841366	9.184219	7.786943	7.494229
8066303_CHD6	6.111861	7.235965	6.860469	6.881031	7.069991	6.728753
8066402_C20orf111	6.552127	7.780246	7.669835	7.103459	7.8587	7.862597
8066776_TP53RK	6.896187	5.744438	6.091659	5.982641	5.880546	5.965119
8066925_PTGIS	8.251355	7.175074	7.50038	7.849718	7.622277	7.896745
8067017_DPM1	7.354692	8.466303	8.310452	8.095512	8.794555	8.580364
8067033_KCNG1	6.35382	5.286343	5.545177	5.594009	5.203067	5.569033
8067842_LOC646442	4.747457	3.509342	3.617012	3.681694	2.999748	3.184413
8068180_HUNK	6.049829	5.038598	5.349103	5.307162	5.40196	5.719015
8068422_DOPEY2	6.160399	7.326819	7.078645	6.562725	6.926597	7.153443
8068522_TTC3	6.675667	7.925218	6.89344	7.070333	8.114043	7.253244
8068620_SH3BGR	7.596634	8.825556	8.839735	7.781353	8.534199	8.628044
8069561_BTF3L4	6.706472	7.710286	7.358474	7.134047	7.880208	7.661196
8069620_MRPL39	6.465792	7.720154	7.441717	6.855744	7.830146	7.564053
8069668_CYYR1	6.688721	7.826735	7.648748	7.439144	8.1322	7.56111
8069676_ADAMTS1	7.485305	6.225791	6.256622	9.018669	6.244499	5.600326
8069711_ZNF294	5.758447	7.048366	6.372746	5.947279	7.319855	6.414433
8069868_KRTAP7-1	5.236916	4.143264	4.153214	4.12571	4.10985	3.779109
8070083_TMEM50B	5.715192	6.947729	6.938682	6.34617	7.060151	6.716719
8070239_HLCS	5.531929	6.87357	6.600945	6.227061	6.774179	6.479449
8070269_DSCR3	6.410235	7.97169	7.463164	6.990444	7.731404	7.879274
8070467_TMPRSS2	5.773085	4.578099	4.923872	4.777735	4.823051	4.743502
8070665_SNF1LK	8.290503	6.896962	6.344887	8.853451	6.469741	6.887445
8071049_LOC51152	5.002801	6.089117	5.536351	4.888236	5.638752	5.107072
8071147_TUBA8	7.716179	8.808884	8.770834	8.026009	8.637367	8.739582
8071332_RANBP1	6.644985	7.706542	7.402615	6.922155	7.64366	7.575917
8071881_SPECC1L	7.066002	8.08545	7.656945	7.870642	7.983263	8.04076
8072279_MTMR3	8.037446	9.097626	8.988645	8.529974	9.068017	8.970113
8072584_LOC402057	5.935874	4.742544	6.109021	5.415102	5.637026	5.505474
8074030_SAPS2	6.413905	7.755363	7.317631	7.399016	7.263101	7.689267
8074817_TOP3B	5.605814	6.808681	6.84916	6.49097	6.706388	6.78513
8074931_ZNF70	5.464876	6.639563	6.477846	5.940206	6.44612	6.446647
8074969_DDT	6.597651	7.898061	7.945794	6.479189	7.518476	7.583418
8075130_PITPNB	6.497092	7.618416	7.397397	7.093157	7.674504	7.387529
8075285_ASCC2	6.234465	7.469671	7.260778	7.191805	7.654528	8.089682

Supplemental Table 1. Gene expression values changed greater than 2 fold.

Affymetrix ST 1.0 Array ID	0-No	6-No	12-No	0-Yes	6-Yes	12-Yes
8075921_LRRC62	6.18799	5.115763	5.484681	5.876888	5.185881	5.439252
8076449_dJ222E13.2	7.786149	9.208582	8.441919	8.969748	9.089245	9.207889
8076655_PNPLA5	6.148646	4.926027	4.992626	5.299593	4.557611	4.730364
8077099_SCO2	7.375792	6.340218	6.146087	6.441928	6.281948	6.537916
8077270_CHL1	4.898484	3.742571	4.191664	4.495168	3.996868	3.659606
8077458_EDEM1	5.190742	6.291294	5.813263	5.633139	6.035072	6.004124
8077499_LOH3CR2A	5.109907	6.58352	6.031962	6.425535	6.846078	6.187131
8078014_SLC6A6	7.037897	8.258157	8.021602	7.368584	8.267934	8.314598
8078110_CAPN7	6.577823	8.195439	7.726956	7.525373	8.213735	7.577705
8078155_GALNTL2	6.276431	7.484404	7.105619	7.153694	7.329304	7.248882
8078187_PLCL2	6.629525	7.663649	7.458072	6.985109	8.008768	7.461112
8078272_NR1D2	7.920283	9.238858	8.932425	8.189589	9.545743	8.935694
8078360_STT3B	8.208526	9.363653	8.956104	8.896044	9.480744	9.103364
8078386_GPD1L	7.539754	9.258488	8.899054	8.336898	9.440554	9.276134
8078569_GOLGA4	6.527112	7.587938	6.730547	7.096375	8.134932	6.521513
8078805_ACVR2B	6.014665	7.559092	6.89962	6.574192	7.319804	7.186324
8078834_WDR48	5.850529	7.124404	7.076655	6.399749	7.1974	6.706578
8078991_ZNF619	4.176204	5.356384	5.501619	4.332067	5.648095	5.513257
8079079_NKTR	6.579645	7.77018	7.100513	6.682766	7.977551	7.274424
8079170_C3orf23	6.362793	7.451747	7.178313	6.765735	8.132552	7.27864
8079229_ZNF501	3.914321	5.311245	5.130136	4.345165	5.272086	4.62822
8079311_LARS2	5.886267	6.946362	6.569382	5.720777	6.734321	6.637816
8079662_ARIH2	7.479721	8.645439	8.37371	7.839396	8.720618	8.788159
8079693_WDR6	6.728428	8.010379	7.706191	7.30805	7.89847	7.900406
8079869_RBM5	7.496135	8.752399	8.781358	7.775399	8.646317	8.548191
8079931_SLC38A3	7.32244	8.362087	7.761786	7.54635	8.187495	8.365579
8080013_MAPKAPK3	7.384438	8.548413	8.564707	7.912146	8.188143	8.730522
8080184_ALAS1	7.607817	8.655146	8.279036	8.225655	8.431105	8.677473
8080773_RPP14	7.583671	8.742931	8.499643	7.825383	8.914495	8.873556
8080810_PTPRG	6.137495	7.145087	6.889224	6.883147	6.852431	6.749992
8080987_HSP90AB5P	4.69788	3.683144	3.485591	4.121923	3.415914	3.569525
8081171_DKFZp667G2110	6.256404	7.705916	6.819661	7.260863	8.225382	7.084902
8081375_FAM55C	7.581606	8.721714	7.949855	8.407887	8.75544	8.619503
8081740_ATP6V1A	5.847629	6.939391	6.77155	6.910968	6.991372	7.01592
8081779_ZDHHC23	6.761247	5.384967	6.295263	6.352047	5.765266	5.758424
8081997_FBXO40	8.671465	9.798351	9.508049	9.275706	9.606785	9.389498
8082035_CD86	5.174373	4.046591	3.991336	4.256634	4.313111	4.244684
8082086_PARP15	4.388597	5.403091	5.003502	4.272761	4.841154	4.746607
8082120_DIRC2	7.627904	8.740486	8.620175	7.90403	8.821646	8.493406
8082400_KLHDC6	7.328912	8.614969	8.349958	7.720779	8.696123	8.475643
8082607_ATP2C1	6.649956	7.838685	7.424165	6.958597	8.199727	7.327524
8082827_CEP63	4.705669	5.846835	5.510798	4.924581	5.971858	5.201963
8082869_PPP2R3A	8.128263	9.79182	9.484043	8.662481	9.78758	9.334387
8083053_TRIM42	5.335558	3.906898	4.212914	4.462976	4.1975	4.354423
8083063_SLC25A36	6.477787	7.74754	7.401162	7.209741	7.85591	7.396848

Supplemental Table 1. Gene expression values changed greater than 2 fold.

Affymetrix ST 1.0 Array ID	0-No	6-No	12-No	0-Yes	6-Yes	12-Yes
8083183_SR140	7.35713	8.357978	8.195457	7.840322	8.833571	7.779685
8083494_MME	6.005337	7.156663	6.985555	6.704545	7.900579	7.181077
8083599_C3orf55	4.128091	5.334866	4.226476	4.54824	4.715103	4.233743
8083616_MLF1	6.535436	7.553337	7.647114	6.80933	8.07444	7.882572
8083630_GFM1	5.960793	7.789989	7.434204	6.973706	7.544672	7.640708
8083656_MFSD1	6.198749	7.572932	7.450335	7.042335	7.539605	7.289724
8083757_NMD3	5.998997	7.643337	7.021481	7.248078	7.842149	6.954909
8084100_USP13	9.727424	10.73098	10.37305	10.30239	10.61338	10.46437
8084219_KLHL24	8.189652	9.286034	9.094408	8.799733	9.538008	9.062371
8084541_VPS8	6.708516	8.392653	7.855771	6.957884	8.462757	7.920657
8084607_SENP2	6.007724	7.034519	6.885398	6.533606	7.063482	6.578836
8084708_SNORA4	4.839039	6.117062	5.992867	5.482524	6.139233	6.075901
8084710_ADIPOQ	6.652029	5.050607	7.386399	7.43758	5.178266	5.982008
8084742_LPP	7.81568	9.026307	8.856594	8.305309	9.138684	8.799104
8084757_FAM79B	6.125474	7.837394	7.461894	6.304491	7.393816	6.436551
8084844_OPA1	6.776596	8.38672	7.548965	7.555436	8.880983	7.684041
8084912_SDHA	8.443275	9.701252	9.626863	9.008591	9.754839	9.630711
8085054_DKFZP434B2016	4.255763	6.026196	5.476753	5.267758	5.447934	5.641898
8085081_CRBN	7.792084	8.825621	8.675825	8.13008	9.051458	8.367844
8085244_CIDEC	7.242583	5.66333	6.78391	6.612779	5.511955	5.682105
8085311_ATP2B2	7.004485	8.057258	7.743362	7.146741	7.857637	8.006808
8085504_GRIP2	5.799632	6.864269	6.154985	6.410943	6.702579	6.526863
8085531_MRPS25	7.313882	8.328562	8.153569	7.356184	8.173987	8.225893
8085537_ZFYVE20	4.746747	6.147882	5.50182	5.876227	6.23993	6.284971
8085815_TOP2B	6.004909	7.105986	6.241096	6.026671	7.215487	6.304749
8085867_NEK10	6.090257	7.631092	7.197746	5.584498	7.490192	6.384245
8085972_GADL1	4.765821	7.026444	6.326088	5.806799	6.522945	6.299081
8086077_CLASP2	8.544527	9.624858	9.291397	8.702431	9.694507	9.295463
8086141_EPM2AIP1	6.18271	7.533547	7.090145	6.841358	7.571494	7.364873
8086216_ACAA1	4.556634	3.432564	3.989402	3.085595	3.406806	3.512413
8086330_AXUD1	7.539693	6.40331	6.276914	8.580238	6.514306	6.498477
8086341_XIRP1	10.35317	9.308409	8.987867	11.2906	9.359772	9.394249
8086344_CX3CR1	4.645247	6.1487	6.772928	5.155847	5.955734	6.268933
8086538_LOC644714	5.599468	4.351937	4.099707	5.02061	3.552668	2.884873
8086572_FYCO1	9.051054	10.11104	9.82287	9.635769	10.02788	10.03766
8086615_LRRC2	7.83505	8.905492	8.847433	8.290597	8.832987	8.505339
8086706_SETD2	6.419533	7.975893	7.488251	7.276702	7.879286	7.359249
8087283_QARS	7.42583	9.011413	8.781369	8.358589	8.760674	9.097616
8088180_WNT5A	4.331536	5.483757	4.894704	4.359457	4.860292	4.835241
8088292_ASB14	7.16719	8.707767	7.85456	7.647107	8.038542	8.209726
8088664_SUCLG2	6.90087	8.313594	8.572264	7.566116	8.241339	8.18548
8088848_PDZRN3	6.747738	8.194304	7.800689	7.091378	7.740909	8.062996
8089000_CGGBP1	6.94197	8.21472	8.075997	7.546553	8.414971	7.885015
8089062_CLDND1	7.795676	8.872375	8.703966	8.884195	9.045088	8.650203
8089112_FILIP1L	7.991701	9.267059	8.366612	8.387036	9.41766	8.307313

Supplemental Table 1. Gene expression values changed greater than 2 fold.

Affymetrix ST 1.0 Array ID	0-No	6-No	12-No	0-Yes	6-Yes	12-Yes
8089544_CCDC80	8.667182	6.681501	6.869756	8.911541	7.150148	7.053267
8090018_PARP9	5.521021	6.646634	6.595455	5.929945	6.46196	6.609159
8090256_SNX4	5.89447	7.065711	6.670922	6.339509	7.069678	6.462937
8090277_OSBPL11	6.447298	7.469312	6.822185	6.784433	7.694022	7.020663
8090715_ACAD11	5.148045	6.286645	6.175786	5.69662	6.541151	6.040829
8090772_TOPBP1	5.306211	6.457461	5.853109	5.604515	6.296872	6.047342
8090823_SLCO2A1	6.496832	5.37812	5.910325	6.17102	5.798079	5.925262
8090866_ANAPC13	6.345175	7.885399	7.533694	7.044456	7.65128	7.616923
8090872_KY	6.278491	8.344924	8.159504	6.40702	7.698461	8.085257
8090898_STAG1	5.521114	7.014029	6.338626	6.47447	7.197907	6.648425
8091103_TFDP2	6.758499	7.892834	7.572999	7.465031	8.098722	8.081059
8091562_DHX36	5.174329	6.569866	5.660998	5.957624	6.725434	6.008995
8091656_METT5D1	5.228337	6.411461	5.442932	5.338218	6.694911	5.614749
8091757_TRIM59	4.414012	3.263724	3.951236	4.064604	3.658283	3.583899
8092000_TERC	7.43673	6.351786	6.694143	6.891623	6.502469	6.761894
8092095_TNIK	6.756947	7.966429	7.602206	7.037341	8.033722	7.631808
8092265_MRPL47	6.502612	7.661311	7.724434	7.113995	7.812764	7.609206
8092321_DCUN1D1	7.625804	8.648374	8.23773	7.749325	8.662654	7.817261
8093039_SDHA	8.947365	10.07747	9.80554	9.313535	10.11862	10.03216
8093053_TFRC	7.693329	8.797586	8.515755	8.30392	8.75289	8.382284
8093137_C3orf43	6.682569	8.053427	8.141285	7.607646	8.657691	8.482891
8093792_HGFAC	7.030895	5.803207	5.743972	6.626037	5.989223	5.933523
8094136_DEFB108B	5.177466	3.841081	3.939958	3.8974	4.016368	3.934324
8094184_C1QTNF7	4.813341	3.413545	3.773129	4.449823	3.634261	4.466221
8094240_CD38	6.861079	7.973947	7.490767	7.201434	7.865206	7.769091
8094361_GBA3	4.757825	3.565678	4.183476	3.933654	3.901705	3.765068
8094408_ANAPC4	5.034964	6.17264	5.809312	5.401148	6.082226	5.473036
8094848_SLC30A9	6.370384	7.439666	7.053991	6.786888	7.955133	7.274402
8094876_GUF1	6.503618	7.593551	7.321887	6.869264	7.954326	7.47973
8095246_ARL9	4.803094	3.65978	3.860048	4.259188	3.69084	4.349673
8095269_POLR2B	8.317193	9.545837	8.98961	8.615877	9.574018	9.234456
8095535_ENAM	6.162067	7.284157	7.059754	5.554153	7.155503	5.911215
8095566_MOBKL1A	4.78465	5.926132	5.409755	4.888669	5.676497	5.626869
8095751_DKFZP564O0823	6.268747	7.411425	6.925951	5.935259	6.625082	7.098955
8095760_THAP6	4.663417	5.711557	5.516	5.05588	6.226731	5.317435
8095773_VDP	8.622567	9.813067	9.36285	9.10508	10.22378	9.186162
8095806_ART3	7.485842	8.935991	8.925621	7.559059	9.31875	8.562136
8096091_THAP9	5.215466	6.374348	5.832296	5.958847	6.23122	5.531841
8096361_HERC5	5.207003	4.171106	5.102861	4.447623	4.45052	4.538453
8096875_ENPEP	4.613973	5.967737	5.588405	5.3342	5.667049	4.908877
8096959_ANK2	6.134856	7.964004	7.289753	6.862264	7.917092	7.520822
8097098_USP53	7.772115	9.20435	8.81978	8.309285	9.605376	8.355961
8097148_KIAA1109	6.765693	8.098138	7.63324	7.242734	8.35717	7.447947
8097373_C4orf29	5.13186	6.948437	6.839526	5.959617	6.841089	6.696972
8097480_NARG1	5.333937	7.159118	6.380653	6.428807	7.454849	6.17838

Supplemental Table 1. Gene expression values changed greater than 2 fold.

Affymetrix ST 1.0 Array ID	0-No	6-No	12-No	0-Yes	6-Yes	12-Yes
8097570_USP38	7.887848	9.172788	8.849461	8.726595	9.271754	8.826264
8097586_GAB1	7.946681	9.027669	8.921125	7.865668	9.170133	8.675385
8097670_MMAA	5.271219	6.61153	6.460769	5.619391	6.97629	6.591034
8097792_SNORD73A	4.500447	5.506315	5.146609	4.366568	5.42708	5.241966
8097867_KIAA0922	7.514137	8.606395	8.440352	7.97885	8.548324	8.247764
8097903_TLR2	6.083288	4.61984	5.430846	5.028737	5.068931	4.939699
8098084_ETFDH	7.722303	8.88549	8.420108	8.357606	9.001122	8.5215
8098705_MTRF1L	7.020316	8.192665	8.076931	8.060979	8.198698	8.179053
8098758_ZNF721	5.465216	7.339076	6.510387	6.302568	7.7211	6.008951
8099121_STX18	6.602246	7.758646	7.55641	7.106022	7.643301	7.29213
8099255_PSAPL1	6.212718	4.994458	5.658711	5.760283	5.55626	5.377573
8099612_GPR125	5.777257	7.160264	7.084518	6.05139	7.137134	6.829823
8099633_PPARGC1A	7.047677	8.054837	7.702889	8.52474	8.019953	7.401549
8099860_RFC1	5.53343	6.622722	6.177432	5.619859	6.6964	5.47896
8099982_APBB2	6.931758	7.968741	7.934232	7.252529	7.726602	7.729429
8100026_ATP8A1	7.538455	8.90967	8.629376	8.203336	9.104395	8.300015
8100076_YIPF7	4.604855	6.148519	6.079689	4.579904	6.650743	6.063931
8100085_GNPDA2	4.04145	5.150901	4.760912	4.728537	5.345567	4.849503
8100318_SGCB	8.490425	9.506002	9.258757	8.89065	9.773689	9.150166
8100984_CXCL3	6.678727	5.647064	5.895026	6.242197	5.623396	6.096525
8100994_CXCL2	6.671572	4.678912	4.818462	7.235061	4.617105	4.988464
8101143_NUP54	5.796901	7.052485	6.512696	6.693284	6.971008	6.872311
8101256_GK2	3.869499	2.774752	3.374381	3.189482	2.938562	3.687706
8101893_ADH1C	5.960032	7.090829	7.503164	6.504048	7.508844	7.352209
8101952_DDIT4L	8.063736	9.480319	9.314479	8.285501	9.362995	9.201422
8101957_EMCN	6.164074	7.632998	7.166211	6.689625	7.849234	7.136508
8102730_MFSD8	5.580639	6.819049	6.919701	5.364905	7.084758	6.677812
8102938_RNF150	6.808076	7.880921	7.730542	7.064123	7.299355	7.442532
8102950_INPP4B	5.930926	7.328314	6.984474	7.152455	7.365976	6.583685
8103188_PET112L	6.370656	7.407844	6.884761	6.934996	6.82481	7.163619
8103206_FBXW7	6.306002	7.495542	6.912581	6.607322	7.572817	6.579363
8103254_SFRP2	6.401331	4.895326	5.148469	6.317316	4.925418	4.906787
8103289_PLRG1	6.785182	7.849204	7.656415	7.550276	8.258553	7.685008
8103881_RWDD4A	6.085065	7.240277	7.284822	6.48222	7.465645	7.359106
8104201_EXOC3	6.889921	7.972755	8.154075	7.909221	8.010258	8.218008
8104220_CEP72	6.623234	5.568245	5.224268	6.032433	5.637088	5.898588
8104369_SRD5A1	7.394628	6.199515	6.757295	6.435359	7.170953	7.027285
8104746_NPR3	4.931874	3.854722	3.583239	5.38402	3.581998	3.790243
8105013_EGFLAM	6.111676	7.440398	7.005781	6.577013	7.071646	6.624938
8105121_GHR	7.108428	8.554229	8.003792	7.821548	9.185893	7.875737
8105144_FLJ10246	5.776376	6.917171	6.393342	6.197426	7.164927	5.944396
8105153_NNT	9.317467	10.62389	10.33697	10.05336	10.64727	10.58112
8105353_SKIV2L2	7.42186	8.734611	8.098198	8.094122	8.981308	8.128236
8105545_IPO11	5.804375	6.839852	6.585442	6.392116	7.443257	6.900546
8105612_SDCCAG10	5.161234	6.166768	5.825299	5.515305	6.416181	5.675943

Supplemental Table 1. Gene expression values changed greater than 2 fold.

Affymetrix ST 1.0 Array ID	0-No	6-No	12-No	0-Yes	6-Yes	12-Yes
8105681_ERBB2IP	6.848452	8.157627	8.113743	7.526184	8.879951	7.489057
8106088_CARTPT	4.871395	3.795715	4.336544	4.1664	4.13505	4.640756
8106107_PTCD2	6.147945	7.27479	7.00259	6.955952	7.530658	6.819898
8106556_CMYA5	10.45578	11.4708	11.02727	10.78533	11.58352	10.97706
8106573_THBS4	10.34515	8.109584	8.275132	10.1714	8.606795	8.910921
8106602_ZFYVE16	4.598666	6.286182	5.784432	5.432183	6.549152	5.656438
8106689_CKMT2	10.35402	11.38708	11.22528	10.44486	10.91921	11.17094
8107350_SRP19	8.2992	7.095378	7.456129	8.004745	7.075124	7.459213
8107375_YTHDC2	6.17367	7.248677	6.825038	6.33082	7.29045	6.726079
8107458_COMMD10	6.64718	7.687486	7.43237	6.567523	7.63998	7.172961
8107722_MEGF10	5.664264	7.257982	6.696802	6.440986	6.188697	6.107065
8107764_CTXN3	4.389747	6.06218	5.930466	4.757283	6.647293	6.74418
8107769_SLC12A2	7.576365	8.891905	8.772202	8.162159	9.240104	8.82636
8108238_SMAD5	6.473502	7.478193	7.44841	6.942935	7.724077	7.292854
8108330_JMJD1B	7.550869	8.943871	8.653065	8.457826	8.958766	9.22376
8108370_EGR1	10.80861	8.262308	7.754484	11.49982	7.02523	7.29497
8108420_SNORA74A	4.279186	5.80512	6.006416	4.816799	5.244828	4.969057
8108603_HARS2	5.521379	6.685658	6.490182	6.168986	6.496342	6.682161
8108716_PCDHB16	4.848211	5.923066	5.321876	5.399931	5.356405	5.529039
8109062_FBXO38	6.162562	7.471303	7.03764	6.812853	7.439125	7.119112
8109490_SGCD	6.980578	8.167074	7.699939	7.830758	8.377323	7.927261
8109752_ODZ2	5.373362	4.361986	4.591275	4.713133	4.619836	4.44341
8111552_C5orf33	5.929045	7.318058	6.88754	6.461787	7.577586	7.106896
8111629_NUP155	5.69849	6.726391	6.550671	6.29995	7.037786	6.235542
8111670_GDNF	5.907029	4.718124	4.57772	5.829392	4.596916	4.609691
8111698_RICTOR	6.685557	7.843648	7.546046	7.162069	8.280245	7.345745
8111941_HMGCS1	5.311001	6.319129	6.453144	6.46406	6.332919	6.268431
8112020_MOCS2	6.309926	7.429128	7.132874	6.837451	7.317999	7.348198
8112302_DKFZP686E2158	4.571459	5.594911	4.927553	5.21778	5.34851	4.849043
8112312_DIMT1L	5.536374	6.600917	6.714803	6.073684	6.701307	6.421941
8112388_TRIM23	5.493024	6.584788	6.224885	6.132694	7.210476	5.97904
8112584_ZNF366	5.649287	6.784911	6.666289	6.000186	6.411001	6.791661
8112622_GFM2	6.574481	7.600168	7.411708	6.768481	7.657783	7.264982
8112687_COL4A3BP	6.276433	7.448868	6.849705	6.77725	7.673969	7.150882
8112803_LHFPL2	7.179554	5.921986	6.383837	6.689382	5.90882	6.394537
8112857_MTX3	4.985054	6.558804	6.59923	5.78973	6.289066	6.068552
8112967_TMEM167	4.699557	5.707513	5.522379	5.148444	6.387733	5.741707
8113278_LIX1	5.002801	3.797533	4.160357	4.174248	4.067877	3.799347
8113551_MCC	5.536942	6.703151	6.116183	6.222625	6.45103	6.76137
8113914_FNIP1	7.629702	8.662353	8.283291	8.505995	8.870033	8.338652
8114256_IL9	5.038004	3.91227	3.898477	4.466384	3.798207	3.906088
8114861_LARS	7.134021	8.170815	7.938342	7.344033	8.202876	7.95416
8115164_RPS14	4.042208	5.164174	4.874151	4.496195	4.653604	4.813943
8115168_RBM22	7.105299	8.117551	8.116304	7.627214	8.374024	8.348523
8115831_DUSP1	10.55148	9.411124	9.301301	11.18536	9.375262	9.572777

Supplemental Table 1. Gene expression values changed greater than 2 fold.

Affymetrix ST 1.0 Array ID	0-No	6-No	12-No	0-Yes	6-Yes	12-Yes
8116227_CLK4	6.919474	7.98335	7.964492	6.800115	8.670108	7.518662
8116245_CLK4	7.263465	8.541848	7.819933	7.130722	8.482423	8.001325
8116807_C6orf151	5.506109	6.678516	6.238868	5.483314	6.975701	6.436212
8116848_PAK1IP1	5.021514	6.199564	6.072715	5.154397	5.931372	5.664162
8117426_HIST1H2BH	7.307225	6.19811	6.548369	6.575416	5.967165	6.601477
8117600_HIST1H2BN	6.157957	4.76542	4.807092	5.305008	5.115452	5.244269
8117646_ZNF192	6.785401	7.8793	7.53867	7.055384	8.103703	7.649144
8117696_COX11P	4.543936	5.546686	5.192733	4.848418	6.178725	5.243518
8117718_OR2J3	4.097828	2.979928	2.448316	3.02333	2.868187	3.008365
8118158_AIF1	6.435225	5.408065	6.254071	6.066275	6.066275	6.159676
8118345_CFB	6.368966	5.340623	5.092551	5.228562	5.622992	5.549184
8119529_UBR2	7.574639	8.854985	8.475815	8.449891	9.057459	8.448057
8120210_IL17A	6.335513	5.044392	5.510798	5.835307	5.545016	5.67288
8120441_PHF3	6.703293	8.152976	7.623341	7.402022	8.581897	7.054873
8120468_BAI3	5.15787	3.860829	4.346117	4.528194	4.067542	4.175898
8120654_KCNQ5	6.614124	8.447592	8.355597	7.294599	8.538165	8.108864
8120883_DOPEY1	6.317051	7.49715	6.777624	6.187353	7.299452	6.519287
8121031_SLC35A1	5.18757	6.371006	6.133139	5.745195	6.64498	6.238357
8121043_ORC3L	6.08424	7.509221	7.192829	6.507496	7.660481	7.163376
8121161_KIAA0776	6.48574	8.125509	7.379442	7.207089	8.216389	7.361615
8121515_SLC16A10	7.939645	9.472992	9.079269	8.203062	9.280748	9.035582
8121685_DCBLD1	5.634605	7.074717	6.57635	6.670821	7.082269	6.412398
8121757_HSF2	7.16796	8.281683	8.176487	7.346175	8.459373	7.806139
8121825_RNF217	5.043739	6.21041	6.294989	5.976954	6.589739	6.022988
8121838_TPD52L1	7.590655	8.652014	8.547109	7.922719	8.385895	7.789453
8122131_TAAR6	4.238544	3.171558	3.527528	3.527123	4.020359	3.663776
8122150_EYA4	6.757042	8.060521	7.590796	7.291535	7.653749	7.098737
8122279_KIAA1244	5.839011	4.734441	4.822813	6.269932	4.92988	4.864172
8122396_AIG1	6.266281	7.481354	7.05804	6.687085	7.303119	7.048261
8122600_STXBP5	6.261446	7.813555	7.422958	7.042233	7.842612	7.288908
8123155_PNLDC1	5.123596	4.071095	4.861984	4.483024	4.445169	4.570912
8123388_UNC93A	5.565845	4.526695	4.866881	5.06284	4.982593	4.870337
8123739_NRN1	6.762253	7.847523	8.295834	6.771045	7.946237	8.080154
8124059_NUP153	7.318942	8.365908	8.17075	8.183377	8.460899	8.31181
8124088_KIF13A	8.105442	9.371868	9.03165	8.462902	9.366693	8.98036
8124134_TPMT	4.990481	6.129591	5.439241	5.685436	5.572556	5.527794
8124459_ZNF322A	4.122629	5.488311	5.191846	4.691617	5.667505	5.231616
8124469_GUSBL1	7.867906	9.051355	8.610413	7.988202	9.036979	8.685418
8124848_IER3	7.625978	6.603385	6.806532	7.262069	6.778871	7.010723
8125134_HSPA1L	4.759407	5.923688	5.588833	5.83772	5.64222	5.781522
8125687_RGL2	6.111137	7.122414	6.667237	7.088903	6.970422	7.033868
8126729_CLIC5	7.487636	8.699654	8.559649	7.810144	8.409918	8.291886
8126750_ENPP5	4.734499	6.383633	5.789456	4.476777	6.266809	5.771103
8126820_GPR110	4.844305	3.608327	4.196085	4.054564	3.960994	4.084547
8127177_KLHL31	8.068096	9.412022	8.966299	7.809475	9.424408	8.974433

Supplemental Table 1. Gene expression values changed greater than 2 fold.

Affymetrix ST 1.0 Array ID	0-No	6-No	12-No	0-Yes	6-Yes	12-Yes
8127234_DST	7.951653	9.060785	8.381339	8.513462	9.438925	8.178783
8127370_KHDRBS2	5.655206	4.26235	4.598033	4.349327	4.402682	4.79986
8127544_EEF1A1	5.835104	4.823882	4.472602	5.292025	5.240235	5.321318
8127698_PHIP	6.782376	7.918428	7.236593	6.937795	8.048676	7.041948
8128260_MAP3K7	6.525992	7.614697	7.272408	6.871712	7.469587	6.985231
8128429_CCNC	5.971934	7.048341	6.826067	6.339611	7.059528	6.709306
8128522_HACE1	5.346292	6.608006	6.211674	6.019534	6.748114	6.588284
8128698_SESN1	9.39319	10.40688	10.31411	9.649355	10.47927	10.17042
8128737_MICAL1	6.056374	4.977443	5.419588	5.649246	5.565166	5.959733
8128894_REV3L	6.517652	7.540848	7.008094	6.768833	7.822739	7.201344
8129089_TSPYL4	6.369486	5.307775	6.088963	5.931674	5.786516	5.497161
8129193_C6orf204	5.677263	6.69937	6.324743	5.989922	7.051197	6.164951
8129522_MED23	5.987159	7.244401	7.10684	6.238317	7.327681	7.065165
8129562_CTGF	8.547309	7.189976	7.218923	8.638706	7.2924	7.404238
8129706_HBS1L	6.714205	7.738492	8.018463	7.699719	8.318512	7.933396
8129773_BCLAF1	6.169353	7.795594	7.185576	7.394332	8.384859	7.129713
8129924_TXLNB	9.608315	10.80179	10.18185	9.765584	10.35989	10.41897
8130038_SHPRH	5.795225	6.972381	6.333401	6.177649	7.441634	6.209194
8130129_NUP43	6.265494	7.556811	7.384687	6.958074	7.927023	7.743942
8130474_SERAC1	4.853644	5.979964	5.208063	4.972038	5.4104	5.493155
8130578_SNORA20	6.361917	7.710873	7.244846	6.397029	6.271592	6.396137
8130732_BRP44L	8.224376	9.376442	9.71656	8.267817	9.271824	9.521044
8130783_RNASET2	5.576174	4.407217	4.333714	4.992504	4.556983	4.89631
8131042_FLJ44124	5.87947	7.311105	6.830333	5.653584	6.878315	6.829627
8131479_FLJ20323	6.726971	7.970299	7.600612	7.281249	8.237524	7.609791
8131539_TMEM106B	7.041566	8.232013	7.668234	7.80514	8.786775	8.010508
8131803_IL6	6.572118	4.298875	3.994312	9.20948	4.191224	4.131359
8131957_SNX10	5.573878	3.920352	4.170033	4.889715	4.608502	4.855112
8131965_LOC441204	7.462791	6.356131	6.727833	7.129064	6.611825	6.531936
8132557_AEBP1	7.952814	6.802362	6.780832	7.926982	6.716187	7.066947
8132805_VWC2	8.739104	7.677048	8.175673	8.198353	7.494029	7.690873
8133030_GABPA	5.393458	7.020879	6.038572	6.123301	7.439119	6.333376
8133057_ZNF138	4.886467	5.99654	5.403214	5.256073	6.116019	5.641991
8133062_ZNF273	4.603018	5.952099	5.07998	5.124031	5.873625	5.435163
8133504_GTF2I	7.813828	9.096696	8.742771	8.264627	9.114404	8.689087
8133531_PMS2L5	7.928617	8.928911	9.011749	8.176117	9.031973	8.895606
8133818_PHTF2	8.356012	9.454205	9.021297	8.685949	9.8155	9.18069
8133914_DMTF1	5.913164	7.277571	7.077857	6.521653	7.673731	6.609981
8134091_CLDN12	6.619802	7.853753	7.853384	7.22748	8.19296	7.560413
8134122_AKAP9	6.503879	7.59561	6.845199	6.216575	7.933074	6.596811
8134211_DKFZP564O0523	5.733115	6.763723	6.215754	5.941009	6.752268	6.633314
8134351_PPP1R9A	5.460034	6.698825	5.845644	6.050042	6.271698	5.919605
8134376_ASB4	4.90585	6.568624	6.408032	5.20451	6.441633	5.900629
8134384_DYNC1I1	5.724442	4.578455	5.172214	5.34686	4.983768	5.310528
8135211_MGC35361	5.468709	6.568478	6.560174	5.393265	7.044164	5.705455

Supplemental Table 1. Gene expression values changed greater than 2 fold.

Affymetrix ST 1.0 Array ID	0-No	6-No	12-No	0-Yes	6-Yes	12-Yes
8135268_EIF4B	8.625557	9.83161	9.615417	8.99289	8.733639	9.471325
8135392_HBP1	7.155427	8.202394	8.07707	7.712741	8.262239	7.946437
8135601_MET	6.884096	8.160199	7.81165	7.406304	8.238907	7.61142
8135734_FLJ21986	7.671451	8.930574	8.426361	8.42979	9.254371	8.194258
8135804_ASB15	8.062724	9.654554	9.329199	8.132809	9.84013	9.063518
8136095_KIAA0828	7.199139	8.234118	7.830827	7.597591	8.33504	8.149081
8136115_FAM40B	6.70743	7.993987	7.498043	7.219516	8.04706	7.876025
8136187_CPA2	3.894109	2.831014	3.543851	3.395431	3.228905	3.398639
8136248_MEST	7.418435	6.243347	6.938053	7.215542	6.669452	6.487175
8136259_MKLN1	7.256971	8.341324	8.009836	7.548556	8.465466	7.867759
8136459_AKR1D1	4.716253	3.613229	3.549816	3.782901	3.206397	3.78111
8136863_TMEM139	5.557026	4.391073	4.583426	5.304851	4.819436	5.107726
8136893_CLCN1	7.722303	9.092003	8.315824	7.576267	8.663184	8.697408
8137826_FTSJ2	6.327096	7.382633	7.133376	6.753584	7.188366	7.182657
8138401_SNX13	7.081517	8.139612	7.802521	7.469031	8.404444	7.766634
8138647_C7orf31	5.605037	4.562535	5.273358	5.19546	4.875607	4.807431
8138922_KBTBD2	7.105578	8.29078	7.852577	7.731739	8.456959	7.732247
8139087_SFRP4	7.318918	5.339785	5.958034	7.1378	5.8658	5.608912
8139198_RWDD4A	6.571829	7.728142	7.657247	6.855061	7.821719	7.559988
8139421_H2AFV	7.004468	8.574892	8.636415	7.984791	8.798202	8.494913
8139656_GRB10	7.61785	8.621975	8.155667	7.84149	8.411154	8.512556
8139746_PHKG1	7.136302	8.70495	7.756743	7.706586	8.866852	8.663568
8140386_STYXL1	6.417247	5.392624	6.107973	6.418276	5.88011	5.736869
8140579_CACNA2D1	8.723805	9.886454	9.242003	9.176528	10.18106	9.539576
8140686_SEMA3D	4.264741	2.771269	3.255586	3.449026	3.280978	3.202961
8140915_PEX1	5.911978	6.982216	6.613795	6.49861	7.107292	6.994913
8141305_ZNF394	5.275237	6.545469	5.869216	5.846053	6.459107	6.072197
8142120_PBEF1	7.102202	8.269138	7.819331	8.738053	8.538643	7.769885
8142143_COG5	7.252658	8.479913	8.264143	7.922425	8.534102	8.143095
8142435_PPP1R3A	8.728671	10.11621	9.314102	8.951165	10.32079	9.097429
8142765_LRRC4	5.881876	4.705423	5.029571	5.344662	5.362125	5.348385
8142912_FLJ14803	5.533551	6.712971	6.913592	6.284126	7.019587	6.736141
8143341_JHDM1D	6.0729	7.548458	7.32423	6.582418	7.476354	7.086989
8143605_FAM115A	4.20359	5.719248	5.773544	5.918647	5.357292	5.893949
8143697_ZNF786	4.751276	5.797184	6.00001	5.43913	5.916705	5.674825
8144078_SHH	7.237496	6.05451	6.418669	6.443424	6.406585	6.438324
8144228_FLJ36840	4.4878	5.523992	5.017353	5.225419	5.592026	5.484789
8144378_AGPAT5	6.100016	7.499636	7.104382	6.087545	7.622755	6.920976
8144528_TNKS	6.615809	8.030481	7.437444	6.833491	8.025432	7.647244
8144786_SLC7A2	9.792084	10.84952	10.71434	9.964478	10.89632	10.61051
8144812_PCM1	7.563048	8.818543	8.351455	8.043548	9.116041	7.644001
8144857_NAT1	4.196952	3.184399	3.918333	3.977187	3.62207	3.964973
8144953_XPO7	6.217014	7.63147	7.605519	7.191086	7.712582	7.757663
8145611_FZD3	4.956663	5.968201	5.832057	5.174896	5.642471	5.552116
8145636_HMBOX1	5.918255	7.473936	7.233388	6.520573	7.417648	7.106803

Supplemental Table 1. Gene expression values changed greater than 2 fold.

Affymetrix ST 1.0 Array ID	0-No	6-No	12-No	0-Yes	6-Yes	12-Yes
8145793_SNORD13	11.38072	12.4707	12.31155	11.75008	12.01438	12.0541
8145894_ASH2L	7.925221	8.931328	8.903525	8.233032	9.055394	9.027351
8146092_INDO	4.999466	6.140742	5.798764	5.143192	6.1739	5.323035
8146243_HOOK3	6.342912	7.465659	7.019236	7.011364	7.716807	7.131005
8146669_TRIM55	6.252404	7.260678	6.627548	6.281953	7.01007	6.302814
8146687_ADHFE1	7.235632	8.394508	8.37254	7.688756	8.487473	8.520686
8147344_PPM2C	8.159565	9.795854	9.330275	8.251011	9.521557	9.242833
8147396_INTS8	5.008885	6.187722	5.782897	5.54778	6.460176	5.826613
8147447_PTDSS1	7.023964	8.051926	7.965195	7.546344	7.904619	8.028539
8147580_VPS13B	5.934621	7.408636	6.923435	6.54419	7.449754	6.737899
8147724_ATP6V1C1	5.733825	6.814414	6.297032	6.560572	7.17036	6.62873
8147970_EBAG9	5.878286	7.056326	6.592695	6.482334	6.84386	6.58437
8148029_COLEC10	4.749852	3.485448	3.850238	3.930499	3.778454	3.951524
8148293_NSMCE2	5.652576	6.790345	6.734756	5.483429	6.7707	6.393092
8148317_MYC	8.694508	6.689812	6.684913	9.890925	6.802518	6.759993
8148435_WISP1	5.936086	4.884484	5.818452	5.813045	5.364396	5.567994
8148501_PTP4A3	9.264847	10.4113	10.3955	9.412617	10.24706	10.42361
8149264_PPP1R3B	8.234417	9.340103	9.014269	8.371153	9.383114	9.066372
8149638_DOK2	6.5333	5.410655	6.22994	6.003798	5.875663	5.945907
8149825_STC1	5.764909	4.307015	4.162155	6.078109	4.528508	4.293601
8149927_CLU	9.109477	7.65747	7.969223	8.465372	7.863081	7.845509
8150599_PRKDC	6.893095	7.893908	7.588222	7.365905	7.9064	7.785131
8150757_RB1CC1	6.561322	7.622467	6.948118	6.736716	7.839419	6.839581
8150830_LYPLA1	6.942122	8.18019	7.969148	7.655597	8.126885	8.327352
8150877_SNORD54	6.451601	8.223743	8.517153	6.360693	7.253968	7.656013
8150908_IMPAD1	6.958008	8.084444	7.758389	7.627061	8.03726	7.915195
8151118_VCPIP1	6.678265	7.772243	7.596467	7.388272	8.247336	7.43453
8151423_JPH1	9.282946	10.39875	10.20309	9.609285	10.281	10.22566
8151549_IMPA1	5.505606	6.541055	6.17711	6.251367	6.925828	6.32258
8151788_RBM12B	5.472375	6.611229	6.439793	6.219528	7.057521	6.238677
8151906_GDF6	6.092541	5.06801	5.535874	5.636086	5.818543	5.715831
8151909_UQCRB	4.645125	5.871309	6.111192	5.286585	6.541372	5.971549
8151931_TSPYL5	6.383805	7.390126	7.346466	6.572607	7.629085	7.817308
8152148_UBR5	6.830521	7.978578	7.545638	7.737344	7.951797	7.698967
8152297_ANGPT1	4.704666	6.313934	5.670928	5.109509	7.121245	5.92354
8152477_RAD21	7.816783	8.95214	8.399646	8.078301	9.22672	8.752663
8153474_TSTA3	5.944697	7.234876	7.079995	7.037333	7.216606	7.271859
8154100_VLDLR	7.450233	8.604017	8.244121	7.956874	8.728996	8.128038
8154178_JAK2	6.378777	7.40793	6.674692	7.04397	7.636566	6.555042
8154233_CD274	3.875938	5.354437	5.194388	4.449241	5.373841	5.178218
8154254_KIAA1432	6.336971	7.437074	7.279574	7.011988	7.746197	7.1498
8154733_ACO1	7.237519	8.466347	8.366375	8.346613	8.276342	8.528381
8154793_NFX1	7.110539	8.138	7.887499	7.421609	8.054851	7.937511
8155636_CBWD3	5.78505	6.814363	6.337353	5.991014	7.0899	6.424235
8155696_FAM122A	7.020222	8.078995	8.0431	7.37196	7.863438	8.101976

Supplemental Table 1. Gene expression values changed greater than 2 fold.

Affymetrix ST 1.0 Array ID	0-No	6-No	12-No	0-Yes	6-Yes	12-Yes
8155734_C9orf61	8.082316	9.258396	8.638712	8.466831	9.386167	9.271753
8155946_VPS13A	6.538362	8.058982	7.299399	7.06504	8.439441	7.237871
8156167_MAK10	6.182654	7.418227	7.367805	7.042983	7.735313	7.222026
8156393_SUSD3	6.338364	5.258395	5.389196	6.046624	5.803801	5.818212
8156838_SEC61B	9.262861	8.156082	8.670424	8.957995	8.141078	8.366916
8156848_NR4A3	8.099947	5.349873	5.370732	9.016953	5.362062	5.331358
8156861_STX17	6.538082	7.653284	7.37313	6.621487	7.833505	7.515824
8156919_LOC347273	8.916457	10.27306	9.82144	9.515768	10.37265	9.863779
8156935_ZNF189	5.092442	6.605495	6.095396	5.660814	6.581373	5.625256
8157012_OR13F1	5.209774	3.994806	4.982924	5.229694	4.52593	4.13588
8157233_HSDL2	7.544384	8.910253	8.48737	8.487062	9.404716	8.732855
8157463_C9orf91	5.219922	6.301367	5.902421	5.35323	6.050508	6.24617
8157668_OR1J2	3.928633	2.591573	2.683846	2.683617	2.562438	2.685519
8158446_NUP188	5.920739	7.151964	6.62216	6.696546	7.042836	7.212984
8158597_GPR107	6.147398	7.164312	7.321513	6.756408	6.921718	7.221474
8158708_PRDM12	7.592434	6.559816	6.978212	7.349563	6.736768	6.866393
8158714_EXOSC2	5.733786	7.44382	7.306493	6.430555	7.470125	7.315069
8158829_KIAA0515	8.488101	9.525189	9.263066	9.021456	9.286868	9.391107
8158961_GTF3C5	6.868376	7.882984	7.84309	7.241362	7.617357	7.899595
8159142_COL5A1	7.269841	6.175613	6.526619	7.007052	6.433376	6.818847
8159815_CBWD1	5.800575	6.83128	6.419683	6.077578	7.017373	6.657095
8159876_RFX3	4.520131	5.615184	4.871509	4.721755	5.608671	5.229014
8159992_ERMP1	6.978844	8.111021	7.515096	6.954614	8.204984	7.738609
8160151_ZDHHC21	5.916814	7.310919	6.728726	6.652883	7.497766	6.457064
8160441_CDKN2A	6.481091	5.426286	5.985469	6.365616	5.647759	5.798905
8160622_SMU1	6.92423	8.099306	7.825763	7.245866	8.326677	7.975058
8160889_CCL21	7.363065	5.946089	5.863478	6.419127	5.80718	5.953388
8161114_GBA2	6.595864	7.749135	7.473755	7.14885	7.816844	7.488923
8161238_RAB1C	6.692535	7.857032	7.257784	7.340181	7.701615	7.787528
8161537_CBWD5	5.771312	6.776196	6.208347	5.965875	6.925605	6.442352
8162086_AGTPBP1	5.357381	6.517156	6.619512	6.145862	6.932099	6.216308
8162373_OGN	6.001492	4.869971	5.046668	6.958801	5.187059	5.459828
8162394_ASPN	5.597515	4.545925	4.331217	5.075227	5.268647	5.027607
8162631_ZNF510	5.195486	6.289964	5.631864	5.776556	6.941214	5.991246
8162759_TBC1D2	6.366153	5.224555	5.600101	5.890275	5.770354	5.88498
8162833_TXNDC4	6.51404	7.650526	7.275491	7.218246	7.896512	7.320183
8162884_ALDOB	5.903922	4.715665	4.947146	5.950101	4.552605	5.111025
8163002_KLF4	7.328327	6.011024	6.559502	8.030248	6.353899	5.98921
8163149_PTPN3	6.472445	7.51877	7.539029	6.666482	7.367811	7.050256
8163257_EDG2	7.754435	6.723831	6.313313	7.485677	6.685501	7.164182
8163452_FKBP15	6.466099	7.550499	7.189919	6.963503	7.557964	7.432494
8163637_TNC	6.182385	5.003929	5.036234	5.977787	4.629497	5.16561
8164131_C9orf126	5.915061	7.161107	6.963206	6.348305	7.342322	7.020739
8165808_XG	6.450289	5.23816	5.710917	6.057889	5.745478	5.671051
8165974_CLCN4	7.116666	8.328249	7.882214	7.694927	8.140718	8.277116

Supplemental Table 1. Gene expression values changed greater than 2 fold.

Affymetrix ST 1.0 Array ID	0-No	6-No	12-No	0-Yes	6-Yes	12-Yes
8166195_ZRSR2	6.801735	5.700585	6.774637	6.323314	6.381456	7.087154
8166402_SMS	8.947158	7.861674	7.767984	8.110168	7.639339	7.523503
8166500_ZFX	7.545083	8.645378	8.131107	7.334455	8.710186	8.160983
8166611_MAGEB2	4.261963	2.881147	3.39399	3.585879	3.116137	3.37598
8166797_MID1IP1	8.437222	9.510228	9.254177	8.907105	9.448765	9.468329
8167998_AR	6.546775	7.929833	7.357997	6.629797	7.69876	7.134298
8168087_IGBP1	7.734872	8.877414	8.799232	7.835497	8.97897	8.996602
8168316_OGT	8.328121	9.368648	9.124206	8.360838	9.442541	8.954289
8168691_DIAPH2	6.355092	8.040352	7.349491	6.977191	8.208286	6.848932
8169073_H2BFXP	5.311891	4.202481	4.70752	4.574347	4.163342	4.285904
8169272_ATG4A	5.443149	6.629992	6.156013	6.397095	6.699078	6.638266
8169378_RGAG1	4.798016	3.76971	3.945986	4.03184	4.137863	4.205578
8169750_STAG2	5.896978	6.951427	6.629165	6.225381	7.466179	6.379744
8169811_OCRL	6.059016	7.248147	6.739147	6.454521	6.756535	6.580592
8170166_HTATSF1	6.307568	7.357591	7.092742	7.3953	7.558348	7.041197
8170648_BGN	8.636516	7.408998	7.683683	8.64168	7.281353	8.265059
8170850_EMD	8.073334	9.21446	9.156377	8.534776	8.797562	9.351931
8171013_VBP1	5.462976	6.565973	6.635893	5.704662	6.999725	6.424218
8171136_P2RY8	6.655946	5.468349	5.891702	6.116815	5.534105	5.676452
8171161_ARSE	6.149961	4.899059	4.917818	4.432383	5.081945	5.184046
8171172_MXRA5	6.398549	5.28984	5.194962	5.924463	5.192564	5.552907
8171338_FAM9C	4.759412	3.49989	3.780622	3.655488	3.601745	4.14323
8171403_ASB11	7.069649	8.241236	7.58726	7.63072	8.662724	8.099547
8171587_PHKA2	6.411175	7.48038	7.22261	6.713051	7.257945	7.162852
8171762_RPS6KA3	8.309097	9.490956	8.974232	9.072388	9.748725	9.092162
8171879_LOC389842	6.765097	5.469232	5.828164	5.387452	6.002204	6.871243
8171921_DMD	7.737972	9.334596	8.765933	8.224703	9.378198	8.642843
8172204_MAOB	7.000094	9.08445	8.611274	8.012955	8.795534	8.489779
8172317_SYN1	6.574238	5.40307	5.937096	6.101059	5.71	5.989074
8172333_CFP	7.089631	6.080067	6.342818	6.661684	6.150607	6.760272
8172399_SSX9	4.104739	3.096776	2.614347	2.83278	2.70141	3.340524
8172806_XAGE3	6.42576	5.397232	5.509309	6.259246	5.960557	5.929602
8173120_PFKFB1	7.981479	9.36265	8.830564	8.147625	9.036863	8.857499
8173206_LOC645251	5.547968	6.604308	6.420568	5.985932	6.48375	6.43969
8173551_PHKA1	7.830956	8.863617	8.333265	8.092375	8.71105	8.465305
8173629_ABCB7	5.998772	7.09471	6.836706	6.406979	7.106453	7.015831
8174029_TAF7L	4.446713	3.410763	3.607329	3.687554	3.647126	3.646547
8174047_TIMM8A	3.520013	4.603609	5.104536	5.41674	5.3823	5.140452
8174513_CHRDL1	6.875302	5.804546	6.517712	6.43852	5.997221	6.634108
8174654_KLHL13	4.466241	5.724211	5.383175	5.473354	5.33315	5.471664
8174779_LAMP2	8.426051	9.68358	9.491432	8.88063	9.886269	9.454535
8174893_THOC2	5.550824	6.707089	6.320014	6.349456	7.152169	6.270167
8174979_ACTRT1	4.437368	3.293426	3.434299	3.53158	3.534872	3.365356
8174985_SMARCA1	5.792793	7.422828	6.400282	6.604237	7.594126	6.300362
8175052_AIFM1	6.327483	7.800749	7.33356	6.713911	7.664255	7.763741

Supplemental Table 1. Gene expression values changed greater than 2 fold.

Affymetrix ST 1.0 Array ID	0-No	6-No	12-No	0-Yes	6-Yes	12-Yes
8175234_GPC3	6.054774	4.721335	5.435296	5.190894	5.100519	5.082432
8175311_CXorf48	5.806885	3.713603	4.654748	5.100195	4.146898	4.521109
8175393_ARHGEF6	7.772394	9.126902	8.590351	8.381973	8.970388	8.515032
8175531_CDR1	9.056747	6.546371	5.693292	8.43884	6.643586	7.088392
8176174_MPP1	6.921284	8.033081	8.448286	7.518075	8.308686	8.593336
8176193_F8	6.397482	7.507309	7.108045	7.001032	7.697576	7.265888
8176276_ATRX	4.807721	5.916878	5.88197	3.963777	6.58672	5.746941
8177026_P2RY8	6.655946	5.468349	5.891702	6.116815	5.534105	5.676452
8177277_RBMY2EP	4.80685	3.122865	3.440672	2.462251	3.371255	3.441513
8177620_CARTPT	4.871395	3.795715	4.336544	4.1664	4.13505	4.640756
8177996_AIF1	6.435225	5.408065	6.254071	6.066275	6.066275	6.159676
8178115_CFB	6.509113	5.358462	5.067138	5.454976	5.650531	5.600937
8178435_IER3	7.641111	6.608866	6.872152	7.330108	6.790241	6.985681
8178650_HSPA1L	4.759407	5.923688	5.588833	5.83772	5.64222	5.781522
8178955_RGL2	6.060374	7.158273	6.730458	7.16017	7.142819	7.077695
8179276_AIF1	6.435225	5.408065	6.254071	6.066275	6.066275	6.159676
8179351_CFB	6.655607	4.829484	5.235375	5.400782	5.792297	5.679602
8179704_IER3	7.625978	6.603385	6.806532	7.262069	6.778871	7.010723
8179848_HSPA1L	4.759407	5.923688	5.588833	5.83772	5.64222	5.781522
8180123_VPS52	7.572099	8.608216	8.306978	7.985317	8.626323	8.651555
8180144_RGL2	6.060374	7.158273	6.730458	7.16017	7.142819	7.077695
7897026_CALML6	8.152193	9.091335	9.398197	8.058426	9.18689	9.172965
7898739_CDC42	6.004664	6.413025	5.952457	5.154321	6.527417	6.046221
7899534_EPB41	7.414787	7.504642	7.96962	6.691719	7.722669	7.817941
7900146_ZC3H12A	6.898825	6.421175	6.483186	7.676773	6.628183	6.70946
7900201_UTP11L	8.554737	9.176876	9.176184	8.320325	9.420209	9.145167
7902227_GADD45A	6.622281	5.863658	6.160154	7.257342	5.945126	5.988499
7902382_RABGGTB	7.156011	8.035715	8.10641	7.222857	8.245698	7.945081
7902553_IFI44	4.923809	4.937919	4.764603	4.392751	5.489942	4.779183
7903162_TMEM56	7.8412	8.243263	8.772076	7.460522	8.916162	8.796952
7903171_RWDD3	5.353331	5.977451	6.043171	4.516362	6.554813	5.697886
7903188_PTBP2	6.96575	7.845931	7.949355	6.91269	8.140224	7.497662
7903358_VCAM1	5.116778	4.734388	4.959856	5.760637	4.685266	4.867394
7903407_AMY2B	5.731398	6.298746	6.007257	5.591381	6.608028	6.15407
7903425_AMY1A	6.323844	7.245952	7.099753	6.111895	7.555655	7.197855
7903440_AMY1B	6.323844	7.245952	7.099753	6.111895	7.555655	7.197855
7904254_ATP1A1	8.998251	8.323153	8.284603	9.623279	8.30646	8.39695
7904717_HFE2	9.392252	10.23718	9.591028	8.759354	10.00372	10.30066
7905067_HIST2H4B	5.980163	6.589405	6.723751	5.223533	6.383597	6.297891
7907171_BLZF1	6.207203	6.715867	6.164703	5.953604	6.969339	6.259559
7907271_FMO2	6.981108	7.873906	7.91326	7.739257	8.871772	7.995331
7908917_BTG2	9.341028	8.610548	8.812774	10.22699	8.65327	8.863656
7909064_CNTN2	6.580872	6.048765	6.026844	6.722114	5.684496	6.289979
7910790_LOC440737	7.9651	8.412031	9.080515	7.776387	8.79241	8.698901
7911539_CCNL2	7.218504	7.91626	7.809861	6.971441	7.972708	7.714937

Supplemental Table 1. Gene expression values changed greater than 2 fold.

Affymetrix ST 1.0 Array ID	0-No	6-No	12-No	0-Yes	6-Yes	12-Yes
7913415_USP48	5.826	6.648849	6.364255	5.995434	7.03047	6.523265
7914153_EYA3	6.571922	6.817746	6.673134	6.109143	7.163282	6.721178
7915130_SF3A3	7.396027	8.29556	7.949985	7.69738	8.787166	8.393572
7916018_LOC51336	3.68284	4.577936	4.416787	3.556609	5.244726	4.509143
7916570_OMA1	6.024489	6.846221	6.878729	6.306891	7.410043	6.845392
7916836_SERBP1	6.80423	7.549002	7.244051	6.693858	7.748415	7.340951
7917612_HFM1	3.419714	3.23542	3.309831	2.748428	3.783384	3.266991
7917850_ARHGAP29	6.38816	7.369958	6.736551	6.550017	7.855615	6.900455
7918134_AMY1C	6.323844	7.245952	7.099753	6.111895	7.555655	7.197855
7918467_C1orf103	5.428668	5.096991	5.183505	4.507315	5.71386	5.40341
7918703_AP4B1	6.366158	6.446966	6.695071	5.664837	6.855241	6.688805
7919627_HIST2H4B	5.980163	6.589405	6.723751	5.223533	6.383597	6.297891
7919898_SEMA6C	8.755288	9.607352	9.245964	8.551205	9.722803	9.347976
7919929_TMOD4	9.38632	9.922347	10.08641	8.728051	9.930645	10.2962
7920873_SNORA42	9.00288	9.065637	9.69422	9.829914	7.691227	8.2898
7921533_KCNJ10	4.452769	3.924995	4.220586	5.000891	3.727226	4.350777
7921821_ADAMTS4	6.092053	5.468916	5.466109	8.154138	5.328287	5.662237
7921873_FCGR3B	6.607052	5.628654	7.010302	5.991644	7.484607	7.188114
7922309_VAMP4	5.388207	6.039337	5.995554	5.23127	6.678789	5.175056
7922504_RFWD2	6.042214	6.490494	6.325093	5.485712	6.780627	6.108279
7922976_PTGS2	5.402488	4.79126	4.082756	5.706059	4.687712	4.510971
7923119_ZBTB41	6.198569	6.735434	6.098463	6.078258	7.297683	5.699137
7924450_DUSP10	6.715573	7.41366	7.096631	6.28089	7.452037	7.120045
7926037_PFKFB3	9.947356	9.270608	9.686983	10.25223	9.235668	9.508835
7926345_RPP38	4.72052	5.39484	5.375817	4.662179	5.725928	5.327802
7926356_PTER	6.355128	7.329744	6.950691	6.256567	7.515682	6.622409
7926531_ARL5B	6.982388	6.87427	6.774338	8.28644	7.2617	6.506597
7927062_ZNF33A	5.530521	6.31775	5.763037	5.457585	6.81121	5.855126
7927173_ZNF485	5.881663	5.757712	6.390427	4.906431	6.111262	4.837699
7927552_CTGLF3	7.350686	7.938395	7.664694	7.089853	8.176884	7.783266
7927814_SIRT1	7.271037	7.722795	7.756539	6.986041	7.999484	7.678094
7927876_CXXC6	4.643232	5.240199	4.864877	4.379003	5.50931	4.859926
7928429_PLAU	7.030331	6.478587	6.34606	8.043733	6.537731	6.455809
7928685_DYDC2	4.209547	3.698895	3.313581	4.136786	3.070716	3.575498
7929065_IFIT1	7.674005	7.780722	7.707719	6.779263	7.80843	7.679992
7929373_LGI1	4.766778	4.840004	5.323584	4.662179	5.801134	4.921157
7929533_C10orf130	3.225332	3.688533	2.93876	4.541442	3.438465	3.184115
7929596_LCOR	7.23534	7.857306	7.558492	6.939687	7.9691	7.481678
7929816_SCD	6.044382	5.579355	6.807779	7.409997	4.718183	5.818217
7930413_DUSP5	8.163034	7.316272	7.471637	8.020495	6.861748	7.404844
7930422_SMC3	5.974304	6.528326	6.387903	5.849234	7.316806	6.292204
7931728_LARP5	7.469	8.440556	8.158615	7.441751	8.592041	8.124652
7931810_KLF6	8.582771	7.731908	7.571503	8.967759	7.850451	7.660207
7932637_ANKRD26	4.907272	5.872884	5.372516	4.747432	6.303664	5.360055
7933092_ZNF248	5.097186	5.916775	5.657539	5.009116	6.328335	5.35581

Supplemental Table 1. Gene expression values changed greater than 2 fold.

Affymetrix ST 1.0 Array ID	0-No	6-No	12-No	0-Yes	6-Yes	12-Yes
7933442_PTPN20B // PTPN20A	4.177966	4.209625	4.064323	3.448515	4.550627	3.350817
7933509_ERCC6	5.994595	6.891399	6.30338	5.830495	7.005078	6.224683
7933574_CTGLF1	7.357208	7.936047	7.654691	7.2084	8.250734	7.768196
7933872_EGR2	6.835197	5.918299	5.524773	6.90013	5.553514	5.810235
7933904_CTNNA3	6.389105	7.265659	6.759454	6.009649	7.59711	6.399686
7933999_RUFY2	5.682049	6.411586	6.394936	5.757603	6.805437	6.574325
7934916_CH25H	5.362375	4.79632	4.684476	6.996476	4.429471	4.570127
7935011_CPEB3	7.753865	8.684309	8.448227	7.770659	8.801258	8.420325
7935146_NOC3L	4.498207	5.255158	4.822739	4.650243	5.812672	4.828681
7936408_DCLRE1A	4.923335	4.661222	4.353269	4.270461	5.310816	4.527042
7936463_ABLIM1	8.810948	8.43593	8.197087	9.470741	8.276084	8.258792
7936826_IKZF5	5.708665	6.579107	6.512345	6.218052	7.2897	6.682661
7936891_LOC399818	5.208543	6.091787	5.974781	5.356231	6.784641	6.330112
7938291_SNORA3	9.652998	9.113131	10.94258	10.19795	8.951416	8.995405
7938762_GTF2H1	5.703393	6.265827	5.970345	5.422654	6.730808	6.162092
7939052_FIBIN	5.330533	5.319433	4.977432	6.082245	5.008924	5.62218
7939072_METT5D1	4.383593	5.028514	5.191939	4.451444	5.652883	5.381409
7939093_DPH4	5.583928	6.249557	6.061172	5.360741	6.419671	6.082316
7939102_ELP4	5.906007	6.66502	6.575086	5.721204	6.775105	6.415173
7940749_SLC22A9	4.838638	4.648493	4.571009	5.502231	4.196328	4.495028
7941260_FRMD8	5.853997	6.087616	5.776637	6.651959	5.539001	5.821896
7942613_DGAT2	7.396203	6.421227	7.463029	7.46095	6.397562	6.990983
7942839_PCF11	6.913873	7.586578	7.221118	6.979837	8.05551	6.915564
7943176_MED17	5.920817	6.469022	6.26368	5.786478	6.814424	6.364354
7943218_PANX1	7.292417	7.047228	7.106838	7.83492	6.770603	6.659086
7943314_JRKL	5.692967	5.735781	5.599424	5.123163	6.141218	5.310108
7943620_ATM	5.794928	6.692803	6.480594	5.888551	7.015375	6.063105
7943892_NCAM1	5.962904	5.364404	5.305471	6.402488	5.201703	5.634575
7943998_NNMT	6.574718	5.77677	6.369644	7.635151	6.063268	6.29668
7944335_BLR1	5.085213	4.652834	4.703184	5.319646	4.143177	4.107622
7947052_IGSF22	4.397111	4.375314	4.239827	4.913055	3.835256	4.574669
7947221_LIN7C	5.888504	6.320396	5.859858	5.864125	6.990871	5.939712
7948908_SNORD26	6.565529	7.259034	7.727077	6.086761	7.899773	7.443769
7950606_RSF1	6.269444	6.84093	6.291503	6.482885	7.532981	6.346254
7950933_NOX4	5.79081	5.282859	4.203145	6.318419	5.250762	5.310108
7951046_MRE11A	4.672498	5.061404	4.473657	4.174008	5.663172	4.296946
7951447_CWF19L2	5.326712	5.805456	5.224263	4.947021	6.189849	5.254191
7952205_MCAM	7.278363	6.758305	6.841369	7.721624	6.611685	6.811257
7952268_THY1	6.429905	5.613531	5.614156	6.777379	5.604138	6.030811
7952810_IGSF9B	6.48191	6.987909	6.810725	6.93417	5.922489	7.09444
7953835_KLRG1	4.441884	5.245695	5.075862	4.5737	5.936659	5.680603
7954065_GPRC5A	6.36155	5.389385	5.616767	6.1952	4.857318	5.515994
7954293_PDE3A	5.338704	5.830802	5.604888	5.360944	6.454751	5.78343
7954503_MED21	6.973494	7.64172	7.84896	6.778024	7.83182	7.5028
7954613_CCDC91	6.38816	6.537204	6.63479	6.06672	7.156812	5.698853

Supplemental Table 1. Gene expression values changed greater than 2 fold.

Affymetrix ST 1.0 Array ID	0-No	6-No	12-No	0-Yes	6-Yes	12-Yes
7954711_C12orf35	6.056081	6.910496	6.678195	6.189796	7.3296	6.117613
7954717_BICD1	6.626867	6.164416	6.296556	7.40498	6.162436	5.871035
7954777_ALG10	5.853444	6.453895	6.170269	5.620214	6.653341	6.170562
7954789_ALG10B	5.873663	6.398435	6.5285	5.657919	6.680301	6.153462
7954810_LRRK2	5.355193	5.64399	5.684732	5.394414	6.4646	5.715227
7954969_IRAK4	4.882006	4.840654	4.899752	3.975198	5.127871	4.941847
7955179_TUBA1C	7.216796	6.388549	6.705447	7.008846	5.90421	6.153462
7955217_SPATS2	6.070883	6.481672	6.580322	5.671449	6.690201	6.319522
7956658_SLC16A7	6.83169	6.858929	6.571713	8.152899	7.030044	6.593598
7956785_XPOT	6.406435	7.376847	7.339049	5.939919	7.204089	7.04471
7956856_MSRB3	7.990235	8.392508	8.082217	7.257895	8.542484	8.068798
7957106_CNOT2	6.925188	7.56524	7.338908	7.053248	8.05583	7.522168
7957551_SOCS2	6.238015	5.762853	5.693436	6.668896	5.268067	5.307076
7957715_NEDD1	6.977459	7.91292	7.502645	7.154437	8.181371	7.224232
7959080_SUDS3	7.868002	7.497224	7.893392	6.615625	7.809421	7.479198
7960850_SLC2A14	6.981887	6.33688	6.856121	7.866414	6.347071	6.996161
7961111_CLEC1A	4.971012	4.941342	5.072862	5.811415	4.782253	4.358745
7961287_TAS2R48	4.395087	5.22046	4.575836	3.974895	5.512299	4.818963
7961293_TAS2R46	3.134166	2.875925	2.507621	2.77517	3.993236	2.67976
7962013_ERGIC2	5.293663	6.223836	5.861456	5.53344	6.71821	5.365451
7962146_FAM60A	3.592478	4.538083	2.903563	4.283312	2.789963	3.726039
7962349_GLT8D3	5.149843	5.305053	5.451889	5.025382	6.168826	5.746338
7962499_SFRS2IP	6.649188	7.161268	6.297706	6.581271	7.636079	6.287636
7962559_SLC38A4	6.92247	7.675658	7.681151	6.446972	8.366565	7.445967
7962590_RPAP3	4.486963	5.365316	4.709734	4.524434	5.795438	4.798182
7963555_KRT78	4.927102	4.860437	5.1898	5.81292	4.797853	5.126202
7964575_AVIL	4.649143	4.998307	5.042222	4.102437	5.130723	4.655665
7965064_OSBPL8	5.68122	6.44096	5.976277	6.054815	7.078728	5.842117
7965200_CCDC59	6.672474	6.831343	6.341612	5.796244	7.045736	6.148369
7965436_EEA1	6.35412	7.124113	6.283786	6.735457	7.737712	5.900348
7965541_FGD6	5.129166	5.441231	5.175211	4.324747	5.396474	5.059192
7967304_RSRC2	6.515242	7.186878	7.055951	6.575543	7.699185	6.672359
7967318_GPR109A	6.193999	5.861396	5.957424	6.544528	5.390644	6.166941
7967386_MPHOSPH9	5.248819	6.209266	5.964244	5.359987	6.655895	5.978375
7967863_ZNF605	4.064992	4.97833	4.509552	4.22745	5.374048	5.014342
7968052_C1QTNF9	5.501051	5.631719	4.984756	4.543185	5.748367	5.690351
7968746_WBP4	5.96	6.725662	6.507565	5.870697	6.911274	6.134294
7969428_UCHL3	8.11092	8.50048	8.499769	7.748494	8.773134	8.594636
7969881_TPP2	7.68777	8.385008	8.232792	7.672071	8.728662	7.98217
7971222_MTRF1	5.496001	6.342607	5.660562	5.059959	6.251507	5.958781
7972157_EDNRB	4.472866	4.084455	5.083353	5.588526	4.316837	4.932094
7972180_C13orf7	4.746816	5.695291	5.710763	5.139486	6.272325	5.522749
7972723_FLJ10154	7.486104	8.354237	8.428909	7.402554	8.680917	8.085188
7972899_DKFZp451A211	7.463364	7.061551	7.191297	8.033663	7.024366	7.178371
7973036_PARP2	6.537861	7.114309	7.488097	6.468822	7.5283	7.050715

Supplemental Table 1. Gene expression values changed greater than 2 fold.

Affymetrix ST 1.0 Array ID	0-No	6-No	12-No	0-Yes	6-Yes	12-Yes
7973067_NP	5.77962	5.369566	5.571206	6.90731	4.915293	5.91824
7973770_SCFD1	5.929366	6.655959	6.612929	5.687469	7.25411	6.079989
7974125_KIAA0423	6.216213	7.021512	6.448408	6.232624	7.392849	6.66341
7974146_PRPF39	5.513995	6.402297	6.406373	5.688442	7.007567	6.006162
7974303_TXNDC1	7.219657	7.635248	7.617155	6.902964	8.148829	7.814507
7974380_PSMC6	6.390464	7.079542	7.425589	6.466965	7.524734	6.986589
7974483_KTN1	7.365467	8.166409	7.464489	7.404335	8.728251	6.966762
7974621_ARID4A	5.322218	5.348656	4.767836	4.617639	5.754984	4.909961
7974653_KIAA0586	5.457048	6.180677	6.131997	5.604699	6.631759	5.830672
7974799_MNAT1	6.898129	7.592512	7.462682	6.661031	7.842912	7.199176
7974870_SNAPC1	6.806753	7.420921	7.057476	6.634449	7.710126	6.90902
7975613_ACOT6	4.189488	4.251012	4.116467	4.885631	3.850309	4.064238
7976073_FLRT2	5.356137	6.336915	5.966732	5.189597	6.272489	5.958879
7976496_SERPINA3	5.078135	4.80543	4.483566	6.607184	4.854179	5.023592
7976506_SERPINA13	5.01697	4.37638	4.687955	5.602654	4.598534	5.023304
7976567_BDKRB1	5.066775	4.962127	4.436341	5.373732	4.337348	4.705741
7976621_VRK1	4.713865	4.737667	5.219819	3.930127	5.201038	5.196871
7976812_SNORD113-4	4.634259	4.36777	4.378839	5.414484	4.255038	4.157825
7976814_SNORD114-2	3.748072	3.984381	3.056365	4.662179	3.105388	3.572671
7977497_CCNB1IP1	5.871912	6.519327	6.685045	5.672452	7.06165	6.391709
7978542_KIAA1143	7.408725	7.678312	8.007551	6.963326	8.191498	7.432032
7978570_SNX6	6.994869	7.714734	7.756336	6.943944	7.972959	7.373838
7978666_MBIP	5.619831	6.428629	6.797475	6.10716	7.213635	6.562157
7978833_RPL36AL	8.80632	9.075143	9.342921	8.540663	9.605072	9.412991
7978838_C14orf104	5.335352	5.332977	5.651241	4.61746	5.920904	5.580866
7979813_ZFP36L1	9.575882	8.957545	9.119352	9.88635	8.843141	8.809175
7979931_MED6	7.296537	7.831107	7.995339	6.912055	7.95484	7.490279
7980454_ALKBH1	5.58689	6.56299	6.056524	5.227656	6.384041	6.180948
7980940_ATXN3	6.114518	6.369247	6.001165	5.377948	6.69003	6.006999
7981242_BCL11B	6.297886	5.962691	6.042214	6.586991	5.447918	6.220537
7981781_OR4M2	4.23323	4.843011	4.588131	4.693269	3.67776	3.705863
7981943_SNRPN // PAR5 // SNO	6.552644	7.295289	7.156291	6.274935	7.529343	7.09976
7981992_SNORD116-22	3.241718	4.028347	4.035602	3.260105	4.688825	3.488123
7982002_SNORD116-27	5.185498	5.117593	4.726673	4.001038	5.436335	4.871587
7983979_C15orf31	5.627121	5.296263	5.518831	6.103601	4.990417	4.973499
7984259_RNU5D	6.916576	6.709395	7.487017	5.445184	7.265733	5.787771
7984985_GOLGA6	4.964591	5.102694	5.785944	6.019182	4.55168	5.85184
7987369_ATPBD4	5.548385	5.948078	5.670942	5.199046	6.560681	5.963283
7987642_NDUFAF1	6.391379	7.378201	7.330001	6.632253	7.695073	7.581832
7988970_KIAA1370	7.734658	8.132843	8.076484	7.601357	8.609504	8.028332
7989094_NEDD4	7.77731	8.342404	7.921033	7.632649	8.779131	7.676938
7989132_RFXDC2	7.094381	7.814415	7.28688	7.104489	8.140629	7.489253
7989473_NLF2	7.490628	6.635306	6.879746	7.702562	6.598454	6.695362
7991313_PLIN	7.447248	6.613165	7.69524	8.043631	6.452609	7.003381
7992897_ZNF174	5.200764	5.804005	5.855838	4.864692	5.930981	6.067108

Supplemental Table 1. Gene expression values changed greater than 2 fold.

Affymetrix ST 1.0 Array ID	0-No	6-No	12-No	0-Yes	6-Yes	12-Yes
7993624_SYT17	5.896366	5.559457	5.208344	6.489833	5.425219	5.874253
7994280_IL4R	6.408434	6.142112	5.935438	6.991211	5.892741	6.047727
7995479_PAPD5	5.855509	5.8862	5.762421	5.233492	6.330171	5.562077
7995668_IRX5	7.54869	6.686674	6.488531	7.300159	6.276651	6.648299
7995806_MT1A	8.134527	7.166582	7.689427	9.871501	7.3113	7.394695
7995825_MT1F	7.291041	6.429585	6.55338	7.256622	6.093341	6.309164
7996027_CX3CL1	7.571125	7.341525	7.330677	8.288071	7.069835	7.340858
7997642_CRISPLD2	6.906099	6.474281	6.365359	7.757108	6.720837	6.436791
8001185_DNAJA2	7.273144	7.932283	8.097757	7.056385	8.08008	7.653996
8001531_MT1G	8.474024	7.783312	8.047473	8.839104	7.391099	8.081059
8001800_CDH11	5.962141	5.649124	5.437774	6.211666	5.169072	5.741329
8001918_RRAD	8.311882	7.906373	7.920519	9.273832	7.777658	8.51642
8004081_ZFP3	6.035765	6.594556	6.336101	5.630188	6.761223	6.2385
8005200_SNORD49B	3.702557	3.299675	3.791969	3.231433	4.569963	3.973458
8005501_FAM18B	6.522863	7.166111	6.901414	6.1438	7.414295	6.679193
8006336_LRRC37B	5.836004	6.017201	6.063712	5.434359	6.542821	6.19016
8006367_RHBDL3	6.065167	5.703656	5.006821	6.298639	5.20755	5.844083
8006453_CCL8	5.000767	4.197549	4.356054	5.721747	3.89703	4.144383
8008566_STXBP4	4.673792	5.381659	4.89104	4.491749	5.526839	4.964548
8008784_PRR11	5.661027	4.857111	4.542514	5.848602	4.68525	4.599825
8009255_CCDC45	6.423891	7.226382	7.10876	6.205526	7.318441	6.673783
8009366_NOL11	5.684227	6.215628	6.408137	5.396224	6.425612	6.162578
8009721_CDR2L	6.912588	6.086875	6.410953	6.958592	5.911201	5.594455
8013112_RASD1	8.194281	7.426198	7.301435	8.781021	6.953073	7.306076
8014956_NR1D1	9.058924	9.695639	9.623031	8.779508	9.78028	9.757139
8015456_KLHL11	6.391909	6.317278	5.645422	4.932008	6.176874	5.830837
8015730_FLJ31222	5.242404	5.715431	5.710552	6.041866	4.987336	5.456636
8016077_GPATCH8	7.223398	8.07195	8.085639	7.038025	8.475915	7.963592
8017162_LOC51136	6.534857	6.999554	7.358741	6.273539	7.419427	7.090427
8017262_BRIP1	4.035444	3.719678	4.300127	4.7019	3.237273	3.976098
8018006_ABCA10	5.288284	5.637759	5.448733	4.908504	6.053736	5.451513
8019643_DOC2B	6.154332	5.400859	5.377302	6.914468	5.344668	5.907296
8020267_CEP192	6.016926	6.907189	6.487206	5.834618	7.066233	6.605185
8020323_RNMT	5.626904	6.203956	5.668696	5.480448	6.567876	5.895326
8020930_ELP2	6.225774	7.203738	7.161121	6.135527	7.262906	6.812903
8020973_FHOD3	6.861857	6.962058	6.620278	7.498429	6.434754	6.617198
8021015_PIK3C3	6.464994	7.259999	6.952221	6.496952	7.499976	6.926928
8022441_ROCK1	5.64765	6.237731	5.243934	5.794887	6.887471	5.750322
8022488_ABHD3	5.512139	6.087174	6.2469	5.178052	6.771128	5.952383
8023259_SNORD58A	5.153326	5.740492	5.146007	4.268449	5.308421	4.906927
8023528_ALPK2	8.218493	8.679356	8.577384	8.31454	9.552545	8.831871
8024228_MIDN	8.810616	8.389772	8.496732	9.62642	8.286409	8.460596
8024572_GNA15	6.273389	6.005662	5.74837	6.298036	5.29691	5.138374
8025601_ICAM1	6.756937	6.393631	6.274061	8.733776	6.475596	6.775353
8025828_LDLR	7.551499	6.668352	6.744151	8.694988	6.803265	6.47048

Supplemental Table 1. Gene expression values changed greater than 2 fold.

Affymetrix ST 1.0 Array ID	0-No	6-No	12-No	0-Yes	6-Yes	12-Yes
8025927_ZNF627	5.47074	5.667933	5.218108	4.642818	5.80592	5.549728
8026163_IER2	7.74293	7.360237	7.535421	8.339903	7.32525	7.044253
8026579_TMEM38A	10.04646	10.93415	10.94217	9.908434	10.93127	11.23637
8026861_B3GNT3	6.020432	5.806731	5.758108	6.481091	5.296967	6.175915
8027233_ZNF101	6.543463	6.649188	6.676479	6.923058	5.647665	6.556122
8027241_ZNF253	5.779227	6.260225	6.174496	5.664509	6.95103	6.011669
8027304_ZNF493	5.006991	5.445029	4.879396	4.461913	5.668647	4.865805
8027674_ZNF302	6.908672	7.649586	7.345634	6.783878	8.197224	7.408141
8028219_ZNF420	5.110928	5.315402	5.538241	4.629788	5.969016	5.264406
8028908_ITPKC	6.223467	6.434608	5.901772	7.424373	5.967576	6.05792
8029580_RELB	6.425098	5.881397	5.893118	6.866549	5.658892	5.965367
8030128_PPP1R15A	7.432551	7.154934	7.299891	8.472571	7.185896	7.347057
8031632_ZNF542	4.445482	5.220755	5.334051	4.669377	5.672685	5.581758
8031720_ZNF543	5.33792	6.337801	6.014733	5.500371	6.725318	6.085863
8031784_ZNF134	5.943614	6.411483	6.524386	5.586033	6.8853	6.851249
8031821_ZNF586	5.79676	6.227017	6.251241	5.194202	6.206037	6.077247
8034401_ZNF564	5.470595	5.951558	5.884582	4.901353	6.182156	5.44847
8034772_ASF1B	5.627658	5.284615	5.230217	5.557384	4.422168	5.311547
8035517_COMP	6.303041	5.410326	5.471364	6.22463	5.12492	5.590167
8035714_GMIP	5.783381	6.22344	6.396676	6.376858	5.33277	6.034993
8036591_LGALS4	6.980175	6.605281	6.533762	6.995736	5.617449	6.402771
8036813_ZNF780B	6.145348	6.995065	6.385077	5.224241	7.400749	6.154045
8037614_GPR4	6.254679	5.761162	5.624958	6.667253	5.523336	5.876416
8039909_CCNL2	7.071517	7.677258	7.664901	6.779291	7.79415	7.618668
8041027_MRPL33	7.786536	7.702473	8.204244	7.034052	8.278955	7.895533
8041048_FOSL2	8.470719	7.929735	7.984932	9.077315	7.902207	8.090069
8041257_SLC30A6	7.139644	7.390976	7.686452	6.835895	7.878732	7.410925
8041360_TTC27	6.245105	6.413755	6.308055	5.857306	6.8757	6.498089
8042119_PAPOLG	6.131064	6.380843	6.560234	5.880207	7.250526	6.61624
8042532_VAX2	7.767608	7.128196	7.222275	8.00861	6.795717	7.128298
8042761_ALMS1L	5.044689	4.994806	5.050975	5.724151	4.594123	4.636821
8042942_HK2	7.599497	6.76346	7.208726	8.400105	6.715323	7.725948
8043349_ANAPC1	5.939003	6.702729	6.63098	5.980493	7.007926	6.906888
8043621_ARID5A	7.10373	6.505413	6.398689	7.545863	6.231705	6.339054
8043687_ANKRD36	5.002801	5.537373	5.647285	4.681329	6.04239	4.808879
8043995_IL1R1	5.496561	5.440635	5.213369	6.843686	5.672685	4.988549
8044149_PLGLA1	3.547542	4.033266	3.723472	3.289842	4.402149	4.028977
8044766_INSIG2	6.430222	6.660116	6.378501	6.139314	7.197495	6.35269
8044965_TSN	6.996801	7.733341	7.692523	6.902847	7.909719	7.693755
8045381_CCNT2	6.358741	7.091759	6.48525	6.32753	7.372637	6.773734
8046099_NOSTRIN	4.526066	4.556555	4.67073	4.266641	5.364693	4.56409
8046527_HOXD12	5.596222	4.696997	4.205812	5.251592	4.229211	4.782871
8046573_MTX2	6.248557	7.075703	7.16339	5.971217	7.043046	6.908975
8046975_WDR75	5.608856	6.427127	6.281699	5.521851	6.722103	5.736956
8047356_NIF3L1	5.308913	5.844364	6.139898	4.87759	6.158083	5.7965

Supplemental Table 1. Gene expression values changed greater than 2 fold.

Affymetrix ST 1.0 Array ID	0-No	6-No	12-No	0-Yes	6-Yes	12-Yes
8047815_FASTKD2	6.109234	7.046725	6.865636	6.06566	7.24876	6.646726
8047839_CREB1	7.272737	8.073399	7.943409	7.294182	8.374461	7.721989
8048227_IL8RB	4.734203	3.785798	5.326231	4.017436	5.988665	5.119635
8049128_ALPPL2	7.013398	6.240889	6.442796	7.209816	6.200397	6.373952
8050007_PXDN	6.278726	6.236992	6.498399	7.251482	6.171561	6.487393
8050079_RNASEH1	5.714371	6.480021	6.586093	5.767402	6.806007	6.358584
8051928_PREPL	7.666707	8.353103	8.213918	7.528149	8.774376	7.953786
8051993_PIGF	5.484868	4.699355	5.572602	4.837504	5.915051	5.623684
8052669_SERTAD2	7.037287	7.094885	7.180851	6.032744	7.052198	6.890666
8052703_WDR92	5.469613	6.353087	6.356287	5.210839	6.441633	6.289135
8052845_TIA1	7.408223	8.112767	7.765201	7.294081	8.738783	7.886106
8053315_LRRTM4	5.214976	5.723748	5.704834	4.876786	6.26835	5.389774
8054064_KIAA1641	4.719867	5.481626	5.151431	4.991398	6.411672	5.062582
8054872_TFCP2L1	5.479109	5.745264	5.637254	6.091872	4.878291	5.498482
8055688_RND3	6.193079	5.228761	5.745604	7.147457	5.893889	5.316192
8055913_PRPF40A	6.435078	7.147074	6.686885	6.461572	7.760719	6.925859
8056047_WDSUB1	4.603125	5.455441	5.526279	4.477334	5.482139	5.423763
8056426_TTC21B	5.559098	6.234139	5.975238	5.527745	6.769835	5.918147
8056518_SCN7A	6.139905	6.445835	6.160235	5.73989	7.210375	6.016738
8057700_ORMDL1	5.764826	6.574733	6.703988	5.240988	6.775662	6.230049
8058075_BOLL	4.803003	5.714355	6.207527	5.021906	6.258482	5.465266
8058127_CLK1	8.105714	8.61231	8.966955	7.701914	9.200132	8.663393
8058905_IL8RA	4.703045	3.958981	5.091224	4.583003	5.788462	5.321652
8059301_PAX3	5.425683	5.828706	5.721246	5.049613	6.334122	5.490933
8059376_SERPINE2	5.52929	5.002745	5.472477	5.814755	4.498709	4.74691
8061019_C20orf7	4.366319	5.36428	5.660289	4.322281	5.45981	5.13648
8061075_SNRPB2	7.108594	7.862692	7.780512	6.904383	7.973303	7.622281
8063410_PARD6B	6.597396	6.815472	6.977064	8.159584	6.884686	7.243107
8063566_RAE1	6.312314	7.045189	7.096296	6.034194	7.154872	7.08386
8064514_ZNF343	5.265014	5.241301	5.854815	4.939198	6.03978	5.548637
8064967_MKKS	5.6529	6.044288	5.896419	5.602732	6.829378	6.145031
8065547_FLJ45832	3.858702	3.407524	3.419792	3.928249	2.816501	3.112474
8066051_NDRG3	7.052482	7.786852	7.732804	6.838991	7.939584	7.948862
8066921_KCNB1	6.286173	5.957303	6.263881	7.176013	5.882979	6.12328
8067626_KCNQ2	5.097304	5.42061	4.924364	5.868384	4.735555	5.481342
8068062_USP16	5.555372	6.441764	6.193116	5.981042	7.331495	5.737392
8068460_MORC3	6.594214	7.392251	7.023242	6.842216	7.974904	7.10202
8069150_KRTAP10-9	7.132271	6.805212	6.392862	6.792851	5.672685	6.676139
8069700_N6AMT1	5.23975	5.889246	6.091692	4.922981	6.214132	5.699036
8069744_RWDD2B	7.066228	8.024041	7.669775	6.699544	7.852769	7.518042
8069872_KRTAP11-1	4.880751	4.588162	4.310996	4.936552	3.844613	4.327746
8070046_C21orf66	6.042727	6.681691	6.516805	5.85403	7.021712	5.811992
8070341_BRWD1	6.373736	6.982735	6.786927	6.477274	7.573169	6.726531
8070389_HMGN1	6.2499	6.873999	7.941305	7.929346	6.582352	6.801953
8071061_psiTPTE22	6.221937	6.255491	6.244121	5.589451	6.670854	5.604191

Supplemental Table 1. Gene expression values changed greater than 2 fold.

Affymetrix ST 1.0 Array ID	0-No	6-No	12-No	0-Yes	6-Yes	12-Yes
8073775_FBLN1	7.394075	6.559968	6.512765	7.556495	6.515767	6.903612
8079005_ZNF621	6.021571	6.017804	6.021165	5.2395	6.300144	5.500315
8080578_CACNA2D3	5.778647	6.687343	6.621276	5.513354	6.686843	6.212689
8080621_CCDC66	4.84727	5.334098	4.978273	4.131388	5.530372	4.695087
8080676_2'-PDE	5.591379	6.49186	6.056892	5.68581	6.689994	6.636101
8080973_PPP4R2	6.266857	7.131832	6.544922	6.238827	7.273933	6.594085
8081036_CADM2	6.19688	6.060763	5.474549	6.610266	5.402398	5.158232
8081069_ZNF654	4.766128	5.202999	5.222582	5.105861	6.116508	4.805696
8081128_NSUN3	4.481078	5.04941	5.026344	4.450498	5.508549	4.600316
8081277_NIT2	6.652147	7.316514	7.405485	6.492289	7.574148	7.589704
8081386_NFKBIZ	6.344573	5.737919	5.771874	7.635133	5.760401	5.705921
8081667_SLC35A5	7.10087	7.467793	7.641242	6.93427	8.034969	7.239484
8082574_TRH	5.475595	5.395922	5.112262	5.886755	4.6415	5.142743
8082940_ARMC8	6.670106	7.363121	7.139409	6.564472	7.642257	6.974562
8083136_ATP1B3	5.241348	5.729908	5.554811	6.863042	5.492335	5.411848
8083233_ZIC1	5.644571	5.359408	5.860908	5.973117	4.90504	5.443841
8083569_TIPARP	7.39502	6.841975	7.002641	8.548352	7.32565	6.952506
8083594_PTX3	4.200305	4.318518	4.054603	5.28578	4.240737	4.587265
8084016_PIK3CA	7.063891	7.631048	6.859066	6.962709	7.96519	6.723192
8084128_TTC14	5.452095	6.134187	5.854671	5.580698	6.687036	5.757251
8084206_B3GNT5	5.200742	5.2751	4.990438	7.003935	5.581347	4.751616
8084838_HRASLS	6.654475	7.61895	8.048309	6.370296	7.966478	8.157236
8084880_HES1	8.403553	7.901287	8.063848	9.050896	7.37599	7.593325
8085350_C3orf31	6.042309	6.640252	6.418028	5.771065	6.788135	6.870858
8086451_HIGD1A	7.070109	7.506381	7.664065	6.526846	7.784512	7.625649
8086498_KIAA1143	6.585075	6.847677	7.112456	6.256474	7.261042	6.590009
8087201_IHPK2	6.223319	6.334085	6.767468	5.540013	6.601696	6.568013
8088092_RFT1	5.873746	6.342396	6.200418	5.446739	6.721725	6.229885
8088415_FAM107A	7.72601	7.642819	7.688279	8.579496	7.577313	7.506717
8088526_THOC7	6.669893	7.085371	7.72737	6.546611	7.613668	7.583902
8088560_ADAMTS9	6.853329	6.748654	6.497414	7.978644	6.813457	6.604665
8089584_C3orf17	6.839649	7.241293	7.112703	6.27714	7.360851	6.888353
8089647_KIAA2018	7.378834	8.374307	8.081225	7.414236	8.756465	8.180788
8089785_POPDC2	6.332523	6.962102	7.031672	6.4679	7.529953	6.88473
8090533_ISY1	5.602036	6.10769	5.996323	5.387183	6.391367	5.841772
8090893_MSL2L1	8.918641	9.388599	9.218991	8.502157	9.522076	9.291783
8091658_CCNL1	7.666819	7.197639	7.307273	8.924835	7.67148	7.112856
8092169_TNFSF10	7.366191	8.340396	8.234653	7.616798	8.643657	8.197161
8092409_PARL	8.401986	8.730353	8.510428	7.46861	8.543848	8.36729
8092621_CRYGS	5.38854	5.168546	5.613489	4.684809	5.750659	4.982786
8092970_APOD	10.37602	10.62864	10.71219	9.955625	11.22382	11.09551
8094226_LOC285550	4.802329	4.626975	4.936278	4.092031	5.100521	4.313834
8094778_UCHL1	6.293571	6.22767	6.16283	7.030599	5.663981	6.168819
8094974_OCIAD1	6.99969	7.721993	7.521798	6.628745	7.809649	7.511106
8095230_SRP72	7.015239	7.477388	7.764369	6.901135	7.95557	7.361471

Supplemental Table 1. Gene expression values changed greater than 2 fold.

Affymetrix ST 1.0 Array ID	0-No	6-No	12-No	0-Yes	6-Yes	12-Yes
8095680_IL8	5.897976	5.341805	5.117891	7.110512	5.090364	5.459977
8095697_CXCL1	6.882879	6.280163	6.153733	7.616308	6.280569	6.32868
8095736_AREG	5.237539	4.50389	4.559739	5.396408	4.242323	4.802057
8095744_AREG	3.596423	2.97147	3.36559	4.23433	2.978384	3.336203
8096301_SPP1	5.299783	4.882556	4.867674	5.850916	4.812614	5.027607
8096463_SMARCAD1	5.150445	6.111082	5.850214	5.359186	6.362761	5.673765
8096938_LARP7	5.088596	5.480585	5.253475	5.038642	6.046803	4.827752
8097529_ELMOD2	4.749209	5.027384	4.621294	4.61726	5.796981	4.695506
8097813_ARFIP1	6.47215	6.50422	6.614151	5.850567	6.884863	6.628923
8098291_CLCN3	6.698949	7.556527	7.430383	6.574879	7.867801	7.35214
8098348_KIAA1712	5.184462	5.23599	5.242013	4.310341	5.5587	5.284199
8099364_KIAA1729	6.004385	6.513444	6.146525	5.820473	6.970773	6.211528
8099696_SEPSECS	5.338027	5.995123	5.645462	4.84096	6.422362	6.046096
8099897_UGDH	6.062028	5.853934	5.910314	6.899716	5.888492	5.966691
8100292_FRYL	5.999997	6.990326	6.584779	6.274367	7.469862	6.837723
8100308_LOC728220	5.389519	5.137042	5.094781	5.794542	4.745248	5.639708
8101228_CNOT6L	6.103359	6.962654	5.854233	6.118088	7.245296	6.201701
8101701_PPM1K	5.41603	6.137357	5.633277	5.144809	6.363319	5.515977
8102200_DKK2	5.782497	5.227818	5.17064	6.453407	5.36401	5.554286
8102560_MAD2L1	3.715725	4.316423	4.195731	3.787912	5.024328	4.275397
8103622_CBR4	6.560927	6.932903	6.643235	6.237391	7.271927	6.326067
8103998_C4orf20	5.376765	6.219049	6.280307	5.189027	6.727986	6.213104
8104601_BASP1	7.666692	6.781887	7.85709	8.366562	7.180025	7.257499
8105633_PPWD1	5.797003	6.417853	6.580823	5.622448	6.8361	6.037576
8105878_RAD17	5.323188	6.21205	5.911152	5.502999	6.55752	5.756289
8106025_BDP1	6.282362	6.944879	6.665991	6.279596	7.474618	6.571197
8106141_FCHO2	6.294967	6.845754	6.690666	5.533385	7.41406	6.657402
8106448_PDE8B	4.887249	4.435551	4.726378	5.478701	4.22977	5.106577
8107044_LRAP	5.477491	5.935538	5.534925	5.473118	6.79714	6.014737
8107282_WDR36	6.207955	6.693766	6.737865	6.11307	7.201761	6.621886
8107474_DMXL1	6.123146	6.880717	6.671004	6.054945	7.137609	6.178956
8107691_RNUXA	5.46116	6.372211	6.182474	5.690076	6.835766	5.506901
8107706_LMNB1	6.13572	5.221156	5.325433	6.079172	5.028015	5.468754
8107859_LYRM7	4.582531	5.18406	5.368498	4.506826	5.832747	5.043659
8107942_RAD50	6.115731	6.609455	6.183208	6.217207	7.321406	5.648183
8108251_NPY6R	7.745355	7.304367	6.908123	8.187067	6.47225	6.838635
8109597_UBLCP1	5.603491	5.929905	5.526742	4.892032	6.409412	4.981955
8109697_CCNG1	7.809577	8.794794	8.667938	7.875332	8.926016	8.388552
8110486_DKFZp686E2433	5.994411	5.484225	5.595083	4.905754	6.054853	5.650212
8110982_DAP	6.43127	5.942994	5.993623	6.808645	5.79562	6.275637
8111677_LIFR	8.056355	9.030615	8.914946	8.446295	9.449327	8.575692
8111788_TTC33	6.481242	7.330452	6.558181	6.442965	7.463181	7.173575
8112538_GTF2H2	6.063915	6.41286	6.549732	5.621618	6.672122	6.373585
8112890_MED27	6.175272	7.021925	7.04611	5.896173	7.188538	6.723395
8113023_TMEM161B	6.229917	7.102778	6.942963	6.369616	7.381197	6.789518

Supplemental Table 1. Gene expression values changed greater than 2 fold.

Affymetrix ST 1.0 Array ID	0-No	6-No	12-No	0-Yes	6-Yes	12-Yes
8113220_ELL2	6.970615	6.679666	6.802038	7.937452	6.937141	6.1894
8113250_ARTS-1	6.788386	7.335756	7.178823	6.420181	7.541391	7.221108
8113286_RIOK2	5.297032	6.06286	5.844554	5.77079	6.827473	5.739905
8113413_NUDT12	5.571743	6.430638	6.265285	5.647178	6.687792	5.983084
8113504_C5orf13	8.104685	9.036089	8.816411	7.753972	9.103136	9.036602
8113691_DTWD2	7.010067	7.226744	7.7398	6.461679	7.906604	7.255784
8114006_FLJ44796	4.491947	5.196334	5.134911	4.755338	5.954538	4.835779
8114010_IRF1	6.300766	5.886288	6.157104	7.658031	6.368894	6.171364
8115666_NUDCD2	4.865745	4.489499	4.96153	4.121002	5.121617	4.418805
8116418_GFPT2	5.93839	5.588525	5.843113	7.032134	5.58645	6.417137
8116835_GCNT2	4.347201	5.249016	4.809304	4.189675	5.260234	5.116117
8117415_HIST1H3E	3.444874	3.903794	3.953242	3.047923	4.558733	4.145775
8118322_SNORD52	4.489374	5.00911	5.348427	4.692696	5.720782	5.172241
8119088_CDKN1A	7.242183	6.294436	6.662832	8.00008	5.973511	6.232096
8119161_PIM1	7.858479	7.023489	7.602949	8.382114	7.072377	7.448038
8120067_SLC25A27	5.532509	6.200448	6.127673	5.305754	6.711866	5.487402
8120251_FBXO9	6.947886	7.673402	7.297975	6.647829	7.73237	7.451775
8120335_FAM83B	4.805469	5.482786	4.837413	4.593034	6.264433	4.930855
8120378_KIAA1586	5.243703	5.53027	5.461523	4.673175	6.183015	5.215927
8120382_ZNF451	5.546594	6.493583	6.485806	5.955666	7.0248	5.961846
8120698_MTO1	5.596975	6.525888	6.771567	5.446654	6.628164	6.726798
8121002_C6orf162	6.209558	7.142319	7.424438	6.02125	7.334839	6.705061
8121257_PRDM1	5.318654	6.107738	5.766427	6.663404	5.343396	5.307314
8121461_CDC40	6.690045	7.670028	7.115369	6.600825	7.991076	7.387078
8121510_BXDC1	6.129562	6.862947	6.671585	5.746011	7.122515	5.889754
8121525_KIAA1919	6.678638	7.361337	7.304497	6.148608	7.204514	6.941613
8121601_FAM26E	4.865508	5.41893	5.12204	4.696376	5.747111	5.30837
8121632_KPNA5	5.147379	6.076996	5.790211	5.476275	6.524368	5.529976
8121672_VGLL2	9.350912	8.594666	8.644333	9.819607	8.732953	9.088783
8121749_GJA1	7.111432	6.998941	6.836588	7.841917	6.807055	6.893085
8121895_TRMT11	5.691919	6.476841	5.804438	5.562812	6.643711	6.067212
8122265_TNFAIP3	5.849085	5.521491	5.580415	7.643896	5.566999	5.484686
8122409_PEX3	5.340396	5.945091	6.203043	5.389677	6.529294	5.944923
8122457_STX11	5.837901	4.912858	5.72539	6.599525	5.383831	5.866024
8122807_AKAP12	7.531615	7.21714	6.948421	8.619675	7.218259	6.856564
8122860_MYCT1	5.712215	6.584569	6.77501	5.662272	6.672791	6.842846
8123609_SERPINB9	6.028468	5.680179	6.117712	6.826595	5.758276	5.961211
8123825_SLC35B3	5.584004	5.821541	5.53486	3.939717	5.924446	4.864501
8123936_NEDD9	7.126325	6.685246	6.567173	7.920323	6.904467	6.628185
8123961_TBC1D7	5.38755	5.98504	5.938588	5.184561	6.195946	5.87362
8124129_NHLRC1	4.288398	4.937427	4.669401	4.097873	5.243657	4.769424
8124280_C6orf32	5.88106	6.589921	6.91415	5.890023	7.157482	6.862403
8125048_DDAH2	6.509935	6.383234	6.595133	7.170769	6.06552	6.429348
8125818_C6orf1	6.605795	6.584597	6.628843	6.8943	5.873776	6.363115
8127193_BMP5	4.918994	5.675906	5.328531	4.835456	6.002276	5.269704

Supplemental Table 1. Gene expression values changed greater than 2 fold.

Affymetrix ST 1.0 Array ID	0-No	6-No	12-No	0-Yes	6-Yes	12-Yes
8127522_DPPA5	5.562381	4.997332	4.725345	5.208942	4.010427	4.478637
8128322_C6orf66	7.171355	8.079125	8.311987	7.193904	8.32033	7.898156
8128356_FBXL4	6.558501	7.361424	7.4486	6.436738	7.441095	7.121601
8128394_SFRS18	7.833635	8.657491	8.356437	7.892068	9.083937	8.169077
8128459_SIM1	5.380199	5.79681	5.540092	4.825958	6.247098	5.524519
8128767_ZBTB24	5.456425	5.400241	5.320967	4.747875	6.055274	5.422573
8129218_MCM9	5.34409	5.767243	5.599938	4.808431	6.044332	5.579739
8129458_ARHGAP18	6.740369	7.455663	6.845184	6.684516	7.902869	7.252401
8130087_PPIL4	6.587911	7.047897	6.357269	6.218473	7.303484	6.542215
8130185_ZBTB2	6.171715	6.315117	6.111868	5.566898	6.697505	6.074
8130765_FAM103A1	6.271132	7.062165	7.143468	5.955303	7.128155	6.74694
8131975_TAX1BP1	6.467774	7.37003	7.282858	6.924963	7.932546	6.965912
8132369_EPDR1	7.549204	8.268213	7.77399	7.334579	8.800855	8.148168
8133202_TYW1	5.88089	6.046	5.707444	5.448113	6.499442	6.16066
8133215_FLJ13195	5.02435	5.890642	6.210593	5.189328	6.302119	6.195091
8133610_DKFZP434A0131	8.114581	8.916439	8.89394	7.894913	8.948584	8.732563
8133809_RSBN1L	6.774596	6.659984	6.68084	6.540749	7.570215	6.670972
8134079_GTPBP10	5.952531	6.575638	6.234323	5.660859	6.830927	6.086964
8134219_CCDC132	4.891673	5.650856	5.613097	4.828309	5.843738	4.837983
8134263_COL1A2	9.948545	9.029828	9.065537	10.16899	9.083663	9.921639
8134318_CASD1	6.767269	7.478345	7.293902	6.971018	8.054214	7.416672
8135069_SERPINE1	6.657064	5.812365	5.85717	8.362505	5.686234	5.795318
8135458_CBLL1	7.109476	7.864747	8.011892	7.014969	8.083966	7.655774
8135688_LSM8	5.859406	6.159134	6.027682	5.210338	6.297233	5.823519
8135718_ING3	6.278358	6.855132	6.777811	5.764776	7.306683	6.466703
8136631_SSBP1	6.462944	7.445861	7.867807	6.500396	7.624094	7.45773
8137240_GIMAP7	5.931249	6.602196	6.764221	5.320926	6.991703	6.732447
8137244_GIMAP4	6.366582	6.353788	6.633413	5.702463	6.780447	6.423123
8137526_INSIG1	6.600495	6.319596	6.922938	7.716378	6.279686	6.468948
8138030_PMS2	6.621886	6.715539	6.719894	5.711512	6.899828	6.828426
8138592_TRA2A	7.112416	7.956741	7.849087	6.639962	8.251318	7.722235
8138749_HOXA9	6.09234	7.082206	7.128325	6.169087	7.354091	7.290452
8139706_SEC61G	7.340135	7.31761	7.565572	6.175828	7.219184	6.919276
8139820_ZNF680	5.357931	6.197203	5.595047	5.214417	6.713883	5.343722
8139977_DKFZP434A0131	8.143051	9.046766	8.929169	7.8995	9.011929	8.830168
8140196_LOC442582	8.104163	8.996904	8.889761	7.930076	8.959257	8.720263
8140814_SLC25A40	4.849479	5.498258	5.961289	4.44059	5.946073	5.377897
8140878_KRIT1	6.610535	7.319144	6.819295	6.462705	7.637033	6.799554
8141016_TFPI2	4.490265	4.045586	4.266724	5.355671	3.913903	4.003129
8141024_BET1	5.839452	6.192578	6.481374	5.393302	6.65647	6.497366
8141094_PDK4	11.99889	11.0147	10.95012	12.34727	11.01651	10.78728
8141206_BAIAP2L1	5.723403	5.555609	5.65984	6.338172	5.308389	5.614394
8141477_GATS	8.39585	9.312346	8.999535	8.260312	9.472408	9.318171
8142136_FLJ36031	7.526555	6.881371	6.890657	7.541097	6.496438	6.995093
8142407_TMEM168	6.245629	7.17569	6.660879	6.269977	7.277153	6.900434

Supplemental Table 1. Gene expression values changed greater than 2 fold.

Affymetrix ST 1.0 Array ID	0-No	6-No	12-No	0-Yes	6-Yes	12-Yes
8142814_OPN1SW	5.500465	5.259853	4.876644	5.550145	4.429939	5.427592
8143441_KIAA1147	5.810128	6.205672	5.973242	4.637613	6.205893	6.106444
8143733_ZNF767	6.605792	7.154735	6.932573	6.351573	7.423338	6.91664
8143781_GIMAP6	6.175797	7.024065	6.861588	6.000599	7.378821	7.400361
8144089_LMBR1	6.87065	7.684372	7.57453	6.778156	7.923615	7.265672
8144982_NPM2	6.505846	6.028111	5.881419	6.331128	5.24727	5.634562
8145122_SLC39A14	6.833086	6.270169	6.582314	7.522273	6.379001	6.472919
8145702_WRN	4.784826	4.750988	5.17177	4.405942	5.451412	4.757219
8146198_POLB	7.400184	8.187524	8.151209	7.145695	8.161196	8.121456
8146357_MCM4	6.280932	6.526283	6.328936	5.357785	6.358462	6.294646
8146462_SOX17	8.460766	7.629125	7.554531	8.906934	7.715173	7.677036
8146544_LOC137886	5.734453	6.347418	5.931737	5.439416	6.787928	6.054829
8146794_DEPDC2	5.8252	6.328081	6.042392	6.065089	7.068352	5.870982
8148333_KIAA0143	8.141186	8.997662	8.967897	8.202433	9.294497	8.707338
8148658_ZNF623	6.515734	6.788625	6.824911	5.743967	6.972302	6.709953
8148966_FLJ45055	4.228218	4.013688	4.126215	3.440878	4.441253	4.233659
8150266_LSM1	6.740457	6.862396	7.041902	5.812634	6.899324	6.867355
8150592_CEBPD	8.824866	7.978872	8.206219	9.059242	7.691528	8.180485
8150714_PCMTD1	9.468423	10.38283	9.88381	9.479206	10.79011	9.59759
8150870_PSMC6	6.665069	6.871984	6.834625	6.228008	7.284869	6.445654
8151066_ARMC1	6.198506	6.956841	6.953952	6.287183	7.3776	7.078692
8151561_ZFAND1	6.29093	6.857371	7.010739	6.210393	7.326137	6.762718
8151768_RUNX1T1	5.452919	6.28898	5.879809	5.430977	6.452858	5.914793
8151816_GEM	5.711055	5.515296	5.298596	6.581283	5.183179	5.790974
8151890_TP53INP1	6.834571	7.738989	7.60615	6.565313	7.581733	7.792286
8151942_HRSP12	5.330698	5.874292	6.522661	5.30383	6.459155	6.153021
8152111_ZNF706	7.317776	8.017426	7.97185	7.093716	8.33711	7.944478
8152133_RRM2B	6.244199	7.051155	6.369362	6.252204	7.35147	6.508754
8152291_ABRA	8.557728	8.309313	8.094995	9.265299	8.233755	7.975515
8152553_TAF2	5.492318	6.420124	6.462157	5.907992	6.946277	6.255347
8154725_LOC442405	6.910513	7.366986	6.806571	6.365081	7.697032	7.048018
8155083_CA9	6.961946	6.570574	6.645897	6.799041	5.641469	6.030615
8155487_KGFLP1	4.138922	4.40127	4.690873	3.713552	4.756933	3.971687
8156126_RMI1	4.866489	5.155325	5.417251	4.295469	5.382066	4.958475
8156538_HIATL1	6.390723	7.222861	7.116346	6.287248	7.409386	7.114601
8156581_LOC375748	5.470396	6.338371	6.172638	4.993646	6.680442	6.284297
8157074_FCMD	4.749165	5.579906	5.151184	4.782672	5.923487	5.461441
8157216_UGCG	6.290567	5.907659	6.13916	7.040956	5.849964	6.168316
8158147_SLC25A25	6.807448	6.409109	5.818429	7.660439	6.621274	6.129785
8160011_KIAA2026	7.401309	8.097604	7.90539	7.586801	8.653823	7.424036
8160088_MPDZ	6.289067	7.260168	6.65251	6.507133	7.603673	6.658088
8160756_WDR40A	8.015972	8.091442	8.913435	7.689013	8.694736	9.388083
8161558_AQP7P1	7.145616	6.280246	6.579401	7.642309	6.497808	6.454254
8162006_GKAP1	4.816405	5.792952	5.629454	4.884465	6.186223	5.419644
8162276_NFIL3	6.329495	5.64381	5.782287	7.884641	5.246184	5.414359

Supplemental Table 1. Gene expression values changed greater than 2 fold.

Affymetrix ST 1.0 Array ID	0-No	6-No	12-No	0-Yes	6-Yes	12-Yes
8162404_ECM2	4.96289	4.805417	4.615562	5.32741	4.140855	4.524091
8162719_HEMGN	4.141367	3.834208	3.858663	3.273431	4.934641	4.228833
8163795_PSMD5	6.435298	6.76766	6.799051	5.663863	6.996596	6.599856
8164105_GOLGA1	6.073164	6.423403	6.071976	5.732506	6.898639	6.560551
8165295_LCN8	4.964732	4.442273	3.440875	5.51654	4.170644	4.091578
8165508_NRARP	7.479111	7.042748	6.762422	7.565563	6.554907	6.843815
8165817_GYG2	5.933066	5.365083	6.423223	6.574878	5.497612	5.715734
8166104_OFD1	4.906788	5.436267	5.039506	4.774211	5.81698	5.118525
8166469_SAT1	7.709967	6.898266	7.929262	8.719091	7.422783	7.348505
8166509_FAM48B1	5.476808	4.744057	4.47445	5.126261	3.790676	4.178633
8166956_UTX	5.910208	6.728347	6.926766	5.925391	7.1656	5.990303
8166989_ZNF673	6.217299	6.175173	6.11523	5.018926	6.069937	5.591566
8167443_MAGIX	5.267827	4.949628	5.109351	5.663099	4.536465	5.092918
8167575_GAGE2	6.887156	6.305254	6.665104	7.513209	6.442425	6.151084
8168470_COX7B	8.789742	9.746183	10.31174	8.15852	9.763055	9.506826
8168737_TNMD	4.788949	3.795541	3.719203	4.636999	3.476412	3.494456
8169233_KRT18P49	4.713567	4.647329	4.74271	6.297106	4.824029	5.204544
8170630_PNMA6A	6.40971	5.753345	6.147017	6.778225	5.708682	5.964149
8171024_LOC401622	5.673075	6.328619	6.187135	5.577251	6.78619	6.201701
8171105_CRLF2	4.679162	4.937192	4.225702	4.918089	3.843294	5.098428
8171435_PIR	6.219255	7.1211	6.517516	5.959093	7.052096	6.770943
8171747_EIF1AX	8.127961	8.921551	8.275009	8.135375	9.246709	8.424968
8171844_FAM48B1	5.476808	4.744057	4.47445	5.126261	3.790676	4.178633
8171865_hCG_1983058	8.7195	8.54927	9.588707	8.074902	9.083297	8.996172
8172119_MED14	8.434058	9.331617	9.197519	8.418589	9.444625	9.154775
8173310_OPHN1	6.229843	6.94174	6.585545	5.735072	6.982122	6.85149
8173673_ATRX	6.149245	6.9452	6.370249	6.286315	7.401193	6.340785
8174119_ZMAT1	5.178037	5.173124	5.215218	4.532273	5.57072	4.839269
8174340_RBM41	5.471262	5.88772	5.278257	5.164535	6.48006	5.725269
8174375_TEX13B	6.096377	5.709407	5.506052	6.188552	5.050968	5.351186
8174717_UPF3B	4.944548	4.636454	4.884588	3.880302	4.91587	4.40698
8174820_C1GALT1C1	4.387868	4.920879	4.627435	4.336903	5.400486	5.12331
8175177_MBNL3	6.386016	6.111496	7.315578	5.603928	7.402764	7.069409
8175432_SNORD61	7.349695	8.306662	7.718751	6.213256	7.335699	7.474243
8176253_LOC401622	4.875163	5.815413	4.599593	4.714663	5.813653	5.610387
8176255_TMLHE	6.757798	7.492785	7.462515	6.671021	7.817971	7.429689
8177507_GTF2H2	6.063915	6.41286	6.549732	5.621618	6.672122	6.373585
8177560_BDP1	6.281654	6.954798	6.698261	6.280675	7.477028	6.579059
8178590_DDAH2	6.653308	6.427193	6.639092	7.138685	6.122843	6.371207
8179819_DDAH2	6.653308	6.427193	6.639092	7.138685	6.122843	6.371207
8161442_	6.762582	6.964027	6.728585	7.530807	6.379124	6.837928
8162260_	6.372272	6.240705	7.200121	5.609784	6.956881	6.611383
7897522_RBP7	4.991099	5.16437	6.075931	5.783917	5.373718	5.738196
7897737_C1orf187	6.601207	5.990502	5.588528	6.25875	5.680642	5.827501
7900413_ZMPSTE24	6.367295	7.268825	7.457134	7.386834	7.634488	7.249871

Supplemental Table 1. Gene expression values changed greater than 2 fold.

Affymetrix ST 1.0 Array ID	0-No	6-No	12-No	0-Yes	6-Yes	12-Yes
7901048_SNORD46	7.232169	7.935962	8.66888	7.801403	7.26597	7.500559
7902721_CLCA1	3.885071	3.243686	2.787771	3.423092	2.645452	3.071884
7904361_FAM46C	7.508297	8.308243	8.535298	7.609064	8.436947	8.904562
7905395_PSMB4	8.836044	9.673685	9.859327	9.182992	9.823839	9.917254
7906041_DAP3	7.015718	7.861984	8.037087	7.185086	7.324045	7.989353
7906365_OR10K1	5.308424	4.633724	4.193307	4.347018	4.139647	4.846683
7906764_HSPA6	7.365357	6.591398	6.340744	6.434863	6.243455	6.449561
7908388_RGS1	4.904858	3.982839	3.786667	4.819324	3.883287	4.157856
7908692_LOC376693	4.716434	5.386714	5.752631	5.862534	5.56503	5.193068
7908766_TIMM17A	6.039553	7.021626	7.059104	6.867309	7.205693	6.867241
7909815_IARS2	7.233809	8.196957	8.242524	7.641699	8.029846	8.071221
7910014_CNIH4	7.862597	8.296654	8.925535	8.117295	8.55798	8.761858
7911267_OR2M3	3.990619	3.703097	2.989404	3.193073	2.846644	3.681746
7912157_ERRFI1	7.569024	6.823054	6.37506	8.111812	7.114334	6.903581
7912670_UQCRH	8.857226	9.447911	10.05278	8.438149	9.36341	9.519378
7914216_SNORA16A	7.045409	7.070052	8.479781	6.833188	7.305147	6.836582
7915896_CYP4Z2P	4.104381	4.232529	2.85092	3.647353	3.683414	2.786047
7918284_TAF13	5.851533	6.691513	6.872126	6.959493	6.955426	6.586421
7920205_SPRR2A	4.538881	4.820828	3.264717	3.698223	3.421204	4.066917
7920252_S100A7	6.194802	5.344319	4.494814	5.555942	4.980778	5.452983
7921487_TAGLN2	8.935539	8.051498	7.466492	8.208811	7.575162	8.077034
7922127_XCL2	4.377287	3.886911	3.283985	3.299441	3.27986	3.335684
7922137_NME7	6.180393	5.321186	5.116429	4.968917	5.024473	5.122935
7922418_SNORD74	6.242885	7.170451	7.40497	7.031722	6.59706	6.824052
7923378_CSRP1	8.271708	7.378493	7.230135	8.276193	7.396706	7.869409
7924119_INTS7	5.160269	5.844899	6.431533	5.795062	6.437438	5.841209
7924172_NSL1	7.148838	8.103195	8.250333	7.60392	8.21804	8.298288
7925182_SNORA14B	5.550414	5.515723	7.141001	5.366708	5.163441	5.24486
7926821_MASTL	4.006459	4.475043	5.169294	4.798822	4.815891	4.64964
7927280_PPYR1	4.345874	3.493876	3.341026	2.581126	2.867271	3.149151
7927502_SLC18A3	6.661856	5.81739	5.617679	6.120291	5.64309	5.41495
7927784_MGC14425	7.131596	6.393221	6.029145	6.734319	6.032926	6.387687
7928602_SFTPA1B	6.448347	5.623583	5.391818	5.740344	5.493867	5.713026
7928632_SFTPA2B	6.448347	5.623583	5.391818	5.740344	5.493867	5.713026
7929116_RPP30	6.414934	7.193507	7.472837	6.678061	7.465085	7.079491
7931389_CTAGEP	3.416246	4.084861	4.646793	4.209807	4.129633	4.288554
7931671_DUX4	7.412907	6.962575	6.409386	6.962824	6.721977	6.735394
7931832_AKR1C2	7.142639	7.82648	9.344638	8.681243	8.485197	8.169376
7932094_PHYH	7.932172	8.603945	8.937428	8.354151	8.893777	8.946588
7932530_PIP4K2A	6.568702	6.63195	8.080858	6.735543	7.319457	8.138304
7933194_CXCL12	7.982629	8.676096	9.053449	8.43728	9.02742	8.836493
7933228_8-Mar	6.360213	7.284438	7.861816	6.841504	7.755583	7.86346
7933366_GDF2	6.488731	5.496109	5.368094	6.040462	5.564269	5.672816
7933484_LRRC18	5.376709	4.625245	4.14737	5.280593	4.904055	4.421758
7935002_SRP9	8.288502	9.220008	9.416162	8.819892	9.361076	9.127201

Supplemental Table 1. Gene expression values changed greater than 2 fold.

Affymetrix ST 1.0 Array ID	0-No	6-No	12-No	0-Yes	6-Yes	12-Yes
7935707_CHUK	5.580229	6.555377	6.749611	6.077094	6.899131	6.391906
7936091_USMG5	9.030151	9.538555	10.17236	8.785136	9.415022	9.538352
7938172_OR10A5	4.283453	3.490453	3.214724	3.119323	3.813023	3.571649
7938176_OR10A2	4.546959	3.588181	3.181519	3.407883	3.481638	3.118846
7938301_C11orf17	5.48927	6.246764	6.62792	5.876973	6.419416	6.460676
7938329_SNORA23	6.38816	6.893355	8.213055	6.691008	6.641333	6.373181
7939150_PRRG4	5.794508	5.050856	4.657771	4.939013	4.936351	4.806424
7939825_NDUFS3	9.014249	9.88539	10.07136	9.441738	9.811472	9.985488
7940147_FAM111B	4.807274	4.151511	3.783658	4.277858	4.012317	4.376531
7940473_TMEM138	5.647502	5.97733	6.80441	5.906367	6.461837	6.573193
7945660_FAM99A	5.079995	4.429543	3.987485	4.779577	4.08958	4.938074
7945801_SNORA54	6.836258	7.388804	8.671158	6.417747	7.055333	6.548425
7945864_ZNF195	5.179186	5.824421	6.218166	5.65659	6.448367	5.904293
7946033_HBB	11.32187	11.38117	12.32562	11.33988	12.05758	12.46073
7946048_HBG2	5.810866	5.685913	7.165334	5.255821	6.175326	5.729337
7946446_NRIP3	5.587478	4.93676	4.511786	5.516535	4.550957	4.880395
7946728_PSMA1	7.910154	8.533182	8.946137	8.465911	8.73057	8.647886
7946986_SAA1	3.981978	3.252228	2.768277	3.08094	2.987171	3.600718
7948420_FABP5	7.461313	7.888904	8.551104	7.709092	8.406896	8.033457
7948898_SNORD31	4.641407	4.965319	5.944927	4.563387	5.240364	5.637269
7949674_RBM4B	7.349206	8.148123	8.531042	8.0982	7.98927	8.219604
7949995_MRPL21	6.846861	7.478787	7.984715	7.106327	7.622032	7.590907
7950753_CCDC90B	5.576197	6.486331	6.591547	6.34248	6.41837	5.968524
7951034_SNORA8	6.403642	7.108209	7.614398	6.543925	6.908863	6.584037
7952335_LOC85391	4.352586	5.232194	5.443914	4.87941	5.122991	4.955006
7953032_LRTM2	6.624944	5.903391	5.577388	6.153152	5.91503	5.844863
7955865_HOXC9	5.207573	6.031609	6.327508	5.917783	5.801261	5.854219
7955890_HNRNPA1	4.064992	4.674253	5.267384	3.878263	4.543137	5.370362
7956031_ORMDL2	4.780878	5.341862	5.929323	5.280678	6.124986	5.938906
7956159_RPL41	5.690728	6.376261	6.933506	5.903146	6.374018	6.524791
7957508_CLLU1	3.791361	2.869501	2.688082	3.195376	2.748128	3.157154
7959597_TMED2	8.20003	9.09135	9.219794	8.931776	9.493807	9.35939
7960052_SNORA49	7.769698	7.885092	8.982616	7.381416	7.86198	7.695082
7960365_EFCAB4B	4.990353	5.68987	6.131886	4.746539	4.994613	4.8627
7960728_SCARNA12	8.295731	9.291355	9.602046	8.461832	8.997492	9.095613
7961757_ST8SIA1	6.296616	5.633676	5.296342	5.254398	5.341285	5.543706
7962312_ABCD2	3.166953	3.639377	4.220662	4.019075	4.426743	3.311898
7962829_SNORA2B	3.62404	4.319273	4.859885	3.826905	4.495986	4.264524
7963406_KRT6B	4.888186	5.072267	5.918975	5.70459	4.938367	5.818212
7963567_KRT8	6.206903	5.318166	5.098699	5.481663	5.393806	5.353297
7963689_NPFF	4.565862	5.215352	5.644959	4.994806	4.84177	5.031545
7964142_APOF	4.906741	4.248562	3.713451	4.465864	4.090985	4.0457
7964271_PRIM1	5.118635	6.072511	6.172529	5.225072	5.98449	5.973239
7964347_KIAA0286	4.946664	5.36126	5.97068	5.238847	5.951111	5.701958
7965846_CCDC53	5.582505	5.809861	6.67265	5.586234	6.01914	5.993548

Supplemental Table 1. Gene expression values changed greater than 2 fold.

Affymetrix ST 1.0 Array ID	0-No	6-No	12-No	0-Yes	6-Yes	12-Yes
7966332_VPS29	6.879909	7.270594	7.880348	6.816558	7.69308	7.421953
7967028_RNU4C	4.448364	4.967394	6.300661	4.11401	4.290613	4.3864
7967030_RNU4B1	7.714719	7.689281	8.813121	7.120098	7.10249	7.44079
7967881_HSMPP8	5.499471	6.239695	6.574129	6.197776	6.798136	6.30753
7968270_LOC432369	6.978962	7.384668	8.049265	7.147938	7.69678	7.702619
7968872_DNAJC15	6.272803	7.181584	7.326573	6.282198	6.835664	6.925663
7973936_PSMA6	8.205266	8.862076	9.324328	8.577505	9.013122	9.256948
7973985_MIPOL1	5.536548	4.89576	4.104874	4.496899	4.828326	4.645205
7974447_SOCS4	4.336517	5.167651	5.340507	5.131311	5.469264	5.235602
7974647_UNQ9438	4.753914	3.875538	3.699179	4.109482	3.827646	3.973032
7975989_C14orf156	9.885709	10.26466	10.9016	9.55197	10.36219	10.44961
7977879_PSMB5	7.339598	8.05387	8.441978	7.416543	8.218868	8.351385
7978123_PSME2	4.915235	5.486327	5.919428	5.510307	5.52255	5.847712
7979864_ERH	8.309494	8.574195	9.450258	8.198373	8.602168	8.696795
7980773_CCDC88C	7.561744	6.927766	6.450913	6.652364	6.876944	7.106813
7981745_IGHV7-81	5.419549	4.555662	4.055549	4.406979	4.319214	4.242418
7981949_SNORD116-1	6.395892	6.882689	8.05004	6.392399	7.088985	7.262234
7981953_SNORD116-3	8.982605	9.771647	10.18341	8.780304	9.578318	9.54465
7981955_SNORD116-4	6.463026	7.378038	7.51118	6.485666	7.011087	7.231085
7981960_SNORD116-6	4.537221	5.504118	6.138357	5.022491	5.303481	5.246783
7981964_SNORD116-8	8.551792	9.218652	9.633554	8.203421	8.988594	8.957351
7981966_SNORD116-3	8.982605	9.771647	10.18341	8.780304	9.578318	9.54465
7981996_SNORD116-24	10.2484	10.8924	11.38635	10.36789	10.93354	10.67441
7982070_SNORD115-32	5.876432	5.709281	7.798697	6.026777	6.501827	6.058663
7982712_C15orf23	4.744602	5.581684	5.843113	5.273001	5.892491	5.978362
7982904_RTF1	6.393274	7.324714	7.41971	6.994223	7.694208	7.429784
7989708_MTFMT	6.436922	7.099813	7.442984	6.276381	7.123533	6.938666
7990191_BRUNOL6	6.861453	6.354406	5.808498	6.302281	5.871603	5.986029
7990442_RPP25	6.858191	5.986436	5.831606	6.821119	5.928533	5.947889
7993083_A2BP1	6.557621	7.483582	7.6735	6.538573	7.53199	7.689692
7994237_LCMT1	7.225349	7.908478	8.255068	7.459725	7.854304	8.19424
7994565_LOC730092	7.568084	8.100186	8.576616	7.754555	8.570761	8.445885
7995267_TP53TG3 // LOC729355	4.980518	4.573371	3.579939	4.49159	4.045901	4.088287
7995282_TP53TG3	4.970253	4.434169	3.934949	4.426363	4.124107	4.20822
7996807_ZFP90	5.175489	5.830837	6.235888	5.487788	6.214823	5.955725
7996934_NIP7	6.120117	6.544734	7.174997	6.732649	6.831851	6.669825
7997188_HP	4.576985	4.144607	2.909475	3.832612	3.035072	3.476251
7997933_RPL13	6.248569	7.110846	7.405118	7.644036	7.236916	7.28581
7998666_SNORA64	5.984398	6.66661	7.51202	6.358517	6.349639	6.240555
7999419_DEXI	7.406742	7.560812	8.421715	6.75485	7.384009	7.797566
7999440_PRM1	5.857141	4.863749	4.490033	5.281946	4.644145	4.647548
7999674_MYH11	7.544652	6.586572	6.091345	6.876256	6.367313	6.9981
8001072_TP53TG3	4.970253	4.434169	3.934949	4.426363	4.124107	4.20822
8001211_ITFG1	6.876071	7.874406	7.987127	7.263765	8.17149	7.722275
8001971_LRRC29	6.675648	5.730376	5.64463	6.035448	5.866411	5.859159

Supplemental Table 1. Gene expression values changed greater than 2 fold.

Affymetrix ST 1.0 Array ID	0-No	6-No	12-No	0-Yes	6-Yes	12-Yes
8002218_RBM35B	6.714487	6.211141	5.711931	6.129339	5.841579	6.192247
8002660_TXNL4B	5.263995	6.232015	6.545619	6.013998	6.394685	6.904896
8003719_HIC1	8.11194	7.455806	7.094351	7.914056	7.210163	7.176768
8004506_SNORA48	10.14868	10.56679	11.30563	10.40883	10.56065	10.43908
8004534_SHBG	6.291507	5.305383	5.0316	5.25968	4.992546	5.139394
8007323_CNTNAP1	6.293044	5.531048	5.040298	6.046488	5.755756	5.501309
8007397_PSME3	6.885412	7.649961	7.953877	7.371258	7.942677	7.913402
8007471_NBR1	7.315206	8.180734	8.332941	7.617066	8.255755	8.250656
8008802_GDPD1	4.213367	5.190648	5.222133	4.518855	4.874682	4.77284
8008870_TMEM49	7.62761	8.13619	8.933946	8.40707	8.891161	8.656447
8011875_DERL2	7.284755	7.664218	8.287755	6.825938	7.653648	7.549588
8012581_STX8	6.598841	7.116681	7.618814	6.904596	7.371948	7.37124
8012663_MYH8	3.832343	4.620603	5.431208	4.354354	4.693874	4.350161
8013319_GRAP	6.032331	6.234802	7.408343	5.947857	6.026336	6.441637
8015642_PSMC3IP	6.192584	5.967183	5.17791	5.899679	6.111186	6.138799
8017019_MTMR4	5.84952	6.621588	6.856561	6.117281	6.520018	6.624957
8018558_ACOX1	6.890687	7.63827	7.934059	7.302959	7.826519	7.808987
8019018_CBX4	7.354774	6.891408	6.209741	7.054838	6.483592	6.58376
8020241_TNFSF5IP1	6.063184	6.955233	7.270936	6.568259	6.704	6.863514
8020349_ANKRD20B	3.506273	2.741508	2.410699	2.453065	2.951857	2.367894
8020762_DSG3	5.249979	4.350938	3.988371	4.374769	4.109721	4.646195
8020827_MEP1B	4.258112	3.65257	3.227633	4.156852	3.274534	3.624417
8021645_SERPINB10	4.499967	3.640908	3.362637	3.546131	3.402676	3.562374
8022972_RPL7A	8.512184	9.29405	9.530956	8.530231	9.429602	9.486573
8023855_CYB5A	6.603375	6.750008	7.801548	6.662021	6.761992	6.829062
8026875_SNORA68	8.745274	8.962025	9.919765	8.321724	8.966137	8.635794
8030383_RCN3	6.903423	5.955765	5.825467	6.44797	6.228923	6.722488
8031840_NAG18	4.251145	3.36359	2.890768	3.066185	3.022686	3.119491
8031893_ZNF8	6.264689	5.988348	5.11486	6.187048	5.909567	6.360029
8034299_ELOF1	6.721955	7.681135	7.892945	7.335651	7.922143	7.909512
8035083_CYP4F2	4.511842	3.848645	3.222295	3.749809	3.330276	3.634408
8036176_NPHS1	5.826985	4.882867	4.746943	5.4881	5.163862	5.334244
8036707_LRFN1	6.261105	5.74347	5.159097	5.71102	5.515916	5.490663
8036830_TTC9B	6.937459	6.503078	5.869592	6.476419	5.8421	6.034878
8037103_GRIK5	6.741319	5.785478	5.555097	6.089973	5.637958	5.785906
8038048_CCDC114	6.645855	5.953968	5.592372	6.305659	5.765266	6.023329
8038919_ZNF350	4.563313	5.316288	5.672081	5.044566	5.444136	5.150843
8039075_VN1R4	3.848976	3.082995	2.534473	2.903335	3.270494	2.848087
8039378_SYT5	6.493549	5.716555	5.443073	6.00637	5.548303	5.689492
8040036_RPS7	7.230949	7.735891	8.235307	7.37095	7.86075	7.959695
8040223_RRM2	3.19665	3.647385	4.206628	3.872117	3.910199	3.808944
8042439_ANTXR1	7.337707	6.533924	6.241339	7.430225	6.490082	6.997866
8043848_MRPL30	6.463296	7.125902	7.523311	6.700972	7.276717	7.499794
8044804_DBI	7.137305	7.455796	8.203703	7.240825	7.687659	7.717855
8045146_FAM128B	6.872479	6.639128	5.834004	6.527117	6.315186	6.367386

Supplemental Table 1. Gene expression values changed greater than 2 fold.

Affymetrix ST 1.0 Array ID	0-No	6-No	12-No	0-Yes	6-Yes	12-Yes
8046628_RBM45	3.471358	4.334807	4.500628	4.010748	4.743904	4.470669
8046646_OSBPL6	7.135762	6.899464	5.958634	7.182717	7.441626	6.662536
8049299_SCARNA6	8.760578	8.646259	9.948416	7.870769	8.510073	8.541967
8051066_MPV17	6.429441	7.135218	7.50982	6.759201	6.990508	6.896514
8051197_CCDC121	6.622601	6.125651	5.315676	5.479351	6.241786	5.626482
8052024_FBXO11	6.340442	7.313321	7.424888	6.615198	7.542066	7.098233
8053036_TPRKB	5.69909	5.997188	6.739956	5.358496	6.253086	6.399792
8053165_MRPL53	8.188533	8.927477	9.356466	8.338451	9.092367	9.281376
8053200_DQX1	5.50944	4.946821	4.46948	5.016875	4.675061	4.942183
8053594_CD8B	4.063561	3.214129	3.029797	3.473104	3.570347	3.369644
8054467_10-Sep	5.589303	6.278224	6.644707	5.8721	6.078341	5.972762
8056753_METTL8	5.002801	4.867668	6.065362	4.931963	5.610335	5.745128
8062908_STK4	6.060828	6.623811	7.130813	6.738051	7.235126	6.987233
8065388_CST11	4.945892	4.19237	3.81116	4.348823	4.456026	4.121129
8068363_C21orf82	5.983039	6.317256	7.035587	5.789844	6.423162	6.77523
8073334_RBX1	7.61955	8.127064	8.841396	7.895501	8.405623	8.459515
8075365_RNF215	7.362869	6.37666	6.326937	6.912516	6.400262	6.555916
8078008_LSM3	6.678021	7.315523	7.952225	6.484243	7.300167	7.449489
8079217_ZNF35	4.05027	5.042496	5.096011	5.271277	5.290827	5.303437
8082270_CCDC37	5.421849	4.997179	4.280795	4.694709	4.672325	4.744434
8084067_ACTL6A	5.035862	5.981092	6.444057	5.424887	5.919713	6.182514
8084766_TP63	7.1574	8.117723	8.168464	7.264683	7.885508	7.702507
8087254_IMPDH2	7.626356	8.558188	8.689896	8.142871	8.680669	8.846457
8089026_STX19	4.283063	3.607483	3.236633	4.254265	3.655042	3.537845
8089771_CD80	4.852697	4.042956	3.825848	4.231614	4.054235	4.159887
8090664_ASTE1	5.829714	5.509551	4.815014	5.015937	5.322421	5.30908
8091402_TM4SF18	5.149292	5.983834	6.501557	5.962797	6.850949	5.393967
8092404_MAP6D1	6.413858	7.084937	7.532867	7.118751	7.229877	7.316399
8093104_TM4SF19	6.640411	6.082623	5.617105	6.345166	5.639365	6.319202
8096415_MMRN1	5.304186	4.392094	4.175542	4.239313	4.110741	4.773914
8096771_DC2	6.498001	6.864644	7.531056	6.406992	6.902138	6.861703
8097056_SNORA24	6.868355	7.237325	8.275806	6.496302	7.270149	7.204948
8097679_LSM6	4.763268	5.593095	5.815806	4.747964	5.304645	5.036106
8098170_TRIM60	5.613949	4.761293	4.570395	4.466945	4.54358	4.564851
8098782_CPLX1	7.046509	6.402672	6.034616	6.731709	6.214132	6.447739
8101762_SNCA	6.759131	6.540864	7.899609	6.618137	7.110796	7.494172
8101925_MAP2K1IP1	6.210988	7.128147	7.353897	6.674994	7.196796	7.068572
8101945_H2AFZ	5.939003	6.176381	7.1525	6.576973	7.338449	6.873148
8102998_GYPA	2.958305	3.117186	4.068838	2.981252	3.094152	3.909788
8104394_ADCY2	8.363691	9.295835	9.580463	9.022706	9.508833	9.388995
8104570_FAM105A	4.210664	4.650443	5.325058	4.669244	4.932786	4.770474
8105331_GZMK	3.038394	3.822122	4.352391	3.996037	3.616235	4.01423
8106271_TINP1	7.019252	7.833429	8.350213	7.480973	8.321769	7.959871
8107814_ISOC1	6.412308	7.308773	7.439718	7.020394	7.484981	7.284526
8109049_SPINK7	3.843461	3.042685	2.819458	3.148739	2.98058	2.772058

Supplemental Table 1. Gene expression values changed greater than 2 fold.

Affymetrix ST 1.0 Array ID	0-No	6-No	12-No	0-Yes	6-Yes	12-Yes
8109428_SAP30L	5.729462	6.627791	6.967594	6.87602	6.678326	6.592163
8109475_MRPL22	5.440522	6.185044	6.474235	5.643421	6.354178	6.280973
8109651_GABRA6	4.619303	3.881469	3.600125	3.913858	4.032452	3.959589
8110886_MED10	5.83895	6.486063	7.04847	5.512035	6.381513	6.516977
8111998_HCN1	4.922187	5.767326	5.941094	5.061854	5.810128	5.72653
8112728_RAP1B	6.857905	7.673147	7.881384	7.779111	7.868958	7.417007
8113591_PGGT1B	5.988955	6.741658	7.019584	6.433247	6.950877	6.430954
8113623_TICAM2	6.023341	7.010126	7.098047	6.563933	7.125378	6.916332
8114249_CXCL14	7.900488	8.877012	8.92424	7.98904	8.919143	8.324277
8114618_NDUFA2	7.077593	7.658314	8.381472	6.985738	7.762661	8.119145
8114628_HARS	6.144686	7.123841	7.241039	7.018395	7.306301	7.086761
8116532_SNORD95	9.57909	10.25657	10.756	9.76263	9.715917	9.706124
8116983_CD83	6.756126	6.231033	5.715615	6.686275	6.404285	6.10609
8117580_HIST1H2AI	4.789223	3.969703	3.674602	4.998631	4.848033	5.006454
8117714_OR2J3	4.809708	3.848482	3.759881	3.780108	3.795875	3.689349
8118571_PSMB9	5.867626	6.652236	7.249053	6.265434	6.729702	7.047723
8118794_HMGA1	8.321013	7.562109	7.211993	7.453268	7.379007	7.489995
8118932_DEF6	7.214966	6.723483	6.192086	6.72881	6.285854	6.741179
8122127_TAAR9	4.987052	4.014951	3.747503	3.926332	3.729382	4.121997
8123584_RP11-145H9.1	8.310055	7.641499	7.20419	7.928967	7.563958	6.527591
8124498_ZNF204	3.401976	3.806152	4.443769	4.285518	4.186509	4.049271
8124521_HIST1H4K	6.038923	6.282195	7.08063	6.659236	6.176598	6.695028
8124865_PSORS1C2	7.483585	6.568889	6.444777	6.725507	6.4013	6.669502
8126095_C6orf129	6.017048	5.107367	4.929458	5.329246	5.188351	5.241169
8128001_CGA	5.136437	4.478414	3.968804	4.705586	4.416974	4.457062
8128638_SCML4	6.447295	5.606784	5.383951	5.666551	5.491721	5.834527
8129649_C6orf192	4.549209	5.158441	5.647876	4.925339	5.335122	5.643591
8130151_RAET1E	4.485738	4.101574	3.290765	3.522027	3.500403	3.695212
8133106_SNORA22	5.376526	5.248307	6.71247	4.935911	5.151579	5.022284
8134599_CPSF4	6.210682	6.982839	7.362725	6.612155	6.92027	6.977538
8136315_LRGUK	4.766272	4.484732	3.697056	4.041809	3.714176	4.251882
8139330_CAMK2B	6.905263	7.841281	8.29748	7.439982	8.080236	8.243492
8139482_SNORA5A	5.519316	6.387874	6.731894	5.532638	6.083447	6.294796
8140500_TMEM60	4.432503	5.065982	6.071932	5.204208	5.591937	4.929887
8140840_STEAP4	5.815552	6.746226	6.912592	6.970942	7.059849	6.608107
8142019_ORC5L	5.576711	6.465474	6.646008	6.042145	6.721977	6.210487
8143433_MRPS33	5.526974	5.939914	6.584005	5.528556	6.006305	6.102844
8143749_ZNF467	6.895054	6.184134	5.812397	6.509051	6.329555	6.495655
8145281_SLC25A37	8.242085	8.481958	9.310201	8.390787	9.034488	9.157306
8145317_ADAMDEC1	4.975407	4.135151	3.788718	4.575658	4.494889	4.40801
8147049_FABP5	7.413387	7.851024	8.520234	7.68826	8.365812	7.993862
8149955_PBK	4.137281	3.155484	2.96467	3.502728	2.947331	3.13242
8151587_C8orf59	4.794648	5.09847	5.885933	4.755755	5.498538	5.226716
8151592_CA1	4.067585	3.895957	5.195469	4.152794	4.762465	5.489309
8152323_EIF3E	7.447572	8.269119	8.462109	7.883099	8.609695	8.061629

Supplemental Table 1. Gene expression values changed greater than 2 fold.

Affymetrix ST 1.0 Array ID	0-No	6-No	12-No	0-Yes	6-Yes	12-Yes
8155665_PGM5	6.242955	6.583866	7.252482	6.21873	6.431622	6.789256
8156826_TGFBR1	6.416239	7.411782	7.43936	7.320518	7.836549	7.171808
8157947_ZBTB34	5.556679	6.246055	6.58462	5.703843	6.285666	6.373561
8162194_CCRK	6.133615	5.375488	5.103652	5.483626	5.49893	5.158345
8162880_MRPL50	6.872709	7.17145	7.883369	6.902197	7.702367	7.66493
8163481_CDC26	5.850667	6.43734	7.019204	6.541726	7.043903	7.122992
8163930_NDUFA8	7.71896	8.072261	8.778638	7.549716	8.257387	8.287035
8166747_SYTL5	4.210773	3.446655	3.03398	3.336696	3.278133	2.868798
8168107_DGAT2L3	5.368477	4.801177	4.292994	4.917823	4.430463	4.743983
8168534_TBX22	4.099654	3.297406	3.080532	3.678775	3.333992	3.375981
8170863_SNORA70	9.540524	10.3098	10.63832	9.879205	10.17783	10.01228
8173179_RP11-278E11.2	6.04291	6.414792	7.093625	5.985116	6.083411	6.215582
8175539_LDOC1	6.719853	6.323732	5.668829	6.558993	5.830704	5.843753
8175558_SPANXE	6.266425	5.760648	4.743894	5.710645	4.835789	5.213196
8177038_SRY	4.072423	3.498201	2.926836	3.314348	3.530409	3.665647
8177694_OR2J3	4.860336	3.938678	3.648962	3.893917	3.678903	3.653909
8178199_HLA-DQA1	7.565856	8.354104	8.574636	8.190291	8.276016	8.029788
8178211_PSMB9	5.867626	6.652236	7.249053	6.265434	6.729702	7.047723
8178445_PSORS1C2	7.483585	6.550678	6.453909	6.753506	6.414404	6.669502
8179003_OR2J3	4.860336	3.938678	3.648962	3.893917	3.678903	3.653909
8179489_HLA-DQA1	7.565856	8.354104	8.574636	8.190291	8.276016	8.029788
8179495_PSMB9	5.867626	6.652236	7.249053	6.265434	6.729702	7.047723
7897675_ANGPTL7	4.789442	4.066275	4.042624	4.878007	3.999782	3.868832
7899604_ZCCHC17	6.276155	6.741598	6.259976	5.79358	6.602512	7.194629
7903742_GSTM4	8.152956	8.816204	8.684694	7.971107	8.921636	9.024446
7906400_IFI16	6.171726	5.719353	5.619992	6.645423	6.041979	5.501863
7906671_USP21	6.538362	7.283828	7.263247	6.374025	7.29552	7.450953
7908492_CFHR4	3.896613	3.496444	3.993551	3.272453	3.120111	4.345457
7911559_MRPL20	8.365421	8.654632	9.132967	7.799774	8.599929	8.892014
7912347_CASZ1	7.048818	7.439129	7.402498	7.688442	7.356504	6.66516
7913211_PLA2G2E	6.46959	6.080048	6.04498	6.532319	6.118628	5.401077
7915787_PIK3R3	6.755583	6.800536	6.668142	7.59055	7.02777	6.56447
7919642_HIST2H2AB	7.111374	6.993364	7.082061	6.329442	7.310354	7.658996
7920264_S100A5	4.717227	3.872452	4.398505	4.68105	4.626724	3.598696
7923528_MYOG	9.015078	9.937634	9.876259	8.991903	9.919168	10.0498
7927732_ARID5B	9.069904	8.511349	8.714872	9.442447	9.105962	8.419579
7927964_SRGN	8.571998	8.21781	8.285058	9.343775	8.679981	8.259792
7928524_VDAC2	6.810433	7.421016	7.387445	6.77799	7.542104	8.098072
7929282_HHEX	6.863919	6.233124	6.393371	5.587256	6.097767	6.642815
7930714_ATRNL1	4.324926	4.805457	4.488853	5.111795	4.597335	3.823251
7932864_LYZL2	3.334244	2.689988	3.753816	3.859843	3.173221	2.770164
7932911_KIF5B	7.889837	8.674722	8.051155	8.640344	9.201756	7.611354
7933877_JMJD1C	6.159091	6.765933	6.73801	7.848089	7.676809	6.020648
7938730_MYOD1	7.050721	7.066543	6.920272	6.681952	6.772077	7.804085
7943413_BIRC3	5.259171	5.455449	5.236853	5.992827	5.453235	4.964368

Supplemental Table 1. Gene expression values changed greater than 2 fold.

Affymetrix ST 1.0 Array ID	0-No	6-No	12-No	0-Yes	6-Yes	12-Yes
7944382_VPS11	7.217252	8.17665	7.714717	7.354062	7.868942	8.359583
7947881_RAPSN	6.950848	7.391014	7.35254	6.574223	7.343007	7.665627
7947917_KBTBD4	7.248663	8.022667	7.794445	7.109602	7.855352	8.137171
7949619_SLC29A2	8.920671	9.429649	9.425034	8.486437	9.237188	9.627932
7950983_CHORDC1	4.850796	5.256424	5.192678	5.367639	5.638979	4.20983
7951032_SNORA1	5.242901	4.705974	4.714957	5.686297	5.093726	4.546237
7951077_SESN3	6.231152	6.986024	6.720618	6.004244	6.953661	7.126101
7955063_TMEM106C	6.046469	6.787155	7.003948	5.960611	6.610476	7.167064
7955156_CCDC65	4.62288	4.128548	4.250251	4.924922	3.939142	3.63535
7955997_OR6C76	3.534748	3.237329	3.656047	4.394214	3.566449	3.241312
7956819_RASSF3	5.837739	5.562305	5.912585	5.276077	5.506774	6.326564
7967212_HPD	5.48528	5.025417	4.870006	6.184374	5.414229	4.997315
7969613_GPC6	5.244796	4.569101	5.162961	5.724818	5.237786	4.667422
7973724_KIAA1305	6.46426	6.438007	5.692978	6.218065	6.021267	7.245559
7973900_C14orf19	3.335834	3.465213	3.476105	3.462209	3.928951	4.548228
7974054_SIP1	5.349341	5.333061	5.95164	4.678447	5.353008	5.8313
7974851_HIF1A	6.392011	6.311212	6.456021	7.187463	6.924111	6.177462
7976698_EML1	6.250298	6.895967	6.873016	6.404347	6.891739	7.414829
7977803_RBM23	7.22795	7.400968	7.762109	7.07159	7.691065	8.098286
7978201_NEDD8	8.8922	9.251156	9.808352	8.636067	9.619841	9.638063
7978846_POLE2	3.469312	3.658551	3.844522	3.316993	3.621242	4.432534
7981951_SNORD116-2	6.357969	5.741576	5.520257	5.129163	5.192197	6.496602
7981974_SNORD116-13	3.38898	4.145599	3.704713	2.83174	3.736807	4.447271
7982096_SNORD115-48	2.937743	3.837934	3.916501	2.614962	3.475034	3.822688
7982889_NUSAP1	5.257128	5.202482	5.661741	4.703918	5.611811	5.828025
7987315_ACTC1	8.256943	7.925735	8.843489	9.608392	9.024636	8.103852
7989975_CLN6	6.851419	6.651598	6.461458	5.700173	6.556095	6.856993
7994926_SNORA30	4.996066	4.098285	4.484287	4.025578	3.823736	5.144706
7995258_ZNF267	4.586463	5.401705	5.014367	5.424747	5.237305	4.392198
7995793_MT1L	8.361926	8.818283	8.156753	9.119847	8.588495	8.027421
7997525_MLYCD	7.665894	8.253769	8.169579	7.753029	8.214088	8.763545
7999754_XYLT1	6.777229	6.925509	6.826658	6.115247	6.684634	7.198246
8000582_SULT1A2	5.912364	5.735537	6.112911	5.878424	5.547233	4.400857
8006621_CCL4L2	4.039581	4.261343	4.348444	5.001338	4.644593	3.994545
8006999_CSF3	8.863384	7.879869	7.865833	9.045772	8.071126	7.810228
8007108_TMEM99	4.598794	4.663455	5.174824	4.086971	4.810435	5.168309
8008277_SGCA	9.440161	10.08889	9.97233	9.124844	10.09436	10.45689
8009493_KCNJ16	4.105325	3.61159	3.881803	4.484894	3.734719	3.411922
8009755_ARMC7	6.139519	5.526179	6.313056	5.363292	5.997135	6.387687
8013641_PIGS	7.111113	7.55214	7.430685	6.668813	7.42931	7.921891
8015991_SLC4A1	5.673526	5.111101	5.700813	5.319636	5.733591	6.671173
8016018_SLC25A39	8.364488	8.208763	8.869658	8.221969	8.539129	9.26095
8019651_CCL4L1	4.039581	4.261343	4.348444	5.001338	4.644593	3.994545
8019930_MRLC2	10.17666	10.26456	10.24222	10.45805	9.610916	9.377811
8021546_ZCCHC2	5.97546	6.590263	5.969365	5.572289	6.440232	6.594449

Supplemental Table 1. Gene expression values changed greater than 2 fold.

Affymetrix ST 1.0 Array ID	0-No	6-No	12-No	0-Yes	6-Yes	12-Yes
8024273_NDUFS7	8.606559	9.166431	9.28662	8.315214	8.740017	9.390234
8026024_ASNA1	7.223115	8.069151	7.859519	7.277144	7.856808	8.327173
8026541_FAM32A	6.195124	6.209499	6.399845	5.477857	6.004807	6.598317
8031792_ZNF211	4.865497	5.529425	5.703959	4.685687	5.39233	5.736904
8036406_ZNF571	5.276699	5.050946	5.340739	4.760423	5.091503	6.093346
8039706_ZNF256	5.89091	5.943855	5.688698	5.313156	5.611287	6.389776
8039939_MRPL20	8.365421	8.654632	9.132967	7.799774	8.599929	8.892014
8040831_C2orf28	8.195084	8.529058	8.969721	7.904878	8.655356	9.047555
8043682_SLC26A1	6.440148	6.797217	6.682711	7.377235	7.001085	6.078763
8046062_XIRP2	9.868613	10.04577	9.357053	10.33872	10.43994	8.974464
8048864_CCL20	4.536628	4.443351	4.269948	4.883774	4.159586	3.763809
8052654_PELI1	6.934071	6.352622	6.840732	7.600008	6.725186	6.397184
8054580_BUB1	4.865623	4.105208	4.085622	4.769393	4.359171	3.69699
8057506_FRZB	4.911078	4.826362	5.683643	3.812124	4.596486	4.816225
8057797_SDPR	7.008914	7.05387	6.799419	7.666638	7.102794	6.563771
8066493_SLPI	7.140273	6.408648	7.070004	7.608275	6.758797	6.548097
8067955_CXADR	4.864217	4.606462	4.482906	5.168386	4.362118	4.121584
8068593_ETS2	8.246086	8.258114	8.28754	9.284365	8.313736	8.114561
8069880_TIAM1	6.076532	5.171426	5.438724	6.343955	5.57629	5.244958
8070489_RIPK4	5.06155	4.883573	4.845417	5.864562	5.074225	4.745344
8070780_KRTAP10-3	7.079173	6.569196	6.372158	7.136084	6.319041	5.852025
8076415_RNU13P1	2.949223	2.893985	3.170818	3.63321	3.024491	2.275633
8082058_CSTA	5.618433	4.992781	5.226315	5.66207	4.887012	4.508571
8082663_NUDT16P	6.502346	5.982582	6.234216	6.799315	6.141194	5.75107
8085094_SUMF1	6.569256	6.269195	6.215571	6.016759	6.459952	7.0419
8088047_MUSTN1	10.01991	9.649483	10.29491	9.023615	9.445705	10.21056
8089714_LSAMP	5.105749	4.997553	5.133501	5.410101	5.124274	4.328632
8097058_CEP170L	4.605319	4.938761	4.148925	4.894461	4.725004	3.804901
8097086_MYOZ2	7.769625	7.757039	7.640658	8.597301	7.638783	7.461437
8098714_FRG1	5.570738	5.450495	5.088697	4.355792	5.219365	5.704406
8100557_SRD5A2L2	6.250585	7.232835	6.758335	7.756011	7.527453	6.478679
8102993_GYPB	3.294275	3.397632	3.356622	3.074889	3.514975	4.647974
8105040_OSMR	6.386626	5.762506	5.522786	6.906116	6.030268	5.463532
8107429_FLJ90650	4.707616	4.259806	4.463068	4.938659	4.27154	3.836777
8107909_SLC22A4	5.369266	5.469394	4.896469	5.931511	5.191809	4.82563
8113130_MCTP1	5.645876	5.363872	5.389071	6.281477	5.423337	5.274529
8116724_FARS2	6.047067	6.61977	6.61977	6.044136	6.464848	7.050783
8117458_BTN3A1	6.518956	6.647692	6.461128	5.799661	6.399514	6.904345
8122756_PLEKHG1	5.421368	5.51541	5.599468	6.307041	5.578404	5.165419
8130071_C15orf29	6.275538	6.368851	6.038116	6.144849	6.253187	4.53678
8130891_WDR27	4.967834	5.30495	5.512808	4.615136	5.362287	5.65783
8132843_FAM29A	5.258484	5.60063	4.769073	3.76288	4.686207	5.028972
8137250_GIMAP2	4.663322	4.768034	4.995006	4.459906	4.721576	5.623892
8138765_HOXA11	5.151284	5.895461	5.603904	4.696444	5.445608	5.98017
8140463_FGL2	8.054277	7.416603	7.366788	8.119413	7.498219	7.118032

Supplemental Table 1. Gene expression values changed greater than 2 fold.

Affymetrix ST 1.0 Array ID	0-No	6-No	12-No	0-Yes	6-Yes	12-Yes
8142524_TSPAN12	6.045742	5.790306	5.884196	6.537243	5.803946	5.426871
8145291_SLC25A37	7.330511	7.275078	7.814518	6.894532	7.864888	7.963064
8145454_BNIP3L	8.122131	7.915833	8.789401	7.834853	8.723036	8.912542
8146660_DNAJC5B	3.915569	3.623768	3.664056	4.485227	3.573993	3.391514
8146921_RDH10	6.213969	6.65962	6.21297	7.142505	6.387033	5.944783
8148208_FAM91A1	5.617275	6.274715	6.320097	6.644661	6.99207	5.626189
8148841_GPR172A	7.156955	6.808329	6.271789	7.480829	6.71708	6.349566
8151532_FABP4	8.027837	7.602491	8.838326	8.383098	7.687032	7.242294
8155849_ANXA1	8.072244	7.491304	8.028387	8.677685	7.949242	7.446582
8156043_PSAT1	4.76274	4.566828	4.090817	5.087203	4.202687	3.914473
8157945_ZBTB34	7.504962	8.180349	7.92624	7.075918	7.809109	8.095652
8160284_FAM29A	5.470673	5.420252	5.737957	4.705234	5.639615	6.05922
8161488_AQP7P2	7.476835	7.03131	7.269015	8.236851	7.318168	7.102831
8163202_SVEP1	6.509953	6.332793	6.323548	7.187453	6.663311	6.081928
8164644_FAM78A	6.754598	7.749325	7.551904	6.784562	7.387138	7.886058
8166714_LANCL3	4.597734	3.941389	4.286032	4.908961	4.091189	3.830587
8168291_ITGB1BP2	5.931814	6.858518	6.864472	5.932562	6.750866	7.146754
8168391_hCG_1731871	5.317295	5.367229	5.09313	5.581115	4.876157	4.54801
8171418_PIGA	5.673693	5.590764	5.611309	6.408059	5.569117	5.314878
8172358_UXT	7.41725	7.624097	8.225835	6.955205	7.751176	7.956558
8173135_ALAS2	6.490158	6.314551	7.436267	6.801545	6.597239	8.505279
7900426_SMAP1L	6.533049	6.007845	7.13162	7.32266	7.007076	6.922139
7908397_RGS13	3.95687	4.441093	3.3897	3.696563	3.409745	3.533805
7908553_PTPRC	5.576538	4.994806	6.012988	5.420911	5.842274	5.211681
7916274_MAGOH	5.94859	5.870215	6.887369	5.772842	6.066812	6.264662
7916564_hCG_2033311	9.465011	8.813292	9.895417	8.436594	8.842843	9.175385
7922773_NCF2	6.020563	5.080857	6.283781	6.401869	6.678098	6.753474
7935116_RBP4	6.147302	5.499371	6.880458	6.164262	5.468297	6.211665
7938286_RPL27A	6.849124	7.362916	6.22969	6.733791	6.73621	7.082227
7949836_CDK2AP2	6.942559	6.634943	7.753766	7.542904	6.683188	6.941648
7952022_AMICA1	5.758945	5.316781	6.414351	5.666541	6.345365	6.20452
7952475_HEPACAM	5.382272	4.689489	5.905793	5.524319	5.013683	5.019866
7953569_PTPN6	5.901713	5.772596	6.880264	6.296437	6.528374	6.532777
7958377_BTBD11	3.134891	3.458119	2.44051	2.837354	2.64908	2.54706
7962827_SNORA2A	6.199946	5.857545	7.095224	5.029156	5.425472	5.045056
7971461_LCP1	7.191066	6.620363	7.690252	7.364239	7.895	7.928597
8000184_IGSF6	5.324913	4.35624	5.359987	5.534385	5.610699	5.71932
8002303_NQO1	6.365284	5.54732	6.71583	6.920881	6.364726	6.241455
8014063_EVI2B	6.461482	5.585223	6.598321	5.848793	6.507542	5.988512
8016832_MMD	6.296847	5.723126	6.901627	6.366689	6.336355	6.167976
8029907_C5AR1	6.40309	5.717925	6.742993	7.107729	6.292155	6.385064
8037947_TPRX1	4.697668	4.002995	5.087213	5.522957	4.782162	4.539182
8043602_NCAPH	4.598221	3.87117	4.983072	4.80072	4.771656	4.655236
8043861_EIF5B	7.298286	7.832332	6.830861	7.091521	8.069132	6.870518
8049512_LRRFIP1	7.535316	8.474	7.282203	8.13838	8.514901	7.543726

Supplemental Table 1. Gene expression values changed greater than 2 fold.

Affymetrix ST 1.0 Array ID	0-No	6-No	12-No	0-Yes	6-Yes	12-Yes
8057418_ZNF533	6.457345	7.102681	6.009701	6.512024	6.434451	6.157969
8058627_ERBB4	6.071224	6.663483	5.546738	6.041313	6.891728	6.219563
8063337_C20orf199	6.648892	6.151326	7.247467	5.873904	6.276644	6.54563
8066256_SNORA71B	8.341427	7.402464	8.459223	8.169294	7.316406	7.459872
8080487_PRKCD	6.178609	5.586181	6.587358	6.348666	6.390004	6.470688
8100971_PPBP	6.266899	5.633088	7.11786	6.786261	6.263447	7.485151
8104901_IL7R	5.482705	4.824535	5.945819	5.497724	5.088688	5.948495
8107208_FER	4.887504	5.426981	4.395261	5.144865	5.950599	4.819022
8115205_LOC134466	3.152577	3.657168	2.365657	3.017302	3.18325	2.531811
8115562_RNF145	7.37993	7.866858	6.588652	7.070046	6.683847	7.335964
8126853_C6orf138	4.756018	4.298548	5.506711	4.519588	5.054243	4.65663
8133688_SNORA14A	6.892104	6.584949	7.753772	6.239049	6.363217	5.877442
8133736_DTX2	6.584027	7.058429	5.744591	6.075387	6.356896	6.487475
8139456_SNORA9	6.184602	5.828887	6.940019	6.866752	6.210551	6.373365
8152976_TMEM71	4.325315	3.831094	5.061284	5.303586	5.224011	5.182084
8154491_ADAMTSL1	4.952783	5.30938	4.204252	4.532811	4.824241	4.494887
8173198_SPIN2A	4.658974	3.84692	4.893922	4.305129	4.053762	4.466052
7902269_SFRS11	8.058014	8.69801	8.490086	8.311749	9.294955	8.207653
7902495_NEXN	9.0054	9.14591	8.5785	9.202801	9.625478	8.411414
7902512_DNAJB4	8.379096	8.673426	8.151655	9.160784	9.336668	8.218173
7908347_OCLM	5.30371	5.912168	5.702179	4.978319	5.804327	4.698595
7908421_TROVE2	6.628878	7.567007	7.303977	7.204431	8.142949	7.017472
7908988_SNRPE	4.532047	5.045445	5.273003	4.515872	5.459489	4.259294
7909188_IKBKE	5.002801	5.872719	5.418964	5.862917	4.883393	6.064266
7911241_OR2L8	4.112454	4.214912	3.921027	4.099971	5.006775	3.973917
7917771_DNTTIP2	5.464234	6.048752	5.941003	6.235038	6.733431	5.562905
7918757_DENND2C	4.75323	4.654508	4.093472	4.101906	4.916145	3.836094
7924773_CDC42BPA	6.732299	7.278711	6.711791	7.038324	7.579326	6.361708
7925500_CHML	4.933205	5.036485	5.311863	4.513386	5.396948	4.386735
7927071_ZNF37A	5.347607	6.18732	5.681369	5.509709	6.270455	5.175431
7929562_ZNF518	5.291873	5.937038	5.154006	5.422691	6.37911	5.309449
7932041_UPF2	5.390244	6.108253	6.009671	5.758636	6.499722	4.857224
7933561_PARG	5.758008	6.489681	5.63127	6.150461	6.684964	5.623684
7946815_PIK3C2A	6.093343	6.776254	6.43158	6.4987	7.33279	6.095909
7951246_MMP8	3.021424	2.961046	3.252901	3.073221	2.675673	3.766381
7951497_NPAT	5.778041	6.753413	6.279309	6.07611	6.969114	5.930123
7953516_LOC283345	5.327782	4.943067	5.344513	5.133263	4.396966	5.656039
7956795_TBK1	5.420481	6.270567	5.98507	5.806534	6.670157	5.46453
7957186_TBC1D15	6.715403	7.461173	7.297563	7.389247	8.043142	6.904494
7957467_C12orf29	5.808215	6.047692	5.728968	5.687613	6.400957	4.786806
7957478_TMTC3	5.513006	6.227687	5.910751	5.900214	6.610492	5.484564
7960158_ZNF10	3.852994	4.722783	4.44486	4.111781	5.049304	4.028467
7969390_C13orf24	4.456624	5.389681	4.653522	4.640877	5.54378	4.508676
7970569_SACS	6.028293	6.998578	6.853607	7.043243	7.626755	6.536224
7970681_RNF6	5.131045	5.786952	4.959812	5.310264	6.036092	4.971852

Supplemental Table 1. Gene expression values changed greater than 2 fold.

Affymetrix ST 1.0 Array ID	0-No	6-No	12-No	0-Yes	6-Yes	12-Yes
7972369_UGCGL2	4.921217	4.830284	4.547744	5.016947	5.501876	4.411236
7972737_LIG4	5.905195	5.834144	6.018853	5.741868	6.510428	5.493795
7978824_RPS29	5.986063	6.088838	6.195913	5.601364	6.596891	5.470304
7982074_SNORD115-34	3.091516	3.535327	2.764527	2.666312	2.561327	3.566593
7989347_NARG2	6.38816	7.281474	6.774491	6.56438	7.53018	6.451237
7994813_ZNF553	5.199757	5.009162	4.733542	4.535932	4.384192	5.518201
8012220_SOX15	6.157141	5.555609	5.720558	5.809566	5.17096	6.198571
8020357_ANKRD30B	2.786907	2.706631	2.421474	2.708497	2.683318	3.686666
8022473_ESCO1	5.445299	6.255452	5.801363	5.699453	6.573318	5.42501
8035078_OR10H1	4.699014	4.877839	4.775903	4.885362	3.919029	5.09437
8035765_ZNF14	4.007627	4.89822	4.29221	5.008063	5.469788	4.151817
8040516_LOC388931	5.466087	4.791608	5.217007	4.783409	5.667187	4.557887
8042588_MPHOSPH10	6.649386	6.81396	6.485282	6.657589	7.516054	6.348776
8044745_DDX18	6.654615	6.549017	6.289347	6.349428	7.278668	6.260171
8045736_FMNL2	6.158519	6.693954	6.754676	6.583711	7.037931	5.891202
8047606_NBEAL1	5.748546	6.557651	5.943195	5.925167	6.842322	5.629183
8056734_TLK1	6.399608	6.542291	6.132291	6.584346	6.976397	5.967146
8056829_CIR	4.687922	4.850116	4.35691	4.535341	5.130107	4.124051
8056995_TTC30B	4.875268	4.733136	4.032271	4.434045	3.885506	5.040171
8065730_EIF2S2	6.850936	6.836804	6.981133	6.992167	7.872191	6.744492
8069553_NRIP1	6.91732	7.828444	7.478179	7.513495	8.351151	7.323709
8080911_KBTBD8	4.681603	4.839803	3.98182	4.341808	3.806969	4.906385
8081503_DZIP3	5.517482	6.357438	5.689601	5.630677	6.584401	5.459328
8083709_SMC4	4.598411	5.505881	5.487716	5.175138	5.949016	4.912626
8087739_CISH	7.574191	7.609294	8.276848	7.804504	7.230013	8.324167
8091354_HLTF	5.139932	5.966952	5.322326	5.563856	6.365039	5.153375
8094596_PTTG2	4.138679	3.850137	3.953598	3.756212	4.744765	3.691856
8098150_FLJ11184	5.068186	5.46532	5.308287	4.740894	5.491275	4.341344
8099410_FAM44A	5.501472	6.488784	6.286801	6.125611	6.619548	5.602072
8100428_CLOCK	5.340608	5.997197	5.694088	6.030426	6.696496	5.618111
8101099_SDAD1	7.020136	7.70005	7.518173	7.45298	8.359432	6.94311
8101659_SPARCL1	9.773297	9.811603	9.689381	10.10939	10.55483	9.251272
8102720_ANKRD50	5.503302	5.46417	4.836031	5.520309	6.00456	4.883116
8104944_NIPBL	6.46168	7.160941	6.702024	6.754832	7.628259	6.512689
8107578_SRFBP1	5.092164	5.891607	5.717923	5.8668	6.622405	5.48444
8113305_CHD1	5.801467	6.31003	6.030627	6.34813	6.983525	5.83744
8113733_CCDC100	6.254193	6.634275	6.338937	6.293478	7.207093	6.101972
8120992_ZNF292	6.618418	7.512275	7.1991	7.152964	8.113394	7.045267
8124540_HIST1H2AM	4.254436	4.07427	4.80918	4.318514	3.647825	5.203175
8127646_FILIP1	7.933805	8.713922	8.801414	8.157308	9.100821	8.068937
8131292_RBAK	5.124869	5.711156	5.196464	5.680796	6.287317	4.735656
8132292_7-Sep	6.798204	7.144979	7.09649	6.627221	7.368629	6.023151
8140967_SAMD9	4.935677	4.304057	4.406515	4.61553	5.345878	4.301241
8141898_ZRF1	5.535722	5.449321	5.298447	5.498161	5.810367	4.770544
8142307_PNPLA8	6.067645	6.277116	6.164386	6.289005	6.922065	5.653557

Supplemental Table 1. Gene expression values changed greater than 2 fold.

Affymetrix ST 1.0 Array ID	0-No	6-No	12-No	0-Yes	6-Yes	12-Yes
8146914_TERF1	6.763456	7.344252	7.086665	6.554656	7.516723	6.417089
8147375_DPY19L4	5.588428	6.079709	6.10104	6.024433	6.683153	5.622528
8147864_TTC35	6.741593	7.470956	7.236151	6.945337	7.883239	6.499072
8149500_MTUS1	7.39842	8.251077	7.968175	7.884776	8.534937	7.386531
8151572_SNX16	4.906993	5.426021	4.461858	5.203623	5.964262	4.899808
8155770_SMC5	5.93434	6.598754	6.873275	6.488413	7.212954	6.063986
8156604_C9orf102	6.454131	6.443021	5.919678	6.141706	7.053525	5.849337
8160274_MGC24103	4.684784	5.575488	5.030373	4.860648	5.84634	4.690299
8163063_CTNNAL1	5.424513	5.692507	5.419939	5.757739	6.216404	5.130279
8166585_FLJ32742	4.166953	3.717347	4.026046	4.149385	4.495986	3.457668
8168412_LOC554203	4.032106	3.741268	3.803451	4.101057	4.461959	3.314328
8177229_BCORL2	3.674929	3.421192	3.221334	3.622908	3.228029	4.592646
8178790_GPSM3	7.249052	7.329778	7.158154	7.351923	6.492415	7.544115
7902205_IL12RB2	4.910226	4.677244	3.97276	3.738513	4.457285	4.340866
7902402_MSH4	4.105139	3.602353	3.79635	3.058167	3.621511	3.753937
7904340_MAN1A2	5.870294	6.540051	6.253984	6.87042	7.099452	6.588976
7906863_UAP1	6.999969	7.290698	7.631621	8.146996	7.422228	7.223425
7915084_YRDC	6.746282	7.379933	7.175331	8.112982	7.418405	7.493714
7917240_CTBS	5.428831	6.425768	6.130455	6.444598	6.411001	5.882316
7917516_GBP1	6.254398	7.104764	6.475442	7.310476	7.046483	6.836342
7922162_SLC19A2	9.215499	9.4685	9.352122	10.26994	9.81531	9.317532
7922391_CENPL	5.597511	4.681274	4.686732	4.595034	4.749337	4.732761
7924499_TLR5	5.53447	5.154692	5.385426	4.52318	5.314676	4.662314
7932069_NUDT5	6.043195	5.377893	5.22705	4.962658	5.305594	5.220944
7938066_OR56A3	5.869269	4.884463	4.941497	4.808119	5.095319	4.678968
7951679_TIMM8B	7.946556	7.148989	7.100851	6.749139	7.020854	7.07529
7952339_LOC85389	8.255534	8.071523	8.300801	6.6892	7.583239	7.262293
7957458_NTS	3.879133	3.266099	3.132003	2.66911	2.851933	2.679615
7962516_SLC38A1	6.681217	7.112739	6.710001	7.712598	6.946417	6.852685
7963421_KRT6A	6.508822	5.801647	6.339546	5.434002	6.191916	5.248709
7969626_GPR180	5.138697	5.745881	5.890325	6.164586	6.169173	5.582413
7971800_THSD1P	5.966171	5.549325	5.178149	4.883745	5.660897	5.437632
7975595_C14orf169	5.819393	6.640982	6.76705	7.003092	6.514961	6.398589
7976488_SERPINA5	4.570154	4.634671	4.998669	5.63132	4.928569	4.655464
7977761_SALL2	5.731482	5.494406	5.565382	4.526176	4.96629	5.035686
7979725_PIGH	4.530631	4.831579	4.790385	5.562866	4.580494	4.800855
7981290_WARS	7.548998	8.356591	8.470029	8.615755	8.620925	8.508198
7982080_SNORD115-37	3.199887	2.321	2.33944	2.114708	2.049078	2.292154
8001133_SHCBP1	5.154345	4.458739	4.748529	4.048142	4.589311	4.46242
8002882_CHST6	6.38816	5.734532	5.684955	5.278222	5.796209	5.689615
8007651_DBF4B	5.068747	5.095736	5.44264	6.387768	5.442768	5.465251
8011817_ZNF232	4.944583	4.54592	4.360731	3.753988	4.033688	4.746636
8013305_ZNF286A	4.376273	4.383988	3.672078	3.307788	3.97606	4.022529
8014233_SLFN11	5.923963	6.227578	6.002807	6.967628	6.247658	6.29668
8021101_CCDC5	5.264147	4.617264	4.990039	3.913537	4.784978	4.366481

Supplemental Table 1. Gene expression values changed greater than 2 fold.

Affymetrix ST 1.0 Array ID	0-No	6-No	12-No	0-Yes	6-Yes	12-Yes
8025132_ZNF557	5.734728	4.962721	5.319806	4.523612	4.725121	5.209182
8029521_TOMM40	6.373537	7.31145	7.287697	7.69446	7.355124	7.604729
8030364_SNORD34	7.89135	8.150539	8.090441	6.837142	7.284775	7.432178
8038952_ZNF616	4.453373	4.198038	4.367416	3.162956	3.861324	3.375981
8039070_ZNF677	4.361943	3.920511	3.527169	3.207803	3.430795	3.474964
8041676_DYNC2LI1	5.318759	4.734391	4.662615	4.266717	4.762931	4.664197
8042830_MTHFD2	5.965271	6.585169	6.625826	7.004755	7.053346	6.416486
8047372_NDUFB3	7.119532	6.583358	6.575161	6.072522	6.416421	6.819357
8057719_HIBCH	5.288493	6.241881	6.043421	6.324776	7.03158	6.249231
8060061_MGC16025	5.914419	6.256901	6.582634	7.023474	6.570559	6.787555
8061772_MAPRE1	6.852678	7.791052	7.74513	7.951971	8.09269	7.88245
8062844_C20orf121	5.46495	4.875836	5.01098	4.455909	5.034282	4.63404
8065359_CD93	7.496609	7.588668	7.840246	8.562429	7.904389	7.846471
8071063_psiTPTE22	6.150762	5.568584	5.657795	5.109697	5.407076	5.76313
8073015_KDELR3	4.116615	4.588433	4.83415	5.168755	5.032486	4.592786
8074980_GSTT1	8.309097	8.608227	8.626511	6.963013	7.912552	7.955463
8074988_GSTTP2	3.140983	2.748051	2.857618	4.284444	3.554478	3.69591
8075263_NIPSNAP1	5.639699	6.150651	6.322981	6.881332	5.906935	5.919979
8081212_OR5K2	4.247553	3.631904	3.363466	3.169774	3.484435	4.023287
8084717_ST6GAL1	6.38261	7.246094	7.301609	7.543224	7.211827	6.961212
8085360_TIMP4	6.520971	7.438359	7.389585	7.864416	7.052883	7.59166
8086600_CCR1	4.934343	4.09213	4.151952	3.663947	4.126647	4.212101
8089478_GCET2	5.642399	4.967829	5.173186	4.540956	4.793259	5.03908
8091097_FLJ11827	3.988832	3.477561	3.904458	2.936614	3.877854	3.110346
8091283_PLOD2	4.700873	4.522608	4.486065	5.852301	5.867484	5.331513
8096635_NFKB1	6.069341	6.796298	6.684219	7.11553	7.139607	7.06443
8098423_NEIL3	4.812866	3.845269	4.099957	3.765018	4.460342	4.530239
8102232_LEF1	5.774014	5.389676	5.503075	4.716031	4.95889	4.855385
8105842_CENPH	4.262029	3.581479	3.456239	3.078197	3.247606	3.522506
8106743_VCAN	6.907185	6.684074	6.824775	7.978148	6.982431	7.027285
8108905_KCTD16	4.910405	4.172378	4.218393	3.866436	4.020811	4.012193
8110734_BRD9	5.573647	6.51757	6.364727	6.69581	6.33758	6.590953
8111533_LMBRD2	5.975042	6.791167	6.811629	7.051183	7.339872	6.715341
8113010_CCNH	5.669617	6.094415	6.551258	6.675352	7.052556	6.644341
8115783_STK10	5.694911	6.550594	6.228703	6.735902	6.578838	6.982697
8117368_HIST1H4C	8.937176	8.740905	9.100365	7.741168	8.395145	8.464793
8122720_ULBP2	5.65801	4.762881	4.939142	4.439057	4.549761	4.699197
8123598_SERPINB1	6.47923	6.262108	6.534543	7.543447	6.737503	6.724758
8124057_LOC728380	5.088809	5.236048	4.704109	4.055841	4.595932	4.923005
8124394_HIST1H2BB	5.726523	4.75273	4.955581	4.708552	4.565875	4.731306
8124689_HCG4	6.663049	5.922561	6.282541	5.652083	5.423854	6.057853
8124862_CDSN	6.964617	6.172195	6.384141	5.950365	6.258126	6.103176
8127743_HMGN3	8.060406	7.746344	8.177895	6.978148	7.887566	7.819032
8130556_SOD2	8.697921	8.926036	9.116282	9.827279	9.206861	9.147647
8130785_GPR31	4.912836	5.667531	5.438448	6.047609	5.536143	5.6991

Supplemental Table 1. Gene expression values changed greater than 2 fold.

Affymetrix ST 1.0 Array ID	0-No	6-No	12-No	0-Yes	6-Yes	12-Yes
8134965_SLC12A9	6.299356	5.858843	6.064877	5.28623	5.593943	5.820357
8135080_AP1S1	6.740369	6.165377	6.230564	5.495177	5.778252	6.416267
8138363_SOSTDC1	4.725197	3.914511	3.813599	3.371703	3.440812	3.534125
8138370_ANKMY2	5.734189	6.692513	6.683413	6.804168	7.256014	6.93894
8138381_AGR2	4.301423	3.666625	3.334815	3.255311	3.619537	3.688076
8138602_DFNA5	4.556494	5.304986	5.180137	5.639797	4.763845	4.662866
8140859_MTERF	3.980108	4.76884	4.568933	5.238859	5.341544	4.349953
8146517_CHCHD7	5.547689	4.804543	5.38792	4.494511	4.985006	5.173479
8157650_PTGS1	5.277689	5.677121	5.611978	6.378138	6.188022	6.288982
8162183_LOC440173	3.308142	4.269224	4.034333	4.746659	4.184363	4.076488
8169473_PLS3	6.418516	7.371739	7.166112	7.514426	7.900375	7.401042
8170811_OPN1LW	4.502118	3.998219	3.720083	3.231482	3.506878	3.561257
8174103_GK	5.680833	6.263852	6.459292	6.925839	6.77519	6.241859
8176624_DDX3Y	4.948363	5.758093	5.51553	6.645794	6.77763	6.357315
8176719_EIF1AY	5.007949	5.445361	5.278837	6.491919	6.530573	6.878759
8177137_UTY	3.834804	4.465686	4.661743	5.059858	5.010483	5.227437
8178086_HSPA1B	9.425415	9.566379	9.569242	10.48545	9.660233	9.716578
8179324_HSPA1B	9.425415	9.566379	9.569242	10.48545	9.660233	9.716578
8179716_CDSN	6.824461	5.870612	6.214918	5.787617	6.048513	5.863868
7903765_GSTM1	6.770406	7.378471	7.628773	7.702847	8.619015	8.53059
7911273_OR2T4	4.147927	4.427365	3.623215	3.808225	3.370496	3.347394
7924150_C1orf75	4.992841	4.533233	5.225661	4.886209	5.636861	4.988083
7930299_C10orf78	3.456906	4.429543	4.077724	3.581077	3.346405	4.068935
7941662_ACTN3	10.8109	11.02431	10.82226	9.829544	9.817282	10.07737
7970864_HSPH1	6.570158	6.363878	6.540071	7.27989	7.401081	6.565679
7971602_RCBTB1	5.744056	4.98529	5.930161	5.526038	6.128827	5.375954
7974697_DAAM1	5.096935	5.261114	5.734886	5.880795	6.452676	5.973146
7981783_OR4N4	3.204796	3.28965	3.279342	2.488423	2.250976	2.827697
8014755_SNORA21	8.421598	9.215389	8.420652	8.569211	8.015314	8.419765
8067864_hCG_2042718	5.369768	5.521052	6.256417	6.23241	6.526452	6.270103
8077366_LRRN1	5.596999	5.450545	5.475056	6.458336	6.509317	5.590274
8082583_UNQ6125	3.945428	4.511432	3.557774	3.899896	3.381407	4.100138
8096919_ALPK1	4.644395	4.34233	4.944327	5.167202	5.705191	4.989498
8100202_CNGA1	3.327366	2.47912	3.324215	3.205874	4.0166	3.146578
8146500_LYN	5.754592	4.989699	5.869798	5.940371	6.272357	6.135805
7926896_CKS1B	6.449512	6.300798	6.967236	5.925105	6.247098	5.843976
7929653_ANKRD2	9.536079	8.976337	8.614109	10.2677	9.691982	9.761154
7938293_SNORA45	5.923302	6.016823	6.916877	5.961954	6.007673	5.912234
7942957_PRSS23	6.619503	7.010416	7.191851	6.112556	6.114112	5.994169
7943369_TMEM133	4.837653	4.651522	5.103943	4.452871	4.532468	3.941669
7955045_FAM113B	5.53474	5.178879	4.642554	5.359487	5.103673	5.708636
7957806_SCYL2	5.714338	6.408989	5.794208	6.388804	6.956963	6.930677
7980828_CCDC88C	5.311304	5.582697	5.556788	4.604488	4.891204	4.31978
7984813_ISLR	6.403723	6.340218	5.732573	6.780717	6.47141	6.978498
7998664_SNORA10	8.000218	7.412336	8.167609	8.035073	7.148111	7.151623

Supplemental Table 1. Gene expression values changed greater than 2 fold.

Affymetrix ST 1.0 Array ID	0-No	6-No	12-No	0-Yes	6-Yes	12-Yes
8004508_SNORA67	6.40535	6.888958	7.257342	6.392427	6.296631	6.191295
8012953_TRIM16	6.595873	7.309455	6.431998	7.410591	7.540643	7.59816
8023392_SNORA37	6.388687	6.326095	7.117679	6.002793	6.176358	5.971879
8025968_ZNF69	6.185996	5.843741	6.284183	6.094353	5.947633	5.207095
8041204_SNORA10	8.000218	7.412336	8.167609	8.035073	7.148111	7.151623
8049297_SCARNA5	9.764841	9.345344	10.31604	8.965115	9.042504	9.154562
8063955_OGFR	7.525732	8.232588	7.620166	8.143153	7.920815	8.67404
8067932_C21orf34	4.187754	4.679426	3.859073	4.172245	4.477819	4.942496
8078918_SNORA62	7.51971	7.281384	8.218526	6.808687	6.836918	5.998769
8108693_PCDHB4	3.690004	3.673922	2.907531	3.636351	4.399466	4.27713
8112327_CKS1B	6.379407	6.241948	6.916185	6.010139	6.241906	5.915573
8126760_RCAN2	7.566139	7.380196	6.585502	7.83314	7.499754	7.598533
8127423_SNORD3B-1	10.84592	10.50556	11.2099	10.25987	9.850414	10.05573
8127987_SNORD50A	8.107223	9.041314	8.826312	7.378601	8.341829	7.54991
8145603_PNOC	5.887107	5.55401	4.928079	6.016139	5.467471	6.069554
8176375_RPS4Y1	5.758789	5.226935	5.532979	5.787913	5.761269	6.577894
8177232_JARID1D	5.143709	5.7077	5.084393	5.680796	5.939805	6.279619

Supplemental Table 1. Gene expression values changed greater than 2 fold.

Affymetrix ST 1.0 Array ID	0-No	6-No	12-No	0-Yes	6-Yes	12-Yes

Supplemental Table 1. Gene expression values changed greater than 2 fold.

Affymetrix ST 1.0 Array ID	0-No	6-No	12-No	0-Yes	6-Yes	12-Yes

Supplemental Table 1. Gene expression values changed greater than 2 fold.

Affymetrix ST 1.0 Array ID	0-No	6-No	12-No	0-Yes	6-Yes	12-Yes

Supplemental Table 1. Gene expression values changed greater than 2 fold.

Affymetrix ST 1.0 Array ID	0-No	6-No	12-No	0-Yes	6-Yes	12-Yes

Supplemental Table 1. Gene expression values changed greater than 2 fold.

Affymetrix ST 1.0 Array ID	0-No	6-No	12-No	0-Yes	6-Yes	12-Yes

Supplemental Table 1. Gene expression values changed greater than 2 fold.

Affymetrix ST 1.0 Array ID	0-No	6-No	12-No	0-Yes	6-Yes	12-Yes

Supplemental Table 1. Gene expression values changed greater than 2 fold.

Affymetrix ST 1.0 Array ID	0-No	6-No	12-No	0-Yes	6-Yes	12-Yes

Supplemental Table 1. Gene expression values changed greater than 2 fold.

Affymetrix ST 1.0 Array ID	0-No	6-No	12-No	0-Yes	6-Yes	12-Yes

Supplemental Table 1. Gene expression values changed greater than 2 fold.

Affymetrix ST 1.0 Array ID	0-No	6-No	12-No	0-Yes	6-Yes	12-Yes

Supplemental Table 1. Gene expression values changed greater than 2 fold.

Affymetrix ST 1.0 Array ID	0-No	6-No	12-No	0-Yes	6-Yes	12-Yes

Supplemental Table 1. Gene expression values changed greater than 2 fold.

Affymetrix ST 1.0 Array ID	0-No	6-No	12-No	0-Yes	6-Yes	12-Yes

Supplemental Table 1. Gene expression values changed greater than 2 fold.

Affymetrix ST 1.0 Array ID	0-No	6-No	12-No	0-Yes	6-Yes	12-Yes

Supplemental Table 1. Gene expression values changed greater than 2 fold.

Affymetrix ST 1.0 Array ID	0-No	6-No	12-No	0-Yes	6-Yes	12-Yes

Supplemental Table 1. Gene expression values changed greater than 2 fold.

Affymetrix ST 1.0 Array ID	0-No	6-No	12-No	0-Yes	6-Yes	12-Yes

Supplemental Table 1. Gene expression values changed greater than 2 fold.

Affymetrix ST 1.0 Array ID	0-No	6-No	12-No	0-Yes	6-Yes	12-Yes

Supplemental Table 1. Gene expression values changed greater than 2 fold.

Affymetrix ST 1.0 Array ID	0-No	6-No	12-No	0-Yes	6-Yes	12-Yes

Supplemental Table 1. Gene expression values changed greater than 2 fold.

Affymetrix ST 1.0 Array ID	0-No	6-No	12-No	0-Yes	6-Yes	12-Yes

Supplemental Table 1. Gene expression values changed greater than 2 fold.

Affymetrix ST 1.0 Array ID	0-No	6-No	12-No	0-Yes	6-Yes	12-Yes

Supplemental Table 1. Gene expression values changed greater than 2 fold.

Affymetrix ST 1.0 Array ID	0-No	6-No	12-No	0-Yes	6-Yes	12-Yes

Supplemental Table 1. Gene expression values changed greater than 2 fold.

Affymetrix ST 1.0 Array ID	0-No	6-No	12-No	0-Yes	6-Yes	12-Yes

Supplemental Table 1. Gene expression values changed greater than 2 fold.

Affymetrix ST 1.0 Array ID	0-No	6-No	12-No	0-Yes	6-Yes	12-Yes

Supplemental Table 1. Gene expression values changed greater than 2 fold.

Affymetrix ST 1.0 Array ID	0-No	6-No	12-No	0-Yes	6-Yes	12-Yes

Supplemental Table 1. Gene expression values changed greater than 2 fold.

Affymetrix ST 1.0 Array ID	0-No	6-No	12-No	0-Yes	6-Yes	12-Yes

Supplemental Table 1. Gene expression values changed greater than 2 fold.

Affymetrix ST 1.0 Array ID	0-No	6-No	12-No	0-Yes	6-Yes	12-Yes

Supplemental Table 1. Gene expression values changed greater than 2 fold.

Affymetrix ST 1.0 Array ID	0-No	6-No	12-No	0-Yes	6-Yes	12-Yes

Supplemental Table 1. Gene expression values changed greater than 2 fold.

Affymetrix ST 1.0 Array ID	0-No	6-No	12-No	0-Yes	6-Yes	12-Yes

Supplemental Table 1. Gene expression values changed greater than 2 fold.

Affymetrix ST 1.0 Array ID	0-No	6-No	12-No	0-Yes	6-Yes	12-Yes

Supplemental Table 1. Gene expression values changed greater than 2 fold.

Affymetrix ST 1.0 Array ID	0-No	6-No	12-No	0-Yes	6-Yes	12-Yes

Supplemental Table 1. Gene expression values changed greater than 2 fold.

Affymetrix ST 1.0 Array ID	0-No	6-No	12-No	0-Yes	6-Yes	12-Yes

Supplemental Table 1. Gene expression values changed greater than 2 fold.

Affymetrix ST 1.0 Array ID	0-No	6-No	12-No	0-Yes	6-Yes	12-Yes

Supplemental Table 1. Gene expression values changed greater than 2 fold.

Affymetrix ST 1.0 Array ID	0-No	6-No	12-No	0-Yes	6-Yes	12-Yes

Supplemental Table 1. Gene expression values changed greater than 2 fold.

Affymetrix ST 1.0 Array ID	0-No	6-No	12-No	0-Yes	6-Yes	12-Yes

Supplemental Table 1. Gene expression values changed greater than 2 fold.

Affymetrix ST 1.0 Array ID	0-No	6-No	12-No	0-Yes	6-Yes	12-Yes

Supplemental Table 1. Gene expression values changed greater than 2 fold.

Affymetrix ST 1.0 Array ID	0-No	6-No	12-No	0-Yes	6-Yes	12-Yes

Supplemental Table 1. Gene expression values changed greater than 2 fold.

Affymetrix ST 1.0 Array ID	0-No	6-No	12-No	0-Yes	6-Yes	12-Yes

Supplemental Table 1. Gene expression values changed greater than 2 fold.

Affymetrix ST 1.0 Array ID	0-No	6-No	12-No	0-Yes	6-Yes	12-Yes

Supplemental Table 1. Gene expression values changed greater than 2 fold.

Affymetrix ST 1.0 Array ID	0-No	6-No	12-No	0-Yes	6-Yes	12-Yes

Supplemental Table 1. Gene expression values changed greater than 2 fold.

Affymetrix ST 1.0 Array ID	0-No	6-No	12-No	0-Yes	6-Yes	12-Yes

Supplemental Table 1. Gene expression values changed greater than 2 fold.

Affymetrix ST 1.0 Array ID	0-No	6-No	12-No	0-Yes	6-Yes	12-Yes

Supplemental Table 1. Gene expression values changed greater than 2 fold.

Affymetrix ST 1.0 Array ID	0-No	6-No	12-No	0-Yes	6-Yes	12-Yes

Supplemental Table 1. Gene expression values changed greater than 2 fold.

Affymetrix ST 1.0 Array ID	0-No	6-No	12-No	0-Yes	6-Yes	12-Yes

Supplemental Table 1. Gene expression values changed greater than 2 fold.

Affymetrix ST 1.0 Array ID	0-No	6-No	12-No	0-Yes	6-Yes	12-Yes

Supplemental Table 1. Gene expression values changed greater than 2 fold.

Affymetrix ST 1.0 Array ID	0-No	6-No	12-No	0-Yes	6-Yes	12-Yes

Supplemental Table 1. Gene expression values changed greater than 2 fold.

Affymetrix ST 1.0 Array ID	0-No	6-No	12-No	0-Yes	6-Yes	12-Yes

Supplemental Table 1. Gene expression values changed greater than 2 fold.

Affymetrix ST 1.0 Array ID	0-No	6-No	12-No	0-Yes	6-Yes	12-Yes

Supplemental Table 1. Gene expression values changed greater than 2 fold.

Affymetrix ST 1.0 Array ID	0-No	6-No	12-No	0-Yes	6-Yes	12-Yes

Supplemental Table 1. Gene expression values changed greater than 2 fold.

Affymetrix ST 1.0 Array ID	0-No	6-No	12-No	0-Yes	6-Yes	12-Yes

Supplemental Table 1. Gene expression values changed greater than 2 fold.

Affymetrix ST 1.0 Array ID	0-No	6-No	12-No	0-Yes	6-Yes	12-Yes

Supplemental Table 1. Gene expression values changed greater than 2 fold.

Affymetrix ST 1.0 Array ID	0-No	6-No	12-No	0-Yes	6-Yes	12-Yes

Supplemental Table 1. Gene expression values changed greater than 2 fold.

Affymetrix ST 1.0 Array ID	0-No	6-No	12-No	0-Yes	6-Yes	12-Yes

Supplemental Table 1. Gene expression values changed greater than 2 fold.

Affymetrix ST 1.0 Array ID	0-No	6-No	12-No	0-Yes	6-Yes	12-Yes

Supplemental Table 1. Gene expression values changed greater than 2 fold.

Affymetrix ST 1.0 Array ID	0-No	6-No	12-No	0-Yes	6-Yes	12-Yes

Supplemental Table 1. Gene expression values changed greater than 2 fold.

Affymetrix ST 1.0 Array ID	0-No	6-No	12-No	0-Yes	6-Yes	12-Yes

Supplemental Table 1. Gene expression values changed greater than 2 fold.

Affymetrix ST 1.0 Array ID	0-No	6-No	12-No	0-Yes	6-Yes	12-Yes

Supplemental Table 1. Gene expression values changed greater than 2 fold.

Affymetrix ST 1.0 Array ID	0-No	6-No	12-No	0-Yes	6-Yes	12-Yes

Supplemental Table 1. Gene expression values changed greater than 2 fold.

Affymetrix ST 1.0 Array ID	0-No	6-No	12-No	0-Yes	6-Yes	12-Yes

Supplemental Table 1. Gene expression values changed greater than 2 fold.

Affymetrix ST 1.0 Array ID	0-No	6-No	12-No	0-Yes	6-Yes	12-Yes

Supplemental Table 1. Gene expression values changed greater than 2 fold.

Affymetrix ST 1.0 Array ID	0-No	6-No	12-No	0-Yes	6-Yes	12-Yes

Supplemental Table 1. Gene expression values changed greater than 2 fold.

Affymetrix ST 1.0 Array ID	0-No	6-No	12-No	0-Yes	6-Yes	12-Yes

Supplemental Table 1. Gene expression values changed greater than 2 fold.

Affymetrix ST 1.0 Array ID	0-No	6-No	12-No	0-Yes	6-Yes	12-Yes

Supplemental Table 1. Gene expression values changed greater than 2 fold.

Affymetrix ST 1.0 Array ID	0-No	6-No	12-No	0-Yes	6-Yes	12-Yes

Supplemental Table 1. Gene expression values changed greater than 2 fold.

Affymetrix ST 1.0 Array ID	0-No	6-No	12-No	0-Yes	6-Yes	12-Yes

Supplemental Table 1. Gene expression values changed greater than 2 fold.

Affymetrix ST 1.0 Array ID	0-No	6-No	12-No	0-Yes	6-Yes	12-Yes

Supplemental Table 1. Gene expression values changed greater than 2 fold.

Affymetrix ST 1.0 Array ID	0-No	6-No	12-No	0-Yes	6-Yes	12-Yes

Supplemental Table 1. Gene expression values changed greater than 2 fold.

Affymetrix ST 1.0 Array ID	0-No	6-No	12-No	0-Yes	6-Yes	12-Yes

Supplemental Table 1. Gene expression values changed greater than 2 fold.

Affymetrix ST 1.0 Array ID	0-No	6-No	12-No	0-Yes	6-Yes	12-Yes

Supplemental Table 1. Gene expression values changed greater than 2 fold.

Affymetrix ST 1.0 Array ID	0-No	6-No	12-No	0-Yes	6-Yes	12-Yes

Supplemental Table 1. Gene expression values changed greater than 2 fold.

Affymetrix ST 1.0 Array ID	0-No	6-No	12-No	0-Yes	6-Yes	12-Yes

Supplemental Table 1. Gene expression values changed greater than 2 fold.

Affymetrix ST 1.0 Array ID	0-No	6-No	12-No	0-Yes	6-Yes	12-Yes

Supplemental Table 1. Gene expression values changed greater than 2 fold.

Affymetrix ST 1.0 Array ID	0-No	6-No	12-No	0-Yes	6-Yes	12-Yes

Supplemental Table 1. Gene expression values changed greater than 2 fold.

Affymetrix ST 1.0 Array ID	0-No	6-No	12-No	0-Yes	6-Yes	12-Yes

Supplemental Table 1. Gene expression values changed greater than 2 fold.

Affymetrix ST 1.0 Array ID	0-No	6-No	12-No	0-Yes	6-Yes	12-Yes

Supplemental Table 1. Gene expression values changed greater than 2 fold.

Affymetrix ST 1.0 Array ID	0-No	6-No	12-No	0-Yes	6-Yes	12-Yes

Supplemental Table 1. Gene expression values changed greater than 2 fold.

Affymetrix ST 1.0 Array ID	0-No	6-No	12-No	0-Yes	6-Yes	12-Yes

Supplemental Table 1. Gene expression values changed greater than 2 fold.

Affymetrix ST 1.0 Array ID	0-No	6-No	12-No	0-Yes	6-Yes	12-Yes

Supplemental Table 1. Gene expression values changed greater than 2 fold.

Affymetrix ST 1.0 Array ID	0-No	6-No	12-No	0-Yes	6-Yes	12-Yes

Supplemental Table 1. Gene expression values changed greater than 2 fold.

Affymetrix ST 1.0 Array ID	0-No	6-No	12-No	0-Yes	6-Yes	12-Yes

Supplemental Table 1. Gene expression values changed greater than 2 fold.

Affymetrix ST 1.0 Array ID	0-No	6-No	12-No	0-Yes	6-Yes	12-Yes

Supplemental Table 1. Gene expression values changed greater than 2 fold.

Affymetrix ST 1.0 Array ID	0-No	6-No	12-No	0-Yes	6-Yes	12-Yes

Supplemental Table 1. Gene expression values changed greater than 2 fold.

Affymetrix ST 1.0 Array ID	0-No	6-No	12-No	0-Yes	6-Yes	12-Yes

Supplemental Table 1. Gene expression values changed greater than 2 fold.

Affymetrix ST 1.0 Array ID	0-No	6-No	12-No	0-Yes	6-Yes	12-Yes

Supplemental Table 1. Gene expression values changed greater than 2 fold.

Affymetrix ST 1.0 Array ID	0-No	6-No	12-No	0-Yes	6-Yes	12-Yes

Supplemental Table 1. Gene expression values changed greater than 2 fold.

Affymetrix ST 1.0 Array ID	0-No	6-No	12-No	0-Yes	6-Yes	12-Yes

Supplemental Table 1. Gene expression values changed greater than 2 fold.

Affymetrix ST 1.0 Array ID	0-No	6-No	12-No	0-Yes	6-Yes	12-Yes

Supplemental Table 1. Gene expression values changed greater than 2 fold.

Affymetrix ST 1.0 Array ID	0-No	6-No	12-No	0-Yes	6-Yes	12-Yes

Supplemental Table 1. Gene expression values changed greater than 2 fold.

Affymetrix ST 1.0 Array ID	0-No	6-No	12-No	0-Yes	6-Yes	12-Yes

Supplemental Table 1. Gene expression values changed greater than 2 fold.

Affymetrix ST 1.0 Array ID	0-No	6-No	12-No	0-Yes	6-Yes	12-Yes

Supplemental Table 1. Gene expression values changed greater than 2 fold.

Affymetrix ST 1.0 Array ID	0-No	6-No	12-No	0-Yes	6-Yes	12-Yes

Supplemental Table 1. Gene expression values changed greater than 2 fold.

Affymetrix ST 1.0 Array ID	0-No	6-No	12-No	0-Yes	6-Yes	12-Yes

Supplemental Table 1. Gene expression values changed greater than 2 fold.

Affymetrix ST 1.0 Array ID	0-No	6-No	12-No	0-Yes	6-Yes	12-Yes

Supplemental Table 1. Gene expression values changed greater than 2 fold.

Affymetrix ST 1.0 Array ID	0-No	6-No	12-No	0-Yes	6-Yes	12-Yes

Supplemental Table 1. Gene expression values changed greater than 2 fold.

Affymetrix ST 1.0 Array ID	0-No	6-No	12-No	0-Yes	6-Yes	12-Yes

Supplemental Table 1. Gene expression values changed greater than 2 fold.

Affymetrix ST 1.0 Array ID	0-No	6-No	12-No	0-Yes	6-Yes	12-Yes

Supplemental Table 1. Gene expression values changed greater than 2 fold.

Affymetrix ST 1.0 Array ID	0-No	6-No	12-No	0-Yes	6-Yes	12-Yes

Supplemental Table 1. Gene expression values changed greater than 2 fold.

Affymetrix ST 1.0 Array ID	0-No	6-No	12-No	0-Yes	6-Yes	12-Yes

Supplemental Table 1. Gene expression values changed greater than 2 fold.

Affymetrix ST 1.0 Array ID	0-No	6-No	12-No	0-Yes	6-Yes	12-Yes

Supplemental Table 1. Gene expression values changed greater than 2 fold.

Affymetrix ST 1.0 Array ID	0-No	6-No	12-No	0-Yes	6-Yes	12-Yes

Supplemental Table 1. Gene expression values changed greater than 2 fold.

Affymetrix ST 1.0 Array ID	0-No	6-No	12-No	0-Yes	6-Yes	12-Yes

Supplemental Table 1. Gene expression values changed greater than 2 fold.

Affymetrix ST 1.0 Array ID	0-No	6-No	12-No	0-Yes	6-Yes	12-Yes

Supplemental Table 1. Gene expression values changed greater than 2 fold.

Affymetrix ST 1.0 Array ID	0-No	6-No	12-No	0-Yes	6-Yes	12-Yes

Supplemental Table 1. Gene expression values changed greater than 2 fold.

Affymetrix ST 1.0 Array ID	0-No	6-No	12-No	0-Yes	6-Yes	12-Yes

Supplemental Table 1. Gene expression values changed greater than 2 fold.

Affymetrix ST 1.0 Array ID	0-No	6-No	12-No	0-Yes	6-Yes	12-Yes

Supplemental Table 1. Gene expression values changed greater than 2 fold.

Affymetrix ST 1.0 Array ID	0-No	6-No	12-No	0-Yes	6-Yes	12-Yes

Supplemental Table 1. Gene expression values changed greater than 2 fold.

Affymetrix ST 1.0 Array ID	0-No	6-No	12-No	0-Yes	6-Yes	12-Yes

Supplemental Table 1. Gene expression values changed greater than 2 fold.

Affymetrix ST 1.0 Array ID	0-No	6-No	12-No	0-Yes	6-Yes	12-Yes

Supplemental Table 1. Gene expression values changed greater than 2 fold.

Affymetrix ST 1.0 Array ID	0-No	6-No	12-No	0-Yes	6-Yes	12-Yes

Supplemental Table 1. Gene expression values changed greater than 2 fold.

Affymetrix ST 1.0 Array ID	0-No	6-No	12-No	0-Yes	6-Yes	12-Yes

Supplemental Table 1. Gene expression values changed greater than 2 fold.

Affymetrix ST 1.0 Array ID	0-No	6-No	12-No	0-Yes	6-Yes	12-Yes

Supplemental Table 1. Gene expression values changed greater than 2 fold.

Affymetrix ST 1.0 Array ID	0-No	6-No	12-No	0-Yes	6-Yes	12-Yes

Supplemental Table 1. Gene expression values changed greater than 2 fold.

Affymetrix ST 1.0 Array ID	0-No	6-No	12-No	0-Yes	6-Yes	12-Yes

Supplemental Table 1. Gene expression values changed greater than 2 fold.

Affymetrix ST 1.0 Array ID	0-No	6-No	12-No	0-Yes	6-Yes	12-Yes

Supplemental Table 1. Gene expression values changed greater than 2 fold.

Affymetrix ST 1.0 Array ID	0-No	6-No	12-No	0-Yes	6-Yes	12-Yes

Supplemental Table 1. Gene expression values changed greater than 2 fold.

Affymetrix ST 1.0 Array ID	0-No	6-No	12-No	0-Yes	6-Yes	12-Yes

Supplemental Table 1. Gene expression values changed greater than 2 fold.

Affymetrix ST 1.0 Array ID	0-No	6-No	12-No	0-Yes	6-Yes	12-Yes

Supplemental Table 1. Gene expression values changed greater than 2 fold.

Affymetrix ST 1.0 Array ID	0-No	6-No	12-No	0-Yes	6-Yes	12-Yes

Supplemental Table 1. Gene expression values changed greater than 2 fold.

Affymetrix ST 1.0 Array ID	0-No	6-No	12-No	0-Yes	6-Yes	12-Yes

Supplemental Table 1. Gene expression values changed greater than 2 fold.

Affymetrix ST 1.0 Array ID	0-No	6-No	12-No	0-Yes	6-Yes	12-Yes

Supplemental Table 1. Gene expression values changed greater than 2 fold.

Affymetrix ST 1.0 Array ID	0-No	6-No	12-No	0-Yes	6-Yes	12-Yes

Supplemental Table 1. Gene expression values changed greater than 2 fold.

Affymetrix ST 1.0 Array ID	0-No	6-No	12-No	0-Yes	6-Yes	12-Yes

Supplemental Table 1. Gene expression values changed greater than 2 fold.

Affymetrix ST 1.0 Array ID	0-No	6-No	12-No	0-Yes	6-Yes	12-Yes

Supplemental Table 1. Gene expression values changed greater than 2 fold.

Affymetrix ST 1.0 Array ID	0-No	6-No	12-No	0-Yes	6-Yes	12-Yes

Supplemental Table 1. Gene expression values changed greater than 2 fold.

Affymetrix ST 1.0 Array ID	0-No	6-No	12-No	0-Yes	6-Yes	12-Yes

Supplemental Table 1. Gene expression values changed greater than 2 fold.

Affymetrix ST 1.0 Array ID	0-No	6-No	12-No	0-Yes	6-Yes	12-Yes

Supplemental Table 1. Gene expression values changed greater than 2 fold.

Affymetrix ST 1.0 Array ID	0-No	6-No	12-No	0-Yes	6-Yes	12-Yes

Supplemental Table 1. Gene expression values changed greater than 2 fold.

Affymetrix ST 1.0 Array ID	0-No	6-No	12-No	0-Yes	6-Yes	12-Yes

Supplemental Table 1. Gene expression values changed greater than 2 fold.

Affymetrix ST 1.0 Array ID	0-No	6-No	12-No	0-Yes	6-Yes	12-Yes

Supplemental Table 1. Gene expression values changed greater than 2 fold.

Affymetrix ST 1.0 Array ID	0-No	6-No	12-No	0-Yes	6-Yes	12-Yes

Supplemental Table 1. Gene expression values changed greater than 2 fold.

Affymetrix ST 1.0 Array ID	0-No	6-No	12-No	0-Yes	6-Yes	12-Yes

Supplemental Table 1. Gene expression values changed greater than 2 fold.

Affymetrix ST 1.0 Array ID	0-No	6-No	12-No	0-Yes	6-Yes	12-Yes

Supplemental Table 1. Gene expression values changed greater than 2 fold.

Affymetrix ST 1.0 Array ID	0-No	6-No	12-No	0-Yes	6-Yes	12-Yes

Supplemental Table 1. Gene expression values changed greater than 2 fold.

Affymetrix ST 1.0 Array ID	0-No	6-No	12-No	0-Yes	6-Yes	12-Yes

Supplemental Table 1. Gene expression values changed greater than 2 fold.

Affymetrix ST 1.0 Array ID	0-No	6-No	12-No	0-Yes	6-Yes	12-Yes

Supplemental Table 1. Gene expression values changed greater than 2 fold.

Affymetrix ST 1.0 Array ID	0-No	6-No	12-No	0-Yes	6-Yes	12-Yes

Supplemental Table 1. Gene expression values changed greater than 2 fold.

Affymetrix ST 1.0 Array ID	0-No	6-No	12-No	0-Yes	6-Yes	12-Yes

Supplemental Table 1. Gene expression values changed greater than 2 fold.

Affymetrix ST 1.0 Array ID	0-No	6-No	12-No	0-Yes	6-Yes	12-Yes

Supplemental Table 1. Gene expression values changed greater than 2 fold.

Affymetrix ST 1.0 Array ID	0-No	6-No	12-No	0-Yes	6-Yes	12-Yes

Supplemental Table 1. Gene expression values changed greater than 2 fold.

Affymetrix ST 1.0 Array ID	0-No	6-No	12-No	0-Yes	6-Yes	12-Yes

Supplemental Table 1. Gene expression values changed greater than 2 fold.

Affymetrix ST 1.0 Array ID	0-No	6-No	12-No	0-Yes	6-Yes	12-Yes

Supplemental Table 1. Gene expression values changed greater than 2 fold.

Affymetrix ST 1.0 Array ID	0-No	6-No	12-No	0-Yes	6-Yes	12-Yes

Supplemental Table 1. Gene expression values changed greater than 2 fold.

Affymetrix ST 1.0 Array ID	0-No	6-No	12-No	0-Yes	6-Yes	12-Yes

Supplemental Table 1. Gene expression values changed greater than 2 fold.

Affymetrix ST 1.0 Array ID	0-No	6-No	12-No	0-Yes	6-Yes	12-Yes

Supplemental Table 1. Gene expression values changed greater than 2 fold.

Affymetrix ST 1.0 Array ID	0-No	6-No	12-No	0-Yes	6-Yes	12-Yes

Supplemental Table 1. Gene expression values changed greater than 2 fold.

Affymetrix ST 1.0 Array ID	0-No	6-No	12-No	0-Yes	6-Yes	12-Yes

Supplemental Table 1. Gene expression values changed greater than 2 fold.

Affymetrix ST 1.0 Array ID	0-No	6-No	12-No	0-Yes	6-Yes	12-Yes

Supplemental Table 1. Gene expression values changed greater than 2 fold.

Affymetrix ST 1.0 Array ID	0-No	6-No	12-No	0-Yes	6-Yes	12-Yes

Supplemental Table 1. Gene expression values changed greater than 2 fold.

Affymetrix ST 1.0 Array ID	0-No	6-No	12-No	0-Yes	6-Yes	12-Yes

Supplemental Table 1. Gene expression values changed greater than 2 fold.

Affymetrix ST 1.0 Array ID	0-No	6-No	12-No	0-Yes	6-Yes	12-Yes

Supplemental Table 1. Gene expression values changed greater than 2 fold.

Affymetrix ST 1.0 Array ID	0-No	6-No	12-No	0-Yes	6-Yes	12-Yes

Supplemental Table 1. Gene expression values changed greater than 2 fold.

Affymetrix ST 1.0 Array ID	0-No	6-No	12-No	0-Yes	6-Yes	12-Yes

Supplemental Table 1. Gene expression values changed greater than 2 fold.

Affymetrix ST 1.0 Array ID	0-No	6-No	12-No	0-Yes	6-Yes	12-Yes

Supplemental Table 1. Gene expression values changed greater than 2 fold.

Affymetrix ST 1.0 Array ID	0-No	6-No	12-No	0-Yes	6-Yes	12-Yes

Supplemental Table 1. Gene expression values changed greater than 2 fold.

Affymetrix ST 1.0 Array ID	0-No	6-No	12-No	0-Yes	6-Yes	12-Yes

Supplemental Table 1. Gene expression values changed greater than 2 fold.

Affymetrix ST 1.0 Array ID	0-No	6-No	12-No	0-Yes	6-Yes	12-Yes

Supplemental Table 1. Gene expression values changed greater than 2 fold.

Affymetrix ST 1.0 Array ID	0-No	6-No	12-No	0-Yes	6-Yes	12-Yes

Supplemental Table 1. Gene expression values changed greater than 2 fold.

Affymetrix ST 1.0 Array ID	0-No	6-No	12-No	0-Yes	6-Yes	12-Yes

Supplemental Table 1. Gene expression values changed greater than 2 fold.

Affymetrix ST 1.0 Array ID	0-No	6-No	12-No	0-Yes	6-Yes	12-Yes

Supplemental Table 1. Gene expression values changed greater than 2 fold.

Affymetrix ST 1.0 Array ID	0-No	6-No	12-No	0-Yes	6-Yes	12-Yes

Supplemental Table 1. Gene expression values changed greater than 2 fold.

Affymetrix ST 1.0 Array ID	0-No	6-No	12-No	0-Yes	6-Yes	12-Yes

Supplemental Table 1. Gene expression values changed greater than 2 fold.

Affymetrix ST 1.0 Array ID	0-No	6-No	12-No	0-Yes	6-Yes	12-Yes

Supplemental Table 1. Gene expression values changed greater than 2 fold.

Affymetrix ST 1.0 Array ID	0-No	6-No	12-No	0-Yes	6-Yes	12-Yes

Supplemental Table 1. Gene expression values changed greater than 2 fold.

Affymetrix ST 1.0 Array ID	0-No	6-No	12-No	0-Yes	6-Yes	12-Yes

Supplemental Table 1. Gene expression values changed greater than 2 fold.

Affymetrix ST 1.0 Array ID	0-No	6-No	12-No	0-Yes	6-Yes	12-Yes

Supplemental Table 1. Gene expression values changed greater than 2 fold.

Affymetrix ST 1.0 Array ID	0-No	6-No	12-No	0-Yes	6-Yes	12-Yes

Supplemental Table 1. Gene expression values changed greater than 2 fold.

Affymetrix ST 1.0 Array ID	0-No	6-No	12-No	0-Yes	6-Yes	12-Yes

Supplemental Table 1. Gene expression values changed greater than 2 fold.

Affymetrix ST 1.0 Array ID	0-No	6-No	12-No	0-Yes	6-Yes	12-Yes

Supplemental Table 1. Gene expression values changed greater than 2 fold.

Affymetrix ST 1.0 Array ID	0-No	6-No	12-No	0-Yes	6-Yes	12-Yes

Supplemental Table 1. Gene expression values changed greater than 2 fold.

Affymetrix ST 1.0 Array ID	0-No	6-No	12-No	0-Yes	6-Yes	12-Yes

Supplemental Table 1. Gene expression values changed greater than 2 fold.

Affymetrix ST 1.0 Array ID	0-No	6-No	12-No	0-Yes	6-Yes	12-Yes

Supplemental Table 1. Gene expression values changed greater than 2 fold.

Affymetrix ST 1.0 Array ID	0-No	6-No	12-No	0-Yes	6-Yes	12-Yes

Supplemental Table 1. Gene expression values changed greater than 2 fold.

Affymetrix ST 1.0 Array ID	0-No	6-No	12-No	0-Yes	6-Yes	12-Yes

Supplemental Table 1. Gene expression values changed greater than 2 fold.

Affymetrix ST 1.0 Array ID	0-No	6-No	12-No	0-Yes	6-Yes	12-Yes

Supplemental Table 1. Gene expression values changed greater than 2 fold.

Affymetrix ST 1.0 Array ID	0-No	6-No	12-No	0-Yes	6-Yes	12-Yes

Supplemental Table 1. Gene expression values changed greater than 2 fold.

Affymetrix ST 1.0 Array ID	0-No	6-No	12-No	0-Yes	6-Yes	12-Yes

Supplemental Table 1. Gene expression values changed greater than 2 fold.

Affymetrix ST 1.0 Array ID	0-No	6-No	12-No	0-Yes	6-Yes	12-Yes

Supplemental Table 1. Gene expression values changed greater than 2 fold.

Affymetrix ST 1.0 Array ID	0-No	6-No	12-No	0-Yes	6-Yes	12-Yes

Supplemental Table 1. Gene expression values changed greater than 2 fold.

Affymetrix ST 1.0 Array ID	0-No	6-No	12-No	0-Yes	6-Yes	12-Yes

Supplemental Table 1. Gene expression values changed greater than 2 fold.

Affymetrix ST 1.0 Array ID	0-No	6-No	12-No	0-Yes	6-Yes	12-Yes

Supplemental Table 1. Gene expression values changed greater than 2 fold.

Affymetrix ST 1.0 Array ID	0-No	6-No	12-No	0-Yes	6-Yes	12-Yes

Supplemental Table 1. Gene expression values changed greater than 2 fold.

Affymetrix ST 1.0 Array ID	0-No	6-No	12-No	0-Yes	6-Yes	12-Yes

Supplemental Table 1. Gene expression values changed greater than 2 fold.

Affymetrix ST 1.0 Array ID	0-No	6-No	12-No	0-Yes	6-Yes	12-Yes

Supplemental Table 1. Gene expression values changed greater than 2 fold.

Affymetrix ST 1.0 Array ID	0-No	6-No	12-No	0-Yes	6-Yes	12-Yes

Supplemental Table 1. Gene expression values changed greater than 2 fold.

Affymetrix ST 1.0 Array ID	0-No	6-No	12-No	0-Yes	6-Yes	12-Yes

Supplemental Table 1. Gene expression values changed greater than 2 fold.

Affymetrix ST 1.0 Array ID	0-No	6-No	12-No	0-Yes	6-Yes	12-Yes

Supplemental Table 1. Gene expression values changed greater than 2 fold.

Affymetrix ST 1.0 Array ID	0-No	6-No	12-No	0-Yes	6-Yes	12-Yes

Supplemental Table 1. Gene expression values changed greater than 2 fold.

Affymetrix ST 1.0 Array ID	0-No	6-No	12-No	0-Yes	6-Yes	12-Yes

Supplemental Table 1. Gene expression values changed greater than 2 fold.

Affymetrix ST 1.0 Array ID	0-No	6-No	12-No	0-Yes	6-Yes	12-Yes

Supplemental Table 1. Gene expression values changed greater than 2 fold.

Affymetrix ST 1.0 Array ID	0-No	6-No	12-No	0-Yes	6-Yes	12-Yes

Supplemental Table 1. Gene expression values changed greater than 2 fold.

Affymetrix ST 1.0 Array ID	0-No	6-No	12-No	0-Yes	6-Yes	12-Yes

Supplemental Table 1. Gene expression values changed greater than 2 fold.

Affymetrix ST 1.0 Array ID	0-No	6-No	12-No	0-Yes	6-Yes	12-Yes

Supplemental Table 1. Gene expression values changed greater than 2 fold.

Affymetrix ST 1.0 Array ID	0-No	6-No	12-No	0-Yes	6-Yes	12-Yes

Supplemental Table 1. Gene expression values changed greater than 2 fold.

Affymetrix ST 1.0 Array ID	0-No	6-No	12-No	0-Yes	6-Yes	12-Yes

Supplemental Table 1. Gene expression values changed greater than 2 fold.

Affymetrix ST 1.0 Array ID	0-No	6-No	12-No	0-Yes	6-Yes	12-Yes

Supplemental Table 1. Gene expression values changed greater than 2 fold.

Affymetrix ST 1.0 Array ID	0-No	6-No	12-No	0-Yes	6-Yes	12-Yes

Supplemental Table 1. Gene expression values changed greater than 2 fold.

Affymetrix ST 1.0 Array ID	0-No	6-No	12-No	0-Yes	6-Yes	12-Yes

Supplemental Table 1. Gene expression values changed greater than 2 fold.

Affymetrix ST 1.0 Array ID	0-No	6-No	12-No	0-Yes	6-Yes	12-Yes

Supplemental Table 1. Gene expression values changed greater than 2 fold.

Affymetrix ST 1.0 Array ID	0-No	6-No	12-No	0-Yes	6-Yes	12-Yes

Supplemental Table 1. Gene expression values changed greater than 2 fold.

Affymetrix ST 1.0 Array ID	0-No	6-No	12-No	0-Yes	6-Yes	12-Yes

Supplemental Table 1. Gene expression values changed greater than 2 fold.

Affymetrix ST 1.0 Array ID	0-No	6-No	12-No	0-Yes	6-Yes	12-Yes

Supplemental Table 1. Gene expression values changed greater than 2 fold.

Affymetrix ST 1.0 Array ID	0-No	6-No	12-No	0-Yes	6-Yes	12-Yes

Supplemental Table 1. Gene expression values changed greater than 2 fold.

Affymetrix ST 1.0 Array ID	0-No	6-No	12-No	0-Yes	6-Yes	12-Yes

Supplemental Table 1. Gene expression values changed greater than 2 fold.

Affymetrix ST 1.0 Array ID	0-No	6-No	12-No	0-Yes	6-Yes	12-Yes

Supplemental Table 1. Gene expression values changed greater than 2 fold.

Affymetrix ST 1.0 Array ID	0-No	6-No	12-No	0-Yes	6-Yes	12-Yes

Supplemental Table 1. Gene expression values changed greater than 2 fold.

Affymetrix ST 1.0 Array ID	0-No	6-No	12-No	0-Yes	6-Yes	12-Yes

Supplemental Table 1. Gene expression values changed greater than 2 fold.

Affymetrix ST 1.0 Array ID	0-No	6-No	12-No	0-Yes	6-Yes	12-Yes

Supplemental Table 1. Gene expression values changed greater than 2 fold.

Affymetrix ST 1.0 Array ID	0-No	6-No	12-No	0-Yes	6-Yes	12-Yes

Supplemental Table 1. Gene expression values changed greater than 2 fold.

Affymetrix ST 1.0 Array ID	0-No	6-No	12-No	0-Yes	6-Yes	12-Yes

Supplemental Table 1. Gene expression values changed greater than 2 fold.

Affymetrix ST 1.0 Array ID	0-No	6-No	12-No	0-Yes	6-Yes	12-Yes

Supplemental Table 1. Gene expression values changed greater than 2 fold.

Affymetrix ST 1.0 Array ID	0-No	6-No	12-No	0-Yes	6-Yes	12-Yes

Supplemental Table 1. Gene expression values changed greater than 2 fold.

Affymetrix ST 1.0 Array ID	0-No	6-No	12-No	0-Yes	6-Yes	12-Yes

Supplemental Table 1. Gene expression values changed greater than 2 fold.

Affymetrix ST 1.0 Array ID	0-No	6-No	12-No	0-Yes	6-Yes	12-Yes

Supplemental Table 1. Gene expression values changed greater than 2 fold.

Affymetrix ST 1.0 Array ID	0-No	6-No	12-No	0-Yes	6-Yes	12-Yes

Supplemental Table 1. Gene expression values changed greater than 2 fold.

Affymetrix ST 1.0 Array ID	0-No	6-No	12-No	0-Yes	6-Yes	12-Yes

Supplemental Table 1. Gene expression values changed greater than 2 fold.

Affymetrix ST 1.0 Array ID	0-No	6-No	12-No	0-Yes	6-Yes	12-Yes

Supplemental Table 1. Gene expression values changed greater than 2 fold.

Affymetrix ST 1.0 Array ID	0-No	6-No	12-No	0-Yes	6-Yes	12-Yes

Supplemental Table 1. Gene expression values changed greater than 2 fold.

Affymetrix ST 1.0 Array ID	0-No	6-No	12-No	0-Yes	6-Yes	12-Yes

Supplemental Table 1. Gene expression values changed greater than 2 fold.

Affymetrix ST 1.0 Array ID	0-No	6-No	12-No	0-Yes	6-Yes	12-Yes

Supplemental Table 1. Gene expression values changed greater than 2 fold.

Affymetrix ST 1.0 Array ID	0-No	6-No	12-No	0-Yes	6-Yes	12-Yes

Supplemental Table 1. Gene expression values changed greater than 2 fold.

Affymetrix ST 1.0 Array ID	0-No	6-No	12-No	0-Yes	6-Yes	12-Yes

Supplemental Table 1. Gene expression values changed greater than 2 fold.

Affymetrix ST 1.0 Array ID	0-No	6-No	12-No	0-Yes	6-Yes	12-Yes

Supplemental Table 1. Gene expression values changed greater than 2 fold.

Affymetrix ST 1.0 Array ID	0-No	6-No	12-No	0-Yes	6-Yes	12-Yes

Supplemental Table 1. Gene expression values changed greater than 2 fold.

Affymetrix ST 1.0 Array ID	0-No	6-No	12-No	0-Yes	6-Yes	12-Yes

Supplemental Table 1. Gene expression values changed greater than 2 fold.

Affymetrix ST 1.0 Array ID	0-No	6-No	12-No	0-Yes	6-Yes	12-Yes

Supplemental Table 1. Gene expression values changed greater than 2 fold.

Affymetrix ST 1.0 Array ID	0-No	6-No	12-No	0-Yes	6-Yes	12-Yes

Supplemental Table 1. Gene expression values changed greater than 2 fold.

Affymetrix ST 1.0 Array ID	0-No	6-No	12-No	0-Yes	6-Yes	12-Yes

Supplemental Table 1. Gene expression values changed greater than 2 fold.

Affymetrix ST 1.0 Array ID	0-No	6-No	12-No	0-Yes	6-Yes	12-Yes

Supplemental Table 1. Gene expression values changed greater than 2 fold.

Affymetrix ST 1.0 Array ID	0-No	6-No	12-No	0-Yes	6-Yes	12-Yes

Supplemental Table 1. Gene expression values changed greater than 2 fold.

Affymetrix ST 1.0 Array ID	0-No	6-No	12-No	0-Yes	6-Yes	12-Yes

Supplemental Table 1. Gene expression values changed greater than 2 fold.

Affymetrix ST 1.0 Array ID	0-No	6-No	12-No	0-Yes	6-Yes	12-Yes

Supplemental Table 1. Gene expression values changed greater than 2 fold.

Affymetrix ST 1.0 Array ID	0-No	6-No	12-No	0-Yes	6-Yes	12-Yes

Supplemental Table 1. Gene expression values changed greater than 2 fold.

Affymetrix ST 1.0 Array ID	0-No	6-No	12-No	0-Yes	6-Yes	12-Yes

Supplemental Table 1. Gene expression values changed greater than 2 fold.

Affymetrix ST 1.0 Array ID	0-No	6-No	12-No	0-Yes	6-Yes	12-Yes

Supplemental Table 1. Gene expression values changed greater than 2 fold.

Affymetrix ST 1.0 Array ID	0-No	6-No	12-No	0-Yes	6-Yes	12-Yes

Supplemental Table 1. Gene expression values changed greater than 2 fold.

Affymetrix ST 1.0 Array ID	0-No	6-No	12-No	0-Yes	6-Yes	12-Yes

Supplemental Table 1. Gene expression values changed greater than 2 fold.

Affymetrix ST 1.0 Array ID	0-No	6-No	12-No	0-Yes	6-Yes	12-Yes

Supplemental Table 1. Gene expression values changed greater than 2 fold.

Affymetrix ST 1.0 Array ID	0-No	6-No	12-No	0-Yes	6-Yes	12-Yes

Supplemental Table 1. Gene expression values changed greater than 2 fold.

Affymetrix ST 1.0 Array ID	0-No	6-No	12-No	0-Yes	6-Yes	12-Yes

Supplemental Table 1. Gene expression values changed greater than 2 fold.

Affymetrix ST 1.0 Array ID	0-No	6-No	12-No	0-Yes	6-Yes	12-Yes

Supplemental Table 1. Gene expression values changed greater than 2 fold.

Affymetrix ST 1.0 Array ID	0-No	6-No	12-No	0-Yes	6-Yes	12-Yes

Supplemental Table 1. Gene expression values changed greater than 2 fold.

Affymetrix ST 1.0 Array ID	0-No	6-No	12-No	0-Yes	6-Yes	12-Yes

Supplemental Table 1. Gene expression values changed greater than 2 fold.

Affymetrix ST 1.0 Array ID	0-No	6-No	12-No	0-Yes	6-Yes	12-Yes

Supplemental Table 1. Gene expression values changed greater than 2 fold.

Affymetrix ST 1.0 Array ID	0-No	6-No	12-No	0-Yes	6-Yes	12-Yes

Supplemental Table 1. Gene expression values changed greater than 2 fold.

Affymetrix ST 1.0 Array ID	0-No	6-No	12-No	0-Yes	6-Yes	12-Yes

Supplemental Table 1. Gene expression values changed greater than 2 fold.

Affymetrix ST 1.0 Array ID	0-No	6-No	12-No	0-Yes	6-Yes	12-Yes

Supplemental Table 1. Gene expression values changed greater than 2 fold.

Affymetrix ST 1.0 Array ID	0-No	6-No	12-No	0-Yes	6-Yes	12-Yes

Supplemental Table 1. Gene expression values changed greater than 2 fold.

Affymetrix ST 1.0 Array ID	0-No	6-No	12-No	0-Yes	6-Yes	12-Yes

Supplemental Table 1. Gene expression values changed greater than 2 fold.

Affymetrix ST 1.0 Array ID	0-No	6-No	12-No	0-Yes	6-Yes	12-Yes

Supplemental Table 1. Gene expression values changed greater than 2 fold.

Affymetrix ST 1.0 Array ID	0-No	6-No	12-No	0-Yes	6-Yes	12-Yes

Supplemental Table 1. Gene expression values changed greater than 2 fold.

Affymetrix ST 1.0 Array ID	0-No	6-No	12-No	0-Yes	6-Yes	12-Yes

Supplemental Table 1. Gene expression values changed greater than 2 fold.

Affymetrix ST 1.0 Array ID	0-No	6-No	12-No	0-Yes	6-Yes	12-Yes

Supplemental Table 1. Gene expression values changed greater than 2 fold.

Affymetrix ST 1.0 Array ID	0-No	6-No	12-No	0-Yes	6-Yes	12-Yes

Supplemental Table 1. Gene expression values changed greater than 2 fold.

Affymetrix ST 1.0 Array ID	0-No	6-No	12-No	0-Yes	6-Yes	12-Yes

Supplemental Table 1. Gene expression values changed greater than 2 fold.

Affymetrix ST 1.0 Array ID	0-No	6-No	12-No	0-Yes	6-Yes	12-Yes

Supplemental Table 1. Gene expression values changed greater than 2 fold.

Affymetrix ST 1.0 Array ID	0-No	6-No	12-No	0-Yes	6-Yes	12-Yes

Supplemental Table 1. Gene expression values changed greater than 2 fold.

Affymetrix ST 1.0 Array ID	0-No	6-No	12-No	0-Yes	6-Yes	12-Yes

Supplemental Table 1. Gene expression values changed greater than 2 fold.

Affymetrix ST 1.0 Array ID	0-No	6-No	12-No	0-Yes	6-Yes	12-Yes

Supplemental Table 1. Gene expression values changed greater than 2 fold.

Affymetrix ST 1.0 Array ID	0-No	6-No	12-No	0-Yes	6-Yes	12-Yes

Supplemental Table 1. Gene expression values changed greater than 2 fold.

Affymetrix ST 1.0 Array ID	0-No	6-No	12-No	0-Yes	6-Yes	12-Yes

Supplemental Table 1. Gene expression values changed greater than 2 fold.

Affymetrix ST 1.0 Array ID	0-No	6-No	12-No	0-Yes	6-Yes	12-Yes

Supplemental Table 1. Gene expression values changed greater than 2 fold.

Affymetrix ST 1.0 Array ID	0-No	6-No	12-No	0-Yes	6-Yes	12-Yes

Supplemental Table 1. Gene expression values changed greater than 2 fold.

Affymetrix ST 1.0 Array ID	0-No	6-No	12-No	0-Yes	6-Yes	12-Yes

Supplemental Table 1. Gene expression values changed greater than 2 fold.

Affymetrix ST 1.0 Array ID	0-No	6-No	12-No	0-Yes	6-Yes	12-Yes

Supplemental Table 1. Gene expression values changed greater than 2 fold.

Affymetrix ST 1.0 Array ID	0-No	6-No	12-No	0-Yes	6-Yes	12-Yes

Supplemental Table 1. Gene expression values changed greater than 2 fold.

Affymetrix ST 1.0 Array ID	0-No	6-No	12-No	0-Yes	6-Yes	12-Yes

Supplemental Table 1. Gene expression values changed greater than 2 fold.

Affymetrix ST 1.0 Array ID	0-No	6-No	12-No	0-Yes	6-Yes	12-Yes

Supplemental Table 1. Gene expression values changed greater than 2 fold.

Affymetrix ST 1.0 Array ID	0-No	6-No	12-No	0-Yes	6-Yes	12-Yes

Supplemental Table 1. Gene expression values changed greater than 2 fold.

Affymetrix ST 1.0 Array ID	0-No	6-No	12-No	0-Yes	6-Yes	12-Yes

Supplemental Table 1. Gene expression values changed greater than 2 fold.

Affymetrix ST 1.0 Array ID	0-No	6-No	12-No	0-Yes	6-Yes	12-Yes

Supplemental Table 1. Gene expression values changed greater than 2 fold.

Affymetrix ST 1.0 Array ID	0-No	6-No	12-No	0-Yes	6-Yes	12-Yes

Supplemental Table 1. Gene expression values changed greater than 2 fold.

Affymetrix ST 1.0 Array ID	0-No	6-No	12-No	0-Yes	6-Yes	12-Yes

Supplemental Table 1. Gene expression values changed greater than 2 fold.

Affymetrix ST 1.0 Array ID	0-No	6-No	12-No	0-Yes	6-Yes	12-Yes

Supplemental Table 1. Gene expression values changed greater than 2 fold.

Affymetrix ST 1.0 Array ID	0-No	6-No	12-No	0-Yes	6-Yes	12-Yes

Supplemental Table 1. Gene expression values changed greater than 2 fold.

Affymetrix ST 1.0 Array ID	0-No	6-No	12-No	0-Yes	6-Yes	12-Yes

Supplemental Table 1. Gene expression values changed greater than 2 fold.

Affymetrix ST 1.0 Array ID	0-No	6-No	12-No	0-Yes	6-Yes	12-Yes

Supplemental Table 1. Gene expression values changed greater than 2 fold.

Affymetrix ST 1.0 Array ID	0-No	6-No	12-No	0-Yes	6-Yes	12-Yes

Supplemental Table 1. Gene expression values changed greater than 2 fold.

Affymetrix ST 1.0 Array ID	0-No	6-No	12-No	0-Yes	6-Yes	12-Yes

Supplemental Table 1. Gene expression values changed greater than 2 fold.

Affymetrix ST 1.0 Array ID	0-No	6-No	12-No	0-Yes	6-Yes	12-Yes

Supplemental Table 1. Gene expression values changed greater than 2 fold.

Affymetrix ST 1.0 Array ID	0-No	6-No	12-No	0-Yes	6-Yes	12-Yes

Supplemental Table 1. Gene expression values changed greater than 2 fold.

Affymetrix ST 1.0 Array ID	0-No	6-No	12-No	0-Yes	6-Yes	12-Yes

Supplemental Table 1. Gene expression values changed greater than 2 fold.

Affymetrix ST 1.0 Array ID	0-No	6-No	12-No	0-Yes	6-Yes	12-Yes

Supplemental Table 1. Gene expression values changed greater than 2 fold.

Affymetrix ST 1.0 Array ID	0-No	6-No	12-No	0-Yes	6-Yes	12-Yes

Supplemental Table 1. Gene expression values changed greater than 2 fold.

Affymetrix ST 1.0 Array ID	0-No	6-No	12-No	0-Yes	6-Yes	12-Yes

Supplemental Table 1. Gene expression values changed greater than 2 fold.

Affymetrix ST 1.0 Array ID	0-No	6-No	12-No	0-Yes	6-Yes	12-Yes

Supplemental Table 1. Gene expression values changed greater than 2 fold.

Affymetrix ST 1.0 Array ID	0-No	6-No	12-No	0-Yes	6-Yes	12-Yes

Supplemental Table 1. Gene expression values changed greater than 2 fold.

Affymetrix ST 1.0 Array ID	0-No	6-No	12-No	0-Yes	6-Yes	12-Yes

Supplemental Table 1. Gene expression values changed greater than 2 fold.

Affymetrix ST 1.0 Array ID	0-No	6-No	12-No	0-Yes	6-Yes	12-Yes

Supplemental Table 1. Gene expression values changed greater than 2 fold.

Affymetrix ST 1.0 Array ID	0-No	6-No	12-No	0-Yes	6-Yes	12-Yes

Supplemental Table 1. Gene expression values changed greater than 2 fold.

Affymetrix ST 1.0 Array ID	0-No	6-No	12-No	0-Yes	6-Yes	12-Yes

Supplemental Table 1. Gene expression values changed greater than 2 fold.

Affymetrix ST 1.0 Array ID	0-No	6-No	12-No	0-Yes	6-Yes	12-Yes

Supplemental Table 1. Gene expression values changed greater than 2 fold.

Affymetrix ST 1.0 Array ID	0-No	6-No	12-No	0-Yes	6-Yes	12-Yes

Supplemental Table 1. Gene expression values changed greater than 2 fold.

Affymetrix ST 1.0 Array ID	0-No	6-No	12-No	0-Yes	6-Yes	12-Yes

Supplemental Table 1. Gene expression values changed greater than 2 fold.

Affymetrix ST 1.0 Array ID	0-No	6-No	12-No	0-Yes	6-Yes	12-Yes

Supplemental Table 1. Gene expression values changed greater than 2 fold.

Affymetrix ST 1.0 Array ID	0-No	6-No	12-No	0-Yes	6-Yes	12-Yes

Supplemental Table 1. Gene expression values changed greater than 2 fold.

Affymetrix ST 1.0 Array ID	0-No	6-No	12-No	0-Yes	6-Yes	12-Yes

Supplemental Table 1. Gene expression values changed greater than 2 fold.

Affymetrix ST 1.0 Array ID	0-No	6-No	12-No	0-Yes	6-Yes	12-Yes

Supplemental Table 1. Gene expression values changed greater than 2 fold.

Affymetrix ST 1.0 Array ID	0-No	6-No	12-No	0-Yes	6-Yes	12-Yes

Supplemental Table 1. Gene expression values changed greater than 2 fold.

Affymetrix ST 1.0 Array ID	0-No	6-No	12-No	0-Yes	6-Yes	12-Yes

Supplemental Table 1. Gene expression values changed greater than 2 fold.

Affymetrix ST 1.0 Array ID	0-No	6-No	12-No	0-Yes	6-Yes	12-Yes

Supplemental Table 1. Gene expression values changed greater than 2 fold.

Affymetrix ST 1.0 Array ID	0-No	6-No	12-No	0-Yes	6-Yes	12-Yes

Supplemental Table 1. Gene expression values changed greater than 2 fold.

Affymetrix ST 1.0 Array ID	0-No	6-No	12-No	0-Yes	6-Yes	12-Yes

Supplemental Table 1. Gene expression values changed greater than 2 fold.

Affymetrix ST 1.0 Array ID	0-No	6-No	12-No	0-Yes	6-Yes	12-Yes

Supplemental Table 1. Gene expression values changed greater than 2 fold.

Affymetrix ST 1.0 Array ID	0-No	6-No	12-No	0-Yes	6-Yes	12-Yes

Supplemental Table 1. Gene expression values changed greater than 2 fold.

Affymetrix ST 1.0 Array ID	0-No	6-No	12-No	0-Yes	6-Yes	12-Yes

Supplemental Table 1. Gene expression values changed greater than 2 fold.

Affymetrix ST 1.0 Array ID	0-No	6-No	12-No	0-Yes	6-Yes	12-Yes

Supplemental Table 1. Gene expression values changed greater than 2 fold.

Affymetrix ST 1.0 Array ID	0-No	6-No	12-No	0-Yes	6-Yes	12-Yes

Supplemental Table 1. Gene expression values changed greater than 2 fold.

Affymetrix ST 1.0 Array ID	0-No	6-No	12-No	0-Yes	6-Yes	12-Yes

Supplemental Table 1. Gene expression values changed greater than 2 fold.

Affymetrix ST 1.0 Array ID	0-No	6-No	12-No	0-Yes	6-Yes	12-Yes

Supplemental Table 1. Gene expression values changed greater than 2 fold.

Affymetrix ST 1.0 Array ID	0-No	6-No	12-No	0-Yes	6-Yes	12-Yes

Supplemental Table 1. Gene expression values changed greater than 2 fold.

Affymetrix ST 1.0 Array ID	0-No	6-No	12-No	0-Yes	6-Yes	12-Yes

Supplemental Table 1. Gene expression values changed greater than 2 fold.

Affymetrix ST 1.0 Array ID	0-No	6-No	12-No	0-Yes	6-Yes	12-Yes

Supplemental Table 1. Gene expression values changed greater than 2 fold.

Affymetrix ST 1.0 Array ID	0-No	6-No	12-No	0-Yes	6-Yes	12-Yes

Supplemental Table 1. Gene expression values changed greater than 2 fold.

Affymetrix ST 1.0 Array ID	0-No	6-No	12-No	0-Yes	6-Yes	12-Yes

Supplemental Table 1. Gene expression values changed greater than 2 fold.

Affymetrix ST 1.0 Array ID	0-No	6-No	12-No	0-Yes	6-Yes	12-Yes

Supplemental Table 1. Gene expression values changed greater than 2 fold.

Affymetrix ST 1.0 Array ID	0-No	6-No	12-No	0-Yes	6-Yes	12-Yes

Supplemental Table 1. Gene expression values changed greater than 2 fold.

Affymetrix ST 1.0 Array ID	0-No	6-No	12-No	0-Yes	6-Yes	12-Yes

Supplemental Table 1. Gene expression values changed greater than 2 fold.

Affymetrix ST 1.0 Array ID	0-No	6-No	12-No	0-Yes	6-Yes	12-Yes

Supplemental Table 1. Gene expression values changed greater than 2 fold.

Affymetrix ST 1.0 Array ID	0-No	6-No	12-No	0-Yes	6-Yes	12-Yes

Supplemental Table 1. Gene expression values changed greater than 2 fold.

Affymetrix ST 1.0 Array ID	0-No	6-No	12-No	0-Yes	6-Yes	12-Yes

Supplemental Table 1. Gene expression values changed greater than 2 fold.

Affymetrix ST 1.0 Array ID	0-No	6-No	12-No	0-Yes	6-Yes	12-Yes

Supplemental Table 1. Gene expression values changed greater than 2 fold.

Affymetrix ST 1.0 Array ID	0-No	6-No	12-No	0-Yes	6-Yes	12-Yes

Supplemental Table 1. Gene expression values changed greater than 2 fold.

Affymetrix ST 1.0 Array ID	0-No	6-No	12-No	0-Yes	6-Yes	12-Yes

Supplemental Table 1. Gene expression values changed greater than 2 fold.

Affymetrix ST 1.0 Array ID	0-No	6-No	12-No	0-Yes	6-Yes	12-Yes

Supplemental Table 1. Gene expression values changed greater than 2 fold.

Affymetrix ST 1.0 Array ID	0-No	6-No	12-No	0-Yes	6-Yes	12-Yes

Supplemental Table 1. Gene expression values changed greater than 2 fold.

Affymetrix ST 1.0 Array ID	0-No	6-No	12-No	0-Yes	6-Yes	12-Yes

Supplemental Table 1. Gene expression values changed greater than 2 fold.

Affymetrix ST 1.0 Array ID	0-No	6-No	12-No	0-Yes	6-Yes	12-Yes

Supplemental Table 1. Gene expression values changed greater than 2 fold.

Affymetrix ST 1.0 Array ID	0-No	6-No	12-No	0-Yes	6-Yes	12-Yes

Supplemental Table 1. Gene expression values changed greater than 2 fold.

Affymetrix ST 1.0 Array ID	0-No	6-No	12-No	0-Yes	6-Yes	12-Yes

Supplemental Table 1. Gene expression values changed greater than 2 fold.

Affymetrix ST 1.0 Array ID	0-No	6-No	12-No	0-Yes	6-Yes	12-Yes

Supplemental Table 1. Gene expression values changed greater than 2 fold.

Affymetrix ST 1.0 Array ID	0-No	6-No	12-No	0-Yes	6-Yes	12-Yes

Supplemental Table 1. Gene expression values changed greater than 2 fold.

Affymetrix ST 1.0 Array ID	0-No	6-No	12-No	0-Yes	6-Yes	12-Yes

Supplemental Table 1. Gene expression values changed greater than 2 fold.

Affymetrix ST 1.0 Array ID	0-No	6-No	12-No	0-Yes	6-Yes	12-Yes

Supplemental Table 1. Gene expression values changed greater than 2 fold.

Affymetrix ST 1.0 Array ID	0-No	6-No	12-No	0-Yes	6-Yes	12-Yes

Supplemental Table 1. Gene expression values changed greater than 2 fold.

Affymetrix ST 1.0 Array ID	0-No	6-No	12-No	0-Yes	6-Yes	12-Yes

Supplemental Table 1. Gene expression values changed greater than 2 fold.

Affymetrix ST 1.0 Array ID	0-No	6-No	12-No	0-Yes	6-Yes	12-Yes

Supplemental Table 1. Gene expression values changed greater than 2 fold.

Affymetrix ST 1.0 Array ID	0-No	6-No	12-No	0-Yes	6-Yes	12-Yes

Supplemental Table 1. Gene expression values changed greater than 2 fold.

Affymetrix ST 1.0 Array ID	0-No	6-No	12-No	0-Yes	6-Yes	12-Yes

Supplemental Table 1. Gene expression values changed greater than 2 fold.

Affymetrix ST 1.0 Array ID	0-No	6-No	12-No	0-Yes	6-Yes	12-Yes

Supplemental Table 1. Gene expression values changed greater than 2 fold.

Affymetrix ST 1.0 Array ID	0-No	6-No	12-No	0-Yes	6-Yes	12-Yes

Supplemental Table 1. Gene expression values changed greater than 2 fold.

Affymetrix ST 1.0 Array ID	0-No	6-No	12-No	0-Yes	6-Yes	12-Yes

Supplemental Table 1. Gene expression values changed greater than 2 fold.

Affymetrix ST 1.0 Array ID	0-No	6-No	12-No	0-Yes	6-Yes	12-Yes

Supplemental Table 1. Gene expression values changed greater than 2 fold.

Affymetrix ST 1.0 Array ID	0-No	6-No	12-No	0-Yes	6-Yes	12-Yes

Supplemental Table 1. Gene expression values changed greater than 2 fold.

Affymetrix ST 1.0 Array ID	0-No	6-No	12-No	0-Yes	6-Yes	12-Yes

Supplemental Table 1. Gene expression values changed greater than 2 fold.

Affymetrix ST 1.0 Array ID	0-No	6-No	12-No	0-Yes	6-Yes	12-Yes

Supplemental Table 1. Gene expression values changed greater than 2 fold.

Affymetrix ST 1.0 Array ID	0-No	6-No	12-No	0-Yes	6-Yes	12-Yes

Supplemental Table 1. Gene expression values changed greater than 2 fold.

Affymetrix ST 1.0 Array ID	0-No	6-No	12-No	0-Yes	6-Yes	12-Yes

Supplemental Table 1. Gene expression values changed greater than 2 fold.

Affymetrix ST 1.0 Array ID	0-No	6-No	12-No	0-Yes	6-Yes	12-Yes

Supplemental Table 1. Gene expression values changed greater than 2 fold.

Affymetrix ST 1.0 Array ID	0-No	6-No	12-No	0-Yes	6-Yes	12-Yes

Supplemental Table 1. Gene expression values changed greater than 2 fold.

Affymetrix ST 1.0 Array ID	0-No	6-No	12-No	0-Yes	6-Yes	12-Yes

Supplemental Table 1. Gene expression values changed greater than 2 fold.

Affymetrix ST 1.0 Array ID	0-No	6-No	12-No	0-Yes	6-Yes	12-Yes

Supplemental Table 1. Gene expression values changed greater than 2 fold.

Affymetrix ST 1.0 Array ID	0-No	6-No	12-No	0-Yes	6-Yes	12-Yes

Supplemental Table 1. Gene expression values changed greater than 2 fold.

Affymetrix ST 1.0 Array ID	0-No	6-No	12-No	0-Yes	6-Yes	12-Yes

Supplemental Table 1. Gene expression values changed greater than 2 fold.

Affymetrix ST 1.0 Array ID	0-No	6-No	12-No	0-Yes	6-Yes	12-Yes

Supplemental Table 1. Gene expression values changed greater than 2 fold.

Affymetrix ST 1.0 Array ID	0-No	6-No	12-No	0-Yes	6-Yes	12-Yes

Supplemental Table 1. Gene expression values changed greater than 2 fold.

Affymetrix ST 1.0 Array ID	0-No	6-No	12-No	0-Yes	6-Yes	12-Yes

Supplemental Table 1. Gene expression values changed greater than 2 fold.

Affymetrix ST 1.0 Array ID	0-No	6-No	12-No	0-Yes	6-Yes	12-Yes

Supplemental Table 1. Gene expression values changed greater than 2 fold.

Affymetrix ST 1.0 Array ID	0-No	6-No	12-No	0-Yes	6-Yes	12-Yes

Supplemental Table 1. Gene expression values changed greater than 2 fold.

Affymetrix ST 1.0 Array ID	0-No	6-No	12-No	0-Yes	6-Yes	12-Yes

Supplemental Table 1. Gene expression values changed greater than 2 fold.

Affymetrix ST 1.0 Array ID	0-No	6-No	12-No	0-Yes	6-Yes	12-Yes

Supplemental Table 1. Gene expression values changed greater than 2 fold.

Affymetrix ST 1.0 Array ID	0-No	6-No	12-No	0-Yes	6-Yes	12-Yes

Supplemental Table 1. Gene expression values changed greater than 2 fold.

Affymetrix ST 1.0 Array ID	0-No	6-No	12-No	0-Yes	6-Yes	12-Yes

Supplemental Table 1. Gene expression values changed greater than 2 fold.

Affymetrix ST 1.0 Array ID	0-No	6-No	12-No	0-Yes	6-Yes	12-Yes

Supplemental Table 1. Gene expression values changed greater than 2 fold.

Affymetrix ST 1.0 Array ID	0-No	6-No	12-No	0-Yes	6-Yes	12-Yes

Supplemental Table 1. Gene expression values changed greater than 2 fold.

Affymetrix ST 1.0 Array ID	0-No	6-No	12-No	0-Yes	6-Yes	12-Yes

Supplemental Table 1. Gene expression values changed greater than 2 fold.

Affymetrix ST 1.0 Array ID	0-No	6-No	12-No	0-Yes	6-Yes	12-Yes

Supplemental Table 1. Gene expression values changed greater than 2 fold.

Affymetrix ST 1.0 Array ID	0-No	6-No	12-No	0-Yes	6-Yes	12-Yes

Supplemental Table 1. Gene expression values changed greater than 2 fold.

Affymetrix ST 1.0 Array ID	0-No	6-No	12-No	0-Yes	6-Yes	12-Yes

Supplemental Table 1. Gene expression values changed greater than 2 fold.

Affymetrix ST 1.0 Array ID	0-No	6-No	12-No	0-Yes	6-Yes	12-Yes

Supplemental Table 1. Gene expression values changed greater than 2 fold.

Affymetrix ST 1.0 Array ID	0-No	6-No	12-No	0-Yes	6-Yes	12-Yes

Supplemental Table 1. Gene expression values changed greater than 2 fold.

Affymetrix ST 1.0 Array ID	0-No	6-No	12-No	0-Yes	6-Yes	12-Yes

Supplemental Table 1. Gene expression values changed greater than 2 fold.

Affymetrix ST 1.0 Array ID	0-No	6-No	12-No	0-Yes	6-Yes	12-Yes

Supplemental Table 1. Gene expression values changed greater than 2 fold.

Affymetrix ST 1.0 Array ID	0-No	6-No	12-No	0-Yes	6-Yes	12-Yes

Supplemental Table 1. Gene expression values changed greater than 2 fold.

Affymetrix ST 1.0 Array ID	0-No	6-No	12-No	0-Yes	6-Yes	12-Yes

Supplemental Table 1. Gene expression values changed greater than 2 fold.

Affymetrix ST 1.0 Array ID	0-No	6-No	12-No	0-Yes	6-Yes	12-Yes

Supplemental Table 1. Gene expression values changed greater than 2 fold.

Affymetrix ST 1.0 Array ID	0-No	6-No	12-No	0-Yes	6-Yes	12-Yes

Supplemental Table 1. Gene expression values changed greater than 2 fold.

Affymetrix ST 1.0 Array ID	0-No	6-No	12-No	0-Yes	6-Yes	12-Yes

Supplemental Table 1. Gene expression values changed greater than 2 fold.

Affymetrix ST 1.0 Array ID	0-No	6-No	12-No	0-Yes	6-Yes	12-Yes

Supplemental Table 1. Gene expression values changed greater than 2 fold.

Affymetrix ST 1.0 Array ID	0-No	6-No	12-No	0-Yes	6-Yes	12-Yes

Supplemental Table 1. Gene expression values changed greater than 2 fold.

Affymetrix ST 1.0 Array ID	0-No	6-No	12-No	0-Yes	6-Yes	12-Yes

Supplemental Table 1. Gene expression values changed greater than 2 fold.

Affymetrix ST 1.0 Array ID	0-No	6-No	12-No	0-Yes	6-Yes	12-Yes

Supplemental Table 1. Gene expression values changed greater than 2 fold.

Affymetrix ST 1.0 Array ID	0-No	6-No	12-No	0-Yes	6-Yes	12-Yes

Supplemental Table 1. Gene expression values changed greater than 2 fold.

Affymetrix ST 1.0 Array ID	0-No	6-No	12-No	0-Yes	6-Yes	12-Yes

Supplemental Table 1. Gene expression values changed greater than 2 fold.

Affymetrix ST 1.0 Array ID	0-No	6-No	12-No	0-Yes	6-Yes	12-Yes

Supplemental Table 1. Gene expression values changed greater than 2 fold.

Affymetrix ST 1.0 Array ID	0-No	6-No	12-No	0-Yes	6-Yes	12-Yes

Supplemental Table 1. Gene expression values changed greater than 2 fold.

Affymetrix ST 1.0 Array ID	0-No	6-No	12-No	0-Yes	6-Yes	12-Yes

Supplemental Table 1. Gene expression values changed greater than 2 fold.

Affymetrix ST 1.0 Array ID	0-No	6-No	12-No	0-Yes	6-Yes	12-Yes

Supplemental Table 1. Gene expression values changed greater than 2 fold.

Affymetrix ST 1.0 Array ID	0-No	6-No	12-No	0-Yes	6-Yes	12-Yes

Supplemental Table 1. Gene expression values changed greater than 2 fold.

Affymetrix ST 1.0 Array ID	0-No	6-No	12-No	0-Yes	6-Yes	12-Yes

Supplemental Table 1. Gene expression values changed greater than 2 fold.

Affymetrix ST 1.0 Array ID	0-No	6-No	12-No	0-Yes	6-Yes	12-Yes

Supplemental Table 1. Gene expression values changed greater than 2 fold.

Affymetrix ST 1.0 Array ID	0-No	6-No	12-No	0-Yes	6-Yes	12-Yes

Supplemental Table 1. Gene expression values changed greater than 2 fold.

Affymetrix ST 1.0 Array ID	0-No	6-No	12-No	0-Yes	6-Yes	12-Yes

Supplemental Table 1. Gene expression values changed greater than 2 fold.

Affymetrix ST 1.0 Array ID	0-No	6-No	12-No	0-Yes	6-Yes	12-Yes

Supplemental Table 1. Gene expression values changed greater than 2 fold.

Affymetrix ST 1.0 Array ID	0-No	6-No	12-No	0-Yes	6-Yes	12-Yes

Supplemental Table 1. Gene expression values changed greater than 2 fold.

Affymetrix ST 1.0 Array ID	0-No	6-No	12-No	0-Yes	6-Yes	12-Yes

Supplemental Table 1. Gene expression values changed greater than 2 fold.

Affymetrix ST 1.0 Array ID	0-No	6-No	12-No	0-Yes	6-Yes	12-Yes

Supplemental Table 1. Gene expression values changed greater than 2 fold.

Affymetrix ST 1.0 Array ID	0-No	6-No	12-No	0-Yes	6-Yes	12-Yes

Supplemental Table 1. Gene expression values changed greater than 2 fold.

Affymetrix ST 1.0 Array ID	0-No	6-No	12-No	0-Yes	6-Yes	12-Yes

Supplemental Table 1. Gene expression values changed greater than 2 fold.

Affymetrix ST 1.0 Array ID	0-No	6-No	12-No	0-Yes	6-Yes	12-Yes

Supplemental Table 1. Gene expression values changed greater than 2 fold.

Affymetrix ST 1.0 Array ID	0-No	6-No	12-No	0-Yes	6-Yes	12-Yes

Supplemental Table 1. Gene expression values changed greater than 2 fold.

Affymetrix ST 1.0 Array ID	0-No	6-No	12-No	0-Yes	6-Yes	12-Yes

Supplemental Table 1. Gene expression values changed greater than 2 fold.

Affymetrix ST 1.0 Array ID	0-No	6-No	12-No	0-Yes	6-Yes	12-Yes

Supplemental Table 1. Gene expression values changed greater than 2 fold.

Affymetrix ST 1.0 Array ID	0-No	6-No	12-No	0-Yes	6-Yes	12-Yes

Supplemental Table 1. Gene expression values changed greater than 2 fold.

Affymetrix ST 1.0 Array ID	0-No	6-No	12-No	0-Yes	6-Yes	12-Yes

Supplemental Table 1. Gene expression values changed greater than 2 fold.

Affymetrix ST 1.0 Array ID	0-No	6-No	12-No	0-Yes	6-Yes	12-Yes

Supplemental Table 1. Gene expression values changed greater than 2 fold.

Affymetrix ST 1.0 Array ID	0-No	6-No	12-No	0-Yes	6-Yes	12-Yes

Supplemental Table 1. Gene expression values changed greater than 2 fold.

Affymetrix ST 1.0 Array ID	0-No	6-No	12-No	0-Yes	6-Yes	12-Yes

Supplemental Table 1. Gene expression values changed greater than 2 fold.

Affymetrix ST 1.0 Array ID	0-No	6-No	12-No	0-Yes	6-Yes	12-Yes

Supplemental Table 1. Gene expression values changed greater than 2 fold.

Affymetrix ST 1.0 Array ID	0-No	6-No	12-No	0-Yes	6-Yes	12-Yes

Supplemental Table 1. Gene expression values changed greater than 2 fold.

Affymetrix ST 1.0 Array ID	0-No	6-No	12-No	0-Yes	6-Yes	12-Yes

Supplemental Table 1. Gene expression values changed greater than 2 fold.

Affymetrix ST 1.0 Array ID	0-No	6-No	12-No	0-Yes	6-Yes	12-Yes

Supplemental Table 1. Gene expression values changed greater than 2 fold.

Affymetrix ST 1.0 Array ID	0-No	6-No	12-No	0-Yes	6-Yes	12-Yes

Supplemental Table 1. Gene expression values changed greater than 2 fold.

Affymetrix ST 1.0 Array ID	0-No	6-No	12-No	0-Yes	6-Yes	12-Yes

Supplemental Table 1. Gene expression values changed greater than 2 fold.

Affymetrix ST 1.0 Array ID	0-No	6-No	12-No	0-Yes	6-Yes	12-Yes

Supplemental Table 1. Gene expression values changed greater than 2 fold.

Affymetrix ST 1.0 Array ID	0-No	6-No	12-No	0-Yes	6-Yes	12-Yes

Supplemental Table 1. Gene expression values changed greater than 2 fold.

Affymetrix ST 1.0 Array ID	0-No	6-No	12-No	0-Yes	6-Yes	12-Yes

Supplemental Table 1. Gene expression values changed greater than 2 fold.

Affymetrix ST 1.0 Array ID	0-No	6-No	12-No	0-Yes	6-Yes	12-Yes

Supplemental Table 1. Gene expression values changed greater than 2 fold.

Affymetrix ST 1.0 Array ID	0-No	6-No	12-No	0-Yes	6-Yes	12-Yes

Supplemental Table 1. Gene expression values changed greater than 2 fold.

Affymetrix ST 1.0 Array ID	0-No	6-No	12-No	0-Yes	6-Yes	12-Yes

Supplemental Table 1. Gene expression values changed greater than 2 fold.

Affymetrix ST 1.0 Array ID	0-No	6-No	12-No	0-Yes	6-Yes	12-Yes

Supplemental Table 1. Gene expression values changed greater than 2 fold.

Affymetrix ST 1.0 Array ID	0-No	6-No	12-No	0-Yes	6-Yes	12-Yes

Supplemental Table 1. Gene expression values changed greater than 2 fold.

Affymetrix ST 1.0 Array ID	0-No	6-No	12-No	0-Yes	6-Yes	12-Yes

Supplemental Table 1. Gene expression values changed greater than 2 fold.

Affymetrix ST 1.0 Array ID	0-No	6-No	12-No	0-Yes	6-Yes	12-Yes

Supplemental Table 1. Gene expression values changed greater than 2 fold.

Affymetrix ST 1.0 Array ID	0-No	6-No	12-No	0-Yes	6-Yes	12-Yes

Supplemental Table 1. Gene expression values changed greater than 2 fold.

Affymetrix ST 1.0 Array ID	0-No	6-No	12-No	0-Yes	6-Yes	12-Yes

Supplemental Table 1. Gene expression values changed greater than 2 fold.

Affymetrix ST 1.0 Array ID	0-No	6-No	12-No	0-Yes	6-Yes	12-Yes

Supplemental Table 1. Gene expression values changed greater than 2 fold.

Affymetrix ST 1.0 Array ID	0-No	6-No	12-No	0-Yes	6-Yes	12-Yes

Supplemental Table 1. Gene expression values changed greater than 2 fold.

Affymetrix ST 1.0 Array ID	0-No	6-No	12-No	0-Yes	6-Yes	12-Yes

Supplemental Table 1. Gene expression values changed greater than 2 fold.

Affymetrix ST 1.0 Array ID	0-No	6-No	12-No	0-Yes	6-Yes	12-Yes

Supplemental Table 1. Gene expression values changed greater than 2 fold.

Affymetrix ST 1.0 Array ID	0-No	6-No	12-No	0-Yes	6-Yes	12-Yes

Supplemental Table 1. Gene expression values changed greater than 2 fold.

Affymetrix ST 1.0 Array ID	0-No	6-No	12-No	0-Yes	6-Yes	12-Yes

Supplemental Table 1. Gene expression values changed greater than 2 fold.

Affymetrix ST 1.0 Array ID	0-No	6-No	12-No	0-Yes	6-Yes	12-Yes

Supplemental Table 1. Gene expression values changed greater than 2 fold.

Affymetrix ST 1.0 Array ID	0-No	6-No	12-No	0-Yes	6-Yes	12-Yes

Supplemental Table 1. Gene expression values changed greater than 2 fold.

Affymetrix ST 1.0 Array ID	0-No	6-No	12-No	0-Yes	6-Yes	12-Yes

Supplemental Table 1. Gene expression values changed greater than 2 fold.

Affymetrix ST 1.0 Array ID	0-No	6-No	12-No	0-Yes	6-Yes	12-Yes

Supplemental Table 1. Gene expression values changed greater than 2 fold.

Affymetrix ST 1.0 Array ID	0-No	6-No	12-No	0-Yes	6-Yes	12-Yes

Supplemental Table 1. Gene expression values changed greater than 2 fold.

Affymetrix ST 1.0 Array ID	0-No	6-No	12-No	0-Yes	6-Yes	12-Yes

Supplemental Table 1. Gene expression values changed greater than 2 fold.

Affymetrix ST 1.0 Array ID	0-No	6-No	12-No	0-Yes	6-Yes	12-Yes

Supplemental Table 1. Gene expression values changed greater than 2 fold.

Affymetrix ST 1.0 Array ID	0-No	6-No	12-No	0-Yes	6-Yes	12-Yes

Supplemental Table 1. Gene expression values changed greater than 2 fold.

Affymetrix ST 1.0 Array ID	0-No	6-No	12-No	0-Yes	6-Yes	12-Yes

Supplemental Table 1. Gene expression values changed greater than 2 fold.

Affymetrix ST 1.0 Array ID	0-No	6-No	12-No	0-Yes	6-Yes	12-Yes

Supplemental Table 1. Gene expression values changed greater than 2 fold.

Affymetrix ST 1.0 Array ID	0-No	6-No	12-No	0-Yes	6-Yes	12-Yes

Supplemental Table 1. Gene expression values changed greater than 2 fold.

Affymetrix ST 1.0 Array ID	0-No	6-No	12-No	0-Yes	6-Yes	12-Yes

Supplemental Table 1. Gene expression values changed greater than 2 fold.

Affymetrix ST 1.0 Array ID	0-No	6-No	12-No	0-Yes	6-Yes	12-Yes

Supplemental Table 1. Gene expression values changed greater than 2 fold.

Affymetrix ST 1.0 Array ID	0-No	6-No	12-No	0-Yes	6-Yes	12-Yes

Supplemental Table 1. Gene expression values changed greater than 2 fold.

Affymetrix ST 1.0 Array ID	0-No	6-No	12-No	0-Yes	6-Yes	12-Yes

Supplemental Table 1. Gene expression values changed greater than 2 fold.

Affymetrix ST 1.0 Array ID	0-No	6-No	12-No	0-Yes	6-Yes	12-Yes

Supplemental Table 1. Gene expression values changed greater than 2 fold.

Affymetrix ST 1.0 Array ID	0-No	6-No	12-No	0-Yes	6-Yes	12-Yes

Supplemental Table 1. Gene expression values changed greater than 2 fold.

Affymetrix ST 1.0 Array ID	0-No	6-No	12-No	0-Yes	6-Yes	12-Yes

Supplemental Table 1. Gene expression values changed greater than 2 fold.

Affymetrix ST 1.0 Array ID	0-No	6-No	12-No	0-Yes	6-Yes	12-Yes

Supplemental Table 1. Gene expression values changed greater than 2 fold.

Affymetrix ST 1.0 Array ID	0-No	6-No	12-No	0-Yes	6-Yes	12-Yes

Supplemental Table 1. Gene expression values changed greater than 2 fold.

Affymetrix ST 1.0 Array ID	0-No	6-No	12-No	0-Yes	6-Yes	12-Yes

Supplemental Table 1. Gene expression values changed greater than 2 fold.

Affymetrix ST 1.0 Array ID	0-No	6-No	12-No	0-Yes	6-Yes	12-Yes

Supplemental Table 1. Gene expression values changed greater than 2 fold.

Affymetrix ST 1.0 Array ID	0-No	6-No	12-No	0-Yes	6-Yes	12-Yes

Supplemental Table 1. Gene expression values changed greater than 2 fold.

Affymetrix ST 1.0 Array ID	0-No	6-No	12-No	0-Yes	6-Yes	12-Yes

Supplemental Table 1. Gene expression values changed greater than 2 fold.

Affymetrix ST 1.0 Array ID	0-No	6-No	12-No	0-Yes	6-Yes	12-Yes

Supplemental Table 1. Gene expression values changed greater than 2 fold.

Affymetrix ST 1.0 Array ID	0-No	6-No	12-No	0-Yes	6-Yes	12-Yes

Supplemental Table 1. Gene expression values changed greater than 2 fold.

Affymetrix ST 1.0 Array ID	0-No	6-No	12-No	0-Yes	6-Yes	12-Yes

Supplemental Table 1. Gene expression values changed greater than 2 fold.

Affymetrix ST 1.0 Array ID	0-No	6-No	12-No	0-Yes	6-Yes	12-Yes

Supplemental Table 1. Gene expression values changed greater than 2 fold.

Affymetrix ST 1.0 Array ID	0-No	6-No	12-No	0-Yes	6-Yes	12-Yes

Supplemental Table 1. Gene expression values changed greater than 2 fold.

Affymetrix ST 1.0 Array ID	0-No	6-No	12-No	0-Yes	6-Yes	12-Yes

Supplemental Table 1. Gene expression values changed greater than 2 fold.

Affymetrix ST 1.0 Array ID	0-No	6-No	12-No	0-Yes	6-Yes	12-Yes

Supplemental Table 1. Gene expression values changed greater than 2 fold.

Affymetrix ST 1.0 Array ID	0-No	6-No	12-No	0-Yes	6-Yes	12-Yes

Supplemental Table 1. Gene expression values changed greater than 2 fold.

Affymetrix ST 1.0 Array ID	0-No	6-No	12-No	0-Yes	6-Yes	12-Yes

Supplemental Table 1. Gene expression values changed greater than 2 fold.

Affymetrix ST 1.0 Array ID	0-No	6-No	12-No	0-Yes	6-Yes	12-Yes

Supplemental Table 1. Gene expression values changed greater than 2 fold.

Affymetrix ST 1.0 Array ID	0-No	6-No	12-No	0-Yes	6-Yes	12-Yes

Supplemental Table 1. Gene expression values changed greater than 2 fold.

Affymetrix ST 1.0 Array ID	0-No	6-No	12-No	0-Yes	6-Yes	12-Yes

Supplemental Table 1. Gene expression values changed greater than 2 fold.

Affymetrix ST 1.0 Array ID	0-No	6-No	12-No	0-Yes	6-Yes	12-Yes

Supplemental Table 1. Gene expression values changed greater than 2 fold.

Affymetrix ST 1.0 Array ID	0-No	6-No	12-No	0-Yes	6-Yes	12-Yes

Supplemental Table 1. Gene expression values changed greater than 2 fold.

Affymetrix ST 1.0 Array ID	0-No	6-No	12-No	0-Yes	6-Yes	12-Yes

Supplemental Table 1. Gene expression values changed greater than 2 fold.

Affymetrix ST 1.0 Array ID	0-No	6-No	12-No	0-Yes	6-Yes	12-Yes

Supplemental Table 1. Gene expression values changed greater than 2 fold.

Affymetrix ST 1.0 Array ID	0-No	6-No	12-No	0-Yes	6-Yes	12-Yes

Supplemental Table 1. Gene expression values changed greater than 2 fold.

Affymetrix ST 1.0 Array ID	0-No	6-No	12-No	0-Yes	6-Yes	12-Yes

Supplemental Table 1. Gene expression values changed greater than 2 fold.

Affymetrix ST 1.0 Array ID	0-No	6-No	12-No	0-Yes	6-Yes	12-Yes

Supplemental Table 1. Gene expression values changed greater than 2 fold.

Affymetrix ST 1.0 Array ID	0-No	6-No	12-No	0-Yes	6-Yes	12-Yes

Supplemental Table 1. Gene expression values changed greater than 2 fold.

Affymetrix ST 1.0 Array ID	0-No	6-No	12-No	0-Yes	6-Yes	12-Yes

Supplemental Table 1. Gene expression values changed greater than 2 fold.

Affymetrix ST 1.0 Array ID	0-No	6-No	12-No	0-Yes	6-Yes	12-Yes

Supplemental Table 1. Gene expression values changed greater than 2 fold.

Affymetrix ST 1.0 Array ID	0-No	6-No	12-No	0-Yes	6-Yes	12-Yes

Supplemental Table 1. Gene expression values changed greater than 2 fold.

Affymetrix ST 1.0 Array ID	0-No	6-No	12-No	0-Yes	6-Yes	12-Yes

Supplemental Table 1. Gene expression values changed greater than 2 fold.

Affymetrix ST 1.0 Array ID	0-No	6-No	12-No	0-Yes	6-Yes	12-Yes

Supplemental Table 1. Gene expression values changed greater than 2 fold.

Affymetrix ST 1.0 Array ID	0-No	6-No	12-No	0-Yes	6-Yes	12-Yes

Supplemental Table 1. Gene expression values changed greater than 2 fold.

Affymetrix ST 1.0 Array ID	0-No	6-No	12-No	0-Yes	6-Yes	12-Yes

Supplemental Table 1. Gene expression values changed greater than 2 fold.

Affymetrix ST 1.0 Array ID	0-No	6-No	12-No	0-Yes	6-Yes	12-Yes

Supplemental Table 1. Gene expression values changed greater than 2 fold.

Affymetrix ST 1.0 Array ID	0-No	6-No	12-No	0-Yes	6-Yes	12-Yes

Supplemental Table 1. Gene expression values changed greater than 2 fold.

Affymetrix ST 1.0 Array ID	0-No	6-No	12-No	0-Yes	6-Yes	12-Yes

Supplemental Table 1. Gene expression values changed greater than 2 fold.

Affymetrix ST 1.0 Array ID	0-No	6-No	12-No	0-Yes	6-Yes	12-Yes

Supplemental Table 1. Gene expression values changed greater than 2 fold.

Affymetrix ST 1.0 Array ID	0-No	6-No	12-No	0-Yes	6-Yes	12-Yes

Supplemental Table 1. Gene expression values changed greater than 2 fold.

Affymetrix ST 1.0 Array ID	0-No	6-No	12-No	0-Yes	6-Yes	12-Yes

Supplemental Table 1. Gene expression values changed greater than 2 fold.

Affymetrix ST 1.0 Array ID	0-No	6-No	12-No	0-Yes	6-Yes	12-Yes

Supplemental Table 1. Gene expression values changed greater than 2 fold.

Affymetrix ST 1.0 Array ID	0-No	6-No	12-No	0-Yes	6-Yes	12-Yes

Supplemental Table 1. Gene expression values changed greater than 2 fold.

Affymetrix ST 1.0 Array ID	0-No	6-No	12-No	0-Yes	6-Yes	12-Yes

Supplemental Table 1. Gene expression values changed greater than 2 fold.

Affymetrix ST 1.0 Array ID	0-No	6-No	12-No	0-Yes	6-Yes	12-Yes

Supplemental Table 1. Gene expression values changed greater than 2 fold.

Affymetrix ST 1.0 Array ID	0-No	6-No	12-No	0-Yes	6-Yes	12-Yes

Supplemental Table 1. Gene expression values changed greater than 2 fold.

Affymetrix ST 1.0 Array ID	0-No	6-No	12-No	0-Yes	6-Yes	12-Yes

Supplemental Table 1. Gene expression values changed greater than 2 fold.

Affymetrix ST 1.0 Array ID	0-No	6-No	12-No	0-Yes	6-Yes	12-Yes

Supplemental Table 1. Gene expression values changed greater than 2 fold.

Affymetrix ST 1.0 Array ID	0-No	6-No	12-No	0-Yes	6-Yes	12-Yes

Supplemental Table 1. Gene expression values changed greater than 2 fold.

Affymetrix ST 1.0 Array ID	0-No	6-No	12-No	0-Yes	6-Yes	12-Yes

Supplemental Table 1. Gene expression values changed greater than 2 fold.

Affymetrix ST 1.0 Array ID	0-No	6-No	12-No	0-Yes	6-Yes	12-Yes

Supplemental Table 1. Gene expression values changed greater than 2 fold.

Affymetrix ST 1.0 Array ID	0-No	6-No	12-No	0-Yes	6-Yes	12-Yes

Supplemental Table 1. Gene expression values changed greater than 2 fold.

Affymetrix ST 1.0 Array ID	0-No	6-No	12-No	0-Yes	6-Yes	12-Yes

Supplemental Table 1. Gene expression values changed greater than 2 fold.

Affymetrix ST 1.0 Array ID	0-No	6-No	12-No	0-Yes	6-Yes	12-Yes

Supplemental Table 1. Gene expression values changed greater than 2 fold.

Affymetrix ST 1.0 Array ID	0-No	6-No	12-No	0-Yes	6-Yes	12-Yes

Supplemental Table 1. Gene expression values changed greater than 2 fold.

Affymetrix ST 1.0 Array ID	0-No	6-No	12-No	0-Yes	6-Yes	12-Yes

Supplemental Table 1. Gene expression values changed greater than 2 fold.

Affymetrix ST 1.0 Array ID	0-No	6-No	12-No	0-Yes	6-Yes	12-Yes

Supplemental Table 1. Gene expression values changed greater than 2 fold.

Affymetrix ST 1.0 Array ID	0-No	6-No	12-No	0-Yes	6-Yes	12-Yes

Supplemental Table 1. Gene expression values changed greater than 2 fold.

Affymetrix ST 1.0 Array ID	0-No	6-No	12-No	0-Yes	6-Yes	12-Yes

Supplemental Table 1. Gene expression values changed greater than 2 fold.

Affymetrix ST 1.0 Array ID	0-No	6-No	12-No	0-Yes	6-Yes	12-Yes

Supplemental Table 1. Gene expression values changed greater than 2 fold.

Affymetrix ST 1.0 Array ID	0-No	6-No	12-No	0-Yes	6-Yes	12-Yes

Supplemental Table 1. Gene expression values changed greater than 2 fold.

Affymetrix ST 1.0 Array ID	0-No	6-No	12-No	0-Yes	6-Yes	12-Yes

Supplemental Table 1. Gene expression values changed greater than 2 fold.

Affymetrix ST 1.0 Array ID	0-No	6-No	12-No	0-Yes	6-Yes	12-Yes

Supplemental Table 1. Gene expression values changed greater than 2 fold.

Affymetrix ST 1.0 Array ID	0-No	6-No	12-No	0-Yes	6-Yes	12-Yes

Supplemental Table 1. Gene expression values changed greater than 2 fold.

Affymetrix ST 1.0 Array ID	0-No	6-No	12-No	0-Yes	6-Yes	12-Yes

Supplemental Table 1. Gene expression values changed greater than 2 fold.

Affymetrix ST 1.0 Array ID	0-No	6-No	12-No	0-Yes	6-Yes	12-Yes

Supplemental Table 1. Gene expression values changed greater than 2 fold.

Affymetrix ST 1.0 Array ID	0-No	6-No	12-No	0-Yes	6-Yes	12-Yes

Supplemental Table 1. Gene expression values changed greater than 2 fold.

Affymetrix ST 1.0 Array ID	0-No	6-No	12-No	0-Yes	6-Yes	12-Yes

Supplemental Table 1. Gene expression values changed greater than 2 fold.

Affymetrix ST 1.0 Array ID	0-No	6-No	12-No	0-Yes	6-Yes	12-Yes

Supplemental Table 1. Gene expression values changed greater than 2 fold.

Affymetrix ST 1.0 Array ID	0-No	6-No	12-No	0-Yes	6-Yes	12-Yes

Supplemental Table 1. Gene expression values changed greater than 2 fold.

Affymetrix ST 1.0 Array ID	0-No	6-No	12-No	0-Yes	6-Yes	12-Yes

Supplemental Table 1. Gene expression values changed greater than 2 fold.

Affymetrix ST 1.0 Array ID	0-No	6-No	12-No	0-Yes	6-Yes	12-Yes

Supplemental Table 1. Gene expression values changed greater than 2 fold.

Affymetrix ST 1.0 Array ID	0-No	6-No	12-No	0-Yes	6-Yes	12-Yes

Supplemental Table 1. Gene expression values changed greater than 2 fold.

Affymetrix ST 1.0 Array ID	0-No	6-No	12-No	0-Yes	6-Yes	12-Yes

Supplemental Table 1. Gene expression values changed greater than 2 fold.

Affymetrix ST 1.0 Array ID	0-No	6-No	12-No	0-Yes	6-Yes	12-Yes

Supplemental Table 1. Gene expression values changed greater than 2 fold.

Affymetrix ST 1.0 Array ID	0-No	6-No	12-No	0-Yes	6-Yes	12-Yes

Supplemental Table 1. Gene expression values changed greater than 2 fold.

Affymetrix ST 1.0 Array ID	0-No	6-No	12-No	0-Yes	6-Yes	12-Yes

Supplemental Table 1. Gene expression values changed greater than 2 fold.

Affymetrix ST 1.0 Array ID	0-No	6-No	12-No	0-Yes	6-Yes	12-Yes

Supplemental Table 1. Gene expression values changed greater than 2 fold.

Affymetrix ST 1.0 Array ID	0-No	6-No	12-No	0-Yes	6-Yes	12-Yes

Supplemental Table 1. Gene expression values changed greater than 2 fold.

Affymetrix ST 1.0 Array ID	0-No	6-No	12-No	0-Yes	6-Yes	12-Yes

Supplemental Table 1. Gene expression values changed greater than 2 fold.

Affymetrix ST 1.0 Array ID	0-No	6-No	12-No	0-Yes	6-Yes	12-Yes

Supplemental Table 1. Gene expression values changed greater than 2 fold.

Affymetrix ST 1.0 Array ID	0-No	6-No	12-No	0-Yes	6-Yes	12-Yes

Supplemental Table 1. Gene expression values changed greater than 2 fold.

Affymetrix ST 1.0 Array ID	0-No	6-No	12-No	0-Yes	6-Yes	12-Yes

Supplemental Table 1. Gene expression values changed greater than 2 fold.

Affymetrix ST 1.0 Array ID	0-No	6-No	12-No	0-Yes	6-Yes	12-Yes

Supplemental Table 1. Gene expression values changed greater than 2 fold.

Affymetrix ST 1.0 Array ID	0-No	6-No	12-No	0-Yes	6-Yes	12-Yes

Supplemental Table 1. Gene expression values changed greater than 2 fold.

Affymetrix ST 1.0 Array ID	0-No	6-No	12-No	0-Yes	6-Yes	12-Yes

Supplemental Table 1. Gene expression values changed greater than 2 fold.

Affymetrix ST 1.0 Array ID	0-No	6-No	12-No	0-Yes	6-Yes	12-Yes

Supplemental Table 1. Gene expression values changed greater than 2 fold.

Affymetrix ST 1.0 Array ID	0-No	6-No	12-No	0-Yes	6-Yes	12-Yes

Supplemental Table 1. Gene expression values changed greater than 2 fold.

Affymetrix ST 1.0 Array ID	0-No	6-No	12-No	0-Yes	6-Yes	12-Yes

Supplemental Table 1. Gene expression values changed greater than 2 fold.

Affymetrix ST 1.0 Array ID	0-No	6-No	12-No	0-Yes	6-Yes	12-Yes

Supplemental Table 1. Gene expression values changed greater than 2 fold.

Affymetrix ST 1.0 Array ID	0-No	6-No	12-No	0-Yes	6-Yes	12-Yes

Supplemental Table 1. Gene expression values changed greater than 2 fold.

Affymetrix ST 1.0 Array ID	0-No	6-No	12-No	0-Yes	6-Yes	12-Yes

Supplemental Table 1. Gene expression values changed greater than 2 fold.

Affymetrix ST 1.0 Array ID	0-No	6-No	12-No	0-Yes	6-Yes	12-Yes

Supplemental Table 1. Gene expression values changed greater than 2 fold.

Affymetrix ST 1.0 Array ID	0-No	6-No	12-No	0-Yes	6-Yes	12-Yes

Supplemental Table 1. Gene expression values changed greater than 2 fold.

Affymetrix ST 1.0 Array ID	0-No	6-No	12-No	0-Yes	6-Yes	12-Yes

Supplemental Table 1. Gene expression values changed greater than 2 fold.

Affymetrix ST 1.0 Array ID	0-No	6-No	12-No	0-Yes	6-Yes	12-Yes

Supplemental Table 1. Gene expression values changed greater than 2 fold.

Affymetrix ST 1.0 Array ID	0-No	6-No	12-No	0-Yes	6-Yes	12-Yes

Supplemental Table 1. Gene expression values changed greater than 2 fold.

Affymetrix ST 1.0 Array ID	0-No	6-No	12-No	0-Yes	6-Yes	12-Yes

Supplemental Table 1. Gene expression values changed greater than 2 fold.

Affymetrix ST 1.0 Array ID	0-No	6-No	12-No	0-Yes	6-Yes	12-Yes

Supplemental Table 1. Gene expression values changed greater than 2 fold.

Affymetrix ST 1.0 Array ID	0-No	6-No	12-No	0-Yes	6-Yes	12-Yes

Supplemental Table 1. Gene expression values changed greater than 2 fold.

Affymetrix ST 1.0 Array ID	0-No	6-No	12-No	0-Yes	6-Yes	12-Yes

Supplemental Table 1. Gene expression values changed greater than 2 fold.

Affymetrix ST 1.0 Array ID	0-No	6-No	12-No	0-Yes	6-Yes	12-Yes

Supplemental Table 1. Gene expression values changed greater than 2 fold.

Affymetrix ST 1.0 Array ID	0-No	6-No	12-No	0-Yes	6-Yes	12-Yes

Supplemental Table 1. Gene expression values changed greater than 2 fold.

Affymetrix ST 1.0 Array ID	0-No	6-No	12-No	0-Yes	6-Yes	12-Yes

Supplemental Table 1. Gene expression values changed greater than 2 fold.

Affymetrix ST 1.0 Array ID	0-No	6-No	12-No	0-Yes	6-Yes	12-Yes

Supplemental Table 1. Gene expression values changed greater than 2 fold.

Affymetrix ST 1.0 Array ID	0-No	6-No	12-No	0-Yes	6-Yes	12-Yes

Supplemental Table 1. Gene expression values changed greater than 2 fold.

Affymetrix ST 1.0 Array ID	0-No	6-No	12-No	0-Yes	6-Yes	12-Yes

Supplemental Table 1. Gene expression values changed greater than 2 fold.

Affymetrix ST 1.0 Array ID	0-No	6-No	12-No	0-Yes	6-Yes	12-Yes

Supplemental Table 1. Gene expression values changed greater than 2 fold.

Affymetrix ST 1.0 Array ID	0-No	6-No	12-No	0-Yes	6-Yes	12-Yes

Supplemental Table 1. Gene expression values changed greater than 2 fold.

Affymetrix ST 1.0 Array ID	0-No	6-No	12-No	0-Yes	6-Yes	12-Yes

Supplemental Table 1. Gene expression values changed greater than 2 fold.

Affymetrix ST 1.0 Array ID	0-No	6-No	12-No	0-Yes	6-Yes	12-Yes

Supplemental Table 1. Gene expression values changed greater than 2 fold.

Affymetrix ST 1.0 Array ID	0-No	6-No	12-No	0-Yes	6-Yes	12-Yes

Supplemental Table 1. Gene expression values changed greater than 2 fold.

Affymetrix ST 1.0 Array ID	0-No	6-No	12-No	0-Yes	6-Yes	12-Yes

Supplemental Table 1. Gene expression values changed greater than 2 fold.

Affymetrix ST 1.0 Array ID	0-No	6-No	12-No	0-Yes	6-Yes	12-Yes

Supplemental Table 1. Gene expression values changed greater than 2 fold.

Affymetrix ST 1.0 Array ID	0-No	6-No	12-No	0-Yes	6-Yes	12-Yes

Supplemental Table 1. Gene expression values changed greater than 2 fold.

Affymetrix ST 1.0 Array ID	0-No	6-No	12-No	0-Yes	6-Yes	12-Yes

Supplemental Table 1. Gene expression values changed greater than 2 fold.

Affymetrix ST 1.0 Array ID	0-No	6-No	12-No	0-Yes	6-Yes	12-Yes

Supplemental Table 1. Gene expression values changed greater than 2 fold.

Affymetrix ST 1.0 Array ID	0-No	6-No	12-No	0-Yes	6-Yes	12-Yes

Supplemental Table 1. Gene expression values changed greater than 2 fold.

Affymetrix ST 1.0 Array ID	0-No	6-No	12-No	0-Yes	6-Yes	12-Yes

Supplemental Table 1. Gene expression values changed greater than 2 fold.

Affymetrix ST 1.0 Array ID	0-No	6-No	12-No	0-Yes	6-Yes	12-Yes

Supplemental Table 1. Gene expression values changed greater than 2 fold.

Affymetrix ST 1.0 Array ID	0-No	6-No	12-No	0-Yes	6-Yes	12-Yes

Supplemental Table 1. Gene expression values changed greater than 2 fold.

Affymetrix ST 1.0 Array ID	0-No	6-No	12-No	0-Yes	6-Yes	12-Yes

Supplemental Table 1. Gene expression values changed greater than 2 fold.

Affymetrix ST 1.0 Array ID	0-No	6-No	12-No	0-Yes	6-Yes	12-Yes

Supplemental Table 1. Gene expression values changed greater than 2 fold.

Affymetrix ST 1.0 Array ID	0-No	6-No	12-No	0-Yes	6-Yes	12-Yes

Supplemental Table 1. Gene expression values changed greater than 2 fold.

Affymetrix ST 1.0 Array ID	0-No	6-No	12-No	0-Yes	6-Yes	12-Yes

Supplemental Table 1. Gene expression values changed greater than 2 fold.

Affymetrix ST 1.0 Array ID	0-No	6-No	12-No	0-Yes	6-Yes	12-Yes

Supplemental Table 1. Gene expression values changed greater than 2 fold.

Affymetrix ST 1.0 Array ID	0-No	6-No	12-No	0-Yes	6-Yes	12-Yes

Supplemental Table 1. Gene expression values changed greater than 2 fold.

Affymetrix ST 1.0 Array ID	0-No	6-No	12-No	0-Yes	6-Yes	12-Yes

Supplemental Table 1. Gene expression values changed greater than 2 fold.

Affymetrix ST 1.0 Array ID	0-No	6-No	12-No	0-Yes	6-Yes	12-Yes

Supplemental Table 1. Gene expression values changed greater than 2 fold.

Affymetrix ST 1.0 Array ID	0-No	6-No	12-No	0-Yes	6-Yes	12-Yes

Supplemental Table 1. Gene expression values changed greater than 2 fold.

Affymetrix ST 1.0 Array ID	0-No	6-No	12-No	0-Yes	6-Yes	12-Yes

Supplemental Table 1. Gene expression values changed greater than 2 fold.

Affymetrix ST 1.0 Array ID	0-No	6-No	12-No	0-Yes	6-Yes	12-Yes

Supplemental Table 1. Gene expression values changed greater than 2 fold.

Affymetrix ST 1.0 Array ID	0-No	6-No	12-No	0-Yes	6-Yes	12-Yes

Supplemental Table 1. Gene expression values changed greater than 2 fold.

Affymetrix ST 1.0 Array ID	0-No	6-No	12-No	0-Yes	6-Yes	12-Yes

Supplemental Table 1. Gene expression values changed greater than 2 fold.

Affymetrix ST 1.0 Array ID	0-No	6-No	12-No	0-Yes	6-Yes	12-Yes

Supplemental Table 1. Gene expression values changed greater than 2 fold.

Affymetrix ST 1.0 Array ID	0-No	6-No	12-No	0-Yes	6-Yes	12-Yes

Supplemental Table 1. Gene expression values changed greater than 2 fold.

Affymetrix ST 1.0 Array ID	0-No	6-No	12-No	0-Yes	6-Yes	12-Yes

Supplemental Table 1. Gene expression values changed greater than 2 fold.

Affymetrix ST 1.0 Array ID	0-No	6-No	12-No	0-Yes	6-Yes	12-Yes

Supplemental Table 1. Gene expression values changed greater than 2 fold.

Affymetrix ST 1.0 Array ID	0-No	6-No	12-No	0-Yes	6-Yes	12-Yes

Supplemental Table 1. Gene expression values changed greater than 2 fold.

Affymetrix ST 1.0 Array ID	0-No	6-No	12-No	0-Yes	6-Yes	12-Yes

Supplemental Table 1. Gene expression values changed greater than 2 fold.

Affymetrix ST 1.0 Array ID	0-No	6-No	12-No	0-Yes	6-Yes	12-Yes

Supplemental Table 1. Gene expression values changed greater than 2 fold.

Affymetrix ST 1.0 Array ID	0-No	6-No	12-No	0-Yes	6-Yes	12-Yes

Supplemental Table 1. Gene expression values changed greater than 2 fold.

Affymetrix ST 1.0 Array ID	0-No	6-No	12-No	0-Yes	6-Yes	12-Yes

Supplemental Table 1. Gene expression values changed greater than 2 fold.

Affymetrix ST 1.0 Array ID	0-No	6-No	12-No	0-Yes	6-Yes	12-Yes

Supplemental Table 1. Gene expression values changed greater than 2 fold.

Affymetrix ST 1.0 Array ID	0-No	6-No	12-No	0-Yes	6-Yes	12-Yes

Supplemental Table 1. Gene expression values changed greater than 2 fold.

Affymetrix ST 1.0 Array ID	0-No	6-No	12-No	0-Yes	6-Yes	12-Yes

Supplemental Table 1. Gene expression values changed greater than 2 fold.

Affymetrix ST 1.0 Array ID	0-No	6-No	12-No	0-Yes	6-Yes	12-Yes

Supplemental Table 1. Gene expression values changed greater than 2 fold.

Affymetrix ST 1.0 Array ID	0-No	6-No	12-No	0-Yes	6-Yes	12-Yes

Supplemental Table 1. Gene expression values changed greater than 2 fold.

Affymetrix ST 1.0 Array ID	0-No	6-No	12-No	0-Yes	6-Yes	12-Yes

Supplemental Table 1. Gene expression values changed greater than 2 fold.

Affymetrix ST 1.0 Array ID	0-No	6-No	12-No	0-Yes	6-Yes	12-Yes

Supplemental Table 1. Gene expression values changed greater than 2 fold.

Affymetrix ST 1.0 Array ID	0-No	6-No	12-No	0-Yes	6-Yes	12-Yes

Supplemental Table 1. Gene expression values changed greater than 2 fold.

Affymetrix ST 1.0 Array ID	0-No	6-No	12-No	0-Yes	6-Yes	12-Yes

Supplemental Table 1. Gene expression values changed greater than 2 fold.

Affymetrix ST 1.0 Array ID	0-No	6-No	12-No	0-Yes	6-Yes	12-Yes

Supplemental Table 1. Gene expression values changed greater than 2 fold.

Affymetrix ST 1.0 Array ID	0-No	6-No	12-No	0-Yes	6-Yes	12-Yes

Supplemental Table 1. Gene expression values changed greater than 2 fold.

Affymetrix ST 1.0 Array ID	0-No	6-No	12-No	0-Yes	6-Yes	12-Yes

Supplemental Table 1. Gene expression values changed greater than 2 fold.

Affymetrix ST 1.0 Array ID	0-No	6-No	12-No	0-Yes	6-Yes	12-Yes

Supplemental Table 1. Gene expression values changed greater than 2 fold.

Affymetrix ST 1.0 Array ID	0-No	6-No	12-No	0-Yes	6-Yes	12-Yes

Supplemental Table 1. Gene expression values changed greater than 2 fold.

Affymetrix ST 1.0 Array ID	0-No	6-No	12-No	0-Yes	6-Yes	12-Yes

Supplemental Table 1. Gene expression values changed greater than 2 fold.

Affymetrix ST 1.0 Array ID	0-No	6-No	12-No	0-Yes	6-Yes	12-Yes

Supplemental Table 1. Gene expression values changed greater than 2 fold.

Affymetrix ST 1.0 Array ID	0-No	6-No	12-No	0-Yes	6-Yes	12-Yes

Supplemental Table 1. Gene expression values changed greater than 2 fold.

Affymetrix ST 1.0 Array ID	0-No	6-No	12-No	0-Yes	6-Yes	12-Yes

Supplemental Table 1. Gene expression values changed greater than 2 fold.

Affymetrix ST 1.0 Array ID	0-No	6-No	12-No	0-Yes	6-Yes	12-Yes

Supplemental Table 1. Gene expression values changed greater than 2 fold.

Affymetrix ST 1.0 Array ID	0-No	6-No	12-No	0-Yes	6-Yes	12-Yes

Supplemental Table 1. Gene expression values changed greater than 2 fold.

Affymetrix ST 1.0 Array ID	0-No	6-No	12-No	0-Yes	6-Yes	12-Yes

Supplemental Table 1. Gene expression values changed greater than 2 fold.

Affymetrix ST 1.0 Array ID	0-No	6-No	12-No	0-Yes	6-Yes	12-Yes

Supplemental Table 1. Gene expression values changed greater than 2 fold.

Affymetrix ST 1.0 Array ID	0-No	6-No	12-No	0-Yes	6-Yes	12-Yes

Supplemental Table 1. Gene expression values changed greater than 2 fold.

Affymetrix ST 1.0 Array ID	0-No	6-No	12-No	0-Yes	6-Yes	12-Yes

Supplemental Table 1. Gene expression values changed greater than 2 fold.

Affymetrix ST 1.0 Array ID	0-No	6-No	12-No	0-Yes	6-Yes	12-Yes

Supplemental Table 1. Gene expression values changed greater than 2 fold.

Affymetrix ST 1.0 Array ID	0-No	6-No	12-No	0-Yes	6-Yes	12-Yes

Supplemental Table 1. Gene expression values changed greater than 2 fold.

Affymetrix ST 1.0 Array ID	0-No	6-No	12-No	0-Yes	6-Yes	12-Yes

Supplemental Table 1. Gene expression values changed greater than 2 fold.

Affymetrix ST 1.0 Array ID	0-No	6-No	12-No	0-Yes	6-Yes	12-Yes

Supplemental Table 1. Gene expression values changed greater than 2 fold.

Affymetrix ST 1.0 Array ID	0-No	6-No	12-No	0-Yes	6-Yes	12-Yes

Supplemental Table 1. Gene expression values changed greater than 2 fold.

Affymetrix ST 1.0 Array ID	0-No	6-No	12-No	0-Yes	6-Yes	12-Yes

Supplemental Table 1. Gene expression values changed greater than 2 fold.

Affymetrix ST 1.0 Array ID	0-No	6-No	12-No	0-Yes	6-Yes	12-Yes

Supplemental Table 1. Gene expression values changed greater than 2 fold.

Affymetrix ST 1.0 Array ID	0-No	6-No	12-No	0-Yes	6-Yes	12-Yes

Supplemental Table 1. Gene expression values changed greater than 2 fold.

Affymetrix ST 1.0 Array ID	0-No	6-No	12-No	0-Yes	6-Yes	12-Yes

Supplemental Table 1. Gene expression values changed greater than 2 fold.

Affymetrix ST 1.0 Array ID	0-No	6-No	12-No	0-Yes	6-Yes	12-Yes

Supplemental Table 1. Gene expression values changed greater than 2 fold.

Affymetrix ST 1.0 Array ID	0-No	6-No	12-No	0-Yes	6-Yes	12-Yes

Supplemental Table 1. Gene expression values changed greater than 2 fold.

Affymetrix ST 1.0 Array ID	0-No	6-No	12-No	0-Yes	6-Yes	12-Yes

Supplemental Table 1. Gene expression values changed greater than 2 fold.

Affymetrix ST 1.0 Array ID	0-No	6-No	12-No	0-Yes	6-Yes	12-Yes

Supplemental Table 1. Gene expression values changed greater than 2 fold.

Affymetrix ST 1.0 Array ID	0-No	6-No	12-No	0-Yes	6-Yes	12-Yes

Supplemental Table 1. Gene expression values changed greater than 2 fold.

Affymetrix ST 1.0 Array ID	0-No	6-No	12-No	0-Yes	6-Yes	12-Yes

Supplemental Table 1. Gene expression values changed greater than 2 fold.

Affymetrix ST 1.0 Array ID	0-No	6-No	12-No	0-Yes	6-Yes	12-Yes

Supplemental Table 1. Gene expression values changed greater than 2 fold.

Affymetrix ST 1.0 Array ID	0-No	6-No	12-No	0-Yes	6-Yes	12-Yes

Supplemental Table 1. Gene expression values changed greater than 2 fold.

Affymetrix ST 1.0 Array ID	0-No	6-No	12-No	0-Yes	6-Yes	12-Yes

Supplemental Table 1. Gene expression values changed greater than 2 fold.

Affymetrix ST 1.0 Array ID	0-No	6-No	12-No	0-Yes	6-Yes	12-Yes

Supplemental Table 1. Gene expression values changed greater than 2 fold.

Affymetrix ST 1.0 Array ID	0-No	6-No	12-No	0-Yes	6-Yes	12-Yes

Supplemental Table 1. Gene expression values changed greater than 2 fold.

Affymetrix ST 1.0 Array ID	0-No	6-No	12-No	0-Yes	6-Yes	12-Yes

Supplemental Table 1. Gene expression values changed greater than 2 fold.

Affymetrix ST 1.0 Array ID	0-No	6-No	12-No	0-Yes	6-Yes	12-Yes

Supplemental Table 1. Gene expression values changed greater than 2 fold.

Affymetrix ST 1.0 Array ID	0-No	6-No	12-No	0-Yes	6-Yes	12-Yes

Supplemental Table 1. Gene expression values changed greater than 2 fold.

Affymetrix ST 1.0 Array ID	0-No	6-No	12-No	0-Yes	6-Yes	12-Yes

Supplemental Table 1. Gene expression values changed greater than 2 fold.

Affymetrix ST 1.0 Array ID	0-No	6-No	12-No	0-Yes	6-Yes	12-Yes

Supplemental Table 1. Gene expression values changed greater than 2 fold.

Affymetrix ST 1.0 Array ID	0-No	6-No	12-No	0-Yes	6-Yes	12-Yes

Supplemental Table 1. Gene expression values changed greater than 2 fold.

Affymetrix ST 1.0 Array ID	0-No	6-No	12-No	0-Yes	6-Yes	12-Yes

Supplemental Table 1. Gene expression values changed greater than 2 fold.

Affymetrix ST 1.0 Array ID	0-No	6-No	12-No	0-Yes	6-Yes	12-Yes

Supplemental Table 1. Gene expression values changed greater than 2 fold.

Affymetrix ST 1.0 Array ID	0-No	6-No	12-No	0-Yes	6-Yes	12-Yes

Supplemental Table 1. Gene expression values changed greater than 2 fold.

Affymetrix ST 1.0 Array ID	0-No	6-No	12-No	0-Yes	6-Yes	12-Yes

Supplemental Table 1. Gene expression values changed greater than 2 fold.

Affymetrix ST 1.0 Array ID	0-No	6-No	12-No	0-Yes	6-Yes	12-Yes

Supplemental Table 1. Gene expression values changed greater than 2 fold.

Affymetrix ST 1.0 Array ID	0-No	6-No	12-No	0-Yes	6-Yes	12-Yes

Supplemental Table 1. Gene expression values changed greater than 2 fold.

Affymetrix ST 1.0 Array ID	0-No	6-No	12-No	0-Yes	6-Yes	12-Yes

Supplemental Table 1. Gene expression values changed greater than 2 fold.

Affymetrix ST 1.0 Array ID	0-No	6-No	12-No	0-Yes	6-Yes	12-Yes

Supplemental Table 1. Gene expression values changed greater than 2 fold.

Affymetrix ST 1.0 Array ID	0-No	6-No	12-No	0-Yes	6-Yes	12-Yes

Supplemental Table 1. Gene expression values changed greater than 2 fold.

Affymetrix ST 1.0 Array ID	0-No	6-No	12-No	0-Yes	6-Yes	12-Yes

Supplemental Table 1. Gene expression values changed greater than 2 fold.

Affymetrix ST 1.0 Array ID	0-No	6-No	12-No	0-Yes	6-Yes	12-Yes

Supplemental Table 1. Gene expression values changed greater than 2 fold.

Affymetrix ST 1.0 Array ID	0-No	6-No	12-No	0-Yes	6-Yes	12-Yes

Supplemental Table 1. Gene expression values changed greater than 2 fold.

Affymetrix ST 1.0 Array ID	0-No	6-No	12-No	0-Yes	6-Yes	12-Yes

Supplemental Table 1. Gene expression values changed greater than 2 fold.

Affymetrix ST 1.0 Array ID	0-No	6-No	12-No	0-Yes	6-Yes	12-Yes

Supplemental Table 1. Gene expression values changed greater than 2 fold.

Affymetrix ST 1.0 Array ID	0-No	6-No	12-No	0-Yes	6-Yes	12-Yes

Supplemental Table 1. Gene expression values changed greater than 2 fold.

Affymetrix ST 1.0 Array ID	0-No	6-No	12-No	0-Yes	6-Yes	12-Yes

Supplemental Table 1. Gene expression values changed greater than 2 fold.

Affymetrix ST 1.0 Array ID	0-No	6-No	12-No	0-Yes	6-Yes	12-Yes

Supplemental Table 1. Gene expression values changed greater than 2 fold.

Affymetrix ST 1.0 Array ID	0-No	6-No	12-No	0-Yes	6-Yes	12-Yes

Supplemental Table 1. Gene expression values changed greater than 2 fold.

Affymetrix ST 1.0 Array ID	0-No	6-No	12-No	0-Yes	6-Yes	12-Yes

Supplemental Table 1. Gene expression values changed greater than 2 fold.

Affymetrix ST 1.0 Array ID	0-No	6-No	12-No	0-Yes	6-Yes	12-Yes

Supplemental Table 1. Gene expression values changed greater than 2 fold.

Affymetrix ST 1.0 Array ID	0-No	6-No	12-No	0-Yes	6-Yes	12-Yes

Supplemental Table 1. Gene expression values changed greater than 2 fold.

Affymetrix ST 1.0 Array ID	0-No	6-No	12-No	0-Yes	6-Yes	12-Yes

Supplemental Table 1. Gene expression values changed greater than 2 fold.

Affymetrix ST 1.0 Array ID	0-No	6-No	12-No	0-Yes	6-Yes	12-Yes

Supplemental Table 1. Gene expression values changed greater than 2 fold.

Affymetrix ST 1.0 Array ID	0-No	6-No	12-No	0-Yes	6-Yes	12-Yes

Supplemental Table 1. Gene expression values changed greater than 2 fold.

Affymetrix ST 1.0 Array ID	0-No	6-No	12-No	0-Yes	6-Yes	12-Yes

Supplemental Table 1. Gene expression values changed greater than 2 fold.

Affymetrix ST 1.0 Array ID	0-No	6-No	12-No	0-Yes	6-Yes	12-Yes

Supplemental Table 1. Gene expression values changed greater than 2 fold.

Affymetrix ST 1.0 Array ID	0-No	6-No	12-No	0-Yes	6-Yes	12-Yes

Supplemental Table 1. Gene expression values changed greater than 2 fold.

Affymetrix ST 1.0 Array ID	0-No	6-No	12-No	0-Yes	6-Yes	12-Yes

Supplemental Table 1. Gene expression values changed greater than 2 fold.

Affymetrix ST 1.0 Array ID	0-No	6-No	12-No	0-Yes	6-Yes	12-Yes

Supplemental Table 1. Gene expression values changed greater than 2 fold.

Affymetrix ST 1.0 Array ID	0-No	6-No	12-No	0-Yes	6-Yes	12-Yes

Supplemental Table 1. Gene expression values changed greater than 2 fold.

Affymetrix ST 1.0 Array ID	0-No	6-No	12-No	0-Yes	6-Yes	12-Yes

Supplemental Table 1. Gene expression values changed greater than 2 fold.

Affymetrix ST 1.0 Array ID	0-No	6-No	12-No	0-Yes	6-Yes	12-Yes

Supplemental Table 1. Gene expression values changed greater than 2 fold.

Affymetrix ST 1.0 Array ID	0-No	6-No	12-No	0-Yes	6-Yes	12-Yes

Supplemental Table 1. Gene expression values changed greater than 2 fold.

Affymetrix ST 1.0 Array ID	0-No	6-No	12-No	0-Yes	6-Yes	12-Yes

Supplemental Table 1. Gene expression values changed greater than 2 fold.

Affymetrix ST 1.0 Array ID	0-No	6-No	12-No	0-Yes	6-Yes	12-Yes

Supplemental Table 1. Gene expression values changed greater than 2 fold.

Affymetrix ST 1.0 Array ID	0-No	6-No	12-No	0-Yes	6-Yes	12-Yes

Supplemental Table 1. Gene expression values changed greater than 2 fold.

Affymetrix ST 1.0 Array ID	0-No	6-No	12-No	0-Yes	6-Yes	12-Yes

Supplemental Table 1. Gene expression values changed greater than 2 fold.

Affymetrix ST 1.0 Array ID	0-No	6-No	12-No	0-Yes	6-Yes	12-Yes

Supplemental Table 1. Gene expression values changed greater than 2 fold.

Affymetrix ST 1.0 Array ID	0-No	6-No	12-No	0-Yes	6-Yes	12-Yes

Supplemental Table 1. Gene expression values changed greater than 2 fold.

Affymetrix ST 1.0 Array ID	0-No	6-No	12-No	0-Yes	6-Yes	12-Yes

Supplemental Table 1. Gene expression values changed greater than 2 fold.

Affymetrix ST 1.0 Array ID	0-No	6-No	12-No	0-Yes	6-Yes	12-Yes

Supplemental Table 1. Gene expression values changed greater than 2 fold.

Affymetrix ST 1.0 Array ID	0-No	6-No	12-No	0-Yes	6-Yes	12-Yes

Supplemental Table 1. Gene expression values changed greater than 2 fold.

Affymetrix ST 1.0 Array ID	0-No	6-No	12-No	0-Yes	6-Yes	12-Yes

Supplemental Table 1. Gene expression values changed greater than 2 fold.

Affymetrix ST 1.0 Array ID	0-No	6-No	12-No	0-Yes	6-Yes	12-Yes

Supplemental Table 1. Gene expression values changed greater than 2 fold.

Affymetrix ST 1.0 Array ID	0-No	6-No	12-No	0-Yes	6-Yes	12-Yes

Supplemental Table 1. Gene expression values changed greater than 2 fold.

Affymetrix ST 1.0 Array ID	0-No	6-No	12-No	0-Yes	6-Yes	12-Yes

Supplemental Table 1. Gene expression values changed greater than 2 fold.

Affymetrix ST 1.0 Array ID	0-No	6-No	12-No	0-Yes	6-Yes	12-Yes

Supplemental Table 1. Gene expression values changed greater than 2 fold.

Affymetrix ST 1.0 Array ID	0-No	6-No	12-No	0-Yes	6-Yes	12-Yes

Supplemental Table 1. Gene expression values changed greater than 2 fold.

Affymetrix ST 1.0 Array ID	0-No	6-No	12-No	0-Yes	6-Yes	12-Yes

Supplemental Table 1. Gene expression values changed greater than 2 fold.

Affymetrix ST 1.0 Array ID	0-No	6-No	12-No	0-Yes	6-Yes	12-Yes

Supplemental Table 1. Gene expression values changed greater than 2 fold.

Affymetrix ST 1.0 Array ID	0-No	6-No	12-No	0-Yes	6-Yes	12-Yes

Supplemental Table 1. Gene expression values changed greater than 2 fold.

Affymetrix ST 1.0 Array ID	0-No	6-No	12-No	0-Yes	6-Yes	12-Yes

Supplemental Table 1. Gene expression values changed greater than 2 fold.

Affymetrix ST 1.0 Array ID	0-No	6-No	12-No	0-Yes	6-Yes	12-Yes

Supplemental Table 1. Gene expression values changed greater than 2 fold.

Affymetrix ST 1.0 Array ID	0-No	6-No	12-No	0-Yes	6-Yes	12-Yes

Supplemental Table 1. Gene expression values changed greater than 2 fold.

Affymetrix ST 1.0 Array ID	0-No	6-No	12-No	0-Yes	6-Yes	12-Yes

Supplemental Table 1. Gene expression values changed greater than 2 fold.

Affymetrix ST 1.0 Array ID	0-No	6-No	12-No	0-Yes	6-Yes	12-Yes

Supplemental Table 1. Gene expression values changed greater than 2 fold.

Affymetrix ST 1.0 Array ID	0-No	6-No	12-No	0-Yes	6-Yes	12-Yes

Supplemental Table 1. Gene expression values changed greater than 2 fold.

Affymetrix ST 1.0 Array ID	0-No	6-No	12-No	0-Yes	6-Yes	12-Yes

Supplemental Table 1. Gene expression values changed greater than 2 fold.

Affymetrix ST 1.0 Array ID	0-No	6-No	12-No	0-Yes	6-Yes	12-Yes

Supplemental Table 1. Gene expression values changed greater than 2 fold.

Affymetrix ST 1.0 Array ID	0-No	6-No	12-No	0-Yes	6-Yes	12-Yes

Supplemental Table 1. Gene expression values changed greater than 2 fold.

Affymetrix ST 1.0 Array ID	0-No	6-No	12-No	0-Yes	6-Yes	12-Yes

Supplemental Table 1. Gene expression values changed greater than 2 fold.

Affymetrix ST 1.0 Array ID	0-No	6-No	12-No	0-Yes	6-Yes	12-Yes

Supplemental Table 1. Gene expression values changed greater than 2 fold.

Affymetrix ST 1.0 Array ID	0-No	6-No	12-No	0-Yes	6-Yes	12-Yes

Supplemental Table 1. Gene expression values changed greater than 2 fold.

Affymetrix ST 1.0 Array ID	0-No	6-No	12-No	0-Yes	6-Yes	12-Yes

Supplemental Table 1. Gene expression values changed greater than 2 fold.

Affymetrix ST 1.0 Array ID	0-No	6-No	12-No	0-Yes	6-Yes	12-Yes

Supplemental Table 1. Gene expression values changed greater than 2 fold.

Affymetrix ST 1.0 Array ID	0-No	6-No	12-No	0-Yes	6-Yes	12-Yes

Supplemental Table 1. Gene expression values changed greater than 2 fold.

Affymetrix ST 1.0 Array ID	0-No	6-No	12-No	0-Yes	6-Yes	12-Yes

Supplemental Table 1. Gene expression values changed greater than 2 fold.

Affymetrix ST 1.0 Array ID	0-No	6-No	12-No	0-Yes	6-Yes	12-Yes

Supplemental Table 1. Gene expression values changed greater than 2 fold.

Affymetrix ST 1.0 Array ID	0-No	6-No	12-No	0-Yes	6-Yes	12-Yes

Supplemental Table 1. Gene expression values changed greater than 2 fold.

Affymetrix ST 1.0 Array ID	0-No	6-No	12-No	0-Yes	6-Yes	12-Yes

Supplemental Table 1. Gene expression values changed greater than 2 fold.

Affymetrix ST 1.0 Array ID	0-No	6-No	12-No	0-Yes	6-Yes	12-Yes

Supplemental Table 1. Gene expression values changed greater than 2 fold.

Affymetrix ST 1.0 Array ID	0-No	6-No	12-No	0-Yes	6-Yes	12-Yes

Supplemental Table 1. Gene expression values changed greater than 2 fold.

Affymetrix ST 1.0 Array ID	0-No	6-No	12-No	0-Yes	6-Yes	12-Yes

Supplemental Table 1. Gene expression values changed greater than 2 fold.

Affymetrix ST 1.0 Array ID	0-No	6-No	12-No	0-Yes	6-Yes	12-Yes

Supplemental Table 1. Gene expression values changed greater than 2 fold.

Affymetrix ST 1.0 Array ID	0-No	6-No	12-No	0-Yes	6-Yes	12-Yes

Supplemental Table 1. Gene expression values changed greater than 2 fold.

Affymetrix ST 1.0 Array ID	0-No	6-No	12-No	0-Yes	6-Yes	12-Yes

Supplemental Table 1. Gene expression values changed greater than 2 fold.

Affymetrix ST 1.0 Array ID	0-No	6-No	12-No	0-Yes	6-Yes	12-Yes

Supplemental Table 1. Gene expression values changed greater than 2 fold.

Affymetrix ST 1.0 Array ID	0-No	6-No	12-No	0-Yes	6-Yes	12-Yes

Supplemental Table 1. Gene expression values changed greater than 2 fold.

Affymetrix ST 1.0 Array ID	0-No	6-No	12-No	0-Yes	6-Yes	12-Yes

Supplemental Table 1. Gene expression values changed greater than 2 fold.

Affymetrix ST 1.0 Array ID	0-No	6-No	12-No	0-Yes	6-Yes	12-Yes

Supplemental Table 1. Gene expression values changed greater than 2 fold.

Affymetrix ST 1.0 Array ID	0-No	6-No	12-No	0-Yes	6-Yes	12-Yes

Supplemental Table 1. Gene expression values changed greater than 2 fold.

Affymetrix ST 1.0 Array ID	0-No	6-No	12-No	0-Yes	6-Yes	12-Yes

Supplemental Table 1. Gene expression values changed greater than 2 fold.

Affymetrix ST 1.0 Array ID	0-No	6-No	12-No	0-Yes	6-Yes	12-Yes

Supplemental Table 1. Gene expression values changed greater than 2 fold.

Affymetrix ST 1.0 Array ID	0-No	6-No	12-No	0-Yes	6-Yes	12-Yes

Supplemental Table 1. Gene expression values changed greater than 2 fold.

Affymetrix ST 1.0 Array ID	0-No	6-No	12-No	0-Yes	6-Yes	12-Yes

Supplemental Table 1. Gene expression values changed greater than 2 fold.

Affymetrix ST 1.0 Array ID	0-No	6-No	12-No	0-Yes	6-Yes	12-Yes

Supplemental Table 1. Gene expression values changed greater than 2 fold.

Affymetrix ST 1.0 Array ID	0-No	6-No	12-No	0-Yes	6-Yes	12-Yes

Supplemental Table 1. Gene expression values changed greater than 2 fold.

Affymetrix ST 1.0 Array ID	0-No	6-No	12-No	0-Yes	6-Yes	12-Yes

Supplemental Table 1. Gene expression values changed greater than 2 fold.

Affymetrix ST 1.0 Array ID	0-No	6-No	12-No	0-Yes	6-Yes	12-Yes

Supplemental Table 1. Gene expression values changed greater than 2 fold.

Affymetrix ST 1.0 Array ID	0-No	6-No	12-No	0-Yes	6-Yes	12-Yes

Supplemental Table 1. Gene expression values changed greater than 2 fold.

Affymetrix ST 1.0 Array ID	0-No	6-No	12-No	0-Yes	6-Yes	12-Yes

Supplemental Table 1. Gene expression values changed greater than 2 fold.

Affymetrix ST 1.0 Array ID	0-No	6-No	12-No	0-Yes	6-Yes	12-Yes

Supplemental Table 1. Gene expression values changed greater than 2 fold.

Affymetrix ST 1.0 Array ID	0-No	6-No	12-No	0-Yes	6-Yes	12-Yes

Supplemental Table 1. Gene expression values changed greater than 2 fold.

Affymetrix ST 1.0 Array ID	0-No	6-No	12-No	0-Yes	6-Yes	12-Yes

Supplemental Table 1. Gene expression values changed greater than 2 fold.

Affymetrix ST 1.0 Array ID	0-No	6-No	12-No	0-Yes	6-Yes	12-Yes

Supplemental Table 1. Gene expression values changed greater than 2 fold.

Affymetrix ST 1.0 Array ID	0-No	6-No	12-No	0-Yes	6-Yes	12-Yes

Supplemental Table 1. Gene expression values changed greater than 2 fold.

Affymetrix ST 1.0 Array ID	0-No	6-No	12-No	0-Yes	6-Yes	12-Yes

Supplemental Table 1. Gene expression values changed greater than 2 fold.

Affymetrix ST 1.0 Array ID	0-No	6-No	12-No	0-Yes	6-Yes	12-Yes

Supplemental Table 1. Gene expression values changed greater than 2 fold.

Affymetrix ST 1.0 Array ID	0-No	6-No	12-No	0-Yes	6-Yes	12-Yes

Supplemental Table 1. Gene expression values changed greater than 2 fold.

Affymetrix ST 1.0 Array ID	0-No	6-No	12-No	0-Yes	6-Yes	12-Yes

Supplemental Table 1. Gene expression values changed greater than 2 fold.

Affymetrix ST 1.0 Array ID	0-No	6-No	12-No	0-Yes	6-Yes	12-Yes

Supplemental Table 1. Gene expression values changed greater than 2 fold.

Affymetrix ST 1.0 Array ID	0-No	6-No	12-No	0-Yes	6-Yes	12-Yes

Supplemental Table 1. Gene expression values changed greater than 2 fold.

Affymetrix ST 1.0 Array ID	0-No	6-No	12-No	0-Yes	6-Yes	12-Yes

Supplemental Table 1. Gene expression values changed greater than 2 fold.

Affymetrix ST 1.0 Array ID	0-No	6-No	12-No	0-Yes	6-Yes	12-Yes

Supplemental Table 1. Gene expression values changed greater than 2 fold.

Affymetrix ST 1.0 Array ID	0-No	6-No	12-No	0-Yes	6-Yes	12-Yes

Supplemental Table 1. Gene expression values changed greater than 2 fold.

Affymetrix ST 1.0 Array ID	0-No	6-No	12-No	0-Yes	6-Yes	12-Yes

Supplemental Table 1. Gene expression values changed greater than 2 fold.

Affymetrix ST 1.0 Array ID	0-No	6-No	12-No	0-Yes	6-Yes	12-Yes

Supplemental Table 1. Gene expression values changed greater than 2 fold.

Affymetrix ST 1.0 Array ID	0-No	6-No	12-No	0-Yes	6-Yes	12-Yes

Supplemental Table 1. Gene expression values changed greater than 2 fold.

Affymetrix ST 1.0 Array ID	0-No	6-No	12-No	0-Yes	6-Yes	12-Yes

Supplemental Table 1. Gene expression values changed greater than 2 fold.

Affymetrix ST 1.0 Array ID	0-No	6-No	12-No	0-Yes	6-Yes	12-Yes

Supplemental Table 1. Gene expression values changed greater than 2 fold.

Affymetrix ST 1.0 Array ID	0-No	6-No	12-No	0-Yes	6-Yes	12-Yes

Supplemental Table 1. Gene expression values changed greater than 2 fold.

Affymetrix ST 1.0 Array ID	0-No	6-No	12-No	0-Yes	6-Yes	12-Yes

Supplemental Table 1. Gene expression values changed greater than 2 fold.

Affymetrix ST 1.0 Array ID	0-No	6-No	12-No	0-Yes	6-Yes	12-Yes

Supplemental Table 1. Gene expression values changed greater than 2 fold.

Affymetrix ST 1.0 Array ID	0-No	6-No	12-No	0-Yes	6-Yes	12-Yes

Supplemental Table 1. Gene expression values changed greater than 2 fold.

Affymetrix ST 1.0 Array ID	0-No	6-No	12-No	0-Yes	6-Yes	12-Yes

Supplemental Table 1. Gene expression values changed greater than 2 fold.

Affymetrix ST 1.0 Array ID	0-No	6-No	12-No	0-Yes	6-Yes	12-Yes

Supplemental Table 1. Gene expression values changed greater than 2 fold.

Affymetrix ST 1.0 Array ID	0-No	6-No	12-No	0-Yes	6-Yes	12-Yes

Supplemental Table 1. Gene expression values changed greater than 2 fold.

Affymetrix ST 1.0 Array ID	0-No	6-No	12-No	0-Yes	6-Yes	12-Yes

Supplemental Table 1. Gene expression values changed greater than 2 fold.

Affymetrix ST 1.0 Array ID	0-No	6-No	12-No	0-Yes	6-Yes	12-Yes

Supplemental Table 1. Gene expression values changed greater than 2 fold.

Affymetrix ST 1.0 Array ID	0-No	6-No	12-No	0-Yes	6-Yes	12-Yes

Supplemental Table 1. Gene expression values changed greater than 2 fold.

Affymetrix ST 1.0 Array ID	0-No	6-No	12-No	0-Yes	6-Yes	12-Yes

Supplemental Table 1. Gene expression values changed greater than 2 fold.

Affymetrix ST 1.0 Array ID	0-No	6-No	12-No	0-Yes	6-Yes	12-Yes

Supplemental Table 1. Gene expression values changed greater than 2 fold.

Affymetrix ST 1.0 Array ID	0-No	6-No	12-No	0-Yes	6-Yes	12-Yes

Supplemental Table 1. Gene expression values changed greater than 2 fold.

Affymetrix ST 1.0 Array ID	0-No	6-No	12-No	0-Yes	6-Yes	12-Yes

Supplemental Table 1. Gene expression values changed greater than 2 fold.

Affymetrix ST 1.0 Array ID	0-No	6-No	12-No	0-Yes	6-Yes	12-Yes

Supplemental Table 1. Gene expression values changed greater than 2 fold.

Affymetrix ST 1.0 Array ID	0-No	6-No	12-No	0-Yes	6-Yes	12-Yes

Supplemental Table 1. Gene expression values changed greater than 2 fold.

Affymetrix ST 1.0 Array ID	0-No	6-No	12-No	0-Yes	6-Yes	12-Yes

Supplemental Table 1. Gene expression values changed greater than 2 fold.

Affymetrix ST 1.0 Array ID	0-No	6-No	12-No	0-Yes	6-Yes	12-Yes

Supplemental Table 1. Gene expression values changed greater than 2 fold.

Affymetrix ST 1.0 Array ID	0-No	6-No	12-No	0-Yes	6-Yes	12-Yes

Supplemental Table 1. Gene expression values changed greater than 2 fold.

Affymetrix ST 1.0 Array ID	0-No	6-No	12-No	0-Yes	6-Yes	12-Yes

Supplemental Table 1. Gene expression values changed greater than 2 fold.

Affymetrix ST 1.0 Array ID	0-No	6-No	12-No	0-Yes	6-Yes	12-Yes

Supplemental Table 1. Gene expression values changed greater than 2 fold.

Affymetrix ST 1.0 Array ID	0-No	6-No	12-No	0-Yes	6-Yes	12-Yes

Supplemental Table 1. Gene expression values changed greater than 2 fold.

Affymetrix ST 1.0 Array ID	0-No	6-No	12-No	0-Yes	6-Yes	12-Yes

Supplemental Table 1. Gene expression values changed greater than 2 fold.

Affymetrix ST 1.0 Array ID	0-No	6-No	12-No	0-Yes	6-Yes	12-Yes

Supplemental Table 1. Gene expression values changed greater than 2 fold.

Affymetrix ST 1.0 Array ID	0-No	6-No	12-No	0-Yes	6-Yes	12-Yes

Supplemental Table 1. Gene expression values changed greater than 2 fold.

Affymetrix ST 1.0 Array ID	0-No	6-No	12-No	0-Yes	6-Yes	12-Yes

Supplemental Table 1. Gene expression values changed greater than 2 fold.

Affymetrix ST 1.0 Array ID	0-No	6-No	12-No	0-Yes	6-Yes	12-Yes

Supplemental Table 1. Gene expression values changed greater than 2 fold.

Affymetrix ST 1.0 Array ID	0-No	6-No	12-No	0-Yes	6-Yes	12-Yes

Supplemental Table 1. Gene expression values changed greater than 2 fold.

Affymetrix ST 1.0 Array ID	0-No	6-No	12-No	0-Yes	6-Yes	12-Yes

Supplemental Table 1. Gene expression values changed greater than 2 fold.

Affymetrix ST 1.0 Array ID	0-No	6-No	12-No	0-Yes	6-Yes	12-Yes

Supplemental Table 1. Gene expression values changed greater than 2 fold.

Affymetrix ST 1.0 Array ID	0-No	6-No	12-No	0-Yes	6-Yes	12-Yes

Supplemental Table 1. Gene expression values changed greater than 2 fold.

Affymetrix ST 1.0 Array ID	0-No	6-No	12-No	0-Yes	6-Yes	12-Yes

Supplemental Table 1. Gene expression values changed greater than 2 fold.

Affymetrix ST 1.0 Array ID	0-No	6-No	12-No	0-Yes	6-Yes	12-Yes

Supplemental Table 1. Gene expression values changed greater than 2 fold.

Affymetrix ST 1.0 Array ID	0-No	6-No	12-No	0-Yes	6-Yes	12-Yes

Supplemental Table 1. Gene expression values changed greater than 2 fold.

Affymetrix ST 1.0 Array ID	0-No	6-No	12-No	0-Yes	6-Yes	12-Yes

Supplemental Table 1. Gene expression values changed greater than 2 fold.

Affymetrix ST 1.0 Array ID	0-No	6-No	12-No	0-Yes	6-Yes	12-Yes

Supplemental Table 1. Gene expression values changed greater than 2 fold.

Affymetrix ST 1.0 Array ID	0-No	6-No	12-No	0-Yes	6-Yes	12-Yes

Supplemental Table 1. Gene expression values changed greater than 2 fold.

Affymetrix ST 1.0 Array ID	0-No	6-No	12-No	0-Yes	6-Yes	12-Yes

Supplemental Table 1. Gene expression values changed greater than 2 fold.

Affymetrix ST 1.0 Array ID	0-No	6-No	12-No	0-Yes	6-Yes	12-Yes

Supplemental Table 1. Gene expression values changed greater than 2 fold.

Affymetrix ST 1.0 Array ID	0-No	6-No	12-No	0-Yes	6-Yes	12-Yes

Supplemental Table 1. Gene expression values changed greater than 2 fold.

Affymetrix ST 1.0 Array ID	0-No	6-No	12-No	0-Yes	6-Yes	12-Yes

Supplemental Table 1. Gene expression values changed greater than 2 fold.

Affymetrix ST 1.0 Array ID	0-No	6-No	12-No	0-Yes	6-Yes	12-Yes

Supplemental Table 1. Gene expression values changed greater than 2 fold.

Affymetrix ST 1.0 Array ID	0-No	6-No	12-No	0-Yes	6-Yes	12-Yes

Supplemental Table 1. Gene expression values changed greater than 2 fold.

Affymetrix ST 1.0 Array ID	0-No	6-No	12-No	0-Yes	6-Yes	12-Yes

Supplemental Table 1. Gene expression values changed greater than 2 fold.

Affymetrix ST 1.0 Array ID	0-No	6-No	12-No	0-Yes	6-Yes	12-Yes

Supplemental Table 1. Gene expression values changed greater than 2 fold.

Affymetrix ST 1.0 Array ID	0-No	6-No	12-No	0-Yes	6-Yes	12-Yes

Supplemental Table 1. Gene expression values changed greater than 2 fold.

Affymetrix ST 1.0 Array ID	0-No	6-No	12-No	0-Yes	6-Yes	12-Yes

Supplemental Table 1. Gene expression values changed greater than 2 fold.

Affymetrix ST 1.0 Array ID	0-No	6-No	12-No	0-Yes	6-Yes	12-Yes

Supplemental Table 1. Gene expression values changed greater than 2 fold.

Affymetrix ST 1.0 Array ID	0-No	6-No	12-No	0-Yes	6-Yes	12-Yes

Supplemental Table 1. Gene expression values changed greater than 2 fold.

Affymetrix ST 1.0 Array ID	0-No	6-No	12-No	0-Yes	6-Yes	12-Yes

Supplemental Table 1. Gene expression values changed greater than 2 fold.

Affymetrix ST 1.0 Array ID	0-No	6-No	12-No	0-Yes	6-Yes	12-Yes

Supplemental Table 1. Gene expression values changed greater than 2 fold.

Affymetrix ST 1.0 Array ID	0-No	6-No	12-No	0-Yes	6-Yes	12-Yes

Supplemental Table 1. Gene expression values changed greater than 2 fold.

Affymetrix ST 1.0 Array ID	0-No	6-No	12-No	0-Yes	6-Yes	12-Yes

Supplemental Table 1. Gene expression values changed greater than 2 fold.

Affymetrix ST 1.0 Array ID	0-No	6-No	12-No	0-Yes	6-Yes	12-Yes

Supplemental Table 1. Gene expression values changed greater than 2 fold.

Affymetrix ST 1.0 Array ID	0-No	6-No	12-No	0-Yes	6-Yes	12-Yes

Supplemental Table 1. Gene expression values changed greater than 2 fold.

Affymetrix ST 1.0 Array ID	0-No	6-No	12-No	0-Yes	6-Yes	12-Yes

Supplemental Table 1. Gene expression values changed greater than 2 fold.

Affymetrix ST 1.0 Array ID	0-No	6-No	12-No	0-Yes	6-Yes	12-Yes

Supplemental Table 1. Gene expression values changed greater than 2 fold.

Affymetrix ST 1.0 Array ID	0-No	6-No	12-No	0-Yes	6-Yes	12-Yes

Supplemental Table 1. Gene expression values changed greater than 2 fold.

Affymetrix ST 1.0 Array ID	0-No	6-No	12-No	0-Yes	6-Yes	12-Yes

Supplemental Table 1. Gene expression values changed greater than 2 fold.

Affymetrix ST 1.0 Array ID	0-No	6-No	12-No	0-Yes	6-Yes	12-Yes

Supplemental Table 1. Gene expression values changed greater than 2 fold.

Affymetrix ST 1.0 Array ID	0-No	6-No	12-No	0-Yes	6-Yes	12-Yes

Supplemental Table 1. Gene expression values changed greater than 2 fold.

Affymetrix ST 1.0 Array ID	0-No	6-No	12-No	0-Yes	6-Yes	12-Yes

Supplemental Table 1. Gene expression values changed greater than 2 fold.

Affymetrix ST 1.0 Array ID	0-No	6-No	12-No	0-Yes	6-Yes	12-Yes

Supplemental Table 1. Gene expression values changed greater than 2 fold.

Affymetrix ST 1.0 Array ID	0-No	6-No	12-No	0-Yes	6-Yes	12-Yes

Supplemental Table 1. Gene expression values changed greater than 2 fold.

Affymetrix ST 1.0 Array ID	0-No	6-No	12-No	0-Yes	6-Yes	12-Yes

Supplemental Table 1. Gene expression values changed greater than 2 fold.

Affymetrix ST 1.0 Array ID	0-No	6-No	12-No	0-Yes	6-Yes	12-Yes

Supplemental Table 1. Gene expression values changed greater than 2 fold.

Affymetrix ST 1.0 Array ID	0-No	6-No	12-No	0-Yes	6-Yes	12-Yes

Supplemental Table 1. Gene expression values changed greater than 2 fold.

Affymetrix ST 1.0 Array ID	0-No	6-No	12-No	0-Yes	6-Yes	12-Yes

Supplemental Table 1. Gene expression values changed greater than 2 fold.

Affymetrix ST 1.0 Array ID	0-No	6-No	12-No	0-Yes	6-Yes	12-Yes

Supplemental Table 1. Gene expression values changed greater than 2 fold.

Affymetrix ST 1.0 Array ID	0-No	6-No	12-No	0-Yes	6-Yes	12-Yes

Supplemental Table 1. Gene expression values changed greater than 2 fold.

Affymetrix ST 1.0 Array ID	0-No	6-No	12-No	0-Yes	6-Yes	12-Yes

Supplemental Table 1. Gene expression values changed greater than 2 fold.

Affymetrix ST 1.0 Array ID	0-No	6-No	12-No	0-Yes	6-Yes	12-Yes

Supplemental Table 1. Gene expression values changed greater than 2 fold.

Affymetrix ST 1.0 Array ID	0-No	6-No	12-No	0-Yes	6-Yes	12-Yes

Supplemental Table 1. Gene expression values changed greater than 2 fold.

Affymetrix ST 1.0 Array ID	0-No	6-No	12-No	0-Yes	6-Yes	12-Yes

Supplemental Table 1. Gene expression values changed greater than 2 fold.

Affymetrix ST 1.0 Array ID	0-No	6-No	12-No	0-Yes	6-Yes	12-Yes

Supplemental Table 1. Gene expression values changed greater than 2 fold.

Affymetrix ST 1.0 Array ID	0-No	6-No	12-No	0-Yes	6-Yes	12-Yes

Supplemental Table 1. Gene expression values changed greater than 2 fold.

Affymetrix ST 1.0 Array ID	0-No	6-No	12-No	0-Yes	6-Yes	12-Yes

Supplemental Table 1. Gene expression values changed greater than 2 fold.

Affymetrix ST 1.0 Array ID	0-No	6-No	12-No	0-Yes	6-Yes	12-Yes

Supplemental Table 1. Gene expression values changed greater than 2 fold.

Affymetrix ST 1.0 Array ID	0-No	6-No	12-No	0-Yes	6-Yes	12-Yes

Supplemental Table 1. Gene expression values changed greater than 2 fold.

Affymetrix ST 1.0 Array ID	0-No	6-No	12-No	0-Yes	6-Yes	12-Yes

Supplemental Table 1. Gene expression values changed greater than 2 fold.

Affymetrix ST 1.0 Array ID	0-No	6-No	12-No	0-Yes	6-Yes	12-Yes

Supplemental Table 1. Gene expression values changed greater than 2 fold.

Affymetrix ST 1.0 Array ID	0-No	6-No	12-No	0-Yes	6-Yes	12-Yes

Supplemental Table 1. Gene expression values changed greater than 2 fold.

Affymetrix ST 1.0 Array ID	0-No	6-No	12-No	0-Yes	6-Yes	12-Yes

Supplemental Table 1. Gene expression values changed greater than 2 fold.

Affymetrix ST 1.0 Array ID	0-No	6-No	12-No	0-Yes	6-Yes	12-Yes

Supplemental Table 1. Gene expression values changed greater than 2 fold.

Affymetrix ST 1.0 Array ID	0-No	6-No	12-No	0-Yes	6-Yes	12-Yes

Supplemental Table 1. Gene expression values changed greater than 2 fold.

Affymetrix ST 1.0 Array ID	0-No	6-No	12-No	0-Yes	6-Yes	12-Yes

Supplemental Table 1. Gene expression values changed greater than 2 fold.

Affymetrix ST 1.0 Array ID	0-No	6-No	12-No	0-Yes	6-Yes	12-Yes

Supplemental Table 1. Gene expression values changed greater than 2 fold.

Affymetrix ST 1.0 Array ID	0-No	6-No	12-No	0-Yes	6-Yes	12-Yes

Supplemental Table 1. Gene expression values changed greater than 2 fold.

Affymetrix ST 1.0 Array ID	0-No	6-No	12-No	0-Yes	6-Yes	12-Yes

Supplemental Table 1. Gene expression values changed greater than 2 fold.

Affymetrix ST 1.0 Array ID	0-No	6-No	12-No	0-Yes	6-Yes	12-Yes

Supplemental Table 1. Gene expression values changed greater than 2 fold.

Affymetrix ST 1.0 Array ID	0-No	6-No	12-No	0-Yes	6-Yes	12-Yes

Supplemental Table 1. Gene expression values changed greater than 2 fold.

Affymetrix ST 1.0 Array ID	0-No	6-No	12-No	0-Yes	6-Yes	12-Yes

Supplemental Table 1. Gene expression values changed greater than 2 fold.

Affymetrix ST 1.0 Array ID	0-No	6-No	12-No	0-Yes	6-Yes	12-Yes

Supplemental Table 1. Gene expression values changed greater than 2 fold.

Affymetrix ST 1.0 Array ID	0-No	6-No	12-No	0-Yes	6-Yes	12-Yes

Supplemental Table 1. Gene expression values changed greater than 2 fold.

Affymetrix ST 1.0 Array ID	0-No	6-No	12-No	0-Yes	6-Yes	12-Yes

Supplemental Table 1. Gene expression values changed greater than 2 fold.

Affymetrix ST 1.0 Array ID	0-No	6-No	12-No	0-Yes	6-Yes	12-Yes

Supplemental Table 1. Gene expression values changed greater than 2 fold.

Affymetrix ST 1.0 Array ID	0-No	6-No	12-No	0-Yes	6-Yes	12-Yes

Supplemental Table 1. Gene expression values changed greater than 2 fold.

Affymetrix ST 1.0 Array ID	0-No	6-No	12-No	0-Yes	6-Yes	12-Yes

Supplemental Table 1. Gene expression values changed greater than 2 fold.

Affymetrix ST 1.0 Array ID	0-No	6-No	12-No	0-Yes	6-Yes	12-Yes

Supplemental Table 1. Gene expression values changed greater than 2 fold.

Affymetrix ST 1.0 Array ID	0-No	6-No	12-No	0-Yes	6-Yes	12-Yes

Supplemental Table 1. Gene expression values changed greater than 2 fold.

Affymetrix ST 1.0 Array ID	0-No	6-No	12-No	0-Yes	6-Yes	12-Yes

Supplemental Table 1. Gene expression values changed greater than 2 fold.

Affymetrix ST 1.0 Array ID	0-No	6-No	12-No	0-Yes	6-Yes	12-Yes

Supplemental Table 1. Gene expression values changed greater than 2 fold.

Affymetrix ST 1.0 Array ID	0-No	6-No	12-No	0-Yes	6-Yes	12-Yes

Supplemental Table 1. Gene expression values changed greater than 2 fold.

Affymetrix ST 1.0 Array ID	0-No	6-No	12-No	0-Yes	6-Yes	12-Yes

Supplemental Table 1. Gene expression values changed greater than 2 fold.

Affymetrix ST 1.0 Array ID	0-No	6-No	12-No	0-Yes	6-Yes	12-Yes

Supplemental Table 1. Gene expression values changed greater than 2 fold.

Affymetrix ST 1.0 Array ID	0-No	6-No	12-No	0-Yes	6-Yes	12-Yes

Supplemental Table 1. Gene expression values changed greater than 2 fold.

Affymetrix ST 1.0 Array ID	0-No	6-No	12-No	0-Yes	6-Yes	12-Yes

Supplemental Table 1. Gene expression values changed greater than 2 fold.

Affymetrix ST 1.0 Array ID	0-No	6-No	12-No	0-Yes	6-Yes	12-Yes

Supplemental Table 1. Gene expression values changed greater than 2 fold.

Affymetrix ST 1.0 Array ID	0-No	6-No	12-No	0-Yes	6-Yes	12-Yes

Supplemental Table 1. Gene expression values changed greater than 2 fold.

Affymetrix ST 1.0 Array ID	0-No	6-No	12-No	0-Yes	6-Yes	12-Yes

Supplemental Table 1. Gene expression values changed greater than 2 fold.

Affymetrix ST 1.0 Array ID	0-No	6-No	12-No	0-Yes	6-Yes	12-Yes

Supplemental Table 1. Gene expression values changed greater than 2 fold.

Affymetrix ST 1.0 Array ID	0-No	6-No	12-No	0-Yes	6-Yes	12-Yes

Supplemental Table 1. Gene expression values changed greater than 2 fold.

Affymetrix ST 1.0 Array ID	0-No	6-No	12-No	0-Yes	6-Yes	12-Yes

Supplemental Table 1. Gene expression values changed greater than 2 fold.

Affymetrix ST 1.0 Array ID	0-No	6-No	12-No	0-Yes	6-Yes	12-Yes

Supplemental Table 1. Gene expression values changed greater than 2 fold.

Affymetrix ST 1.0 Array ID	0-No	6-No	12-No	0-Yes	6-Yes	12-Yes

Supplemental Table 1. Gene expression values changed greater than 2 fold.

Affymetrix ST 1.0 Array ID	0-No	6-No	12-No	0-Yes	6-Yes	12-Yes

Supplemental Table 1. Gene expression values changed greater than 2 fold.

Affymetrix ST 1.0 Array ID	0-No	6-No	12-No	0-Yes	6-Yes	12-Yes

Supplemental Table 1. Gene expression values changed greater than 2 fold.

Affymetrix ST 1.0 Array ID	0-No	6-No	12-No	0-Yes	6-Yes	12-Yes

Supplemental Table 1. Gene expression values changed greater than 2 fold.

Affymetrix ST 1.0 Array ID	0-No	6-No	12-No	0-Yes	6-Yes	12-Yes

Supplemental Table 1. Gene expression values changed greater than 2 fold.

Affymetrix ST 1.0 Array ID	0-No	6-No	12-No	0-Yes	6-Yes	12-Yes

Supplemental Table 1. Gene expression values changed greater than 2 fold.

Affymetrix ST 1.0 Array ID	0-No	6-No	12-No	0-Yes	6-Yes	12-Yes

Supplemental Table 1. Gene expression values changed greater than 2 fold.

Affymetrix ST 1.0 Array ID	0-No	6-No	12-No	0-Yes	6-Yes	12-Yes

Supplemental Table 1. Gene expression values changed greater than 2 fold.

Affymetrix ST 1.0 Array ID	0-No	6-No	12-No	0-Yes	6-Yes	12-Yes

Supplemental Table 1. Gene expression values changed greater than 2 fold.

Affymetrix ST 1.0 Array ID	0-No	6-No	12-No	0-Yes	6-Yes	12-Yes

Supplemental Table 1. Gene expression values changed greater than 2 fold.

Affymetrix ST 1.0 Array ID	0-No	6-No	12-No	0-Yes	6-Yes	12-Yes

Supplemental Table 1. Gene expression values changed greater than 2 fold.

Affymetrix ST 1.0 Array ID	0-No	6-No	12-No	0-Yes	6-Yes	12-Yes

Supplemental Table 1. Gene expression values changed greater than 2 fold.

Affymetrix ST 1.0 Array ID	0-No	6-No	12-No	0-Yes	6-Yes	12-Yes

Supplemental Table 1. Gene expression values changed greater than 2 fold.

Affymetrix ST 1.0 Array ID	0-No	6-No	12-No	0-Yes	6-Yes	12-Yes

Supplemental Table 1. Gene expression values changed greater than 2 fold.

Affymetrix ST 1.0 Array ID	0-No	6-No	12-No	0-Yes	6-Yes	12-Yes

Supplemental Table 1. Gene expression values changed greater than 2 fold.

Affymetrix ST 1.0 Array ID	0-No	6-No	12-No	0-Yes	6-Yes	12-Yes

Supplemental Table 1. Gene expression values changed greater than 2 fold.

Affymetrix ST 1.0 Array ID	0-No	6-No	12-No	0-Yes	6-Yes	12-Yes

Supplemental Table 1. Gene expression values changed greater than 2 fold.

Affymetrix ST 1.0 Array ID	0-No	6-No	12-No	0-Yes	6-Yes	12-Yes

Supplemental Table 1. Gene expression values changed greater than 2 fold.

Affymetrix ST 1.0 Array ID	0-No	6-No	12-No	0-Yes	6-Yes	12-Yes

Supplemental Table 1. Gene expression values changed greater than 2 fold.

Affymetrix ST 1.0 Array ID	0-No	6-No	12-No	0-Yes	6-Yes	12-Yes

Supplemental Table 1. Gene expression values changed greater than 2 fold.

Affymetrix ST 1.0 Array ID	0-No	6-No	12-No	0-Yes	6-Yes	12-Yes

Supplemental Table 1. Gene expression values changed greater than 2 fold.

Affymetrix ST 1.0 Array ID	0-No	6-No	12-No	0-Yes	6-Yes	12-Yes

Supplemental Table 1. Gene expression values changed greater than 2 fold.

Affymetrix ST 1.0 Array ID	0-No	6-No	12-No	0-Yes	6-Yes	12-Yes

Supplemental Table 1. Gene expression values changed greater than 2 fold.

Affymetrix ST 1.0 Array ID	0-No	6-No	12-No	0-Yes	6-Yes	12-Yes

Supplemental Table 1. Gene expression values changed greater than 2 fold.

Affymetrix ST 1.0 Array ID	0-No	6-No	12-No	0-Yes	6-Yes	12-Yes

Supplemental Table 1. Gene expression values changed greater than 2 fold.

Affymetrix ST 1.0 Array ID	0-No	6-No	12-No	0-Yes	6-Yes	12-Yes

Supplemental Table 1. Gene expression values changed greater than 2 fold.

Affymetrix ST 1.0 Array ID	0-No	6-No	12-No	0-Yes	6-Yes	12-Yes

Supplemental Table 1. Gene expression values changed greater than 2 fold.

Affymetrix ST 1.0 Array ID	0-No	6-No	12-No	0-Yes	6-Yes	12-Yes

Supplemental Table 1. Gene expression values changed greater than 2 fold.

Affymetrix ST 1.0 Array ID	0-No	6-No	12-No	0-Yes	6-Yes	12-Yes

Supplemental Table 1. Gene expression values changed greater than 2 fold.

Affymetrix ST 1.0 Array ID	0-No	6-No	12-No	0-Yes	6-Yes	12-Yes

Supplemental Table 1. Gene expression values changed greater than 2 fold.

Affymetrix ST 1.0 Array ID	0-No	6-No	12-No	0-Yes	6-Yes	12-Yes

Supplemental Table 1. Gene expression values changed greater than 2 fold.

Affymetrix ST 1.0 Array ID	0-No	6-No	12-No	0-Yes	6-Yes	12-Yes

Supplemental Table 1. Gene expression values changed greater than 2 fold.

Affymetrix ST 1.0 Array ID	0-No	6-No	12-No	0-Yes	6-Yes	12-Yes

Supplemental Table 1. Gene expression values changed greater than 2 fold.

Affymetrix ST 1.0 Array ID	0-No	6-No	12-No	0-Yes	6-Yes	12-Yes

Supplemental Table 1. Gene expression values changed greater than 2 fold.

Affymetrix ST 1.0 Array ID	0-No	6-No	12-No	0-Yes	6-Yes	12-Yes

Supplemental Table 1. Gene expression values changed greater than 2 fold.

Affymetrix ST 1.0 Array ID	0-No	6-No	12-No	0-Yes	6-Yes	12-Yes

Supplemental Table 1. Gene expression values changed greater than 2 fold.

Affymetrix ST 1.0 Array ID	0-No	6-No	12-No	0-Yes	6-Yes	12-Yes

Supplemental Table 1. Gene expression values changed greater than 2 fold.

Affymetrix ST 1.0 Array ID	0-No	6-No	12-No	0-Yes	6-Yes	12-Yes

Supplemental Table 1. Gene expression values changed greater than 2 fold.

Affymetrix ST 1.0 Array ID	0-No	6-No	12-No	0-Yes	6-Yes	12-Yes

Supplemental Table 1. Gene expression values changed greater than 2 fold.

Affymetrix ST 1.0 Array ID	0-No	6-No	12-No	0-Yes	6-Yes	12-Yes

Supplemental Table 1. Gene expression values changed greater than 2 fold.

Affymetrix ST 1.0 Array ID	0-No	6-No	12-No	0-Yes	6-Yes	12-Yes

Supplemental Table 1. Gene expression values changed greater than 2 fold.

Affymetrix ST 1.0 Array ID	0-No	6-No	12-No	0-Yes	6-Yes	12-Yes

Supplemental Table 1. Gene expression values changed greater than 2 fold.

Affymetrix ST 1.0 Array ID	0-No	6-No	12-No	0-Yes	6-Yes	12-Yes

Supplemental Table 1. Gene expression values changed greater than 2 fold.

Affymetrix ST 1.0 Array ID	0-No	6-No	12-No	0-Yes	6-Yes	12-Yes

Supplemental Table 1. Gene expression values changed greater than 2 fold.

Affymetrix ST 1.0 Array ID	0-No	6-No	12-No	0-Yes	6-Yes	12-Yes

Supplemental Table 1. Gene expression values changed greater than 2 fold.

Affymetrix ST 1.0 Array ID	0-No	6-No	12-No	0-Yes	6-Yes	12-Yes

Supplemental Table 1. Gene expression values changed greater than 2 fold.

Affymetrix ST 1.0 Array ID	0-No	6-No	12-No	0-Yes	6-Yes	12-Yes

Supplemental Table 1. Gene expression values changed greater than 2 fold.

Affymetrix ST 1.0 Array ID	0-No	6-No	12-No	0-Yes	6-Yes	12-Yes

Supplemental Table 1. Gene expression values changed greater than 2 fold.

Affymetrix ST 1.0 Array ID	0-No	6-No	12-No	0-Yes	6-Yes	12-Yes

Supplemental Table 1. Gene expression values changed greater than 2 fold.

Affymetrix ST 1.0 Array ID	0-No	6-No	12-No	0-Yes	6-Yes	12-Yes

Supplemental Table 1. Gene expression values changed greater than 2 fold.

Affymetrix ST 1.0 Array ID	0-No	6-No	12-No	0-Yes	6-Yes	12-Yes

Supplemental Table 1. Gene expression values changed greater than 2 fold.

Affymetrix ST 1.0 Array ID	0-No	6-No	12-No	0-Yes	6-Yes	12-Yes

Supplemental Table 1. Gene expression values changed greater than 2 fold.

Affymetrix ST 1.0 Array ID	0-No	6-No	12-No	0-Yes	6-Yes	12-Yes

Supplemental Table 1. Gene expression values changed greater than 2 fold.

Affymetrix ST 1.0 Array ID	0-No	6-No	12-No	0-Yes	6-Yes	12-Yes

Supplemental Table 1. Gene expression values changed greater than 2 fold.

Affymetrix ST 1.0 Array ID	0-No	6-No	12-No	0-Yes	6-Yes	12-Yes

Supplemental Table 1. Gene expression values changed greater than 2 fold.

Affymetrix ST 1.0 Array ID	0-No	6-No	12-No	0-Yes	6-Yes	12-Yes

Supplemental Table 1. Gene expression values changed greater than 2 fold.

Affymetrix ST 1.0 Array ID	0-No	6-No	12-No	0-Yes	6-Yes	12-Yes

Supplemental Table 1. Gene expression values changed greater than 2 fold.

Affymetrix ST 1.0 Array ID	0-No	6-No	12-No	0-Yes	6-Yes	12-Yes

Supplemental Table 1. Gene expression values changed greater than 2 fold.

Affymetrix ST 1.0 Array ID	0-No	6-No	12-No	0-Yes	6-Yes	12-Yes

Supplemental Table 1. Gene expression values changed greater than 2 fold.

Affymetrix ST 1.0 Array ID	0-No	6-No	12-No	0-Yes	6-Yes	12-Yes

Supplemental Table 1. Gene expression values changed greater than 2 fold.

Affymetrix ST 1.0 Array ID	0-No	6-No	12-No	0-Yes	6-Yes	12-Yes

Supplemental Table 1. Gene expression values changed greater than 2 fold.

Affymetrix ST 1.0 Array ID	0-No	6-No	12-No	0-Yes	6-Yes	12-Yes

Supplemental Table 1. Gene expression values changed greater than 2 fold.

Affymetrix ST 1.0 Array ID	0-No	6-No	12-No	0-Yes	6-Yes	12-Yes

Supplemental Table 1. Gene expression values changed greater than 2 fold.

Affymetrix ST 1.0 Array ID	0-No	6-No	12-No	0-Yes	6-Yes	12-Yes

Supplemental Table 1. Gene expression values changed greater than 2 fold.

Affymetrix ST 1.0 Array ID	0-No	6-No	12-No	0-Yes	6-Yes	12-Yes

Supplemental Table 1. Gene expression values changed greater than 2 fold.

Affymetrix ST 1.0 Array ID	0-No	6-No	12-No	0-Yes	6-Yes	12-Yes

Supplemental Table 1. Gene expression values changed greater than 2 fold.

Affymetrix ST 1.0 Array ID	0-No	6-No	12-No	0-Yes	6-Yes	12-Yes

Supplemental Table 1. Gene expression values changed greater than 2 fold.

Affymetrix ST 1.0 Array ID	0-No	6-No	12-No	0-Yes	6-Yes	12-Yes

Supplemental Table 1. Gene expression values changed greater than 2 fold.

Affymetrix ST 1.0 Array ID	0-No	6-No	12-No	0-Yes	6-Yes	12-Yes

Supplemental Table 1. Gene expression values changed greater than 2 fold.

Affymetrix ST 1.0 Array ID	0-No	6-No	12-No	0-Yes	6-Yes	12-Yes

Supplemental Table 1. Gene expression values changed greater than 2 fold.

Affymetrix ST 1.0 Array ID	0-No	6-No	12-No	0-Yes	6-Yes	12-Yes

Supplemental Table 1. Gene expression values changed greater than 2 fold.

Affymetrix ST 1.0 Array ID	0-No	6-No	12-No	0-Yes	6-Yes	12-Yes

Supplemental Table 1. Gene expression values changed greater than 2 fold.

Affymetrix ST 1.0 Array ID	0-No	6-No	12-No	0-Yes	6-Yes	12-Yes

Supplemental Table 1. Gene expression values changed greater than 2 fold.

Affymetrix ST 1.0 Array ID	0-No	6-No	12-No	0-Yes	6-Yes	12-Yes

Supplemental Table 1. Gene expression values changed greater than 2 fold.

Affymetrix ST 1.0 Array ID	0-No	6-No	12-No	0-Yes	6-Yes	12-Yes

Supplemental Table 1. Gene expression values changed greater than 2 fold.

Affymetrix ST 1.0 Array ID	0-No	6-No	12-No	0-Yes	6-Yes	12-Yes

Supplemental Table 1. Gene expression values changed greater than 2 fold.

Affymetrix ST 1.0 Array ID	0-No	6-No	12-No	0-Yes	6-Yes	12-Yes

Supplemental Table 1. Gene expression values changed greater than 2 fold.

Affymetrix ST 1.0 Array ID	0-No	6-No	12-No	0-Yes	6-Yes	12-Yes

Supplemental Table 1. Gene expression values changed greater than 2 fold.

Affymetrix ST 1.0 Array ID	0-No	6-No	12-No	0-Yes	6-Yes	12-Yes

Supplemental Table 1. Gene expression values changed greater than 2 fold.

Affymetrix ST 1.0 Array ID	0-No	6-No	12-No	0-Yes	6-Yes	12-Yes

Supplemental Table 1. Gene expression values changed greater than 2 fold.

Affymetrix ST 1.0 Array ID	0-No	6-No	12-No	0-Yes	6-Yes	12-Yes

Supplemental Table 1. Gene expression values changed greater than 2 fold.

Affymetrix ST 1.0 Array ID	0-No	6-No	12-No	0-Yes	6-Yes	12-Yes

Supplemental Table 1. Gene expression values changed greater than 2 fold.

Affymetrix ST 1.0 Array ID	0-No	6-No	12-No	0-Yes	6-Yes	12-Yes

Supplemental Table 1. Gene expression values changed greater than 2 fold.

Affymetrix ST 1.0 Array ID	0-No	6-No	12-No	0-Yes	6-Yes	12-Yes

Supplemental Table 1. Gene expression values changed greater than 2 fold.

Affymetrix ST 1.0 Array ID	0-No	6-No	12-No	0-Yes	6-Yes	12-Yes

Supplemental Table 1. Gene expression values changed greater than 2 fold.

Affymetrix ST 1.0 Array ID	0-No	6-No	12-No	0-Yes	6-Yes	12-Yes

Supplemental Table 1. Gene expression values changed greater than 2 fold.

Affymetrix ST 1.0 Array ID	0-No	6-No	12-No	0-Yes	6-Yes	12-Yes

Supplemental Table 1. Gene expression values changed greater than 2 fold.

Affymetrix ST 1.0 Array ID	0-No	6-No	12-No	0-Yes	6-Yes	12-Yes

Supplemental Table 1. Gene expression values changed greater than 2 fold.

Affymetrix ST 1.0 Array ID	0-No	6-No	12-No	0-Yes	6-Yes	12-Yes

Supplemental Table 1. Gene expression values changed greater than 2 fold.

Affymetrix ST 1.0 Array ID	0-No	6-No	12-No	0-Yes	6-Yes	12-Yes

Supplemental Table 1. Gene expression values changed greater than 2 fold.

Affymetrix ST 1.0 Array ID	0-No	6-No	12-No	0-Yes	6-Yes	12-Yes

Supplemental Table 1. Gene expression values changed greater than 2 fold.

Affymetrix ST 1.0 Array ID	0-No	6-No	12-No	0-Yes	6-Yes	12-Yes

Supplemental Table 1. Gene expression values changed greater than 2 fold.

Affymetrix ST 1.0 Array ID	0-No	6-No	12-No	0-Yes	6-Yes	12-Yes

Supplemental Table 1. Gene expression values changed greater than 2 fold.

Affymetrix ST 1.0 Array ID	0-No	6-No	12-No	0-Yes	6-Yes	12-Yes

Supplemental Table 1. Gene expression values changed greater than 2 fold.

Affymetrix ST 1.0 Array ID	0-No	6-No	12-No	0-Yes	6-Yes	12-Yes

Supplemental Table 1. Gene expression values changed greater than 2 fold.

Affymetrix ST 1.0 Array ID	0-No	6-No	12-No	0-Yes	6-Yes	12-Yes

Supplemental Table 1. Gene expression values changed greater than 2 fold.

Affymetrix ST 1.0 Array ID	0-No	6-No	12-No	0-Yes	6-Yes	12-Yes

Supplemental Table 1. Gene expression values changed greater than 2 fold.

Affymetrix ST 1.0 Array ID	0-No	6-No	12-No	0-Yes	6-Yes	12-Yes

Supplemental Table 1. Gene expression values changed greater than 2 fold.

Affymetrix ST 1.0 Array ID	0-No	6-No	12-No	0-Yes	6-Yes	12-Yes

Supplemental Table 1. Gene expression values changed greater than 2 fold.

Affymetrix ST 1.0 Array ID	0-No	6-No	12-No	0-Yes	6-Yes	12-Yes

Supplemental Table 1. Gene expression values changed greater than 2 fold.

Affymetrix ST 1.0 Array ID	0-No	6-No	12-No	0-Yes	6-Yes	12-Yes

Supplemental Table 1. Gene expression values changed greater than 2 fold.

Affymetrix ST 1.0 Array ID	0-No	6-No	12-No	0-Yes	6-Yes	12-Yes

Supplemental Table 1. Gene expression values changed greater than 2 fold.

Affymetrix ST 1.0 Array ID	0-No	6-No	12-No	0-Yes	6-Yes	12-Yes

Supplemental Table 1. Gene expression values changed greater than 2 fold.

Affymetrix ST 1.0 Array ID	0-No	6-No	12-No	0-Yes	6-Yes	12-Yes

Supplemental Table 1. Gene expression values changed greater than 2 fold.

Affymetrix ST 1.0 Array ID	0-No	6-No	12-No	0-Yes	6-Yes	12-Yes

Supplemental Table 1. Gene expression values changed greater than 2 fold.

Affymetrix ST 1.0 Array ID	0-No	6-No	12-No	0-Yes	6-Yes	12-Yes

Supplemental Table 1. Gene expression values changed greater than 2 fold.

Affymetrix ST 1.0 Array ID	0-No	6-No	12-No	0-Yes	6-Yes	12-Yes

Supplemental Table 1. Gene expression values changed greater than 2 fold.

Affymetrix ST 1.0 Array ID	0-No	6-No	12-No	0-Yes	6-Yes	12-Yes

Supplemental Table 1. Gene expression values changed greater than 2 fold.

Affymetrix ST 1.0 Array ID	0-No	6-No	12-No	0-Yes	6-Yes	12-Yes

Supplemental Table 1. Gene expression values changed greater than 2 fold.

Affymetrix ST 1.0 Array ID	0-No	6-No	12-No	0-Yes	6-Yes	12-Yes

Supplemental Table 1. Gene expression values changed greater than 2 fold.

Affymetrix ST 1.0 Array ID	0-No	6-No	12-No	0-Yes	6-Yes	12-Yes

Supplemental Table 1. Gene expression values changed greater than 2 fold.

Affymetrix ST 1.0 Array ID	0-No	6-No	12-No	0-Yes	6-Yes	12-Yes

Supplemental Table 1. Gene expression values changed greater than 2 fold.

Affymetrix ST 1.0 Array ID	0-No	6-No	12-No	0-Yes	6-Yes	12-Yes

Supplemental Table 1. Gene expression values changed greater than 2 fold.

Affymetrix ST 1.0 Array ID	0-No	6-No	12-No	0-Yes	6-Yes	12-Yes

Supplemental Table 1. Gene expression values changed greater than 2 fold.

Affymetrix ST 1.0 Array ID	0-No	6-No	12-No	0-Yes	6-Yes	12-Yes

Supplemental Table 1. Gene expression values changed greater than 2 fold.

Affymetrix ST 1.0 Array ID	0-No	6-No	12-No	0-Yes	6-Yes	12-Yes

Supplemental Table 1. Gene expression values changed greater than 2 fold.

Affymetrix ST 1.0 Array ID	0-No	6-No	12-No	0-Yes	6-Yes	12-Yes

Supplemental Table 1. Gene expression values changed greater than 2 fold.

Affymetrix ST 1.0 Array ID	0-No	6-No	12-No	0-Yes	6-Yes	12-Yes

Supplemental Table 1. Gene expression values changed greater than 2 fold.

Affymetrix ST 1.0 Array ID	0-No	6-No	12-No	0-Yes	6-Yes	12-Yes

Supplemental Table 1. Gene expression values changed greater than 2 fold.

Affymetrix ST 1.0 Array ID	0-No	6-No	12-No	0-Yes	6-Yes	12-Yes

Supplemental Table 1. Gene expression values changed greater than 2 fold.

Affymetrix ST 1.0 Array ID	0-No	6-No	12-No	0-Yes	6-Yes	12-Yes

Supplemental Table 1. Gene expression values changed greater than 2 fold.

Affymetrix ST 1.0 Array ID	0-No	6-No	12-No	0-Yes	6-Yes	12-Yes

Supplemental Table 1. Gene expression values changed greater than 2 fold.

Affymetrix ST 1.0 Array ID	0-No	6-No	12-No	0-Yes	6-Yes	12-Yes

Supplemental Table 1. Gene expression values changed greater than 2 fold.

Affymetrix ST 1.0 Array ID	0-No	6-No	12-No	0-Yes	6-Yes	12-Yes

Supplemental Table 1. Gene expression values changed greater than 2 fold.

Affymetrix ST 1.0 Array ID	0-No	6-No	12-No	0-Yes	6-Yes	12-Yes

Supplemental Table 1. Gene expression values changed greater than 2 fold.

Affymetrix ST 1.0 Array ID	0-No	6-No	12-No	0-Yes	6-Yes	12-Yes

Supplemental Table 1. Gene expression values changed greater than 2 fold.

Affymetrix ST 1.0 Array ID	0-No	6-No	12-No	0-Yes	6-Yes	12-Yes

Supplemental Table 1. Gene expression values changed greater than 2 fold.

Affymetrix ST 1.0 Array ID	0-No	6-No	12-No	0-Yes	6-Yes	12-Yes

Supplemental Table 1. Gene expression values changed greater than 2 fold.

Affymetrix ST 1.0 Array ID	0-No	6-No	12-No	0-Yes	6-Yes	12-Yes

Supplemental Table 1. Gene expression values changed greater than 2 fold.

Affymetrix ST 1.0 Array ID	0-No	6-No	12-No	0-Yes	6-Yes	12-Yes

Supplemental Table 1. Gene expression values changed greater than 2 fold.

Affymetrix ST 1.0 Array ID	0-No	6-No	12-No	0-Yes	6-Yes	12-Yes

Supplemental Table 1. Gene expression values changed greater than 2 fold.

Affymetrix ST 1.0 Array ID	0-No	6-No	12-No	0-Yes	6-Yes	12-Yes

Supplemental Table 1. Gene expression values changed greater than 2 fold.

Affymetrix ST 1.0 Array ID	0-No	6-No	12-No	0-Yes	6-Yes	12-Yes

Supplemental Table 1. Gene expression values changed greater than 2 fold.

Affymetrix ST 1.0 Array ID	0-No	6-No	12-No	0-Yes	6-Yes	12-Yes

Supplemental Table 1. Gene expression values changed greater than 2 fold.

Affymetrix ST 1.0 Array ID	0-No	6-No	12-No	0-Yes	6-Yes	12-Yes

Supplemental Table 1. Gene expression values changed greater than 2 fold.

Affymetrix ST 1.0 Array ID	0-No	6-No	12-No	0-Yes	6-Yes	12-Yes

Supplemental Table 1. Gene expression values changed greater than 2 fold.

Affymetrix ST 1.0 Array ID	0-No	6-No	12-No	0-Yes	6-Yes	12-Yes

Supplemental Table 1. Gene expression values changed greater than 2 fold.

Affymetrix ST 1.0 Array ID	0-No	6-No	12-No	0-Yes	6-Yes	12-Yes

Supplemental Table 1. Gene expression values changed greater than 2 fold.

Affymetrix ST 1.0 Array ID	0-No	6-No	12-No	0-Yes	6-Yes	12-Yes

Supplemental Table 1. Gene expression values changed greater than 2 fold.

Affymetrix ST 1.0 Array ID	0-No	6-No	12-No	0-Yes	6-Yes	12-Yes

Supplemental Table 1. Gene expression values changed greater than 2 fold.

Affymetrix ST 1.0 Array ID	0-No	6-No	12-No	0-Yes	6-Yes	12-Yes

Supplemental Table 1. Gene expression values changed greater than 2 fold.

Affymetrix ST 1.0 Array ID	0-No	6-No	12-No	0-Yes	6-Yes	12-Yes

Supplemental Table 1. Gene expression values changed greater than 2 fold.

Affymetrix ST 1.0 Array ID	0-No	6-No	12-No	0-Yes	6-Yes	12-Yes

Supplemental Table 1. Gene expression values changed greater than 2 fold.

Affymetrix ST 1.0 Array ID	0-No	6-No	12-No	0-Yes	6-Yes	12-Yes

Supplemental Table 1. Gene expression values changed greater than 2 fold.

Affymetrix ST 1.0 Array ID	0-No	6-No	12-No	0-Yes	6-Yes	12-Yes

Supplemental Table 1. Gene expression values changed greater than 2 fold.

Affymetrix ST 1.0 Array ID	0-No	6-No	12-No	0-Yes	6-Yes	12-Yes

Supplemental Table 1. Gene expression values changed greater than 2 fold.

Affymetrix ST 1.0 Array ID	0-No	6-No	12-No	0-Yes	6-Yes	12-Yes

Supplemental Table 1. Gene expression values changed greater than 2 fold.

Affymetrix ST 1.0 Array ID	0-No	6-No	12-No	0-Yes	6-Yes	12-Yes

Supplemental Table 1. Gene expression values changed greater than 2 fold.

Affymetrix ST 1.0 Array ID	0-No	6-No	12-No	0-Yes	6-Yes	12-Yes

Supplemental Table 1. Gene expression values changed greater than 2 fold.

Affymetrix ST 1.0 Array ID	0-No	6-No	12-No	0-Yes	6-Yes	12-Yes

Supplemental Table 1. Gene expression values changed greater than 2 fold.

Affymetrix ST 1.0 Array ID	0-No	6-No	12-No	0-Yes	6-Yes	12-Yes

Supplemental Table 1. Gene expression values changed greater than 2 fold.

Affymetrix ST 1.0 Array ID	0-No	6-No	12-No	0-Yes	6-Yes	12-Yes

Supplemental Table 1. Gene expression values changed greater than 2 fold.

Affymetrix ST 1.0 Array ID	0-No	6-No	12-No	0-Yes	6-Yes	12-Yes

Supplemental Table 1. Gene expression values changed greater than 2 fold.

Affymetrix ST 1.0 Array ID	0-No	6-No	12-No	0-Yes	6-Yes	12-Yes

Supplemental Table 1. Gene expression values changed greater than 2 fold.

Affymetrix ST 1.0 Array ID	0-No	6-No	12-No	0-Yes	6-Yes	12-Yes

Supplemental Table 1. Gene expression values changed greater than 2 fold.

Affymetrix ST 1.0 Array ID	0-No	6-No	12-No	0-Yes	6-Yes	12-Yes

Supplemental Table 1. Gene expression values changed greater than 2 fold.

Affymetrix ST 1.0 Array ID	0-No	6-No	12-No	0-Yes	6-Yes	12-Yes

Supplemental Table 1. Gene expression values changed greater than 2 fold.

Affymetrix ST 1.0 Array ID	0-No	6-No	12-No	0-Yes	6-Yes	12-Yes

Supplemental Table 1. Gene expression values changed greater than 2 fold.

Affymetrix ST 1.0 Array ID	0-No	6-No	12-No	0-Yes	6-Yes	12-Yes

Supplemental Table 1. Gene expression values changed greater than 2 fold.

Affymetrix ST 1.0 Array ID	0-No	6-No	12-No	0-Yes	6-Yes	12-Yes

Supplemental Table 1. Gene expression values changed greater than 2 fold.

Affymetrix ST 1.0 Array ID	0-No	6-No	12-No	0-Yes	6-Yes	12-Yes

Supplemental Table 1. Gene expression values changed greater than 2 fold.

Affymetrix ST 1.0 Array ID	0-No	6-No	12-No	0-Yes	6-Yes	12-Yes

Supplemental Table 1. Gene expression values changed greater than 2 fold.

Affymetrix ST 1.0 Array ID	0-No	6-No	12-No	0-Yes	6-Yes	12-Yes

Supplemental Table 1. Gene expression values changed greater than 2 fold.

Affymetrix ST 1.0 Array ID	0-No	6-No	12-No	0-Yes	6-Yes	12-Yes

Supplemental Table 1. Gene expression values changed greater than 2 fold.

Affymetrix ST 1.0 Array ID	0-No	6-No	12-No	0-Yes	6-Yes	12-Yes

Supplemental Table 1. Gene expression values changed greater than 2 fold.

Affymetrix ST 1.0 Array ID	0-No	6-No	12-No	0-Yes	6-Yes	12-Yes

Supplemental Table 1. Gene expression values changed greater than 2 fold.

Affymetrix ST 1.0 Array ID	0-No	6-No	12-No	0-Yes	6-Yes	12-Yes

Supplemental Table 1. Gene expression values changed greater than 2 fold.

Affymetrix ST 1.0 Array ID	0-No	6-No	12-No	0-Yes	6-Yes	12-Yes

Supplemental Table 1. Gene expression values changed greater than 2 fold.

Affymetrix ST 1.0 Array ID	0-No	6-No	12-No	0-Yes	6-Yes	12-Yes

Supplemental Table 1. Gene expression values changed greater than 2 fold.

Affymetrix ST 1.0 Array ID	0-No	6-No	12-No	0-Yes	6-Yes	12-Yes

Supplemental Table 1. Gene expression values changed greater than 2 fold.

Affymetrix ST 1.0 Array ID	0-No	6-No	12-No	0-Yes	6-Yes	12-Yes

Supplemental Table 1. Gene expression values changed greater than 2 fold.

Affymetrix ST 1.0 Array ID	0-No	6-No	12-No	0-Yes	6-Yes	12-Yes

Supplemental Table 1. Gene expression values changed greater than 2 fold.

Affymetrix ST 1.0 Array ID	0-No	6-No	12-No	0-Yes	6-Yes	12-Yes

Supplemental Table 1. Gene expression values changed greater than 2 fold.

Affymetrix ST 1.0 Array ID	0-No	6-No	12-No	0-Yes	6-Yes	12-Yes

Supplemental Table 1. Gene expression values changed greater than 2 fold.

Affymetrix ST 1.0 Array ID	0-No	6-No	12-No	0-Yes	6-Yes	12-Yes

Supplemental Table 1. Gene expression values changed greater than 2 fold.

Affymetrix ST 1.0 Array ID	0-No	6-No	12-No	0-Yes	6-Yes	12-Yes

Supplemental Table 1. Gene expression values changed greater than 2 fold.

Affymetrix ST 1.0 Array ID	0-No	6-No	12-No	0-Yes	6-Yes	12-Yes

Supplemental Table 1. Gene expression values changed greater than 2 fold.

Affymetrix ST 1.0 Array ID	0-No	6-No	12-No	0-Yes	6-Yes	12-Yes

Supplemental Table 1. Gene expression values changed greater than 2 fold.

Affymetrix ST 1.0 Array ID	0-No	6-No	12-No	0-Yes	6-Yes	12-Yes

Supplemental Table 1. Gene expression values changed greater than 2 fold.

Affymetrix ST 1.0 Array ID	0-No	6-No	12-No	0-Yes	6-Yes	12-Yes

Supplemental Table 1. Gene expression values changed greater than 2 fold.

Affymetrix ST 1.0 Array ID	0-No	6-No	12-No	0-Yes	6-Yes	12-Yes

Supplemental Table 1. Gene expression values changed greater than 2 fold.

Affymetrix ST 1.0 Array ID	0-No	6-No	12-No	0-Yes	6-Yes	12-Yes

Supplemental Table 1. Gene expression values changed greater than 2 fold.

Affymetrix ST 1.0 Array ID	0-No	6-No	12-No	0-Yes	6-Yes	12-Yes

Supplemental Table 1. Gene expression values changed greater than 2 fold.

Affymetrix ST 1.0 Array ID	0-No	6-No	12-No	0-Yes	6-Yes	12-Yes

Supplemental Table 1. Gene expression values changed greater than 2 fold.

Affymetrix ST 1.0 Array ID	0-No	6-No	12-No	0-Yes	6-Yes	12-Yes

Supplemental Table 1. Gene expression values changed greater than 2 fold.

Affymetrix ST 1.0 Array ID	0-No	6-No	12-No	0-Yes	6-Yes	12-Yes

Supplemental Table 1. Gene expression values changed greater than 2 fold.

Affymetrix ST 1.0 Array ID	0-No	6-No	12-No	0-Yes	6-Yes	12-Yes

Supplemental Table 1. Gene expression values changed greater than 2 fold.

Affymetrix ST 1.0 Array ID	0-No	6-No	12-No	0-Yes	6-Yes	12-Yes

Supplemental Table 1. Gene expression values changed greater than 2 fold.

Affymetrix ST 1.0 Array ID	0-No	6-No	12-No	0-Yes	6-Yes	12-Yes

Supplemental Table 1. Gene expression values changed greater than 2 fold.

Affymetrix ST 1.0 Array ID	0-No	6-No	12-No	0-Yes	6-Yes	12-Yes

Supplemental Table 1. Gene expression values changed greater than 2 fold.

Affymetrix ST 1.0 Array ID	0-No	6-No	12-No	0-Yes	6-Yes	12-Yes

Supplemental Table 1. Gene expression values changed greater than 2 fold.

Affymetrix ST 1.0 Array ID	0-No	6-No	12-No	0-Yes	6-Yes	12-Yes

Supplemental Table 1. Gene expression values changed greater than 2 fold.

Affymetrix ST 1.0 Array ID	0-No	6-No	12-No	0-Yes	6-Yes	12-Yes

Supplemental Table 1. Gene expression values changed greater than 2 fold.

Affymetrix ST 1.0 Array ID	0-No	6-No	12-No	0-Yes	6-Yes	12-Yes

Supplemental Table 1. Gene expression values changed greater than 2 fold.

Affymetrix ST 1.0 Array ID	0-No	6-No	12-No	0-Yes	6-Yes	12-Yes

Supplemental Table 1. Gene expression values changed greater than 2 fold.

Affymetrix ST 1.0 Array ID	0-No	6-No	12-No	0-Yes	6-Yes	12-Yes

Supplemental Table 1. Gene expression values changed greater than 2 fold.

Affymetrix ST 1.0 Array ID	0-No	6-No	12-No	0-Yes	6-Yes	12-Yes

Supplemental Table 1. Gene expression values changed greater than 2 fold.

Affymetrix ST 1.0 Array ID	0-No	6-No	12-No	0-Yes	6-Yes	12-Yes

Supplemental Table 1. Gene expression values changed greater than 2 fold.

Affymetrix ST 1.0 Array ID	0-No	6-No	12-No	0-Yes	6-Yes	12-Yes

Supplemental Table 1. Gene expression values changed greater than 2 fold.

Affymetrix ST 1.0 Array ID	0-No	6-No	12-No	0-Yes	6-Yes	12-Yes

Supplemental Table 1. Gene expression values changed greater than 2 fold.

Affymetrix ST 1.0 Array ID	0-No	6-No	12-No	0-Yes	6-Yes	12-Yes

Supplemental Table 1. Gene expression values changed greater than 2 fold.

Affymetrix ST 1.0 Array ID	0-No	6-No	12-No	0-Yes	6-Yes	12-Yes

Supplemental Table 1. Gene expression values changed greater than 2 fold.

Affymetrix ST 1.0 Array ID	0-No	6-No	12-No	0-Yes	6-Yes	12-Yes

Supplemental Table 1. Gene expression values changed greater than 2 fold.

Affymetrix ST 1.0 Array ID	0-No	6-No	12-No	0-Yes	6-Yes	12-Yes

Supplemental Table 1. Gene expression values changed greater than 2 fold.

Affymetrix ST 1.0 Array ID	0-No	6-No	12-No	0-Yes	6-Yes	12-Yes

Supplemental Table 1. Gene expression values changed greater than 2 fold.

Affymetrix ST 1.0 Array ID	0-No	6-No	12-No	0-Yes	6-Yes	12-Yes

Supplemental Table 1. Gene expression values changed greater than 2 fold.

Affymetrix ST 1.0 Array ID	0-No	6-No	12-No	0-Yes	6-Yes	12-Yes

Supplemental Table 1. Gene expression values changed greater than 2 fold.

Affymetrix ST 1.0 Array ID	0-No	6-No	12-No	0-Yes	6-Yes	12-Yes

Supplemental Table 1. Gene expression values changed greater than 2 fold.

Affymetrix ST 1.0 Array ID	0-No	6-No	12-No	0-Yes	6-Yes	12-Yes

Supplemental Table 1. Gene expression values changed greater than 2 fold.

Affymetrix ST 1.0 Array ID	0-No	6-No	12-No	0-Yes	6-Yes	12-Yes

Supplemental Table 1. Gene expression values changed greater than 2 fold.

Affymetrix ST 1.0 Array ID	0-No	6-No	12-No	0-Yes	6-Yes	12-Yes

Supplemental Table 1. Gene expression values changed greater than 2 fold.

Affymetrix ST 1.0 Array ID	0-No	6-No	12-No	0-Yes	6-Yes	12-Yes

Supplemental Table 1. Gene expression values changed greater than 2 fold.

Affymetrix ST 1.0 Array ID	0-No	6-No	12-No	0-Yes	6-Yes	12-Yes

Supplemental Table 1. Gene expression values changed greater than 2 fold.

Affymetrix ST 1.0 Array ID	0-No	6-No	12-No	0-Yes	6-Yes	12-Yes

Supplemental Table 1. Gene expression values changed greater than 2 fold.

Affymetrix ST 1.0 Array ID	0-No	6-No	12-No	0-Yes	6-Yes	12-Yes

Supplemental Table 1. Gene expression values changed greater than 2 fold.

Affymetrix ST 1.0 Array ID	0-No	6-No	12-No	0-Yes	6-Yes	12-Yes

Supplemental Table 1. Gene expression values changed greater than 2 fold.

Affymetrix ST 1.0 Array ID	0-No	6-No	12-No	0-Yes	6-Yes	12-Yes

Supplemental Table 1. Gene expression values changed greater than 2 fold.

Affymetrix ST 1.0 Array ID	0-No	6-No	12-No	0-Yes	6-Yes	12-Yes

Supplemental Table 1. Gene expression values changed greater than 2 fold.

Affymetrix ST 1.0 Array ID	0-No	6-No	12-No	0-Yes	6-Yes	12-Yes

Supplemental Table 1. Gene expression values changed greater than 2 fold.

Affymetrix ST 1.0 Array ID	0-No	6-No	12-No	0-Yes	6-Yes	12-Yes

Supplemental Table 1. Gene expression values changed greater than 2 fold.

Affymetrix ST 1.0 Array ID	0-No	6-No	12-No	0-Yes	6-Yes	12-Yes

Supplemental Table 1. Gene expression values changed greater than 2 fold.

Affymetrix ST 1.0 Array ID	0-No	6-No	12-No	0-Yes	6-Yes	12-Yes

Supplemental Table 1. Gene expression values changed greater than 2 fold.

Affymetrix ST 1.0 Array ID	0-No	6-No	12-No	0-Yes	6-Yes	12-Yes

Supplemental Table 1. Gene expression values changed greater than 2 fold.

Affymetrix ST 1.0 Array ID	0-No	6-No	12-No	0-Yes	6-Yes	12-Yes

Supplemental Table 1. Gene expression values changed greater than 2 fold.

Affymetrix ST 1.0 Array ID	0-No	6-No	12-No	0-Yes	6-Yes	12-Yes

Supplemental Table 1. Gene expression values changed greater than 2 fold.

Affymetrix ST 1.0 Array ID	0-No	6-No	12-No	0-Yes	6-Yes	12-Yes

Supplemental Table 1. Gene expression values changed greater than 2 fold.

Affymetrix ST 1.0 Array ID	0-No	6-No	12-No	0-Yes	6-Yes	12-Yes

Supplemental Table 1. Gene expression values changed greater than 2 fold.

Affymetrix ST 1.0 Array ID	0-No	6-No	12-No	0-Yes	6-Yes	12-Yes

Supplemental Table 1. Gene expression values changed greater than 2 fold.

Affymetrix ST 1.0 Array ID	0-No	6-No	12-No	0-Yes	6-Yes	12-Yes

Supplemental Table 1. Gene expression values changed greater than 2 fold.

Affymetrix ST 1.0 Array ID	0-No	6-No	12-No	0-Yes	6-Yes	12-Yes

Supplemental Table 1. Gene expression values changed greater than 2 fold.

Affymetrix ST 1.0 Array ID	0-No	6-No	12-No	0-Yes	6-Yes	12-Yes

Supplemental Table 1. Gene expression values changed greater than 2 fold.

Affymetrix ST 1.0 Array ID	0-No	6-No	12-No	0-Yes	6-Yes	12-Yes

Supplemental Table 1. Gene expression values changed greater than 2 fold.

Affymetrix ST 1.0 Array ID	0-No	6-No	12-No	0-Yes	6-Yes	12-Yes

Supplemental Table 1. Gene expression values changed greater than 2 fold.

Affymetrix ST 1.0 Array ID	0-No	6-No	12-No	0-Yes	6-Yes	12-Yes

Supplemental Table 1. Gene expression values changed greater than 2 fold.

Affymetrix ST 1.0 Array ID	0-No	6-No	12-No	0-Yes	6-Yes	12-Yes

Supplemental Table 1. Gene expression values changed greater than 2 fold.

Affymetrix ST 1.0 Array ID	0-No	6-No	12-No	0-Yes	6-Yes	12-Yes

Supplemental Table 1. Gene expression values changed greater than 2 fold.

Affymetrix ST 1.0 Array ID	0-No	6-No	12-No	0-Yes	6-Yes	12-Yes

Supplemental Table 1. Gene expression values changed greater than 2 fold.

Affymetrix ST 1.0 Array ID	0-No	6-No	12-No	0-Yes	6-Yes	12-Yes

Supplemental Table 1. Gene expression values changed greater than 2 fold.

Affymetrix ST 1.0 Array ID	0-No	6-No	12-No	0-Yes	6-Yes	12-Yes

Supplemental Table 1. Gene expression values changed greater than 2 fold.

Affymetrix ST 1.0 Array ID	0-No	6-No	12-No	0-Yes	6-Yes	12-Yes

Supplemental Table 1. Gene expression values changed greater than 2 fold.

Affymetrix ST 1.0 Array ID	0-No	6-No	12-No	0-Yes	6-Yes	12-Yes

Supplemental Table 1. Gene expression values changed greater than 2 fold.

Affymetrix ST 1.0 Array ID	0-No	6-No	12-No	0-Yes	6-Yes	12-Yes

Supplemental Table 1. Gene expression values changed greater than 2 fold.

Affymetrix ST 1.0 Array ID	0-No	6-No	12-No	0-Yes	6-Yes	12-Yes

Supplemental Table 1. Gene expression values changed greater than 2 fold.

Affymetrix ST 1.0 Array ID	0-No	6-No	12-No	0-Yes	6-Yes	12-Yes

Supplemental Table 1. Gene expression values changed greater than 2 fold.

Affymetrix ST 1.0 Array ID	0-No	6-No	12-No	0-Yes	6-Yes	12-Yes

Supplemental Table 1. Gene expression values changed greater than 2 fold.

Affymetrix ST 1.0 Array ID	0-No	6-No	12-No	0-Yes	6-Yes	12-Yes

Supplemental Table 1. Gene expression values changed greater than 2 fold.

Affymetrix ST 1.0 Array ID	0-No	6-No	12-No	0-Yes	6-Yes	12-Yes

Supplemental Table 1. Gene expression values changed greater than 2 fold.

Affymetrix ST 1.0 Array ID	0-No	6-No	12-No	0-Yes	6-Yes	12-Yes

Supplemental Table 1. Gene expression values changed greater than 2 fold.

Affymetrix ST 1.0 Array ID	0-No	6-No	12-No	0-Yes	6-Yes	12-Yes

Supplemental Table 1. Gene expression values changed greater than 2 fold.

Affymetrix ST 1.0 Array ID	0-No	6-No	12-No	0-Yes	6-Yes	12-Yes

Supplemental Table 1. Gene expression values changed greater than 2 fold.

Affymetrix ST 1.0 Array ID	0-No	6-No	12-No	0-Yes	6-Yes	12-Yes

Supplemental Table 1. Gene expression values changed greater than 2 fold.

Affymetrix ST 1.0 Array ID	0-No	6-No	12-No	0-Yes	6-Yes	12-Yes

Supplemental Table 1. Gene expression values changed greater than 2 fold.

Affymetrix ST 1.0 Array ID	0-No	6-No	12-No	0-Yes	6-Yes	12-Yes

Supplemental Table 1. Gene expression values changed greater than 2 fold.

Affymetrix ST 1.0 Array ID	0-No	6-No	12-No	0-Yes	6-Yes	12-Yes

Supplemental Table 1. Gene expression values changed greater than 2 fold.

Affymetrix ST 1.0 Array ID	0-No	6-No	12-No	0-Yes	6-Yes	12-Yes

Supplemental Table 1. Gene expression values changed greater than 2 fold.

Affymetrix ST 1.0 Array ID	0-No	6-No	12-No	0-Yes	6-Yes	12-Yes

Supplemental Table 1. Gene expression values changed greater than 2 fold.

Affymetrix ST 1.0 Array ID	0-No	6-No	12-No	0-Yes	6-Yes	12-Yes

Supplemental Table 1. Gene expression values changed greater than 2 fold.

Affymetrix ST 1.0 Array ID	0-No	6-No	12-No	0-Yes	6-Yes	12-Yes

Supplemental Table 1. Gene expression values changed greater than 2 fold.

Affymetrix ST 1.0 Array ID	0-No	6-No	12-No	0-Yes	6-Yes	12-Yes

Supplemental Table 1. Gene expression values changed greater than 2 fold.

Affymetrix ST 1.0 Array ID	0-No	6-No	12-No	0-Yes	6-Yes	12-Yes

Supplemental Table 1. Gene expression values changed greater than 2 fold.

Affymetrix ST 1.0 Array ID	0-No	6-No	12-No	0-Yes	6-Yes	12-Yes

Supplemental Table 1. Gene expression values changed greater than 2 fold.

Affymetrix ST 1.0 Array ID	0-No	6-No	12-No	0-Yes	6-Yes	12-Yes

Supplemental Table 1. Gene expression values changed greater than 2 fold.

Affymetrix ST 1.0 Array ID	0-No	6-No	12-No	0-Yes	6-Yes	12-Yes

Supplemental Table 1. Gene expression values changed greater than 2 fold.

Affymetrix ST 1.0 Array ID	0-No	6-No	12-No	0-Yes	6-Yes	12-Yes

Supplemental Table 1. Gene expression values changed greater than 2 fold.

Affymetrix ST 1.0 Array ID	0-No	6-No	12-No	0-Yes	6-Yes	12-Yes

Supplemental Table 1. Gene expression values changed greater than 2 fold.

Affymetrix ST 1.0 Array ID	0-No	6-No	12-No	0-Yes	6-Yes	12-Yes

Supplemental Table 1. Gene expression values changed greater than 2 fold.

Affymetrix ST 1.0 Array ID	0-No	6-No	12-No	0-Yes	6-Yes	12-Yes

Supplemental Table 1. Gene expression values changed greater than 2 fold.

Affymetrix ST 1.0 Array ID	0-No	6-No	12-No	0-Yes	6-Yes	12-Yes

Supplemental Table 1. Gene expression values changed greater than 2 fold.

Affymetrix ST 1.0 Array ID	0-No	6-No	12-No	0-Yes	6-Yes	12-Yes

Supplemental Table 1. Gene expression values changed greater than 2 fold.

Affymetrix ST 1.0 Array ID	0-No	6-No	12-No	0-Yes	6-Yes	12-Yes

Supplemental Table 1. Gene expression values changed greater than 2 fold.

Affymetrix ST 1.0 Array ID	0-No	6-No	12-No	0-Yes	6-Yes	12-Yes

Supplemental Table 1. Gene expression values changed greater than 2 fold.

Affymetrix ST 1.0 Array ID	0-No	6-No	12-No	0-Yes	6-Yes	12-Yes

Supplemental Table 1. Gene expression values changed greater than 2 fold.

Affymetrix ST 1.0 Array ID	0-No	6-No	12-No	0-Yes	6-Yes	12-Yes

Supplemental Table 1. Gene expression values changed greater than 2 fold.

Affymetrix ST 1.0 Array ID	0-No	6-No	12-No	0-Yes	6-Yes	12-Yes

Supplemental Table 1. Gene expression values changed greater than 2 fold.

Affymetrix ST 1.0 Array ID	0-No	6-No	12-No	0-Yes	6-Yes	12-Yes

Supplemental Table 1. Gene expression values changed greater than 2 fold.

Affymetrix ST 1.0 Array ID	0-No	6-No	12-No	0-Yes	6-Yes	12-Yes

Supplemental Table 1. Gene expression values changed greater than 2 fold.

Affymetrix ST 1.0 Array ID	0-No	6-No	12-No	0-Yes	6-Yes	12-Yes

Supplemental Table 1. Gene expression values changed greater than 2 fold.

Affymetrix ST 1.0 Array ID	0-No	6-No	12-No	0-Yes	6-Yes	12-Yes

Supplemental Table 1. Gene expression values changed greater than 2 fold.

Affymetrix ST 1.0 Array ID	0-No	6-No	12-No	0-Yes	6-Yes	12-Yes

Supplemental Table 1. Gene expression values changed greater than 2 fold.

Affymetrix ST 1.0 Array ID	0-No	6-No	12-No	0-Yes	6-Yes	12-Yes

Supplemental Table 1. Gene expression values changed greater than 2 fold.

Affymetrix ST 1.0 Array ID	0-No	6-No	12-No	0-Yes	6-Yes	12-Yes

Supplemental Table 1. Gene expression values changed greater than 2 fold.

Affymetrix ST 1.0 Array ID	0-No	6-No	12-No	0-Yes	6-Yes	12-Yes

Supplemental Table 1. Gene expression values changed greater than 2 fold.

Affymetrix ST 1.0 Array ID	0-No	6-No	12-No	0-Yes	6-Yes	12-Yes

Supplemental Table 1. Gene expression values changed greater than 2 fold.

Affymetrix ST 1.0 Array ID	0-No	6-No	12-No	0-Yes	6-Yes	12-Yes

Supplemental Table 1. Gene expression values changed greater than 2 fold.

Affymetrix ST 1.0 Array ID	0-No	6-No	12-No	0-Yes	6-Yes	12-Yes

Supplemental Table 1. Gene expression values changed greater than 2 fold.

Affymetrix ST 1.0 Array ID	0-No	6-No	12-No	0-Yes	6-Yes	12-Yes

Supplemental Table 1. Gene expression values changed greater than 2 fold.

Affymetrix ST 1.0 Array ID	0-No	6-No	12-No	0-Yes	6-Yes	12-Yes

Supplemental Table 1. Gene expression values changed greater than 2 fold.

Affymetrix ST 1.0 Array ID	0-No	6-No	12-No	0-Yes	6-Yes	12-Yes

Supplemental Table 1. Gene expression values changed greater than 2 fold.

Affymetrix ST 1.0 Array ID	0-No	6-No	12-No	0-Yes	6-Yes	12-Yes

Supplemental Table 1. Gene expression values changed greater than 2 fold.

Affymetrix ST 1.0 Array ID	0-No	6-No	12-No	0-Yes	6-Yes	12-Yes

Supplemental Table 1. Gene expression values changed greater than 2 fold.

Affymetrix ST 1.0 Array ID	0-No	6-No	12-No	0-Yes	6-Yes	12-Yes

Supplemental Table 1. Gene expression values changed greater than 2 fold.

Affymetrix ST 1.0 Array ID	0-No	6-No	12-No	0-Yes	6-Yes	12-Yes

Supplemental Table 1. Gene expression values changed greater than 2 fold.

Affymetrix ST 1.0 Array ID	0-No	6-No	12-No	0-Yes	6-Yes	12-Yes

Supplemental Table 1. Gene expression values changed greater than 2 fold.

Affymetrix ST 1.0 Array ID	0-No	6-No	12-No	0-Yes	6-Yes	12-Yes

Supplemental Table 1. Gene expression values changed greater than 2 fold.

Affymetrix ST 1.0 Array ID	0-No	6-No	12-No	0-Yes	6-Yes	12-Yes

Supplemental Table 1. Gene expression values changed greater than 2 fold.

Affymetrix ST 1.0 Array ID	0-No	6-No	12-No	0-Yes	6-Yes	12-Yes

Supplemental Table 1. Gene expression values changed greater than 2 fold.

Affymetrix ST 1.0 Array ID	0-No	6-No	12-No	0-Yes	6-Yes	12-Yes

Supplemental Table 1. Gene expression values changed greater than 2 fold.

Affymetrix ST 1.0 Array ID	0-No	6-No	12-No	0-Yes	6-Yes	12-Yes

Supplemental Table 1. Gene expression values changed greater than 2 fold.

Affymetrix ST 1.0 Array ID	0-No	6-No	12-No	0-Yes	6-Yes	12-Yes

Supplemental Table 1. Gene expression values changed greater than 2 fold.

Affymetrix ST 1.0 Array ID	0-No	6-No	12-No	0-Yes	6-Yes	12-Yes

Supplemental Table 1. Gene expression values changed greater than 2 fold.

Affymetrix ST 1.0 Array ID	0-No	6-No	12-No	0-Yes	6-Yes	12-Yes

Supplemental Table 1. Gene expression values changed greater than 2 fold.

Affymetrix ST 1.0 Array ID	0-No	6-No	12-No	0-Yes	6-Yes	12-Yes

Supplemental Table 1. Gene expression values changed greater than 2 fold.

Affymetrix ST 1.0 Array ID	0-No	6-No	12-No	0-Yes	6-Yes	12-Yes

Supplemental Table 1. Gene expression values changed greater than 2 fold.

Affymetrix ST 1.0 Array ID	0-No	6-No	12-No	0-Yes	6-Yes	12-Yes

Supplemental Table 1. Gene expression values changed greater than 2 fold.

Affymetrix ST 1.0 Array ID	0-No	6-No	12-No	0-Yes	6-Yes	12-Yes

Supplemental Table 1. Gene expression values changed greater than 2 fold.

Affymetrix ST 1.0 Array ID	0-No	6-No	12-No	0-Yes	6-Yes	12-Yes

Supplemental Table 1. Gene expression values changed greater than 2 fold.

Affymetrix ST 1.0 Array ID	0-No	6-No	12-No	0-Yes	6-Yes	12-Yes

Supplemental Table 1. Gene expression values changed greater than 2 fold.

Affymetrix ST 1.0 Array ID	0-No	6-No	12-No	0-Yes	6-Yes	12-Yes

Supplemental Table 1. Gene expression values changed greater than 2 fold.

Affymetrix ST 1.0 Array ID	0-No	6-No	12-No	0-Yes	6-Yes	12-Yes

Supplemental Table 1. Gene expression values changed greater than 2 fold.

Affymetrix ST 1.0 Array ID	0-No	6-No	12-No	0-Yes	6-Yes	12-Yes

Supplemental Table 1. Gene expression values changed greater than 2 fold.

Affymetrix ST 1.0 Array ID	0-No	6-No	12-No	0-Yes	6-Yes	12-Yes

Supplemental Table 1. Gene expression values changed greater than 2 fold.

Affymetrix ST 1.0 Array ID	0-No	6-No	12-No	0-Yes	6-Yes	12-Yes

Supplemental Table 1. Gene expression values changed greater than 2 fold.

Affymetrix ST 1.0 Array ID	0-No	6-No	12-No	0-Yes	6-Yes	12-Yes

Supplemental Table 1. Gene expression values changed greater than 2 fold.

Affymetrix ST 1.0 Array ID	0-No	6-No	12-No	0-Yes	6-Yes	12-Yes

Supplemental Table 1. Gene expression values changed greater than 2 fold.

Affymetrix ST 1.0 Array ID	0-No	6-No	12-No	0-Yes	6-Yes	12-Yes

Supplemental Table 1. Gene expression values changed greater than 2 fold.

Affymetrix ST 1.0 Array ID	0-No	6-No	12-No	0-Yes	6-Yes	12-Yes

Supplemental Table 1. Gene expression values changed greater than 2 fold.

Affymetrix ST 1.0 Array ID	0-No	6-No	12-No	0-Yes	6-Yes	12-Yes

Supplemental Table 1. Gene expression values changed greater than 2 fold.

Affymetrix ST 1.0 Array ID	0-No	6-No	12-No	0-Yes	6-Yes	12-Yes

Supplemental Table 1. Gene expression values changed greater than 2 fold.

Affymetrix ST 1.0 Array ID	0-No	6-No	12-No	0-Yes	6-Yes	12-Yes

Supplemental Table 1. Gene expression values changed greater than 2 fold.

Affymetrix ST 1.0 Array ID	0-No	6-No	12-No	0-Yes	6-Yes	12-Yes

Supplemental Table 1. Gene expression values changed greater than 2 fold.

Affymetrix ST 1.0 Array ID	0-No	6-No	12-No	0-Yes	6-Yes	12-Yes

Supplemental Table 1. Gene expression values changed greater than 2 fold.

Affymetrix ST 1.0 Array ID	0-No	6-No	12-No	0-Yes	6-Yes	12-Yes

Supplemental Table 1. Gene expression values changed greater than 2 fold.

Affymetrix ST 1.0 Array ID	0-No	6-No	12-No	0-Yes	6-Yes	12-Yes

Supplemental Table 1. Gene expression values changed greater than 2 fold.

Affymetrix ST 1.0 Array ID	0-No	6-No	12-No	0-Yes	6-Yes	12-Yes

Supplemental Table 1. Gene expression values changed greater than 2 fold.

Affymetrix ST 1.0 Array ID	0-No	6-No	12-No	0-Yes	6-Yes	12-Yes

Supplemental Table 1. Gene expression values changed greater than 2 fold.

Affymetrix ST 1.0 Array ID	0-No	6-No	12-No	0-Yes	6-Yes	12-Yes

Supplemental Table 1. Gene expression values changed greater than 2 fold.

Affymetrix ST 1.0 Array ID	0-No	6-No	12-No	0-Yes	6-Yes	12-Yes

Supplemental Table 1. Gene expression values changed greater than 2 fold.

Affymetrix ST 1.0 Array ID	0-No	6-No	12-No	0-Yes	6-Yes	12-Yes

Supplemental Table 1. Gene expression values changed greater than 2 fold.

Affymetrix ST 1.0 Array ID	0-No	6-No	12-No	0-Yes	6-Yes	12-Yes

Supplemental Table 1. Gene expression values changed greater than 2 fold.

Affymetrix ST 1.0 Array ID	0-No	6-No	12-No	0-Yes	6-Yes	12-Yes

Supplemental Table 1. Gene expression values changed greater than 2 fold.

Affymetrix ST 1.0 Array ID	0-No	6-No	12-No	0-Yes	6-Yes	12-Yes

Supplemental Table 1. Gene expression values changed greater than 2 fold.

Affymetrix ST 1.0 Array ID	0-No	6-No	12-No	0-Yes	6-Yes	12-Yes

Supplemental Table 1. Gene expression values changed greater than 2 fold.

Affymetrix ST 1.0 Array ID	0-No	6-No	12-No	0-Yes	6-Yes	12-Yes

Supplemental Table 1. Gene expression values changed greater than 2 fold.

Affymetrix ST 1.0 Array ID	0-No	6-No	12-No	0-Yes	6-Yes	12-Yes

Supplemental Table 1. Gene expression values changed greater than 2 fold.

Affymetrix ST 1.0 Array ID	0-No	6-No	12-No	0-Yes	6-Yes	12-Yes

Supplemental Table 1. Gene expression values changed greater than 2 fold.

Affymetrix ST 1.0 Array ID	0-No	6-No	12-No	0-Yes	6-Yes	12-Yes

Supplemental Table 1. Gene expression values changed greater than 2 fold.

Affymetrix ST 1.0 Array ID	0-No	6-No	12-No	0-Yes	6-Yes	12-Yes

Supplemental Table 1. Gene expression values changed greater than 2 fold.

Affymetrix ST 1.0 Array ID	0-No	6-No	12-No	0-Yes	6-Yes	12-Yes

Supplemental Table 1. Gene expression values changed greater than 2 fold.

Affymetrix ST 1.0 Array ID	0-No	6-No	12-No	0-Yes	6-Yes	12-Yes

Supplemental Table 1. Gene expression values changed greater than 2 fold.

Affymetrix ST 1.0 Array ID	0-No	6-No	12-No	0-Yes	6-Yes	12-Yes

Supplemental Table 1. Gene expression values changed greater than 2 fold.

Affymetrix ST 1.0 Array ID	0-No	6-No	12-No	0-Yes	6-Yes	12-Yes

Supplemental Table 1. Gene expression values changed greater than 2 fold.

Affymetrix ST 1.0 Array ID	0-No	6-No	12-No	0-Yes	6-Yes	12-Yes

Supplemental Table 1. Gene expression values changed greater than 2 fold.

Affymetrix ST 1.0 Array ID	0-No	6-No	12-No	0-Yes	6-Yes	12-Yes

Supplemental Table 1. Gene expression values changed greater than 2 fold.

Affymetrix ST 1.0 Array ID	0-No	6-No	12-No	0-Yes	6-Yes	12-Yes

Supplemental Table 1. Gene expression values changed greater than 2 fold.

Affymetrix ST 1.0 Array ID	0-No	6-No	12-No	0-Yes	6-Yes	12-Yes

Supplemental Table 1. Gene expression values changed greater than 2 fold.

Affymetrix ST 1.0 Array ID	0-No	6-No	12-No	0-Yes	6-Yes	12-Yes

Supplemental Table 1. Gene expression values changed greater than 2 fold.

Affymetrix ST 1.0 Array ID	0-No	6-No	12-No	0-Yes	6-Yes	12-Yes

Supplemental Table 1. Gene expression values changed greater than 2 fold.

Affymetrix ST 1.0 Array ID	0-No	6-No	12-No	0-Yes	6-Yes	12-Yes

Supplemental Table 1. Gene expression values changed greater than 2 fold.

Affymetrix ST 1.0 Array ID	0-No	6-No	12-No	0-Yes	6-Yes	12-Yes

Supplemental Table 1. Gene expression values changed greater than 2 fold.

Affymetrix ST 1.0 Array ID	0-No	6-No	12-No	0-Yes	6-Yes	12-Yes

Supplemental Table 1. Gene expression values changed greater than 2 fold.

Affymetrix ST 1.0 Array ID	0-No	6-No	12-No	0-Yes	6-Yes	12-Yes

Supplemental Table 1. Gene expression values changed greater than 2 fold.

Affymetrix ST 1.0 Array ID	0-No	6-No	12-No	0-Yes	6-Yes	12-Yes

Supplemental Table 1. Gene expression values changed greater than 2 fold.

Affymetrix ST 1.0 Array ID	0-No	6-No	12-No	0-Yes	6-Yes	12-Yes

Supplemental Table 1. Gene expression values changed greater than 2 fold.

Affymetrix ST 1.0 Array ID	0-No	6-No	12-No	0-Yes	6-Yes	12-Yes

Supplemental Table 1. Gene expression values changed greater than 2 fold.

Affymetrix ST 1.0 Array ID	0-No	6-No	12-No	0-Yes	6-Yes	12-Yes

Supplemental Table 1. Gene expression values changed greater than 2 fold.

Affymetrix ST 1.0 Array ID	0-No	6-No	12-No	0-Yes	6-Yes	12-Yes

Supplemental Table 1. Gene expression values changed greater than 2 fold.

Affymetrix ST 1.0 Array ID	0-No	6-No	12-No	0-Yes	6-Yes	12-Yes

Supplemental Table 1. Gene expression values changed greater than 2 fold.

Affymetrix ST 1.0 Array ID	0-No	6-No	12-No	0-Yes	6-Yes	12-Yes

Supplemental Table 1. Gene expression values changed greater than 2 fold.

Affymetrix ST 1.0 Array ID	0-No	6-No	12-No	0-Yes	6-Yes	12-Yes

Supplemental Table 1. Gene expression values changed greater than 2 fold.

Affymetrix ST 1.0 Array ID	0-No	6-No	12-No	0-Yes	6-Yes	12-Yes

Supplemental Table 1. Gene expression values changed greater than 2 fold.

Affymetrix ST 1.0 Array ID	0-No	6-No	12-No	0-Yes	6-Yes	12-Yes

Supplemental Table 1. Gene expression values changed greater than 2 fold.

Affymetrix ST 1.0 Array ID	0-No	6-No	12-No	0-Yes	6-Yes	12-Yes

Supplemental Table 1. Gene expression values changed greater than 2 fold.

Affymetrix ST 1.0 Array ID	0-No	6-No	12-No	0-Yes	6-Yes	12-Yes

Supplemental Table 1. Gene expression values changed greater than 2 fold.

Affymetrix ST 1.0 Array ID	0-No	6-No	12-No	0-Yes	6-Yes	12-Yes

Supplemental Table 1. Gene expression values changed greater than 2 fold.

Affymetrix ST 1.0 Array ID	0-No	6-No	12-No	0-Yes	6-Yes	12-Yes

Supplemental Table 1. Gene expression values changed greater than 2 fold.

Affymetrix ST 1.0 Array ID	0-No	6-No	12-No	0-Yes	6-Yes	12-Yes

Supplemental Table 1. Gene expression values changed greater than 2 fold.

Affymetrix ST 1.0 Array ID	0-No	6-No	12-No	0-Yes	6-Yes	12-Yes

Supplemental Table 1. Gene expression values changed greater than 2 fold.

Affymetrix ST 1.0 Array ID	0-No	6-No	12-No	0-Yes	6-Yes	12-Yes

Supplemental Table 1. Gene expression values changed greater than 2 fold.

Affymetrix ST 1.0 Array ID	0-No	6-No	12-No	0-Yes	6-Yes	12-Yes

Supplemental Table 1. Gene expression values changed greater than 2 fold.

Affymetrix ST 1.0 Array ID	0-No	6-No	12-No	0-Yes	6-Yes	12-Yes

Supplemental Table 1. Gene expression values changed greater than 2 fold.

Affymetrix ST 1.0 Array ID	0-No	6-No	12-No	0-Yes	6-Yes	12-Yes

Supplemental Table 1. Gene expression values changed greater than 2 fold.

Affymetrix ST 1.0 Array ID	0-No	6-No	12-No	0-Yes	6-Yes	12-Yes

Supplemental Table 1. Gene expression values changed greater than 2 fold.

Affymetrix ST 1.0 Array ID	0-No	6-No	12-No	0-Yes	6-Yes	12-Yes

Supplemental Table 1. Gene expression values changed greater than 2 fold.

Affymetrix ST 1.0 Array ID	0-No	6-No	12-No	0-Yes	6-Yes	12-Yes

Supplemental Table 1. Gene expression values changed greater than 2 fold.

Affymetrix ST 1.0 Array ID	0-No	6-No	12-No	0-Yes	6-Yes	12-Yes

Supplemental Table 1. Gene expression values changed greater than 2 fold.

Affymetrix ST 1.0 Array ID	0-No	6-No	12-No	0-Yes	6-Yes	12-Yes

Supplemental Table 1. Gene expression values changed greater than 2 fold.

Affymetrix ST 1.0 Array ID	0-No	6-No	12-No	0-Yes	6-Yes	12-Yes

Supplemental Table 1. Gene expression values changed greater than 2 fold.

Affymetrix ST 1.0 Array ID	0-No	6-No	12-No	0-Yes	6-Yes	12-Yes

Supplemental Table 1. Gene expression values changed greater than 2 fold.

Affymetrix ST 1.0 Array ID	0-No	6-No	12-No	0-Yes	6-Yes	12-Yes

Supplemental Table 1. Gene expression values changed greater than 2 fold.

Affymetrix ST 1.0 Array ID	0-No	6-No	12-No	0-Yes	6-Yes	12-Yes

Supplemental Table 1. Gene expression values changed greater than 2 fold.

Affymetrix ST 1.0 Array ID	0-No	6-No	12-No	0-Yes	6-Yes	12-Yes

Supplemental Table 1. Gene expression values changed greater than 2 fold.

Affymetrix ST 1.0 Array ID	0-No	6-No	12-No	0-Yes	6-Yes	12-Yes

Supplemental Table 1. Gene expression values changed greater than 2 fold.

Affymetrix ST 1.0 Array ID	0-No	6-No	12-No	0-Yes	6-Yes	12-Yes

Supplemental Table 1. Gene expression values changed greater than 2 fold.

Affymetrix ST 1.0 Array ID	0-No	6-No	12-No	0-Yes	6-Yes	12-Yes

Supplemental Table 1. Gene expression values changed greater than 2 fold.

Affymetrix ST 1.0 Array ID	0-No	6-No	12-No	0-Yes	6-Yes	12-Yes

Supplemental Table 1. Gene expression values changed greater than 2 fold.

Affymetrix ST 1.0 Array ID	0-No	6-No	12-No	0-Yes	6-Yes	12-Yes

Supplemental Table 1. Gene expression values changed greater than 2 fold.

Affymetrix ST 1.0 Array ID	0-No	6-No	12-No	0-Yes	6-Yes	12-Yes

Supplemental Table 1. Gene expression values changed greater than 2 fold.

Affymetrix ST 1.0 Array ID	0-No	6-No	12-No	0-Yes	6-Yes	12-Yes

Supplemental Table 1. Gene expression values changed greater than 2 fold.

Affymetrix ST 1.0 Array ID	0-No	6-No	12-No	0-Yes	6-Yes	12-Yes

Supplemental Table 1. Gene expression values changed greater than 2 fold.

Affymetrix ST 1.0 Array ID	0-No	6-No	12-No	0-Yes	6-Yes	12-Yes

Supplemental Table 1. Gene expression values changed greater than 2 fold.

Affymetrix ST 1.0 Array ID	0-No	6-No	12-No	0-Yes	6-Yes	12-Yes

Supplemental Table 1. Gene expression values changed greater than 2 fold.

Affymetrix ST 1.0 Array ID	0-No	6-No	12-No	0-Yes	6-Yes	12-Yes

Supplemental Table 1. Gene expression values changed greater than 2 fold.

Affymetrix ST 1.0 Array ID	0-No	6-No	12-No	0-Yes	6-Yes	12-Yes

Supplemental Table 1. Gene expression values changed greater than 2 fold.

Affymetrix ST 1.0 Array ID	0-No	6-No	12-No	0-Yes	6-Yes	12-Yes

Supplemental Table 1. Gene expression values changed greater than 2 fold.

Affymetrix ST 1.0 Array ID	0-No	6-No	12-No	0-Yes	6-Yes	12-Yes

Supplemental Table 1. Gene expression values changed greater than 2 fold.

Affymetrix ST 1.0 Array ID	0-No	6-No	12-No	0-Yes	6-Yes	12-Yes

Supplemental Table 1. Gene expression values changed greater than 2 fold.

Affymetrix ST 1.0 Array ID	0-No	6-No	12-No	0-Yes	6-Yes	12-Yes

Supplemental Table 1. Gene expression values changed greater than 2 fold.

Affymetrix ST 1.0 Array ID	0-No	6-No	12-No	0-Yes	6-Yes	12-Yes

Supplemental Table 1. Gene expression values changed greater than 2 fold.

Affymetrix ST 1.0 Array ID	0-No	6-No	12-No	0-Yes	6-Yes	12-Yes

Supplemental Table 1. Gene expression values changed greater than 2 fold.

Affymetrix ST 1.0 Array ID	0-No	6-No	12-No	0-Yes	6-Yes	12-Yes

Supplemental Table 1. Gene expression values changed greater than 2 fold.

Affymetrix ST 1.0 Array ID	0-No	6-No	12-No	0-Yes	6-Yes	12-Yes

Supplemental Table 1. Gene expression values changed greater than 2 fold.

Affymetrix ST 1.0 Array ID	0-No	6-No	12-No	0-Yes	6-Yes	12-Yes

Supplemental Table 1. Gene expression values changed greater than 2 fold.

Affymetrix ST 1.0 Array ID	0-No	6-No	12-No	0-Yes	6-Yes	12-Yes

Supplemental Table 1. Gene expression values changed greater than 2 fold.

Affymetrix ST 1.0 Array ID	0-No	6-No	12-No	0-Yes	6-Yes	12-Yes

Supplemental Table 1. Gene expression values changed greater than 2 fold.

Affymetrix ST 1.0 Array ID	0-No	6-No	12-No	0-Yes	6-Yes	12-Yes

Supplemental Table 1. Gene expression values changed greater than 2 fold.

Affymetrix ST 1.0 Array ID	0-No	6-No	12-No	0-Yes	6-Yes	12-Yes

Supplemental Table 1. Gene expression values changed greater than 2 fold.

Affymetrix ST 1.0 Array ID	0-No	6-No	12-No	0-Yes	6-Yes	12-Yes

Supplemental Table 1. Gene expression values changed greater than 2 fold.

Affymetrix ST 1.0 Array ID	0-No	6-No	12-No	0-Yes	6-Yes	12-Yes

Supplemental Table 1. Gene expression values changed greater than 2 fold.

Affymetrix ST 1.0 Array ID	0-No	6-No	12-No	0-Yes	6-Yes	12-Yes

Supplemental Table 1. Gene expression values changed greater than 2 fold.

Affymetrix ST 1.0 Array ID	0-No	6-No	12-No	0-Yes	6-Yes	12-Yes

Supplemental Table 1. Gene expression values changed greater than 2 fold.

Affymetrix ST 1.0 Array ID	0-No	6-No	12-No	0-Yes	6-Yes	12-Yes

Supplemental Table 1. Gene expression values changed greater than 2 fold.

Affymetrix ST 1.0 Array ID	0-No	6-No	12-No	0-Yes	6-Yes	12-Yes

Supplemental Table 1. Gene expression values changed greater than 2 fold.

Affymetrix ST 1.0 Array ID	0-No	6-No	12-No	0-Yes	6-Yes	12-Yes

Supplemental Table 1. Gene expression values changed greater than 2 fold.

Affymetrix ST 1.0 Array ID	0-No	6-No	12-No	0-Yes	6-Yes	12-Yes

Supplemental Table 1. Gene expression values changed greater than 2 fold.

Affymetrix ST 1.0 Array ID	0-No	6-No	12-No	0-Yes	6-Yes	12-Yes

Supplemental Table 1. Gene expression values changed greater than 2 fold.

Affymetrix ST 1.0 Array ID	0-No	6-No	12-No	0-Yes	6-Yes	12-Yes

Supplemental Table 1. Gene expression values changed greater than 2 fold.

Affymetrix ST 1.0 Array ID	0-No	6-No	12-No	0-Yes	6-Yes	12-Yes

Supplemental Table 1. Gene expression values changed greater than 2 fold.

Affymetrix ST 1.0 Array ID	0-No	6-No	12-No	0-Yes	6-Yes	12-Yes

Supplemental Table 1. Gene expression values changed greater than 2 fold.

Affymetrix ST 1.0 Array ID	0-No	6-No	12-No	0-Yes	6-Yes	12-Yes

Supplemental Table 1. Gene expression values changed greater than 2 fold.

Affymetrix ST 1.0 Array ID	0-No	6-No	12-No	0-Yes	6-Yes	12-Yes

Supplemental Table 1. Gene expression values changed greater than 2 fold.

Affymetrix ST 1.0 Array ID	0-No	6-No	12-No	0-Yes	6-Yes	12-Yes

Supplemental Table 1. Gene expression values changed greater than 2 fold.

Affymetrix ST 1.0 Array ID	0-No	6-No	12-No	0-Yes	6-Yes	12-Yes

Supplemental Table 1. Gene expression values changed greater than 2 fold.

Affymetrix ST 1.0 Array ID	0-No	6-No	12-No	0-Yes	6-Yes	12-Yes

Supplemental Table 1. Gene expression values changed greater than 2 fold.

Affymetrix ST 1.0 Array ID	0-No	6-No	12-No	0-Yes	6-Yes	12-Yes

Supplemental Table 1. Gene expression values changed greater than 2 fold.

Affymetrix ST 1.0 Array ID	0-No	6-No	12-No	0-Yes	6-Yes	12-Yes

Supplemental Table 1. Gene expression values changed greater than 2 fold.

Affymetrix ST 1.0 Array ID	0-No	6-No	12-No	0-Yes	6-Yes	12-Yes

Supplemental Table 1. Gene expression values changed greater than 2 fold.

Affymetrix ST 1.0 Array ID	0-No	6-No	12-No	0-Yes	6-Yes	12-Yes

Supplemental Table 1. Gene expression values changed greater than 2 fold.

Affymetrix ST 1.0 Array ID	0-No	6-No	12-No	0-Yes	6-Yes	12-Yes

Supplemental Table 1. Gene expression values changed greater than 2 fold.

Affymetrix ST 1.0 Array ID	0-No	6-No	12-No	0-Yes	6-Yes	12-Yes

Supplemental Table 1. Gene expression values changed greater than 2 fold.

Affymetrix ST 1.0 Array ID	0-No	6-No	12-No	0-Yes	6-Yes	12-Yes

Supplemental Table 1. Gene expression values changed greater than 2 fold.

Affymetrix ST 1.0 Array ID	0-No	6-No	12-No	0-Yes	6-Yes	12-Yes

Supplemental Table 1. Gene expression values changed greater than 2 fold.

Affymetrix ST 1.0 Array ID	0-No	6-No	12-No	0-Yes	6-Yes	12-Yes

Supplemental Table 1. Gene expression values changed greater than 2 fold.

Affymetrix ST 1.0 Array ID	0-No	6-No	12-No	0-Yes	6-Yes	12-Yes

Supplemental Table 1. Gene expression values changed greater than 2 fold.

Affymetrix ST 1.0 Array ID	0-No	6-No	12-No	0-Yes	6-Yes	12-Yes

Supplemental Table 1. Gene expression values changed greater than 2 fold.

Affymetrix ST 1.0 Array ID	0-No	6-No	12-No	0-Yes	6-Yes	12-Yes

Supplemental Table 1. Gene expression values changed greater than 2 fold.

Affymetrix ST 1.0 Array ID	0-No	6-No	12-No	0-Yes	6-Yes	12-Yes

Supplemental Table 1. Gene expression values changed greater than 2 fold.

Affymetrix ST 1.0 Array ID	0-No	6-No	12-No	0-Yes	6-Yes	12-Yes

Supplemental Table 1. Gene expression values changed greater than 2 fold.

Affymetrix ST 1.0 Array ID	0-No	6-No	12-No	0-Yes	6-Yes	12-Yes

Supplemental Table 1. Gene expression values changed greater than 2 fold.

Affymetrix ST 1.0 Array ID	0-No	6-No	12-No	0-Yes	6-Yes	12-Yes

Supplemental Table 1. Gene expression values changed greater than 2 fold.

Affymetrix ST 1.0 Array ID	0-No	6-No	12-No	0-Yes	6-Yes	12-Yes

Supplemental Table 1. Gene expression values changed greater than 2 fold.

Affymetrix ST 1.0 Array ID	0-No	6-No	12-No	0-Yes	6-Yes	12-Yes

Supplemental Table 1. Gene expression values changed greater than 2 fold.

Affymetrix ST 1.0 Array ID	0-No	6-No	12-No	0-Yes	6-Yes	12-Yes

Supplemental Table 1. Gene expression values changed greater than 2 fold.

Affymetrix ST 1.0 Array ID	0-No	6-No	12-No	0-Yes	6-Yes	12-Yes

Supplemental Table 1. Gene expression values changed greater than 2 fold.

Affymetrix ST 1.0 Array ID	0-No	6-No	12-No	0-Yes	6-Yes	12-Yes

Supplemental Table 1. Gene expression values changed greater than 2 fold.

Affymetrix ST 1.0 Array ID	0-No	6-No	12-No	0-Yes	6-Yes	12-Yes

Supplemental Table 1. Gene expression values changed greater than 2 fold.

Affymetrix ST 1.0 Array ID	0-No	6-No	12-No	0-Yes	6-Yes	12-Yes

Supplemental Table 1. Gene expression values changed greater than 2 fold.

Affymetrix ST 1.0 Array ID	0-No	6-No	12-No	0-Yes	6-Yes	12-Yes

Supplemental Table 1. Gene expression values changed greater than 2 fold.

Affymetrix ST 1.0 Array ID	0-No	6-No	12-No	0-Yes	6-Yes	12-Yes

Supplemental Table 1. Gene expression values changed greater than 2 fold.

Affymetrix ST 1.0 Array ID	0-No	6-No	12-No	0-Yes	6-Yes	12-Yes

Supplemental Table 1. Gene expression values changed greater than 2 fold.

Affymetrix ST 1.0 Array ID	0-No	6-No	12-No	0-Yes	6-Yes	12-Yes

Supplemental Table 1. Gene expression values changed greater than 2 fold.

Affymetrix ST 1.0 Array ID	0-No	6-No	12-No	0-Yes	6-Yes	12-Yes

Supplemental Table 1. Gene expression values changed greater than 2 fold.

Affymetrix ST 1.0 Array ID	0-No	6-No	12-No	0-Yes	6-Yes	12-Yes

Supplemental Table 1. Gene expression values changed greater than 2 fold.

Affymetrix ST 1.0 Array ID	0-No	6-No	12-No	0-Yes	6-Yes	12-Yes

Supplemental Table 1. Gene expression values changed greater than 2 fold.

Affymetrix ST 1.0 Array ID	0-No	6-No	12-No	0-Yes	6-Yes	12-Yes

Supplemental Table 1. Gene expression values changed greater than 2 fold.

Affymetrix ST 1.0 Array ID	0-No	6-No	12-No	0-Yes	6-Yes	12-Yes

Supplemental Table 1. Gene expression values changed greater than 2 fold.

Affymetrix ST 1.0 Array ID	0-No	6-No	12-No	0-Yes	6-Yes	12-Yes

Supplemental Table 1. Gene expression values changed greater than 2 fold.

Affymetrix ST 1.0 Array ID	0-No	6-No	12-No	0-Yes	6-Yes	12-Yes

Supplemental Table 1. Gene expression values changed greater than 2 fold.

Affymetrix ST 1.0 Array ID	0-No	6-No	12-No	0-Yes	6-Yes	12-Yes

Supplemental Table 1. Gene expression values changed greater than 2 fold.

Affymetrix ST 1.0 Array ID	0-No	6-No	12-No	0-Yes	6-Yes	12-Yes

Supplemental Table 1. Gene expression values changed greater than 2 fold.

Affymetrix ST 1.0 Array ID	0-No	6-No	12-No	0-Yes	6-Yes	12-Yes

Supplemental Table 1. Gene expression values changed greater than 2 fold.

Affymetrix ST 1.0 Array ID	0-No	6-No	12-No	0-Yes	6-Yes	12-Yes

Supplemental Table 1. Gene expression values changed greater than 2 fold.

Affymetrix ST 1.0 Array ID	0-No	6-No	12-No	0-Yes	6-Yes	12-Yes

Supplemental Table 1. Gene expression values changed greater than 2 fold.

Affymetrix ST 1.0 Array ID	0-No	6-No	12-No	0-Yes	6-Yes	12-Yes

Supplemental Table 1. Gene expression values changed greater than 2 fold.

Affymetrix ST 1.0 Array ID	0-No	6-No	12-No	0-Yes	6-Yes	12-Yes

Supplemental Table 1. Gene expression values changed greater than 2 fold.

Affymetrix ST 1.0 Array ID	0-No	6-No	12-No	0-Yes	6-Yes	12-Yes

Supplemental Table 1. Gene expression values changed greater than 2 fold.

Affymetrix ST 1.0 Array ID	0-No	6-No	12-No	0-Yes	6-Yes	12-Yes

Supplemental Table 1. Gene expression values changed greater than 2 fold.

Affymetrix ST 1.0 Array ID	0-No	6-No	12-No	0-Yes	6-Yes	12-Yes

Supplemental Table 1. Gene expression values changed greater than 2 fold.

Affymetrix ST 1.0 Array ID	0-No	6-No	12-No	0-Yes	6-Yes	12-Yes

Supplemental Table 1. Gene expression values changed greater than 2 fold.

Affymetrix ST 1.0 Array ID	0-No	6-No	12-No	0-Yes	6-Yes	12-Yes

Supplemental Table 1. Gene expression values changed greater than 2 fold.

Affymetrix ST 1.0 Array ID	0-No	6-No	12-No	0-Yes	6-Yes	12-Yes

Supplemental Table 1. Gene expression values changed greater than 2 fold.

Affymetrix ST 1.0 Array ID	0-No	6-No	12-No	0-Yes	6-Yes	12-Yes

Supplemental Table 1. Gene expression values changed greater than 2 fold.

Affymetrix ST 1.0 Array ID	0-No	6-No	12-No	0-Yes	6-Yes	12-Yes

Supplemental Table 1. Gene expression values changed greater than 2 fold.

Affymetrix ST 1.0 Array ID	0-No	6-No	12-No	0-Yes	6-Yes	12-Yes

Supplemental Table 1. Gene expression values changed greater than 2 fold.

Affymetrix ST 1.0 Array ID	0-No	6-No	12-No	0-Yes	6-Yes	12-Yes

Supplemental Table 1. Gene expression values changed greater than 2 fold.

Affymetrix ST 1.0 Array ID	0-No	6-No	12-No	0-Yes	6-Yes	12-Yes

Supplemental Table 1. Gene expression values changed greater than 2 fold.

Affymetrix ST 1.0 Array ID	0-No	6-No	12-No	0-Yes	6-Yes	12-Yes

Supplemental Table 1. Gene expression values changed greater than 2 fold.

Affymetrix ST 1.0 Array ID	0-No	6-No	12-No	0-Yes	6-Yes	12-Yes

Supplemental Table 1. Gene expression values changed greater than 2 fold.

Affymetrix ST 1.0 Array ID	0-No	6-No	12-No	0-Yes	6-Yes	12-Yes

Supplemental Table 1. Gene expression values changed greater than 2 fold.

Affymetrix ST 1.0 Array ID	0-No	6-No	12-No	0-Yes	6-Yes	12-Yes

Supplemental Table 1. Gene expression values changed greater than 2 fold.

Affymetrix ST 1.0 Array ID	0-No	6-No	12-No	0-Yes	6-Yes	12-Yes

Supplemental Table 1. Gene expression values changed greater than 2 fold.

Affymetrix ST 1.0 Array ID	0-No	6-No	12-No	0-Yes	6-Yes	12-Yes

Supplemental Table 1. Gene expression values changed greater than 2 fold.

Affymetrix ST 1.0 Array ID	0-No	6-No	12-No	0-Yes	6-Yes	12-Yes

Supplemental Table 1. Gene expression values changed greater than 2 fold.

Affymetrix ST 1.0 Array ID	0-No	6-No	12-No	0-Yes	6-Yes	12-Yes

Supplemental Table 1. Gene expression values changed greater than 2 fold.

Affymetrix ST 1.0 Array ID	0-No	6-No	12-No	0-Yes	6-Yes	12-Yes

Supplemental Table 1. Gene expression values changed greater than 2 fold.

Affymetrix ST 1.0 Array ID	0-No	6-No	12-No	0-Yes	6-Yes	12-Yes

Supplemental Table 1. Gene expression values changed greater than 2 fold.

Affymetrix ST 1.0 Array ID	0-No	6-No	12-No	0-Yes	6-Yes	12-Yes

Supplemental Table 1. Gene expression values changed greater than 2 fold.

Affymetrix ST 1.0 Array ID	0-No	6-No	12-No	0-Yes	6-Yes	12-Yes

Supplemental Table 1. Gene expression values changed greater than 2 fold.

Affymetrix ST 1.0 Array ID	0-No	6-No	12-No	0-Yes	6-Yes	12-Yes

Supplemental Table 1. Gene expression values changed greater than 2 fold.

Affymetrix ST 1.0 Array ID	0-No	6-No	12-No	0-Yes	6-Yes	12-Yes

Supplemental Table 1. Gene expression values changed greater than 2 fold.

Affymetrix ST 1.0 Array ID	0-No	6-No	12-No	0-Yes	6-Yes	12-Yes

Supplemental Table 1. Gene expression values changed greater than 2 fold.

Affymetrix ST 1.0 Array ID	0-No	6-No	12-No	0-Yes	6-Yes	12-Yes

Supplemental Table 1. Gene expression values changed greater than 2 fold.

Affymetrix ST 1.0 Array ID	0-No	6-No	12-No	0-Yes	6-Yes	12-Yes

Supplemental Table 1. Gene expression values changed greater than 2 fold.

Affymetrix ST 1.0 Array ID	0-No	6-No	12-No	0-Yes	6-Yes	12-Yes

Supplemental Table 1. Gene expression values changed greater than 2 fold.

Affymetrix ST 1.0 Array ID	0-No	6-No	12-No	0-Yes	6-Yes	12-Yes

Supplemental Table 1. Gene expression values changed greater than 2 fold.

Affymetrix ST 1.0 Array ID	0-No	6-No	12-No	0-Yes	6-Yes	12-Yes

Supplemental Table 1. Gene expression values changed greater than 2 fold.

Affymetrix ST 1.0 Array ID	0-No	6-No	12-No	0-Yes	6-Yes	12-Yes

Supplemental Table 1. Gene expression values changed greater than 2 fold.

Affymetrix ST 1.0 Array ID	0-No	6-No	12-No	0-Yes	6-Yes	12-Yes

Supplemental Table 1. Gene expression values changed greater than 2 fold.

Affymetrix ST 1.0 Array ID	0-No	6-No	12-No	0-Yes	6-Yes	12-Yes

Supplemental Table 1. Gene expression values changed greater than 2 fold.

Affymetrix ST 1.0 Array ID	0-No	6-No	12-No	0-Yes	6-Yes	12-Yes

Supplemental Table 1. Gene expression values changed greater than 2 fold.

Affymetrix ST 1.0 Array ID	0-No	6-No	12-No	0-Yes	6-Yes	12-Yes

Supplemental Table 1. Gene expression values changed greater than 2 fold.

Affymetrix ST 1.0 Array ID	0-No	6-No	12-No	0-Yes	6-Yes	12-Yes

Supplemental Table 1. Gene expression values changed greater than 2 fold.

Affymetrix ST 1.0 Array ID	0-No	6-No	12-No	0-Yes	6-Yes	12-Yes

Supplemental Table 1. Gene expression values changed greater than 2 fold.

Affymetrix ST 1.0 Array ID	0-No	6-No	12-No	0-Yes	6-Yes	12-Yes

Supplemental Table 1. Gene expression values changed greater than 2 fold.

Affymetrix ST 1.0 Array ID	0-No	6-No	12-No	0-Yes	6-Yes	12-Yes

Supplemental Table 1. Gene expression values changed greater than 2 fold.

Affymetrix ST 1.0 Array ID	0-No	6-No	12-No	0-Yes	6-Yes	12-Yes

Supplemental Table 1. Gene expression values changed greater than 2 fold.

Affymetrix ST 1.0 Array ID	0-No	6-No	12-No	0-Yes	6-Yes	12-Yes

Supplemental Table 1. Gene expression values changed greater than 2 fold.

Affymetrix ST 1.0 Array ID	0-No	6-No	12-No	0-Yes	6-Yes	12-Yes

Supplemental Table 1. Gene expression values changed greater than 2 fold.

Affymetrix ST 1.0 Array ID	0-No	6-No	12-No	0-Yes	6-Yes	12-Yes

Supplemental Table 1. Gene expression values changed greater than 2 fold.

Affymetrix ST 1.0 Array ID	0-No	6-No	12-No	0-Yes	6-Yes	12-Yes

Supplemental Table 1. Gene expression values changed greater than 2 fold.

Affymetrix ST 1.0 Array ID	0-No	6-No	12-No	0-Yes	6-Yes	12-Yes

Supplemental Table 1. Gene expression values changed greater than 2 fold.

Affymetrix ST 1.0 Array ID	0-No	6-No	12-No	0-Yes	6-Yes	12-Yes

Supplemental Table 1. Gene expression values changed greater than 2 fold.

Affymetrix ST 1.0 Array ID	0-No	6-No	12-No	0-Yes	6-Yes	12-Yes

Supplemental Table 1. Gene expression values changed greater than 2 fold.

Affymetrix ST 1.0 Array ID	0-No	6-No	12-No	0-Yes	6-Yes	12-Yes

Supplemental Table 1. Gene expression values changed greater than 2 fold.

Affymetrix ST 1.0 Array ID	0-No	6-No	12-No	0-Yes	6-Yes	12-Yes

Supplemental Table 1. Gene expression values changed greater than 2 fold.

Affymetrix ST 1.0 Array ID	0-No	6-No	12-No	0-Yes	6-Yes	12-Yes

Supplemental Table 1. Gene expression values changed greater than 2 fold.

Affymetrix ST 1.0 Array ID	0-No	6-No	12-No	0-Yes	6-Yes	12-Yes

Supplemental Table 1. Gene expression values changed greater than 2 fold.

Affymetrix ST 1.0 Array ID	0-No	6-No	12-No	0-Yes	6-Yes	12-Yes

Supplemental Table 1. Gene expression values changed greater than 2 fold.

Affymetrix ST 1.0 Array ID	0-No	6-No	12-No	0-Yes	6-Yes	12-Yes

Supplemental Table 1. Gene expression values changed greater than 2 fold.

Affymetrix ST 1.0 Array ID	0-No	6-No	12-No	0-Yes	6-Yes	12-Yes

Supplemental Table 1. Gene expression values changed greater than 2 fold.

Affymetrix ST 1.0 Array ID	0-No	6-No	12-No	0-Yes	6-Yes	12-Yes

Supplemental Table 1. Gene expression values changed greater than 2 fold.

Affymetrix ST 1.0 Array ID	0-No	6-No	12-No	0-Yes	6-Yes	12-Yes

Supplemental Table 1. Gene expression values changed greater than 2 fold.

Affymetrix ST 1.0 Array ID	0-No	6-No	12-No	0-Yes	6-Yes	12-Yes

Supplemental Table 1. Gene expression values changed greater than 2 fold.

Affymetrix ST 1.0 Array ID	0-No	6-No	12-No	0-Yes	6-Yes	12-Yes

Supplemental Table 1. Gene expression values changed greater than 2 fold.

Affymetrix ST 1.0 Array ID	0-No	6-No	12-No	0-Yes	6-Yes	12-Yes

Supplemental Table 1. Gene expression values changed greater than 2 fold.

Affymetrix ST 1.0 Array ID	0-No	6-No	12-No	0-Yes	6-Yes	12-Yes

Supplemental Table 1. Gene expression values changed greater than 2 fold.

Affymetrix ST 1.0 Array ID	0-No	6-No	12-No	0-Yes	6-Yes	12-Yes

Supplemental Table 1. Gene expression values changed greater than 2 fold.

Affymetrix ST 1.0 Array ID	0-No	6-No	12-No	0-Yes	6-Yes	12-Yes

Supplemental Table 1. Gene expression values changed greater than 2 fold.

Affymetrix ST 1.0 Array ID	0-No	6-No	12-No	0-Yes	6-Yes	12-Yes

Supplemental Table 1. Gene expression values changed greater than 2 fold.

Affymetrix ST 1.0 Array ID	0-No	6-No	12-No	0-Yes	6-Yes	12-Yes

Supplemental Table 1. Gene expression values changed greater than 2 fold.

Affymetrix ST 1.0 Array ID	0-No	6-No	12-No	0-Yes	6-Yes	12-Yes

Supplemental Table 1. Gene expression values changed greater than 2 fold.

Affymetrix ST 1.0 Array ID	0-No	6-No	12-No	0-Yes	6-Yes	12-Yes

Supplemental Table 1. Gene expression values changed greater than 2 fold.

Affymetrix ST 1.0 Array ID	0-No	6-No	12-No	0-Yes	6-Yes	12-Yes

Supplemental Table 1. Gene expression values changed greater than 2 fold.

Affymetrix ST 1.0 Array ID	0-No	6-No	12-No	0-Yes	6-Yes	12-Yes

Supplemental Table 1. Gene expression values changed greater than 2 fold.

Affymetrix ST 1.0 Array ID	0-No	6-No	12-No	0-Yes	6-Yes	12-Yes

Supplemental Table 1. Gene expression values changed greater than 2 fold.

Affymetrix ST 1.0 Array ID	0-No	6-No	12-No	0-Yes	6-Yes	12-Yes

Supplemental Table 1. Gene expression values changed greater than 2 fold.

Affymetrix ST 1.0 Array ID	0-No	6-No	12-No	0-Yes	6-Yes	12-Yes

Supplemental Table 1. Gene expression values changed greater than 2 fold.

Affymetrix ST 1.0 Array ID	0-No	6-No	12-No	0-Yes	6-Yes	12-Yes
