# Supplementary material for: Reduction in Inflammatory Gene Expression in Skeletal Muscle from Roux-en-Y Gastric Bypass Patients Randomized to Omentectomy
Source: PLoS One. 2011 Dec 16;6(12):e28577. doi: 10.1371/journal.pone.0028577 (PMC3241684; doi:10.1371/journal.pone.0028577)
Supplement: Table S4 — Average gene expression values for those molecular interaction members within the indicated molecular pathway. Pathways are ranked highest to lowest in terms of the number of genes detected per pathway. (PDF) [file pone.0028577.s006.pdf]

Supplemental Table 4. Array-revealed Average Gene Expression Values for Members of Various Molecular Pathways

Number of Genes in Pathway	Average (No Omentectomy; 6 vs. 0 Mo)	Average (Yes Omentectomy; 6 vs. 0 Mo)	Average (No Omentectomy; 12 vs. 0 Mo)	Average (Yes Omentectomy; 12 vs. 0 Mo)	Pathway Number	Pathway
137	9.14E-01	6.97E-01	7.59E-01	5.90E-01	86	Metabolic pathways
43	9.20E-01	1.07E+00	8.42E-01	8.58E-01	105	Cytokine-cytokine receptor interaction
42	1.05E+00	1.04E+00	8.84E-01	8.17E-01	102	MAPK signaling pathway
38	8.89E-01	1.02E+00	7.71E-01	7.92E-01	106	Chemokine signaling pathway
36	8.99E-01	7.61E-01	8.78E-01	4.48E-01	93	Spliceosome
30	1.01E+00	1.04E+00	8.46E-01	6.78E-01	109	Cell cycle
29	9.83E-01	8.91E-01	7.71E-01	5.96E-01	131	Focal adhesion
29	1.12E+00	7.21E-01	8.07E-01	5.76E-01	118	Phagosome
27	6.54E-01	8.19E-01	9.37E-01	8.10E-01	88	Ribosome
27	8.18E-01	6.45E-01	6.88E-01	4.45E-01	115	Protein processing in endoplasmic reticulum
26	1.02E+00	9.04E-01	8.51E-01	5.26E-01	112	Ubiquitin mediated proteolysis
26	8.36E-01	5.49E-01	8.07E-01	4.90E-01	157	Olfactory transduction
25	8.93E-01	1.20E+00	7.78E-01	9.39E-01	144	Jak-STAT signaling pathway
24	9.62E-01	9.08E-01	6.10E-01	4.67E-01	117	Endocytosis
24	1.09E+00	8.91E-01	9.23E-01	6.10E-01	124	Wnt signaling pathway
24	8.55E-01	8.13E-01	6.58E-01	5.04E-01	160	Regulation of actin cytoskeleton
24	9.86E-01	7.11E-01	7.76E-01	5.69E-01	104	Calcium signaling pathway
22	8.33E-01	6.23E-01	5.48E-01	5.41E-01	190	Toxoplasmosis
21	9.58E-01	1.18E+00	8.80E-01	9.91E-01	140	Toll-like receptor signaling pathway
20	1.01E+00	1.07E+00	6.96E-01	7.26E-01	161	Insulin signaling pathway
20	8.59E-01	1.00E+00	7.24E-01	8.32E-01	191	Amoebiasis
20	6.18E-01	7.68E-01	8.63E-01	7.26E-01	178	Parkinson's disease
20	8.36E-01	7.22E-01	9.30E-01	6.61E-01	177	Alzheimer's disease
20	8.02E-01	6.40E-01	9.49E-01	5.70E-01	16	Oxidative phosphorylation
19	1.24E+00	1.15E+00	9.73E-01	7.86E-01	128	TGF-beta signaling pathway
19	7.88E-01	7.85E-01	8.57E-01	7.47E-01	180	Huntington's disease
19	9.64E-01	7.75E-01	9.79E-01	5.14E-01	110	Oocyte meiosis
19	8.83E-01	7.69E-01	7.36E-01	5.49E-01	17	Purine metabolism
19	8.45E-01	7.31E-01	6.83E-01	4.37E-01	135	Tight junction
19	9.66E-01	7.18E-01	7.45E-01	5.82E-01	175	Pancreatic secretion
19	1.13E+00	7.17E-01	8.89E-01	4.41E-01	123	Vascular smooth muscle contraction
18	7.13E-01	8.21E-01	4.81E-01	5.85E-01	151	Leukocyte transendothelial migration
18	8.53E-01	7.33E-01	6.91E-01	5.18E-01	155	Neurotrophin signaling pathway
18	9.09E-01	5.87E-01	8.15E-01	4.06E-01	108	Neuroactive ligand-receptor interaction
17	1.15E+00	1.64E+00	1.08E+00	1.37E+00	188	Chagas disease
17	9.31E-01	8.29E-01	7.33E-01	5.54E-01	184	Epithelial cell signaling in Helicobacter pylori infection
17	9.02E-01	7.05E-01	7.09E-01	5.47E-01	107	Phosphatidylinositol signaling system
17	7.78E-01	5.49E-01	7.84E-01	4.91E-01	209	Systemic lupus erythematosus
16	1.01E+00	9.96E-01	8.22E-01	5.38E-01	89	RNA degradation
16	1.05E+00	9.14E-01	7.64E-01	5.83E-01	129	Axon guidance
16	1.10E+00	8.44E-01	7.67E-01	6.57E-01	173	Salivary secretion
16	8.73E-01	7.83E-01	7.85E-01	6.71E-01	133	Cell adhesion molecules (CAMs)
16	1.00E+00	7.17E-01	8.60E-01	5.44E-01	162	GnRH signaling pathway
16	9.75E-01	6.75E-01	8.64E-01	4.24E-01	163	Progesterone-mediated oocyte maturation
15	1.10E+00	1.70E+00	1.09E+00	1.69E+00	189	Malaria
15	1.17E+00	1.58E+00	1.06E+00	1.32E+00	141	NOD-like receptor signaling pathway
15	1.15E+00	1.14E+00	9.53E-01	9.78E-01	213	Hypertrophic cardiomyopathy (HCM)
15	1.01E+00	1.07E+00	8.10E-01	8.19E-01	103	ErbB signaling pathway
15	9.86E-01	9.38E-01	9.20E-01	7.29E-01	147	T cell receptor signaling pathway
15	8.68E-01	8.62E-01	7.59E-01	7.30E-01	96	PPAR signaling pathway
15	8.38E-01	6.76E-01	8.95E-01	5.02E-01	119	Peroxisome
15	9.53E-01	6.55E-01	8.99E-01	6.00E-01	81	Aminoacyl-tRNA biosynthesis
15	8.74E-01	6.46E-01	5.78E-01	4.95E-01	150	Fc gamma R-mediated phagocytosis
14	9.94E-01	1.38E+00	7.73E-01	1.08E+00	111	p53 signaling pathway
14	9.74E-01	8.72E-01	8.50E-01	7.32E-01	174	Gastric acid secretion
14	9.14E-01	8.56E-01	6.31E-01	5.65E-01	214	Arrhythmogenic right ventricular cardiomyopathy (ARVC)
14	1.02E+00	8.14E-01	8.49E-01	6.64E-01	215	Dilated cardiomyopathy
14	8.53E-01	7.73E-01	7.78E-01	6.14E-01	146	Natural killer cell mediated cytotoxicity
14	9.47E-01	7.47E-01	7.98E-01	7.29E-01	116	Lysosome

Supplemental Table 4. Array-revealed Average Gene Expression Values for Members of Various Molecular Pathways

Number of Genes in Pathway	Average (No Omentectomy; 6 vs. 0 Mo)	Average (Yes Omentectomy; 6 vs. 0 Mo)	Average (No Omentectomy; 12 vs. 0 Mo)	Average (Yes Omentectomy; 12 vs. 0 Mo)	Pathway Number	Pathway
14	9.78E-01	7.26E-01	8.47E-01	4.73E-01	164	Melanogenesis
13	1.19E+00	1.09E+00	9.69E-01	7.07E-01	132	ECM-receptor interaction
13	1.01E+00	8.89E-01	8.71E-01	8.39E-01	187	Leishmaniasis
13	7.48E-01	8.12E-01	7.23E-01	5.75E-01	194	Renal cell carcinoma
12	7.92E-01	1.02E+00	8.38E-01	9.49E-01	122	Cardiac muscle contraction
12	1.04E+00	9.51E-01	9.34E-01	7.27E-01	148	B cell receptor signaling pathway
12	9.02E-01	8.57E-01	7.57E-01	6.59E-01	19	Pyrimidine metabolism
12	7.83E-01	8.52E-01	7.44E-01	5.33E-01	98	Nucleotide excision repair
12	9.57E-01	8.44E-01	8.04E-01	6.22E-01	197	Glioma
12	9.54E-01	7.59E-01	7.55E-01	4.29E-01	198	Prostate cancer
12	9.74E-01	7.44E-01	7.10E-01	5.29E-01	52	Inositol phosphate metabolism
12	8.41E-01	6.62E-01	9.50E-01	4.67E-01	216	Viral myocarditis
12	8.87E-01	6.18E-01	5.93E-01	4.68E-01	149	Fc epsilon RI signaling pathway
12	1.12E+00	6.15E-01	8.82E-01	5.69E-01	54	Glycerophospholipid metabolism
12	1.12E+00	5.93E-01	7.24E-01	3.99E-01	156	Long-term depression
12	9.55E-01	5.63E-01	8.00E-01	4.35E-01	183	Vibrio cholerae infection
12	1.11E+00	4.04E-01	1.02E+00	3.71E-01	138	Antigen processing and presentation
11	9.20E-01	1.16E+00	6.36E-01	8.49E-01	166	Type II diabetes mellitus
11	8.74E-01	1.09E+00	8.93E-01	1.11E+00	145	Hematopoietic cell lineage
11	9.68E-01	1.05E+00	6.99E-01	6.81E-01	186	Shigellosis
11	8.84E-01	9.20E-01	6.49E-01	6.44E-01	204	Acute myeloid leukemia
11	1.07E+00	8.55E-01	8.49E-01	4.38E-01	136	Gap junction
11	7.55E-01	7.89E-01	6.00E-01	2.83E-01	134	Adherens junction
11	8.81E-01	7.44E-01	6.45E-01	3.83E-01	195	Pancreatic cancer
11	7.94E-01	6.93E-01	6.42E-01	6.32E-01	130	VEGF signaling pathway
11	7.34E-01	5.14E-01	9.64E-01	5.10E-01	94	Proteasome
10	1.14E+00	1.13E+00	8.39E-01	9.08E-01	165	Adipocytokine signaling pathway
10	8.47E-01	1.13E+00	7.29E-01	1.04E+00	137	Complement and coagulation cascades
10	9.65E-01	1.05E+00	7.84E-01	7.78E-01	203	Chronic myeloid leukemia
10	5.95E-01	8.38E-01	4.96E-01	7.29E-01	121	Apoptosis
10	9.15E-01	8.04E-01	7.19E-01	4.59E-01	142	RIG-I-like receptor signaling pathway
9	1.19E+00	1.46E+00	1.15E+00	1.18E+00	193	Colorectal cancer
9	1.11E+00	6.94E-01	9.44E-01	4.68E-01	154	Long-term potentiation
9	8.31E-01	8.45E-01	6.20E-01	5.28E-01	201	Melanoma
9	8.25E-01	7.49E-01	6.29E-01	5.75E-01	169	Aldosterone-regulated sodium reabsorption
9	6.99E-01	8.96E-01	7.35E-01	8.47E-01	205	Small cell lung cancer
8	1.06E+00	8.96E-01	7.96E-01	7.39E-01	83	Drug metabolism - cytochrome P450
8	9.91E-01	8.99E-01	7.68E-01	4.61E-01	87	ABC transporters
8	8.93E-01	8.57E-01	6.23E-01	4.76E-01	185	Pathogenic Escherichia coli infection
8	8.76E-01	6.89E-01	6.82E-01	4.63E-01	120	mTOR signaling pathway
8	8.53E-01	7.08E-01	6.34E-01	4.06E-01	206	Non-small cell lung cancer
8	8.50E-01	8.40E-01	6.78E-01	6.88E-01	39	Glutathione metabolism
8	8.45E-01	6.90E-01	8.27E-01	5.21E-01	91	Basal transcription factors
8	8.35E-01	1.19E+00	5.06E-01	7.76E-01	182	Bacterial invasion of epithelial cells
8	8.13E-01	4.51E-01	7.39E-01	4.88E-01	56	Arachidonic acid metabolism
8	7.41E-01	5.90E-01	6.69E-01	4.16E-01	41	N-Glycan biosynthesis
8	6.61E-01	1.10E+00	4.22E-01	8.29E-01	176	Carbohydrate digestion and absorption
7	1.22E+00	6.50E-01	9.09E-01	7.44E-01	27	Arginine and proline metabolism
7	1.06E+00	1.04E+00	7.12E-01	5.85E-01	5	Fructose and mannose metabolism
7	9.99E-01	1.27E+00	9.49E-01	1.14E+00	143	Cytosolic DNA-sensing pathway
7	9.74E-01	1.14E+00	6.11E-01	8.45E-01	71	Nicotinate and nicotinamide metabolism
7	8.87E-01	1.18E+00	6.56E-01	7.80E-01	196	Endometrial cancer
7	8.87E-01	7.79E-01	8.61E-01	8.13E-01	82	Metabolism of xenobiotics by cytochrome P450
7	8.62E-01	8.86E-01	4.18E-01	6.29E-01	40	Starch and sucrose metabolism
7	8.21E-01	5.72E-01	5.44E-01	5.45E-01	200	Basal cell carcinoma
7	7.71E-01	9.63E-01	7.45E-01	5.94E-01	113	SNARE interactions in vesicular transport
7	7.27E-01	1.00E+00	5.18E-01	5.35E-01	45	Amino sugar and nucleotide sugar metabolism
6	1.28E+00	1.90E+00	9.61E-01	1.73E+00	202	Bladder cancer
6	1.21E+00	6.18E-01	1.19E+00	4.30E-01	2	Citrate cycle (TCA cycle)

Supplemental Table 4. Array-revealed Average Gene Expression Values for Members of Various Molecular Pathways

Number of Genes in Pathway	Average (No Omentectomy; 6 vs. 0 Mo)	Average (Yes Omentectomy; 6 vs. 0 Mo)	Average (No Omentectomy; 12 vs. 0 Mo)	Average (Yes Omentectomy; 12 vs. 0 Mo)	Pathway Number	Pathway
6	1.19E+00	6.51E-01	9.14E-01	5.93E-01	10	Fatty acid metabolism
6	1.12E+00	7.92E-01	8.20E-01	4.05E-01	170	Vasopressin-regulated water reabsorption
6	1.04E+00	7.63E-01	9.80E-01	7.59E-01	29	Tyrosine metabolism
6	9.87E-01	8.30E-01	9.36E-01	7.60E-01	153	Circadian rhythm - mammal
6	9.04E-01	7.86E-01	5.98E-01	6.78E-01	127	Hedgehog signaling pathway
6	8.17E-01	6.79E-01	8.13E-01	7.31E-01	97	Base excision repair
6	7.38E-01	9.61E-01	7.44E-01	6.01E-01	95	Protein export
6	7.04E-01	8.84E-01	7.33E-01	7.48E-01	92	DNA replication
5	1.34E+00	1.12E+00	1.49E+00	1.20E+00	211	Graft-versus-host disease
5	1.14E+00	6.73E-01	9.46E-01	5.31E-01	23	Valine, leucine and isoleucine degradation
5	1.14E+00	1.10E+00	9.20E-01	8.76E-01	199	Thyroid cancer
5	1.14E+00	1.19E+00	1.37E+00	1.14E+00	152	Intestinal immune network for IgA production
5	1.07E+00	3.07E-01	5.44E-01	3.98E-01	84	Drug metabolism - other enzymes
5	1.04E+00	4.30E-01	8.74E-01	4.87E-01	15	Steroid hormone biosynthesis
5	1.01E+00	9.74E-01	8.23E-01	9.34E-01	20	Alanine, aspartate and glutamate metabolism
5	9.92E-01	6.71E-01	8.52E-01	5.92E-01	55	Ether lipid metabolism
5	9.59E-01	6.14E-01	4.03E-01	7.53E-01	76	Retinol metabolism
5	8.82E-01	4.16E-01	6.08E-01	4.41E-01	172	Collecting duct acid secretion
5	7.86E-01	9.34E-01	5.04E-01	4.55E-01	158	Taste transduction
5	5.25E-01	9.15E-01	3.50E-01	9.09E-01	53	Glycosylphosphatidylinositol(GPI)-anchor biosynthesis
4	1.39E+00	2.88E+00	1.61E+00	2.70E+00	181	Prion diseases
4	1.30E+00	8.40E-01	1.01E+00	6.89E-01	28	Histidine metabolism
4	9.89E-01	6.39E-01	9.83E-01	4.63E-01	65	Propanoate metabolism
4	8.80E-01	4.29E-01	5.91E-01	8.34E-01	77	Porphyrin and chlorophyll metabolism
4	8.76E-01	5.19E-01	9.75E-01	9.51E-01	21	Glycine, serine and threonine metabolism
4	8.53E-01	5.64E-01	7.66E-01	6.23E-01	58	alpha-Linolenic acid metabolism
4	8.46E-01	1.52E-01	1.10E+00	1.23E-01	208	Autoimmune thyroid disease
4	8.37E-01	2.93E-01	7.30E-01	2.18E-01	212	Primary immunodeficiency
4	8.26E-01	4.10E-01	6.13E-01	3.83E-01	179	Amyotrophic lateral sclerosis (ALS)
4	7.67E-01	9.83E-01	7.29E-01	4.86E-01	100	Homologous recombination
4	7.63E-01	4.30E-01	4.94E-01	4.57E-01	51	Glycerolipid metabolism
4	4.89E-01	9.76E-01	2.69E-01	3.40E-01	101	Non-homologous end-joining
4	4.63E-01	9.41E-01	4.84E-01	9.43E-01	60	Glycosphingolipid biosynthesis - lacto and neolacto series
3	1.53E+00	1.30E+00	1.37E+00	9.49E-01	3	Pentose phosphate pathway
3	1.34E+00	6.06E-01	1.11E+00	2.55E-01	31	Tryptophan metabolism
3	1.22E+00	6.99E-01	1.06E+00	8.23E-01	30	Phenylalanine metabolism
3	1.19E+00	8.49E-01	7.50E-01	4.44E-01	114	Regulation of autophagy
3	1.09E+00	9.88E-01	8.43E-01	9.23E-01	4	Pentose and glucuronate interconversions
3	1.08E+00	5.84E-01	9.26E-01	6.48E-01	22	Cysteine and methionine metabolism
3	1.05E+00	1.36E+00	9.64E-01	7.87E-01	1	Glycolysis / Gluconeogenesis
3	1.02E+00	7.54E-01	8.32E-01	6.52E-01	24	Valine, leucine and isoleucine biosynthesis
3	9.77E-01	1.59E-01	8.58E-01	3.91E-01	64	Glyoxylate and dicarboxylate metabolism
3	9.25E-01	5.18E-01	5.56E-01	3.04E-01	43	O-Glycan biosynthesis
3	9.09E-01	1.07E-01	1.07E+00	8.14E-02	167	Type I diabetes mellitus
3	9.09E-01	1.07E-01	1.07E+00	8.14E-02	210	Allograft rejection
3	8.98E-01	1.25E+00	9.33E-01	9.02E-01	13	Primary bile acid biosynthesis
3	8.88E-01	5.77E-01	6.73E-01	6.62E-01	57	Linoleic acid metabolism
3	8.76E-01	5.46E-01	8.13E-01	5.58E-01	33	beta-Alanine metabolism
3	8.40E-01	1.07E+00	9.49E-01	1.16E+00	79	Nitrogen metabolism
3	8.34E-01	7.38E-01	5.91E-01	5.97E-01	35	Selenoamino acid metabolism
3	8.23E-01	5.88E-01	6.63E-01	4.54E-01	26	Lysine degradation
3	8.22E-01	9.01E-01	5.44E-01	8.71E-01	168	Maturity onset diabetes of the young
3	7.88E-01	4.18E-01	8.17E-01	5.51E-01	67	One carbon pool by folate
3	7.84E-01	1.04E+00	5.48E-01	1.01E+00	171	Proximal tubule bicarbonate reclamation
3	7.81E-01	9.13E-01	5.65E-01	5.68E-01	125	Dorso-ventral axis formation
3	7.22E-01	1.13E+00	7.16E-01	7.38E-01	99	Mismatch repair
3	3.80E-01	8.50E-01	5.03E-01	7.60E-01	126	Notch signaling pathway
2	1.25E+00	7.64E-01	9.77E-01	4.51E-01	139	Renin-angiotensin system
2	1.15E+00	8.84E-01	8.31E-01	7.90E-01	90	RNA polymerase

Supplemental Table 4. Array-revealed Average Gene Expression Values for Members of Various Molecular Pathways

Number of Genes in Pathway	Average (No Omentectomy; 6 vs. 0 Mo)	Average (Yes Omentectomy; 6 vs. 0 Mo)	Average (No Omentectomy; 12 vs. 0 Mo)	Average (Yes Omentectomy; 12 vs. 0 Mo)	Pathway Number	Pathway
2	1.03E+00	1.01E+00	8.05E-01	7.59E-01	75	Folate biosynthesis
2	9.57E-01	3.77E-01	1.07E+00	3.60E-01	207	Asthma
2	8.94E-01	5.61E-01	8.80E-01	4.28E-01	62	Glycosphingolipid biosynthesis - ganglio series
2	8.94E-01	9.70E-01	6.25E-01	5.87E-01	159	Phototransduction
2	7.05E-01	9.65E-01	5.62E-01	3.13E-01	49	Glycosaminoglycan biosynthesis - keratan sulfate
2	6.83E-01	6.16E-01	1.76E-01	8.56E-01	7	Ascorbate and aldarate metabolism
2	6.06E-01	1.61E+00	9.03E-01	1.05E+00	85	Biosynthesis of unsaturated fatty acids
1	1.34E+00	5.47E-01	1.07E+00	2.52E-01	73	Biotin metabolism
1	1.19E+00	3.19E-02	5.74E-01	1.69E-01	36	Cyanoamino acid metabolism
1	1.12E+00	5.44E-01	9.68E-01	8.05E-01	32	Phenylalanine, tyrosine and tryptophan biosynthesis
1	1.01E+00	3.55E-01	2.79E-01	1.22E-02	18	Caffeine metabolism
1	1.01E+00	1.31E-01	1.14E+00	1.96E-01	11	Synthesis and degradation of ketone bodies
1	1.01E+00	1.31E-01	1.14E+00	1.96E-01	66	Butanoate metabolism
1	1.01E+00	1.31E-01	1.14E+00	1.96E-01	78	Terpenoid backbone biosynthesis
1	8.36E-01	1.68E+00	3.91E-01	6.74E-01	6	Galactose metabolism
1	8.36E-01	1.68E+00	3.91E-01	6.74E-01	46	Butirosin and neomycin biosynthesis
1	6.63E-01	8.69E-02	1.00E+00	2.89E-01	61	Glycosphingolipid biosynthesis - globo series
1	4.60E-01	7.70E-01	6.15E-01	1.19E+00	14	Ubiquinone and other terpenoid-quinone biosynthesis
1	3.83E-01	1.19E+00	1.51E-01	8.73E-01	59	Sphingolipid metabolism
1	1.96E-01	8.85E-01	6.72E-01	1.17E+00	70	Vitamin B6 metabolism
1	1.77E-01	3.31E-01	2.01E-01	1.48E+00	80	Sulfur metabolism
1	1.48E-01	5.69E-01	4.94E-02	1.08E+00	48	Glycosaminoglycan biosynthesis - chondroitin sulfate
1	1.48E-01	5.69E-01	4.94E-02	1.08E+00	50	Glycosaminoglycan biosynthesis - heparan sulfate
